# Shotgun sequence-based metataxonomic and predictive functional profiles of *Pe poke*, a naturally fermented soybean food of Myanmar

**DOI:** 10.1371/journal.pone.0260777

**Published:** 2021-12-17

**Authors:** Jyoti Prakash Tamang, Pynhunlang Kharnaior, Priyambada Pariyar, Namrata Thapa, Ni Lar, Khin Si Win, Ae Mar, Nyo Nyo

**Affiliations:** 1 Department of Microbiology, DAICENTER (DBT-AIST International Centre for Translational and Environmental Research) and Bioinformatics Centre, School of Life Sciences, Sikkim University, Gangtok, Sikkim, India; 2 Department of Zoology, Biotech Hub, Nar Bahadur Bhandari Degree College, Sikkim University, Tadong, Sikkim, India; 3 Department of Industrial Chemistry, University of Mandalay, Mandalay, Myanmar; 4 Department of Geography, University of Mandalay, Mandalay, Myanmar; Dr. H.S. Gour Central University, INDIA

## Abstract

*Pe poke* is a naturally fermented sticky soybean food of Myanmar. The present study was aimed to profile the whole microbial community structure and their predictive gene functionality of *pe poke* samples prepared in different fermentation periods viz. 3 day (3ds), 4 days (4ds), 5 days (5ds) and sun-dried sample (Sds). The pH of samples was 7.6 to 8.7, microbial load was 2.1–3.9 x 10^8^ cfu/g with dynamic viscosity of 4.0±1.0 to 8.0±1.0cP. Metataxonomic profile of *pe poke* samples showed different domains viz. bacteria (99.08%), viruses (0.65%), eukaryota (0.08%), archaea (0.03%) and unclassified sequences (0.16%). *Firmicutes* (63.78%) was the most abundant phylum followed by *Proteobacteria* (29.54%) and *Bacteroidetes* (5.44%). *Bacillus thermoamylovorans* was significantly abundant in 3ds and 4ds (*p*<0.05); *Ignatzschineria larvae* was significantly abundant in 5ds (*p*<0.05), whereas, *Bacillus subtilis* was significantly abundant in Sds (*p* <0.05). A total of 172 species of *Bacillus* was detected. In minor abundance, the existence of bacteriophages, archaea, and eukaryotes were also detected. Alpha diversity analysis showed the highest Simpson’s diversity index in Sds comparable to other samples. Similarly, a non-parametric Shannon’s diversity index was also highest in Sds. Good’s coverage of 0.99 was observed in all samples. Beta diversity analysis using PCoA showed no significant clustering. Several species were shared between samples and many species were unique to each sample. In KEGG database, a total number of 33 super-pathways and 173 metabolic sub-pathways were annotated from the metagenomic Open Reading Frames. Predictive functional features of *pe poke* metagenome revealed the genes for the synthesis and metabolism of wide range of bioactive compounds including various essential amino acids, different vitamins, and enzymes. Spearman’s correlation was inferred between the abundant species and functional features.

## Introduction

**Fig 1:**
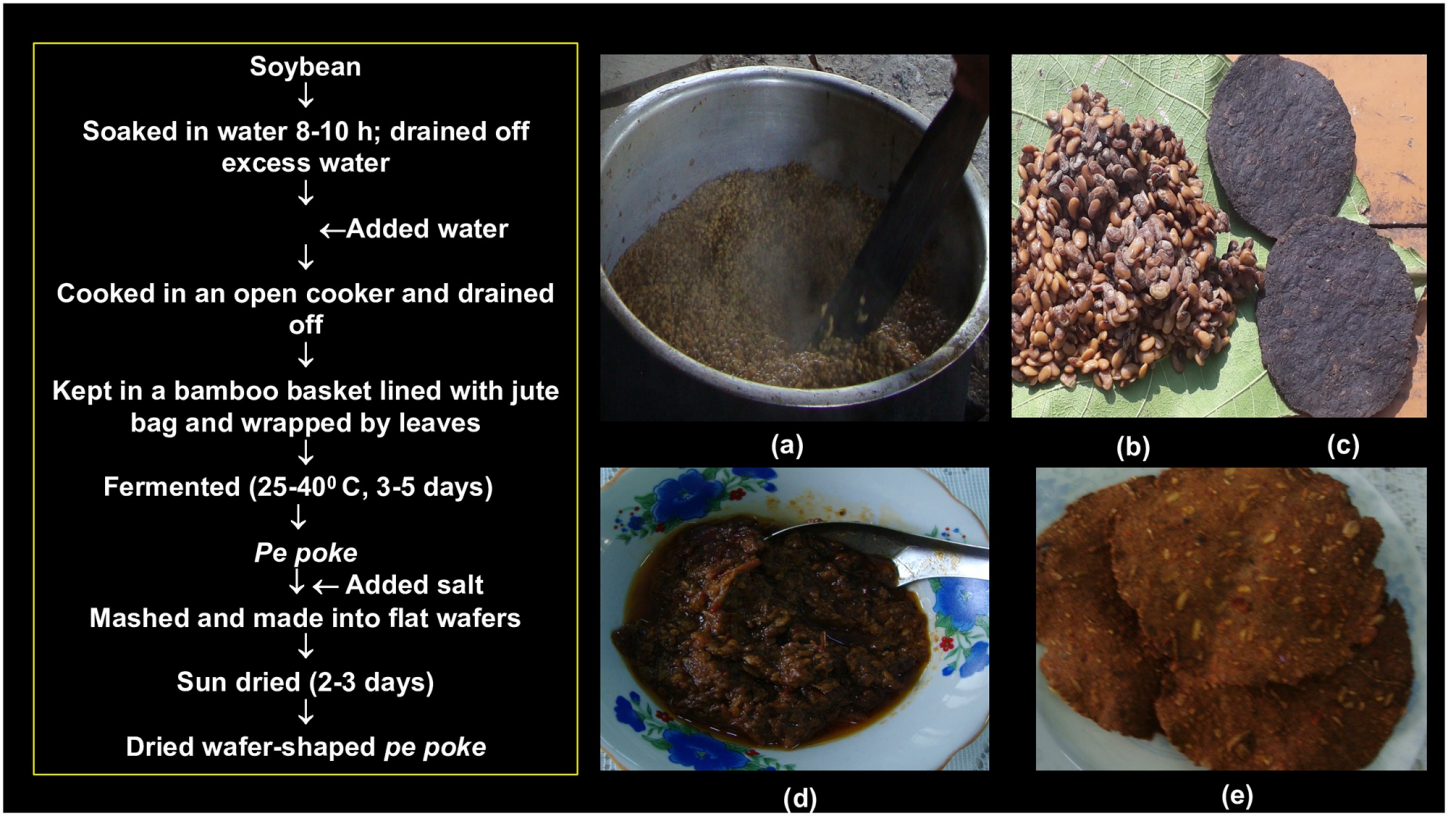
Traditional method of preparation of *pe poke* in Myanmar, (a) Boiling of soybeans; (b) freshly fermented *pe poke*; (c) sun-dried wafer-shaped *pe poke*; (d) *pe poke* curry; and (e) fried *pe poke* fritters.

The community-specific ethnic fermented foods have been centre of interest for their unique gastronomy as well as colossal microbial diversity [1]. Myanmar has several ethnic fermented foods and beverages including fermented soybeans, which have been traditionally prepared and consumed by more than 135 different ethnic communities [2]. Among fermented foods, traditional methods of fermentation of locally grown soybean is an ancient practice mostly seen in North-western regions of Myanmar bordering with North East states of India and North Western parts of Myanmar bordering with Northern Thailand. Both mould-fermented and bacterial-fermented soybean are prepared and consumed widely in Myanmar [3]. *Pe poke* is an ethnic fermented soybean food of northern Myanmar. There is no historical documentation of origin of *pe poke* in Myanmar, however, it is believed that soybean has been introduced to Myanmar from Yunnan province of China [4]. During traditional method of preparation of *pe poke*, soybeans are soaked in water overnight, dewatered, boiled and wrapped in leaves, and are kept in a warm place for natural fermentation of 3–5 days (Fig 1a and 1b). Sometimes, freshly prepared *pe poke* is mashed with addition of salt and hot pepper, shaped as flat wafers, and are sun dried (Fig 1c). Some people prefer to eat *pe poke* immediately after fermentation and make into a typical Burmese-style cuisine as a side dish (Fig 1d) and fried fritters (Fig 1e) with boiled rice in main meal. This is mostly observed in the North-western regions of Myanmar bordering with India, where similar types of sticky fermented soybean foods are prepared such as *hawaijar* in Manipur, *bekang* in Mizoram, *peruyaan* and *peron namsing* in Arunachal Pradesh and *axone* or *aakhone* in Nagaland states of India [5]. Whereas, in the North-eastern regions of Myanmar bordering with Thailand, freshly prepared *pe poke* is made into flat wafers, and are sun dried, which is similar to *thua nao* of Northern Thailand [6]. *Pe poke* is one of the delicacies in the diets of ethnic people of Myanmar, however, the consumption of *pe poke* among younger generation is declining. Traditionally prepared *pe poke* is sold in local markets by marginal farmers in many regions of Myanmar. *Pe poke* is similar to other sticky fermented soybean foods of Asia such as *kinema* of India, Nepal and Bhutan, *natto* of Japan, *thua nao* of Thailand, *cheonggukjang* of Korea, *douchi* of Yunnan province of China and *sieng* of Laos [7, 8].

Though *pe poke* is a popular ethnic food in Burmese gastronomy, but information on microbiology and nutritional aspects of *pe poke* is very rare, except few reports on *Bacillus subtilis* as the main fermenting bacterium in *pe poke* [9, 10]. It is necessary to understand the microbial community structure in *pe poke*, which is prepared by natural fermentation, moreover, such rare ethnic product has not been studied in details to profile its microbial community structure. We choose the shotgun metagenome sequence tool to profile the entire microbial community up to species, which is considered as one of the most reliable metataxonomic tools [11], that may sequence the genomes of untargeted cells in a microbial community to decode community structures including culturable and unculturable bacteria, yeasts, fungi, virus and archaea in food samples [12]. Hence, we aimed to study the metataxonomic of abundant domains in naturally fermented *pe poke* of Myanmar, prepared in different fermentation periods, by shotgun metagenomic sequencing method, supported by machine learning tools. Functional profiles of metagenomes were also predicted using the SqueezeMeta pipeline [13] and KEGG database [14]. We believe this is the first report of microbial community structures in naturally fermented *pe poke* by shotgun metagenome sequence tool.

## Materials and methods

### Sample collection and analysis of pH

Samples of *pe poke*, traditionally prepared in different fermentation periods viz. 3 days (3ds), 4 days (4ds), 5 days (5ds) and sun-dried sample (Sds) were collected from Pyinnolwin village in Mandalay state of Myanmar. Samples were collected in pre-sterile containers kept in ice-box carriers and transported to the Department of Industrial Chemistry, University of Mandalay and stored at 4°C. All samples were kept in ice-box carrier, by feeling with fresh ice in every 5–6 hours, till we reached to the Department of Microbiology, Sikkim University, Gangtok, India for immediate microbiological analysis. The pH of *pe poke* samples was determined by homogenizing 1 g of sample in physiological saline (0.85% sodium chloride, NaCl) and was measured using digital pH-meter (Orion 910003, Thermo Fisher Scientific, USA).

### Total viable count

Samples were coarsely crushed by a sterile spatula, and ten grams of the sample were homogenized with 90 mL of 0.85% physiological saline in a stomacher lab blender 40 (Seward, United Kingdom) for 5 min. The homogenized samples were serially diluted in the same diluents, and 1 mL of appropriate diluents was plated in plate count agar (M091S, HiMedia, India) using pour plate method and incubated at 37°C for 24 h. The number of colonies was counted as colony forming unit (cfu/g).


$$\text{cfu}/\text{g}=\left(\text{No}.\;\text{of}\;\text{colonies}\;\text{x}\;\text{dilution}\;\text{factor}\right)/\text{volume}\;\text{of}\;\text{inoculum}\;\text{taken}$$

### Measurement of viscosity

The dynamic viscosity of *pe poke* samples was determined using the method described by Ratha and Jhon [15]. Thirty grams of samples were mixed with 30 mL of distilled water and subjected to vigorous shaking in a conical flask (250mL) for 30 min. The slimy part was collected and 30 mL of its aliquot (100 rpm at 20°C) was measured for dynamic viscosity in centipoise (cP) using a viscometer (DV1MRVTJ0, Brookfield AMETEK, MA, USA). The experiment was done in triplicate sets.

### Genomic DNA extraction

Ten grams of coarsely crushed samples of *pe poke* were homogenised in Stomacher (400 Circulator, Seward, UK) with 90 mL of sterile 0.1 M phosphate buffer saline (pH 6.4) for 5 min. After homogenization, the homogenate was filtered and the filtrate was used for the extraction of genomic DNA using the Nucleospin^®^ Food DNA kit (MACHEREY-NAGEL GmbH & Co. KG, Duran, Germany) as per the manufacturer’s protocol. Concentration of DNA was then quantified using spectrophotometer (Eppendorf, USA). The quality of DNA was checked in 0.8% agarose gel electrophoresis and visualized using Gel Doc EZ imager (BioRad, USA).

### Metagenomics sequencing and library preparation

*Pe poke* metagenome library preparation for long reads sequencing was performed by following the method of Sevim et al. [16]. The 10 μg of DNA was used to create the ONT (Oxford Nanopore Technologies) library. The generated DNA fragments was sheared using Covaris g-tubes (Covaris Inc., Woburn, MA USA) and DNA was repaired using NEBNext FFPE (Formalin-Fixed, Paraffin-Embedded) Repair Mix (New England BioLabs, Ipswich, MA USA) according to the manufacturer’s instructions. AMPure XP beads (62 μl) were added to the FFPE-repair reaction and incubated at room temperature for 30 min on a Hula mixer, followed by two washes with 70% ethanol. Beads were then resuspended with 93 μl of nuclease free water and incubated for 30 min at room temperature on a Hula mixer; 90 μl of the eluate was then transferred to a clean 1.5 mL Eppendorf tube.

The fragmented and repaired DNA underwent end repair and A-tailing using the NEBNExt End Repair/dA-Tailing Module (New England BioLabs) following manufacturer’s protocol: The reaction volume was doubled to 120 μl, incubation was performed at 20°C for 20 min and at 65°C for 20 min. AMPure XP beads (120 μl) were added to the end-prep reaction and incubated for 30 min at room temperature on a Hula mixer, followed by two washes with 70% ethanol. Beads were then resuspended in 31 μl of nuclease free water and incubated for 30 min at room temperature on a Hula mixer; 61 μl of the eluate was then transferred to a clean 1.5 mL Eppendorf tube. The resulting DNA was quantified using the Qubit HS DNA kit.

The resulting DNA ligation and clean-up were performed using the SQK-LSK108 kit (Oxford Nanopore Technologies, Oxford, United Kingdom) following manufacturer’s instructions. The ligation reaction was incubated at room temperature for 10 min and then overnight at 4°C. The ligated samples were purified using 40 μl of AMPure XP beads, incubated for 30 min at room temperature on a Hula mixer followed by two washes using the kit-provided wash buffer. The beads were resuspended in 15 μl of the kit-provided elution buffer and then incubated for 30 min at room temperature on a Hula mixer; 15 μl of the eluate was then transferred to a clean 1.5 mL tube and quantified using the Qubit HS DNA kit. The library was then sequenced on a MinION using the R9 flow cell sequencing chemistry and were processed using the MinKNOW software (v1.13.1).

### Bioinformatics analysis

#### Metataxonomic

Raw data derived from MinION (TM) ONT (Oxford Nanopore Technologies) in fast5 format was converted into a fastq files using poretools v0.6.0 for the bioinformatics analysis of *pe poke* metagenome [17]. After conversion, the quality of the fastq files were then examined using NanoPlot [18] and generated the corrected-assembled data via canu-assembler [19]. A database derived from GenBank containing millions of protein sequences from bacteria, archaea, viruses, fungi, and other microbial eukaryotes was downloaded within Kaiju via *kaiju-makedb -s nr_euk*. Taxonomy assignment of the assembled quality sequences was performed using a taxonomical pipeline, Kaiju [20] in which a default “greedy algorithm” was used to map the sequences against the database [21]. A cut-off for a minimum required match length (*m* = 11, default), minimum match score of 80 (*s* = 80) and the *E*-value (*E* = 0.05) was set to filter the mismatches. Filtering of query sequences containing low-complexity regions was performed to avoid false positive taxon assignments that may cause by bogus matches or other sequencing noises [20]. Amino acid substitution model was performed with a total score for each match calculated as in amino acid sequence alignment and ranked a multiple match and taxon classification from the database. After translation of ORFs into a set of amino acid fragments, we ranked the fragments by their BLOSUM62 (BLOcks SUbstitution Matrix) score and start the database search with the highest scoring fragment [22]. The fragments were searched backwards via BWT (Burrows-Wheeler transform) algorithm against database [23] and the higher score of fragments in the search was used for classifying the reads and outputs the taxon identifier [20].

#### Predictive functional features

Predictive functional features of the metagenome was performed on Quality-filtered contigs using the SqueezeMeta pipeline version 1.3.0 [13]. After importing of data, the contigs of <500bp were removed using prinseq [24], followed by gene prediction of the assembled using Prodigal (v2.6.2) [25] and the predicted genes were searched for homologies against the functional databases using DIAMOND, computational tool for the alignment of sequencing reads against a protein reference database [26]. After running the DIAMOND, the method assigned as functions to each Open Reading Frames (ORF) was carried out using the fun3 method (fun3 method produced functional assignments to compare genes sequences against the functional database) for Clusters of Orthologous Groups/Non-supervised Orthologous Groups (COGs/NOGs) using evolutionary genealogy of genes: Non-supervised Orthologous Groups (eggNOG) database [27] and Kyoto Encyclopedia of Genes and Genomes (KEGG) database [14]. In the process of analysis, the highest-scoring ORFs in the contig with an exceeding of 30% (default) were considered for annotation [13]. Best hits gene annotations were further processed for pathways prediction and enzyme classification [28]. Metabolic pathways assigned against the KEGG database was categorised in three level: high-level function (Level 1), lower-level function (Level 2) and the sub-pathways (Level-3) [29]. Enzymes involved in lysine biosynthesis, alanine, aspartate, glutamate, glycine, serine, threonine metabolism, pentose phosphate pathways, galactose metabolism and phosphotransferase system were mapped against the KEGG pathways database [30].

### Statistical analysis

#### Inter species diversity

Significance among the abundant species (>1%) was calculated using Fisher exact test [31]. Inter species diversity of *pe poke* metagenome was performed among the samples (3ds, 4ds, 5ds and Sds) using Tukey’s test in IBM SPSS v20.0 [32]. Shared and unique species was calculated using InteractiVenn: a web-based tool for the analysis [33].

#### Alpha and beta diversity

Differences of species distribution among the samples was measured using diversity indices, Simpson and Shannon diversity index was calculated, and principal coordinate analysis (PCoA) was plotted based on Bray-Curtis dissimilarities using PASTv4 (Paleontological Statistics Software Package) [34]. Furthermore, UPGMA (Unweighted Pair Group Method with Arithmetic mean) hierarchical clustering was performed for similarity analysis based on microbial communities which was compared between the samples and support the result observed in beta diversity [35].

#### Predictive functional features

The predictive functional profiles of *pe poke* were tested using Tukey’s test to check the inter pathways distribution among the samples (3ds, 4ds, 5ds and Sds) using IBM SPSS v20.0 [32]. UPGMA hierarchical clustering was also performed to compare the functional distribution among the samples [35]. Heatmap visualization of functional profiles, level-1 and level-2 was carried out using a web tool: ClustVis [36]. Correlation between the major species and functional features was performed by a non-parametric Spearman’s rank correlation using IBM SPSS v20.0 (Statistical Package for the Social Sciences), and a network-based visualization was generated using MetScape v3.1.3 in Cytoscape v3.8.2.

## Results

The pH of *pe poke* samples was 7.6 to 8.7 with the microbial load of 2.1–3.9 x 10^8^ cfu/g. The dynamic viscosity of samples was 4.0±1.0 to 8.0±1.0 cP (centipoise).

### Microbial community

**Fig 2:**
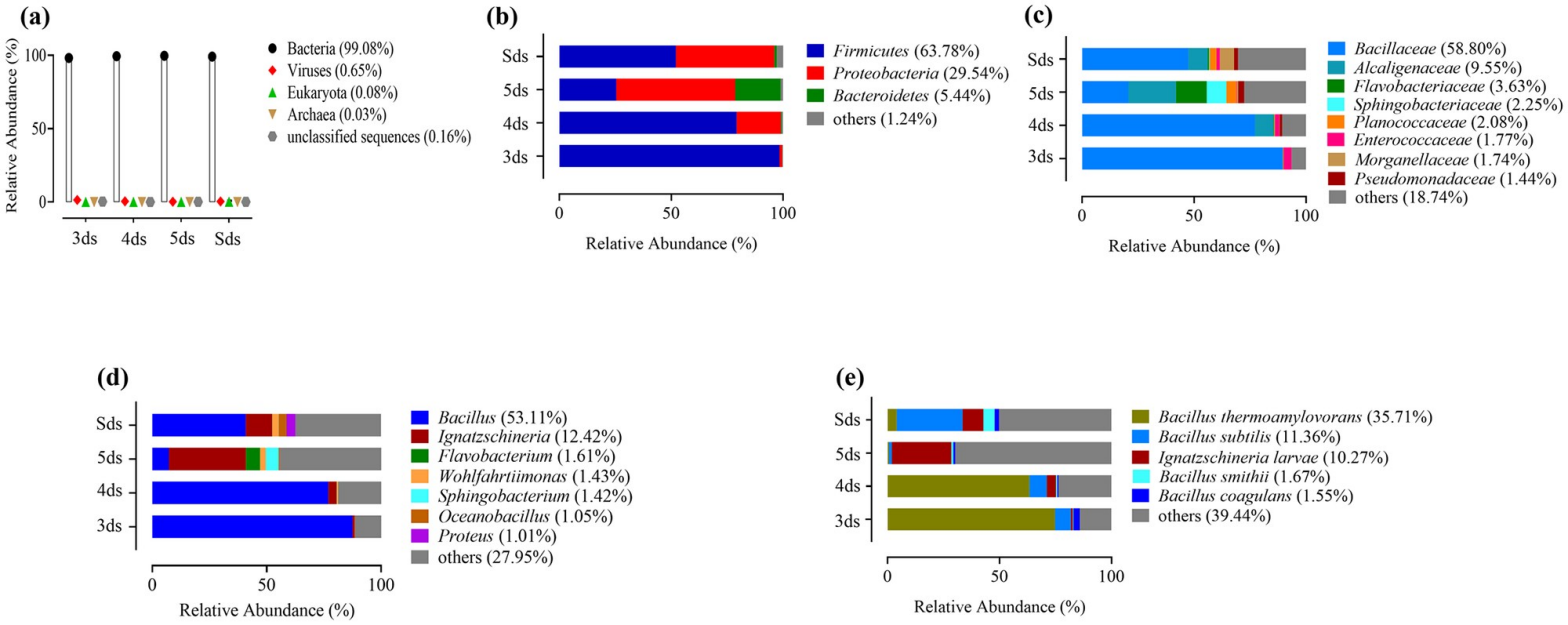
Relative abundance of microbial communities in *pe poke* (a) domain, (b) phyla, (c) family, (d) genera and (e) species.

A total of 1085311 reads were obtained from all samples of *pe poke* with an average of 271327.7 reads per sample. Average length of the reads was found 901.5. Total number of bases recovered from the samples were 314,801,023 bases for 3ds, 176,709,193 bases for 4ds, 372,945,882 bases for 5ds and 41,455,352 bases for Sds, respectively. Shotgun metagenomic sequence analysis of *pe poke* metagenome showed different domains viz., bacteria, archaea, viruses and eukaryotes with 46 phyla, 328 families, 718 genera and 1475 species. Taxonomic classification at domain level revealed the abundance of bacteria (99.08%) followed by viruses (0.65%), eukaryota (0.08%), archaea (0.03%) and unclassified sequences (0.16%) (Fig 2a). At bacterial phylum level, *Firmicutes* was the most abundant phylum followed by *Proteobacteria*, *Bacteroidetes* and others (1.24%) (Fig 2b) including phyla with a relative abundance of <1% (S1 Table). *Bacillaceae* was the most abundant family followed by *Alcaligenaceae*, *Flavobacteriaceae*, *Sphingobacteriaceae*, *Planococcaceae*, *Enterococcaceae*, *Morganellaceae*, *Pseudomonadaceae* (Fig 2c) and others detected at <1% abundance (S2 Table). Taxonomic annotation revealed the abundance of *Bacillus* (53.11%) at genus level followed by *Ignatzschineria*, *Flavobacterium*, *Wohlfahrtiimonas*, *Sphingobacterium*, *Oceanobacillus*, *Proteus* (Fig 2d) and others detected at <1% abundance (S3 Table). At species level, *Bacillus thermoamylovorans* was the most abundant species in *pe poke* samples, followed by *B*. *subtilis*, *Ignatzschineria larvae*, *B*. *smithii*, *B*. *coagulans* (Fig 2e) and others detected at <1% abundance (S4 Table). No phylum, family, genus and species with a relative abundance of >1% were detected from other domains viz. archaea, viruses and eukaryote in *pe poke* metagenomes.

**Fig 3:**
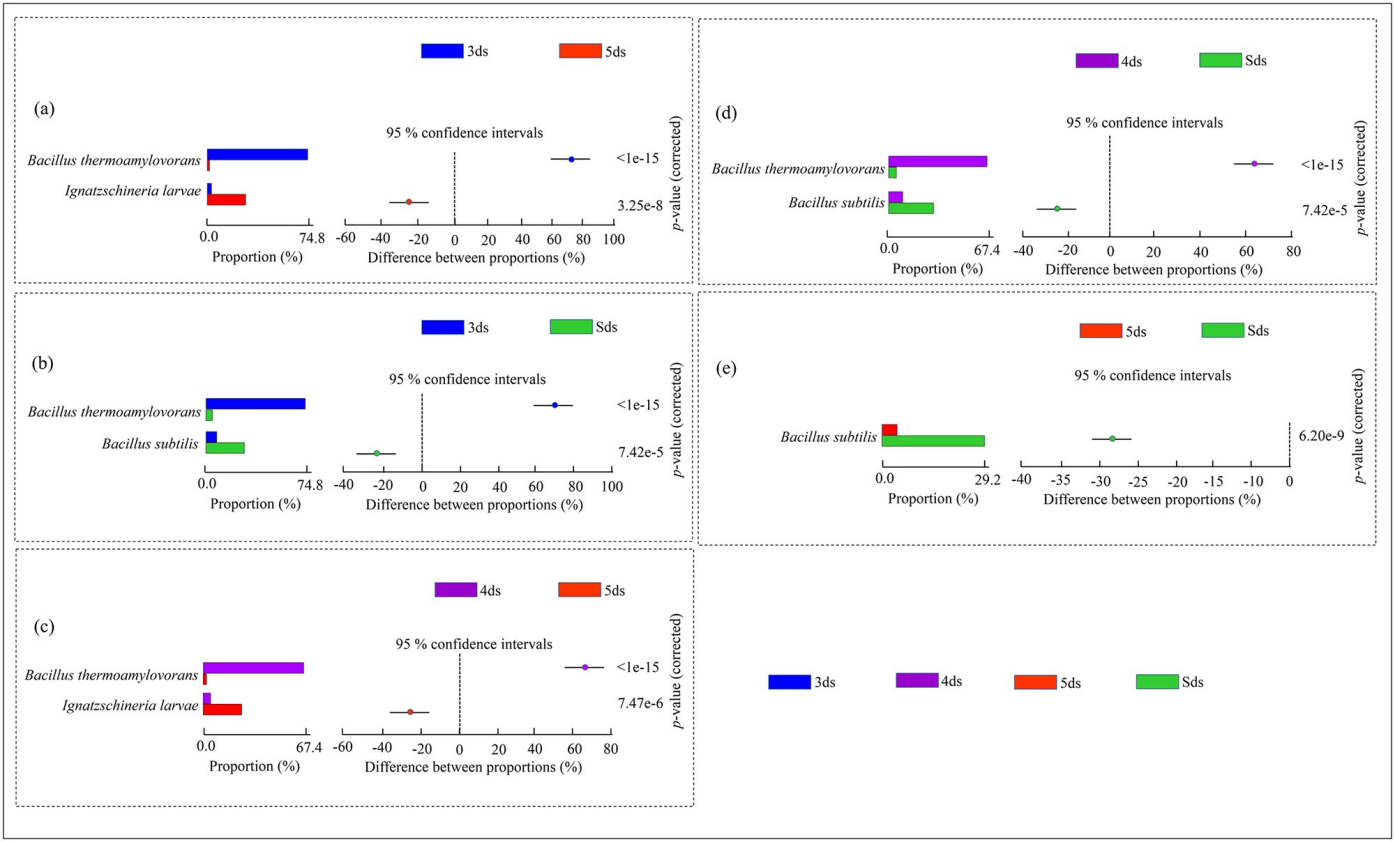
Fisher exact test performed at species level to observe the significant differences and similarities of predominant species comparing between the samples.

*Firmicutes* was found abundant phylum in samples of 3ds, 4ds and Sds, whereas *Proteobacteria* was observed abundant in 5ds. In comparison among 3ds, 4ds and Sds, *Bacillus thermoamylovorans* was significantly (*p*<0.05) abundant in 3ds (Fig 3a and 3b) and *Ignatzschineria larvae* was significantly (*p*<0.05) abundant in 5ds (Fig 3a); whereas, *B*. *subtilis* was significantly (*p*<0.05) abundant in Sds (Fig 3b). Similarly, when compared among 4ds, 5ds and Sds, *B*. *thermoamylovorans* was significantly (*p*<0.05) abundant in the 4ds (Fig 3c and 3d), *Ignatzschineria larvae* in 5ds (Fig 3c) and *Bacillus subtilis* was significantly (*p*<0.05) abundant in Sds (Fig 3d and 3e). In *pe poke* metagenomic analysis, a total of 172 species of *Bacillus* were detected, out of which the abundant species with a relative abundance of >1% were *B*. *thermoamylovorans*, *B*. *subtilis*, *B*. *smithii* and *B*. *coagulans* (S5 Table). Besides *Bacillus*, lactic acid bacteria (LAB) were also detected with a cumulative abundance of 1.78%, which included 94 species (S6 Table). Apart from bacterial domain, taxonomic annotation also revealed 16 species of archaea (S7 Table), 31 species of eukaryotes i.e., 3 species of yeasts, 15 species of filamentous moulds and 13 species of other eukaryotes (S8 Table), and 33 species of virus (bacteriophages), out of which 21 species were *Bacillus* phages (S9 Table).

### Diversity indices

**Fig 4:**
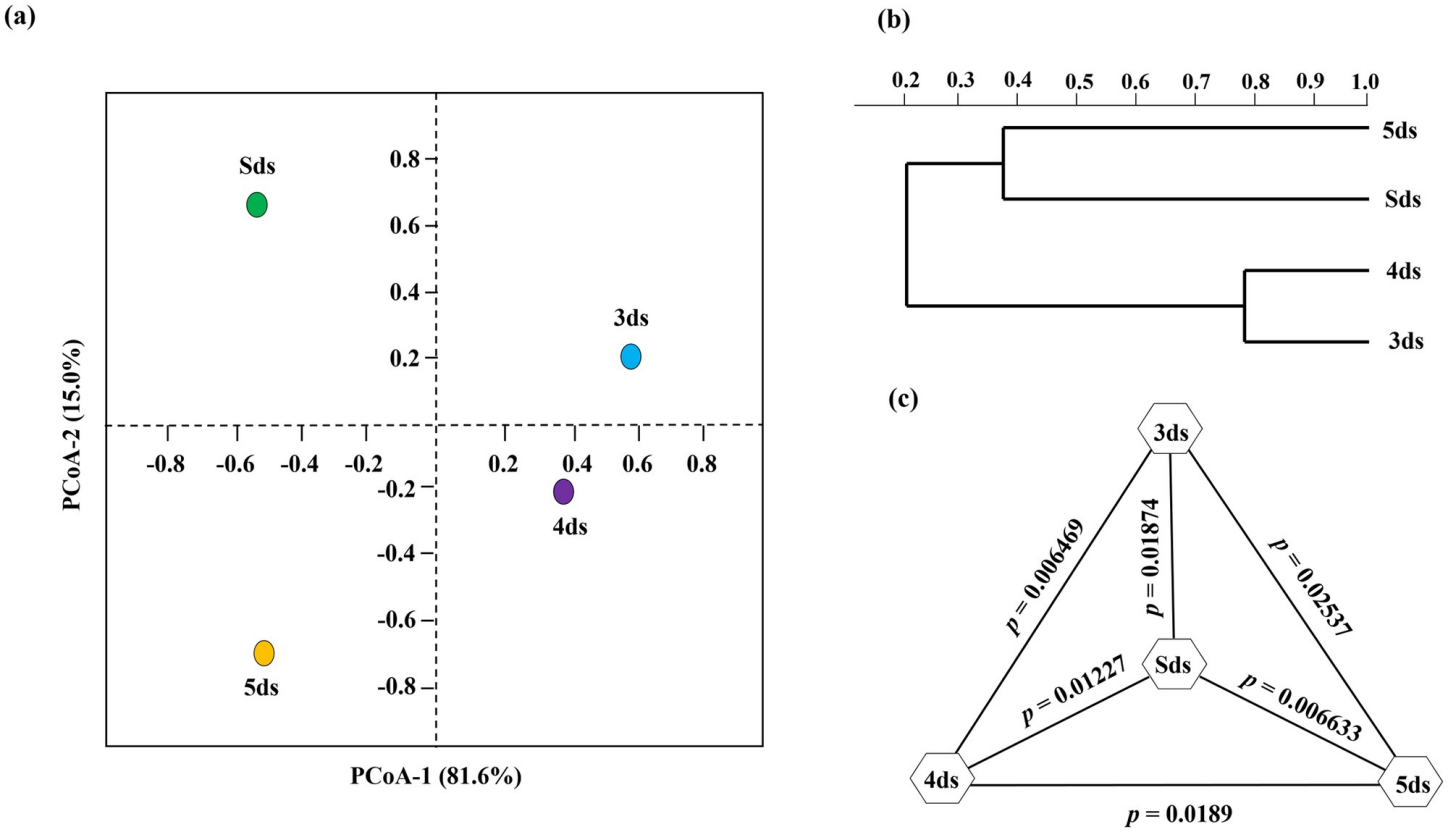
(a) PCoA of *pe poke* metagenomes based on microbial species. (b) UPGMA showed no clustering between samples, and (c) one-way ANOVA Tukey’s test revealed the significant differences among samples (*p* <0.05).

Alpha diversity analysis showed the highest Simpson’s diversity index in Sds comparable to other samples (Table 1). Similarly, a non-parametric Shannon’s diversity index was also highest in Sds (Table 1). Good’s coverage of 0.99 was observed in all the samples. Beta diversity analysis using PCoA (Fig 4a) and UPGMA (Fig 4b) showed no significant clustering. Statistically, in term of species abundance, the inter species diversity among the samples was calculated using Tukey’s test (Fig 4c) and a significant difference was observed between 3ds and 4ds (*p* = 0.006469), 5ds (*p* = 0.02537) and Sds (*p* = 0.01874). Similarly, 4ds was significantly different from 5ds (*p* = 0.0189) and Sds (*p* = 0.01227), and 5ds was significantly different from Sds (*p* = 0.006633).

**Table 1:** Indices of alpha diversity among the four samples of *pe poke*.

Indices	Samples			
	3ds	4ds	5ds	Sds
Simpson (1-D)	0.43	0.62	0.86	0.91
Shannon	2.25	3.14	5.46	5.99
Goods Coverage	0.99	0.99	0.99	0.99

### Shared and unique species

**Fig 5:**
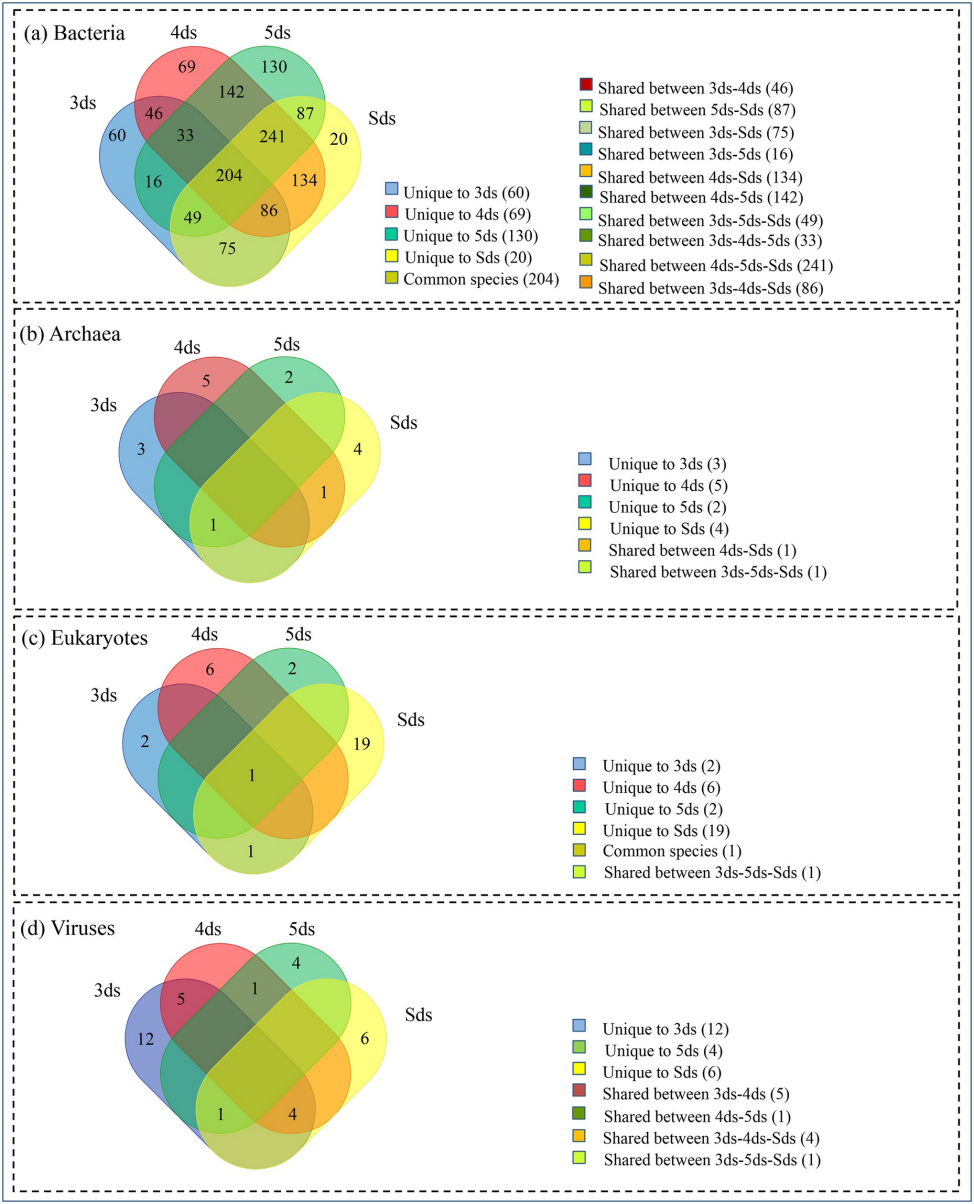
Shared, unique and common species in *pe poke* metagenome represented by InteractiVenn.

Metataxonomic annotation of *pe poke* metagenome revealed a huge diversity of microbial communities including shared and unique species (Fig 5a–5d). Based on different domains that have been classified via taxonomic classification, we observed about 204 bacterial core species were common in all the samples (Fig 1a). Additionally, the unique species were 60, 69, 130 and 20 in 3ds, 4ds, 5ds and Sds, respectively (S10 Table). Among archaeal species, no core species was found common to all samples. *Methanobacterium formicicum* was shared between 3ds, 5ds and Sds, and *Halapricum salinum* was shared between 4ds and Sds (Fig 1b). The unique species were 3, 5, 2 and 4 in 3ds, 4ds, 5ds and Sds, respectively (S11 Table). Among eukaryota, *Mucor ambiguus* was the core species commonly found in all samples. *Batrachochytrium dendrobatidis* shared between 3ds 5ds and Sds (Fig 1c) and the unique species were 2, 6, 2, 19 in the 3ds, 4ds, 5ds and Sds, respectively (S12 Table). In the category of viruses, no common species was observed. *Aeribacillus* phage AP45 was shared between 4ds and 5ds, and *Geobacillus* virus E3 was shared between 3ds, 5ds and Sds (Fig 5d). The unique species were 12, 4, 6 in 3ds, 5ds and Sds, respectively (S13 Table).

### Predictive functional features

**Fig 6:**
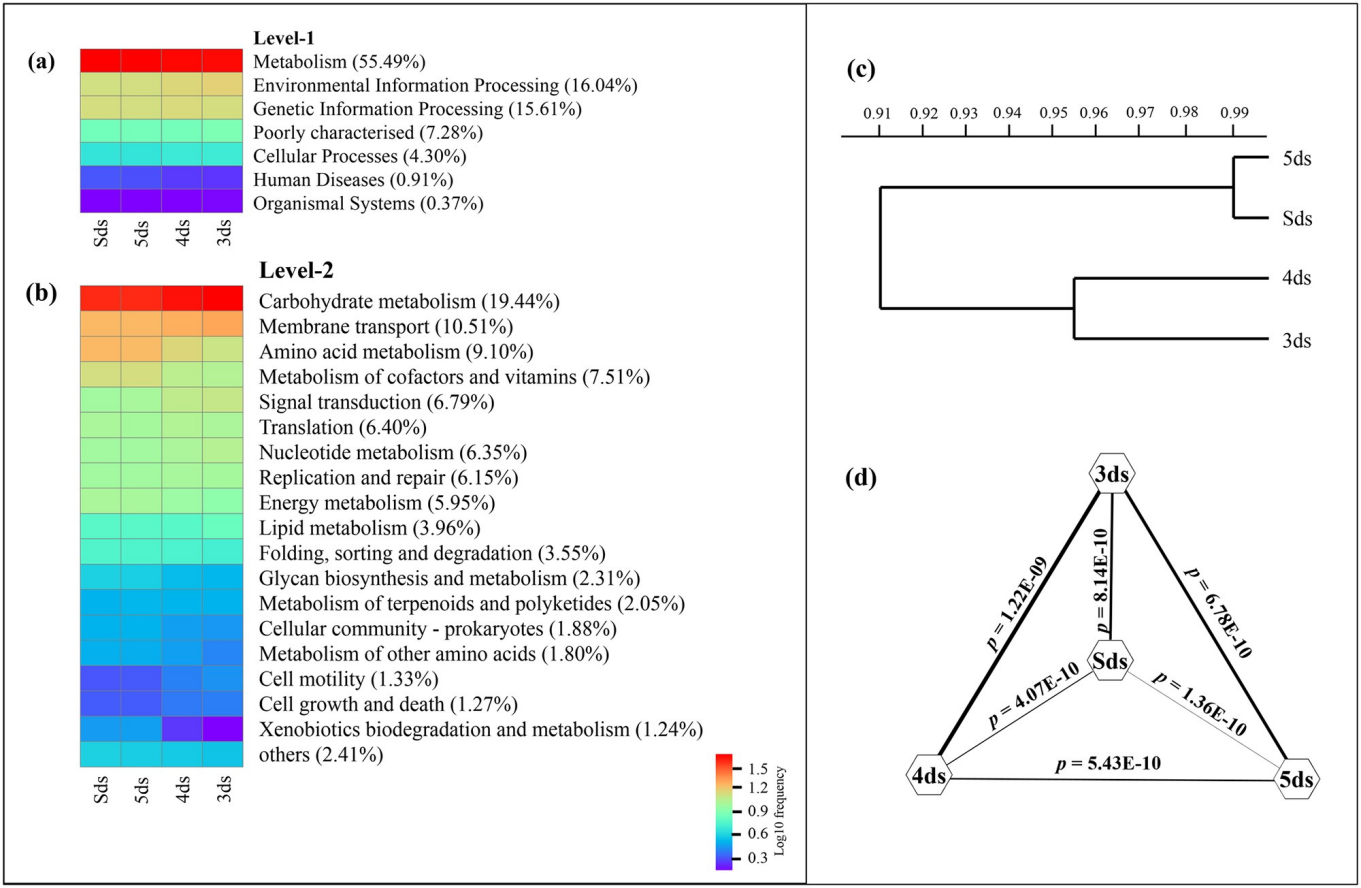
The abundant functional features in *pe poke* metagenome (a) Level-1 and (b) Level-2 with a relative abundance more than 1%. (c) UPGMA showed no clustering between samples and (d) significant differences among samples observed using one-way ANOVA Tukey’s test.

The mapping of metagenomic sequences against the databases of orthologous gene groups (COG and KO) revealed many enriched functional features. About 56% were assigned to COG functional genes and the remaining 44% ORFs were assigned to KEGG functional pathways. In COG annotation, general function prediction only was the abundant followed by DNA replication, recombination and repair, amino acid transport and metabolism, carbohydrate transport and metabolism, transcription, translation, ribosomal structure and biogenesis, inorganic ion transport and metabolism, energy production and conversion, cell envelope biogenesis, outer membrane (S14 Table). In KEGG database, a total number of 33 super-pathways and 173 metabolic sub-pathways were annotated from the metagenomic ORFs. At KO level-1, metabolism was the most abundant followed by environmental information processing, genetic information processing, cellular processes, human diseases, organismal systems and poorly characterised (Fig 6a). At KO level—2, the abundant functional prediction was carbohydrate metabolism followed by other metabolisms (Fig 6b) and super-pathways with relative abundance of <1% mapped against KEGG (S15 Table). Furthermore, at KO level-3, super-pathways with relative abundance of <1% mapped against KEGG showed genes related to ABC transporters was the most abundant followed by other predictive metabolic pathways (S16 Table). Based on the distribution of functional features, no clustering of samples was observed by performing the UPGMA analysis (Fig 6c). Tukey’s test was performed to check the significant differences of functional features between the samples (Fig 6d).

**Fig 7:**
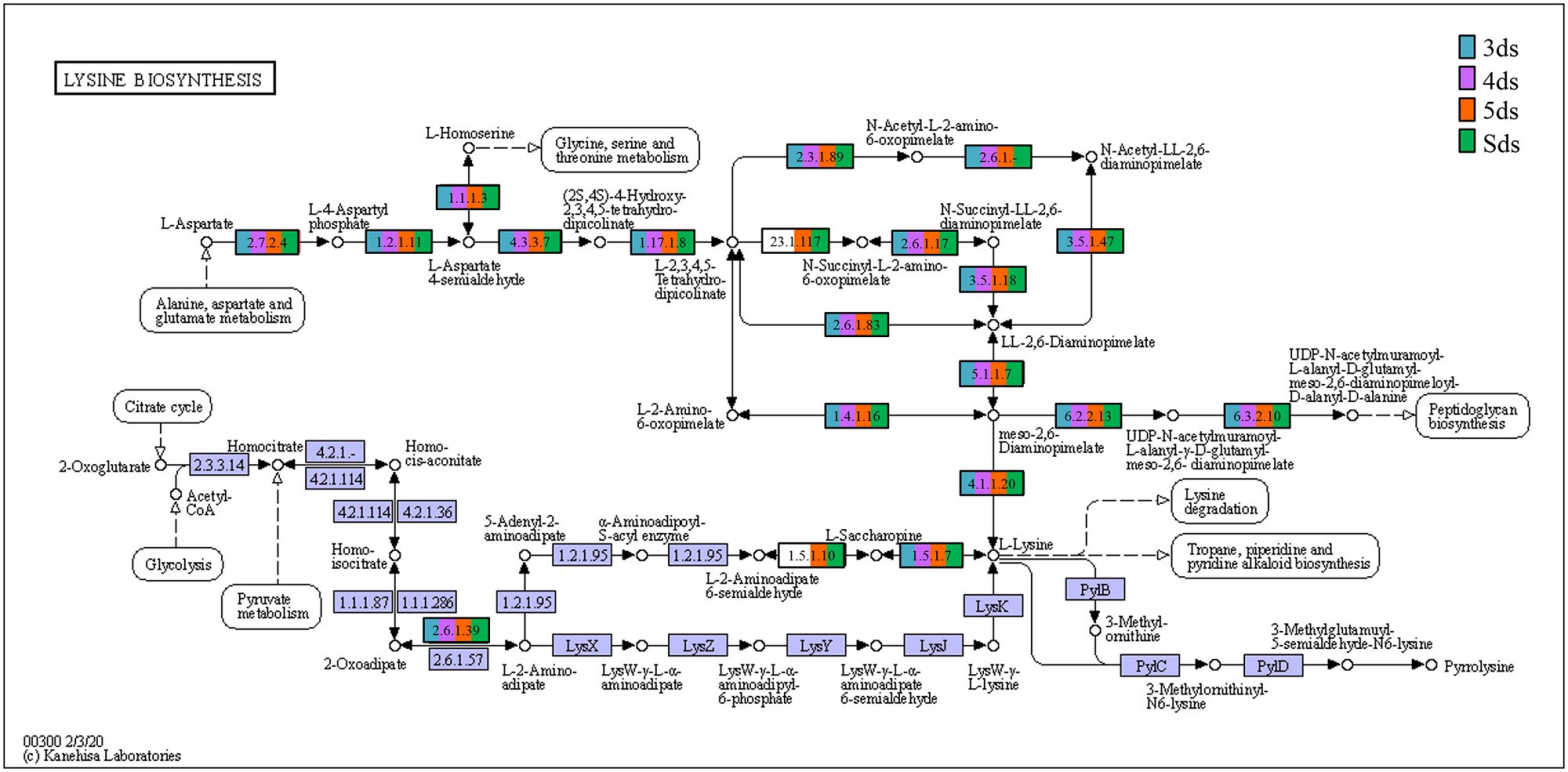
Enzymes involve in lysine biosynthesis detected in *pe poke* metagenome.

**Fig 8:**
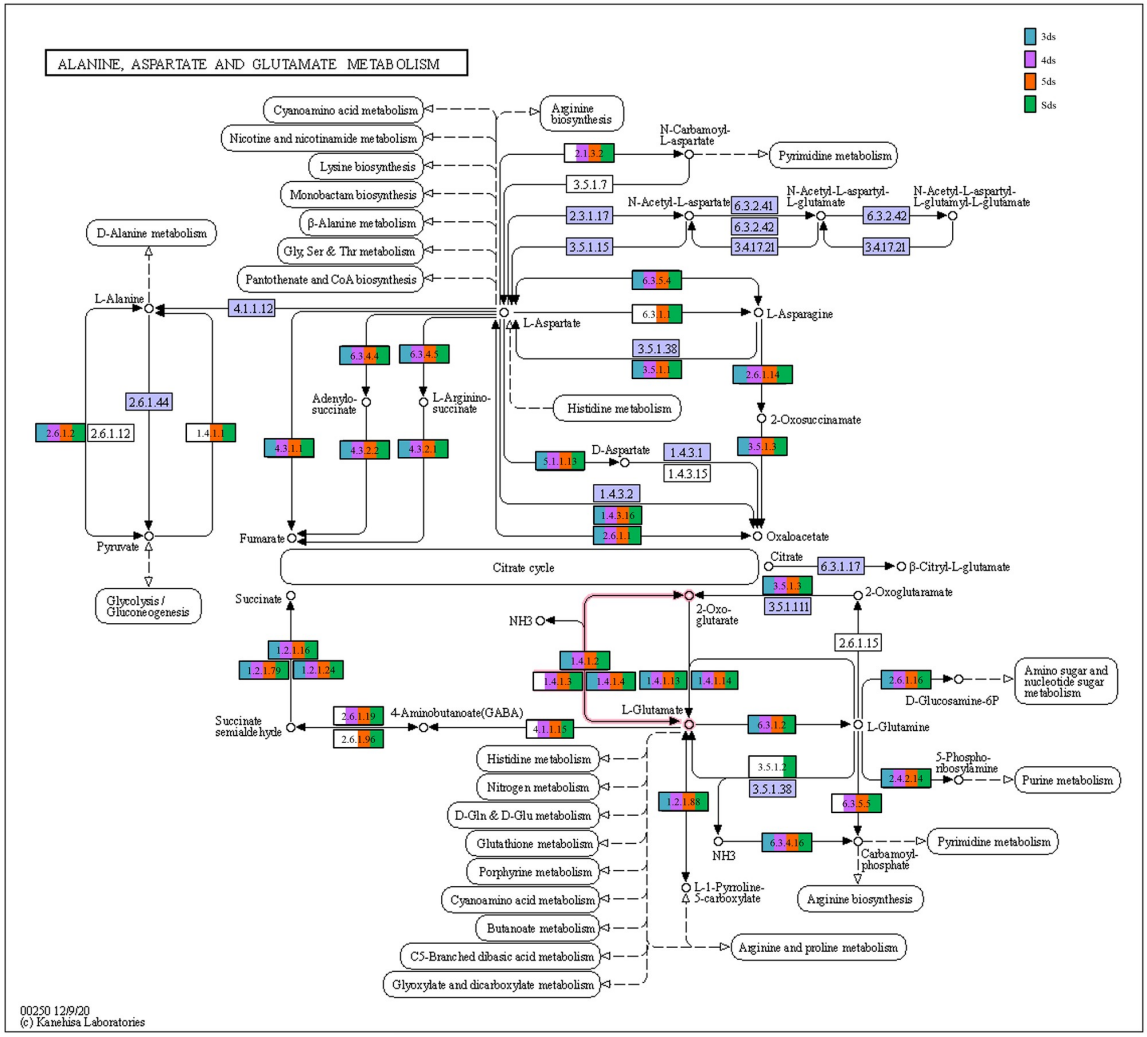
Enzymes involved in alanine, aspartate and glutamate metabolism and 4-aminobutanoic acid (γ-aminobutyric acid, or GABA) detected in *pe poke* metagenome.

**Fig 9:**
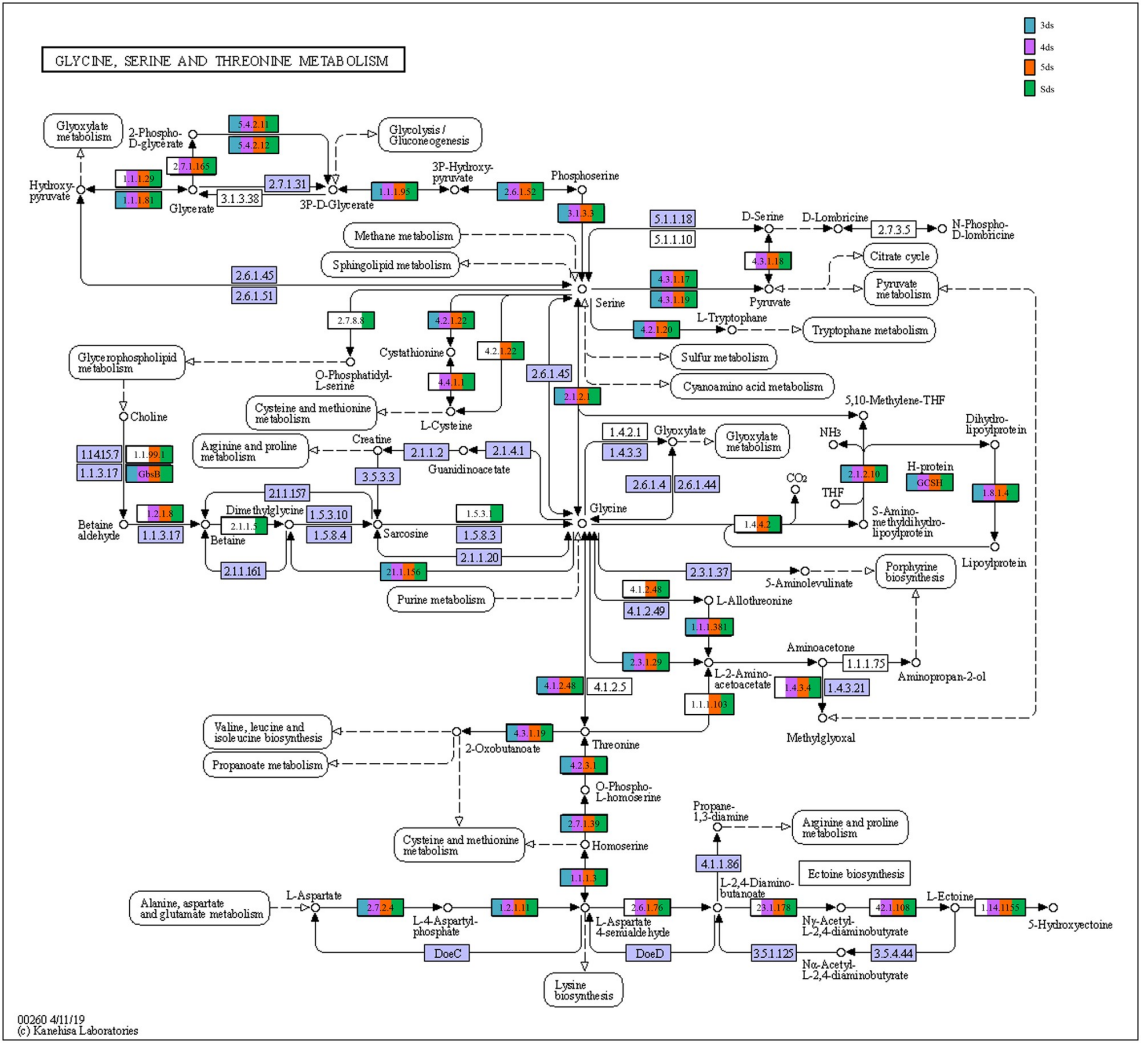
Enzymes involved in glycine, serine and threonine metabolism and ectoine biosynthesis detected in *pe poke* metagenome.

**Fig 10:**
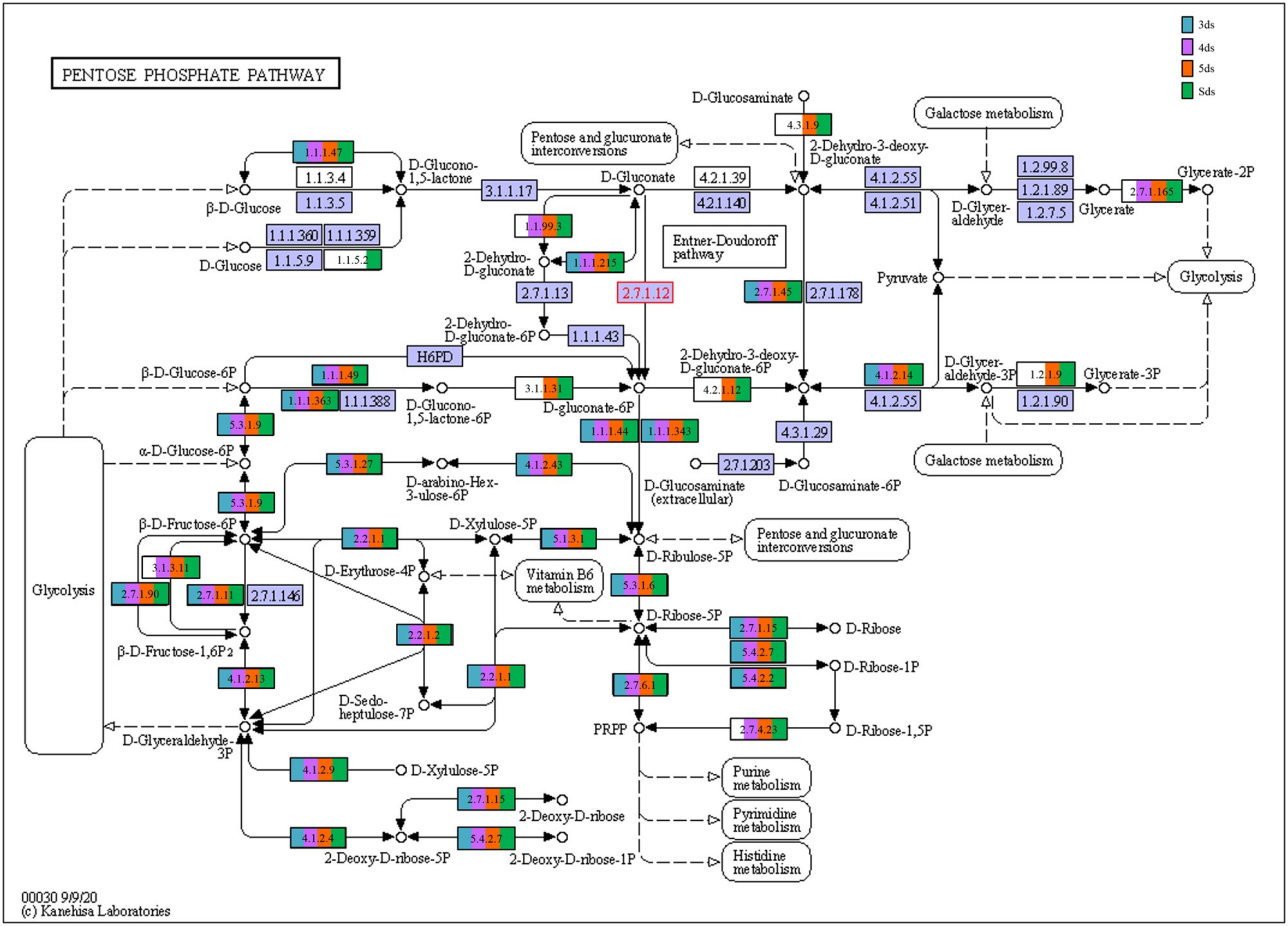
Enzymes involved in pentose phosphate pathways.

**Fig 11:**
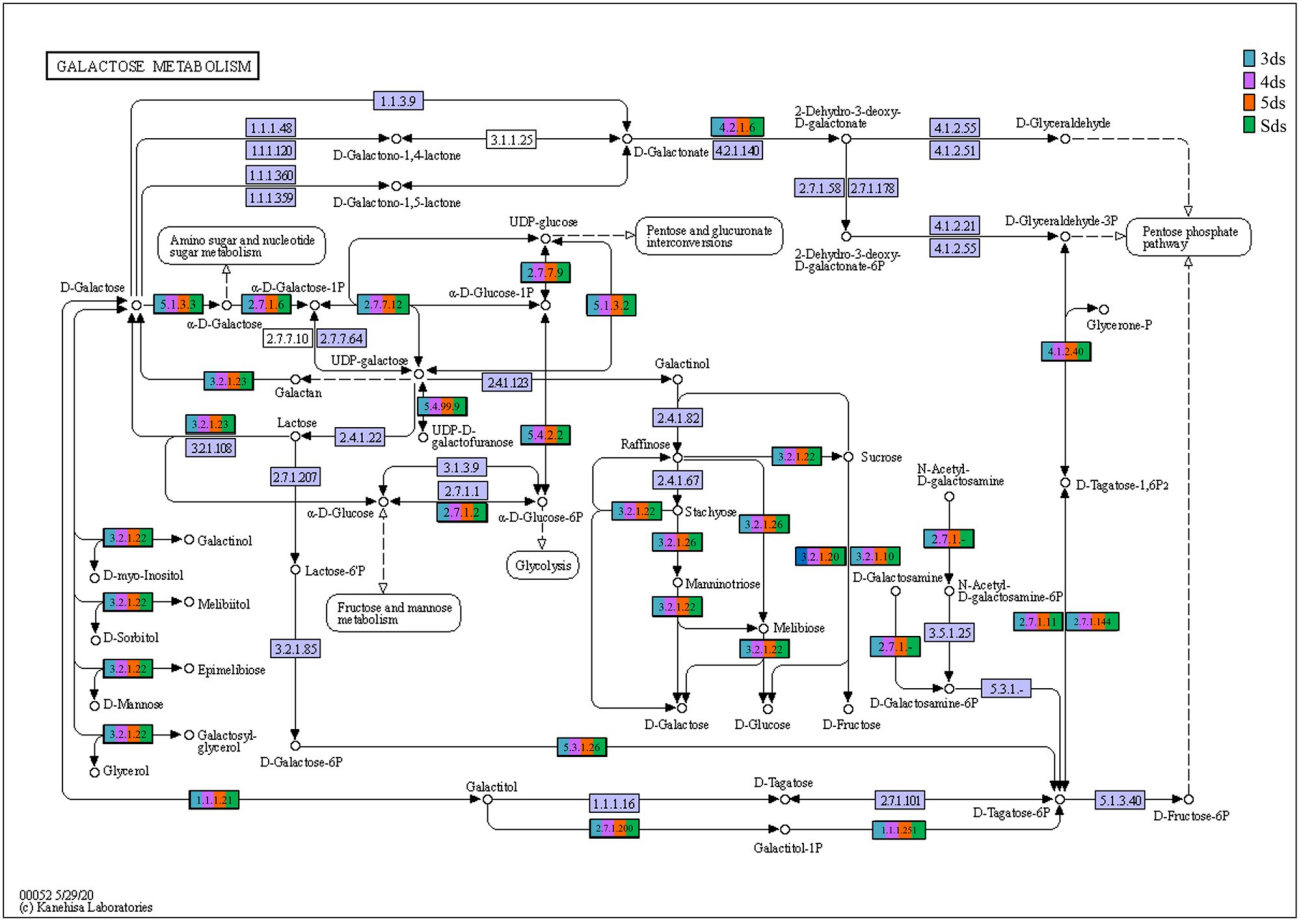
Enzymes involved in galactose metabolism.

In enzyme classification, we detected genes encoding for enzymatic activity such as protease, serine protease, amylase, lipase, galactosidase, glucosidase, glutamate decarboxylase and functions involve in poly-γ-glutamic acid biosynthesis (S17 Table). Predictive metabolic pathways were mapped against the KEGG pathways database such as lysine biosynthesis (Fig 7), alanine, aspartate and glutamate metabolism (Fig 8), glycine, serine and threonine metabolism (Fig 9), pentose phosphate pathways (Fig 10) and galactose metabolism (Fig 11). The predictive enzymes involved in different pathways were observed such as lysine metabolism (S18a Table), alanine, aspartate and glutamate metabolism and 4-aminobutanoic acid (γ-aminobutyric acid, or GABA) (S18b Table), glycine, serine, threonine metabolism and ectoine biosynthesis (S18c Table), pentose phosphate pathways (S18d Table) and galactose metabolism (S18e Table).

### Correlation between predominant species and predictive functions

**Fig 12:**
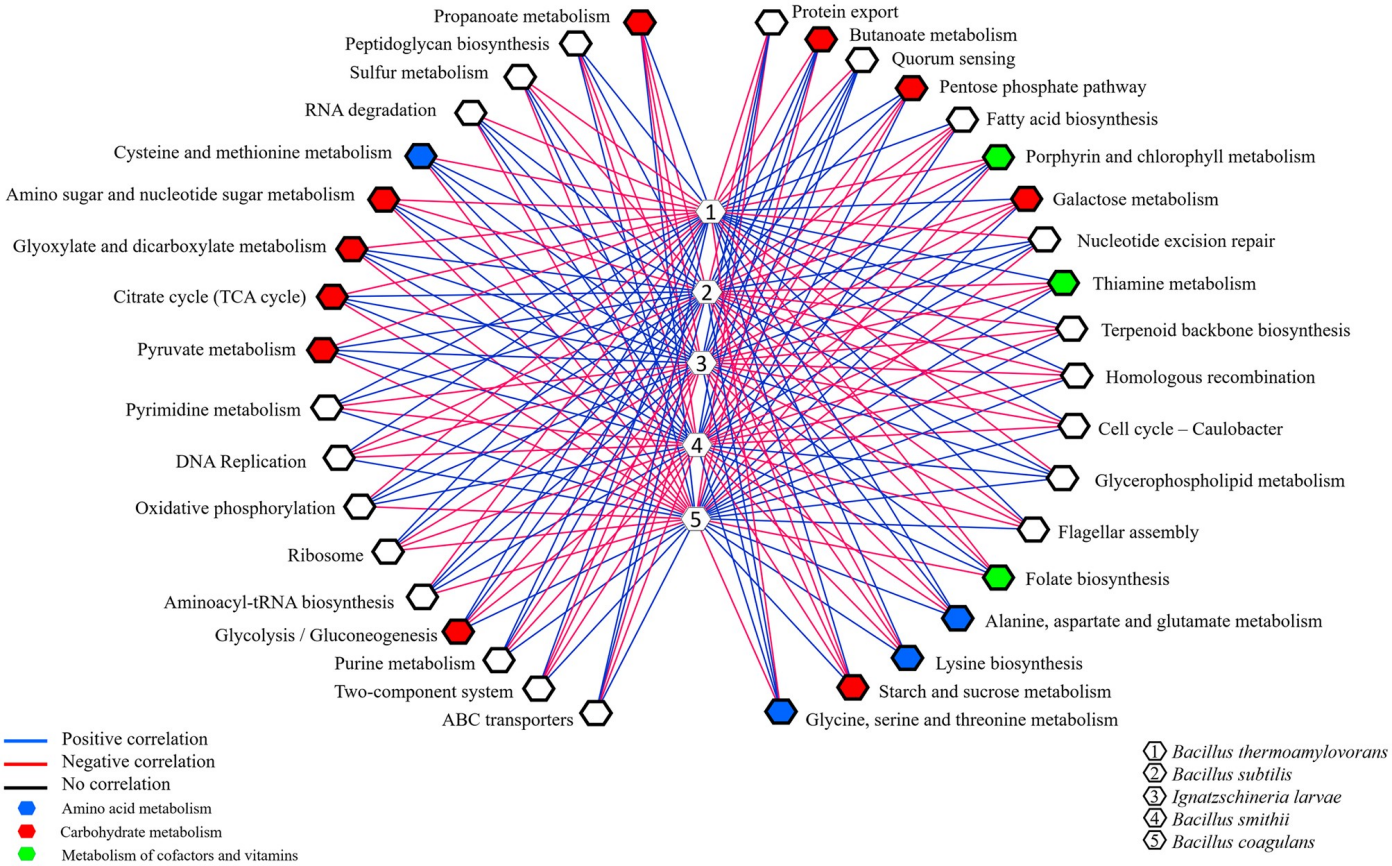
Spearman-s correlation was performed between the predominant species and functional features that has a relative abundance >1% using IBM SPSS (Statistical Package for the Social Sciences) Statistics v.20 and represented via correlation-based network.

Spearman’s correlation was inferred between the abundant species and functional features (Fig 12). *Bacillus thermoamylovorans*, *B*. *subtilis*, *B*. *smithii* and *B*. *coagulans* were positively correlated with alanine, aspartate and glutamate metabolism, pentose phosphate pathway, and glycolysis/gluconeogenesis. Lysine biosynthesis and galactose metabolism were positively correlated with *B*. *thermoamylovorans* and *B*. *coagulans*, whereas negatively correlated with *B*. *subtilis* and *B*. *smithii*. Glycine, serine and threonine metabolism and cysteine and methionine metabolism was positively correlated with *B*. *subtilis* and *B*. *smithii*, and negatively correlated with *B*. *thermoamylovorans* and *B*. *coagulans*. Among metabolism of cofactors and vitamins, *B*. *thermoamylovorans* and *B*. *coagulans* were positively correlated with thiamine metabolism; *B*. *smithii* with porphyrin and chlorophyll metabolism and *B*. *subtilis* with folate biosynthesis (Fig 12).

## Discussion

### Microbial community

*Pe poke* is an alkaline (pH 7.8–8.7) naturally fermented soybean food of Myanmar, which is prepared traditionally by ethnic Burmese people. Though *pe poke* is considered as a sticky fermented soybean food, however, the dynamic viscosity of samples was 8.0±1.0cP as compared with that of *natto*, a highly sticky Japanese fermented soybean with dynamic viscosity of >23cP [37]. The microbiological population of *pe poke*, as determined by cultural method, showed a viable load of 10^8^ cfu/g, indicating its richness in microbial diversity. Since fermentation periods during natural fermentation of *pe poke* vary from 3 to 5 days, we collected the samples fermented for 3 days, 4 days and 5 days, and also the sun-dried samples for profiling the microbial community using the shotgun metagenome sequence tool to know the abundant microbial domains with their predictive functional features. Bacteria were detected as the most abundant domain, and the least abundant domains were archaea, eukaryotes and viruses, which reflects the comprehensive general picture of the microbial communities of *pe poke*. The higher abundance of *Firmicutes* and the presence of *Proteobacteria*, *Bacteroidetes* and *Actinobacteria* in the minority groups were previously reported in other fermented soybean foods such as *kinema* of India, Nepal and Bhutan [8], *douchi* of China [38] and *da-jiang* of Korea [39]. *Bacillaceae* and *Bacillus* were reported in *pe poke* as the abundant family and genus, respectively. A colossal interspecies diversity of *Bacillus* with more than 172 species was detected in *pe poke* metagenomes by shotgun sequence tool. By cultural method, only *B*. *subtilis* was reported in *pe poke* [9, 10]. At species level, we observed the abundance of *B*. *thermoamylovorans* in 3ds and 4ds, *Ignatzschineria larvae* in 5ds, and *B*. *subtilis* in Sds sample, respectively. *B*. *thermoamylovorans* is a heat resistant [40] and amylolytic bacterium [41], which is reported in *cheonggukjang* [42], *kinema* [8] and *douchi* [43], and it may also involve in producing thermo-stable enzymes during fermentation at high temperatures [44]. *B*. *subtilis*, the second abundant species in *pe poke*, is one of the major bacterial species in many Asian fermented soybean foods [8, 45–47]. We also observed *B*. *coagulans*, which is resistance to high temperatures, and produces various enzymes applicable to food industry [48]. The abundance of *B*. *smithii* in *pe poke* metagenome was also previously reported in fermented soybean foods such as *tungrymbai* of Meghalaya state and *bekang* of Mizoram state of North-East India [46]. Abundance of *Bacillus* species indicates high proteolytic activity, amylase activity and lipase activity [49–52]. *Ignatzschineria larvae* was also found abundant in 5 days-*pe poke*, probably contaminated from flies [53], during prolonged fermentation under unhygienic condition. Some LAB were also detected in samples of *pe poke*, which may have beneficial antimicrobial activity against pathogenic bacteria [54].

*Myoviridae*, *Podoviridae* and *Siphoviridae* were the abundant families of viruses belonging to the order *Caudovirales* in *pe poke*. In fermented soybean food, bacteriophages have been reported to cause food spoilage [55] and the abnormal effect on products that may cause reduction of viscous poly-γ-glutamic acid in fermented soybean foods [56]. Bacteriophages may kill the beneficial starter, hamper the bacterial growth, delay fermentation process, yield low-quality, and lower down the bioactivities of the food product [57]. However, some suggested an alternative hypothesis that the presence of bacteriophages is considered to be a very useful therapy in reducing pathogenic bacteria in food products [58]. The presence of archaeal and eukaryotic species were in low abundances in *pe poke* metagenomes. Archaea contributes to development of taste, aroma flavour, dietary supplements, acetate production during fermentation, and even protect food from spoilage by yeasts [59]. Domain Eukarya consisted of yeasts, filamentous moulds, different species of algae, protozoa and parasites was detected in low abundances in *pe poke*. Filamentous moulds are known to contribute flavour in fermented soybean product [60] and possess high proteolytic activity [61].

Diversity index, which considers both number of species as well as relative abundance of each species for evaluating diversity [62], showed highest value for the Sds of *pe poke*, probably due to the duration of fermentation that may cause the changes in species abundance [63]. A goods’ coverage observed in our study indicates a maximum microbial diversity [64] in the samples. In beta diversity, we observed a discrete association among metagenome samples corroborated by PCoA plot, based on their taxonomic features, which may be due to the changes with fermentation time and environmental factors [39, 65]. Several unique and shared species were observed in different samples, probably due to abiotic factors or unusual associations among species from different domains [66].

We found that natural fermentation days of 3–4 days may be suitable for consumption of *pe poke* due to abundance of *B*. *thermoamylovorans* and *B*. *subtilis*, which are considered as safe fermenting bacteria in fermented foods [8, 67] comparable to 5 days *pe poke* with abundance of *Proteobacteria*, which contains several pathogenic bacteria [68].

### Predictive functional features

The predictive functional analysis of *pe poke* metagenome, mapped against KEGG database, suggested the abundance of metabolism including pathways for carbohydrate metabolism and amino acid metabolism. Abundance of genes related to carbohydrate metabolism (pentose phosphate pathway, and glycolysis) is important for microbial metabolism [69]. The genes for predictive enzymes such as α-glucosidase, α-galactosidase, and β-galactosidase were detected in galactose metabolism pathway in *pe poke*, essential for degradation of starch and oligosaccharides into simpler forms during fermentation [70]. Genes involved in the processing of lignocellulose were also detected in *pe poke* metagenome, which suggested that plants-derived carbohydrate act as the source of energy for aerobic (via tricarboxylic acid, TCA cycle) or anaerobic (fermentation) microbes [71]. It was also reported that β-glucosidase could be involved in the hydrolysis of cello-oligosaccharides [72], and biosynthesis of isoflavone glycosides [73], and also involved in digestion and hydrolysis of macromolecules present in soybean seeds during fermentation [74]. Genes related to glycine, serine and threonine metabolism detected in *pe poke*, may enhance the nutritional value of the product [8, 75]. The abundance of genes related to alanine, aspartate, glutamate metabolism in *pe poke* metagenome may contribute to the enhancement of taste and flavour of the product [76]. Folate biosynthesis, the key pathway of new therapies against infectious diseases caused by various microorganisms [77] was detected in *pe poke*. The positive correlation between *Bacillus subtilis* and folate biosynthesis was observed in *pe poke*, the key pathway of new therapies against infectious diseases [78] and also confers the protection against inflammation, cancer, anaemia, cardiovascular diseases [79].

Abundance of genes related to ABC transporters specific for peptides were detected in *pe poke* metagenomes, which may felicitate the uptake of di-/tripeptides [80]. The active role of the microbial population in the transformation of polysaccharide and short-chain carbohydrate in *pe poke* has been supported by the phosphotransferase system (PTS), the source of transport and phosphorylation of various sugar which forms mono/disaccharides, amino sugars, polyols, and other sugar derivatives [73].

In enzyme classification, we observed the presence of predictive enzymes involvement in the biosynthesis of lysine, alanine, aspartate, glutamate, glycine, serine and threonine, which enhance the nutritional value of the product [54]. Additionally, we also detected enzymes typically encoded by *ect*ABCD gene cluster of bacteria [81] that have an excellent function-preserving property [82]. Genes related to serine protease such as fibrinolytic enzymes were detected in *pe poke*, which may play as antithrombotic agents [83, 84]. Gene related to signal transduction system that regulates poly-γ-glutamic acid (PGA) synthesis [85, 86].

A positive correlation observed between *Bacillus* species and predictive amino acids metabolism indicates the ability to accumulate most of amino acids such as alanine, aspartate, glutamate, glycine, serine, and threonine that enhance the nutritional values in the product [87], and also contributes to taste perception and flavour enhancement [88]. A positive correlation between lysine biosynthesis and *B*. *thermoamylovorans* was detected in *pe poke*, which was also reported in *douchi* [89]. Lysine has several health promoting benefits to consumers [90]. *B*. *coagulans* showed positive correlation with biosynthesis of thiamine (vitamin B1), one of the major growth factors that promotes the growth of *B*. *coagulans* [91], similarly *B*. *coagulans* showed a positive correlation with galactose metabolism, where α- and β-galactosidases (detected in galactose metabolism pathway) can hydrolyse a non-digestible galactoside present in the food matrix [92]. *B*. *subtilis* showed positive correlation with predictive folate (vitamin B_9_) biosynthesis; *B*. *subtilis* is reported to harbour pathways component for folate (vitamin B_9_) production [79]. Though prediction of some pathways related to human disease were also observed, but their abundance were too low to make any significant impact.

## Conclusion

*Pe poke* is a popular traditional fermented soybean cuisine in the Burmese food culture, however, its microbiology and functional properties have not been studied in details, except few reports on *Bacillus* sp. Hence, we profiled the microbial community in samples of *pe poke*, which were naturally fermented for 3 days, 4 days, 5 days, respectively and also sundried *pe poke*, by shotgun metagenomic analysis. Colossal diversity of microbial communities in *pe poke* was observed. We found that natural fermentation days of 3–4 days may be suitable for consumption of *pe poke* due to abundance of *Bacillus thermoamylovorans* and *B*. *subtilis*. Several predictive biosynthesis of amino acids, vitamins and other bioactive compounds have been inferred indicting the functional properties of this unique Burmese fermented soybean food, and moreover, the information obtained from this study may help to sensitise the commercial producers and consumers aware on microbial community, the health benefits, hygiene and general safety in *pe poke*.

## Supporting information

S1 Table The minor phyla with a relative abundance of <1% detected in *pe poke*. (DOCX)

S2 Table The minor families with a relative abundance of <1% detected in *pe poke*. (DOCX)

S3 Table The minor genera with a relative abundance of <1% detected in *pe poke*. (DOCX)

S4 Table The minor species with a relative abundance of <1% detected in *pe poke*. (DOCX)

S5 Table The overall species of *Bacillus* detected in *pe poke*. (DOCX)

S6 Table The species of lactic acid bacteria detected in *pe poke*. (DOCX)

S7 Table Archaeal species detected in samples of *pe poke*. (DOCX)

S8 Table Eukaryotic species including yeasts, moulds, algae, protozoa and parasites detected in *pe poke*. (DOCX)

S9 Table Viral species detected in samples of *pe poke*. (DOCX)

S10 Table Shared and unique bacterial species detected in *pe poke*. (DOCX)

S11 Table Shared and unique archaeal species detected in *pe poke*. (DOCX)

S12 Table Shared and unique eukaryotic species detected in *pe poke*. (DOCX)

S13 Table Shared and unique viral species detected in *pe poke*. (DOCX)

S14 Table Clusters of Orthologous Groups (COGs) detected in *pe poke*. (DOCX)

S15 Table The relative abundance of <1% mapped against KEGG database at level-2 (Super-pathways). (DOCX)

S16 Table The relative abundance of <1% mapped against KEGG database at level-3 (Sub-pathways). (DOCX)

S17 Table Predictive enzyme classification detected in *pe poke*. (DOCX)

S18 Table Predictive enzymes classification involved in different pathways in *pe poke*: (a) Lysine biosynthesis; (b) Biosynthesis of alanine, aspartate and glutamate metabolism and 4-aminobutanoic acid (GABA); (c) Glycine, serine, threonine metabolism and ectoine biosynthesis; (d) Pentose phosphate pathways and (e) Galactose metabolism. (DOCX)
