# Supplementary material for: Shotgun sequence-based metataxonomic and predictive functional profiles of Pe poke, a naturally fermented soybean food of Myanmar
Source: PLoS One. 2021 Dec 17;16(12):e0260777. doi: 10.1371/journal.pone.0260777 (PMC8682898; doi:10.1371/journal.pone.0260777)
Supplement: S1 Table — (DOCX) [file pone.0260777.s001.docx]

**Supplementary Table 1.** The minor phyla with a relative abundance of <1% detected in *pe poke*.

| Sl. No. | Phyla | Relative abundance (%) | | | | Domain |
| --- | --- | --- | --- | --- | --- | --- |
|  |  | 3ds | 4ds | 5ds | Sds |  |
| 1 | *Actinobacteria* | 0.118367 | 0.252769 | 0.286519 | 1.018305 | Bacteria |
| 2 | *Cyanobacteria* | 0.009206 | 0.04186 | 0.067988 | 0.151534 | Bacteria |
| 3 | *Spirochaetes* | 0.015782 | 0.01932 | 0.029138 | 0.103043 | Bacteria |
| 4 | *Acidobacteria* | 0.005261 | 0.01288 | 0.019425 | 0.084859 | Bacteria |
| 5 | *Chloroflexi* | 0.006576 | 0.00483 | 0.009713 | 0.09092 | Bacteria |
| 6 | *Chytridiomycota* | 0.003946 | 0.00966 | 0 | 0.096981 | Eukaryota |
| 7 | *Deinococcus-Thermus* | 0.00263 | 0.01288 | 0.029138 | 0.054552 | Bacteria |
| 8 | *Euryarchaeota* | 0.010521 | 0.01288 | 0.024281 | 0.048491 | Archaea |
| 9 | *Fusobacteria* | 0.030249 | 0.00161 | 0.004856 | 0.048491 | Bacteria |
| 10 | *Chlamydiae* | 0.00263 | 0.01449 | 0.024281 | 0.042429 | Bacteria |
| 11 | *Tenericutes* | 0.003946 | 0.00966 | 0.019425 | 0.048491 | Bacteria |
| 12 | *Verrucomicrobia* | 0.006576 | 0.00483 | 0.014569 | 0.048491 | Bacteria |
| 13 | *Nitrospirae* | 0.006576 | 0 | 0.009713 | 0.054552 | Bacteria |
| 14 | *Chlorobi* | 0 | 0.00644 | 0.033994 | 0.030307 | Bacteria |
| 15 | *Planctomycetes* | 0.00263 | 0.00805 | 0.019425 | 0.024245 | Bacteria |
| 16 | *Ascomycota* | 0.001315 | 0.00483 | 0.004856 | 0.042429 | Eukaryota |
| 17 | *Aquificae* | 0 | 0.00322 | 0.019425 | 0.030307 | Bacteria |
| 18 | *Gemmatimonadetes* | 0.001315 | 0.00644 | 0.019425 | 0.018184 | Bacteria |
| 19 | *Thermotogae* | 0.001315 | 0 | 0.004856 | 0.036368 | Bacteria |
| 20 | *Mucoromycota* | 0.001315 | 0.00322 | 0.014569 | 0.018184 | Eukaryota |
| 21 | *Lentisphaerae* | 0 | 0.00644 | 0.009713 | 0.018184 | Bacteria |
| 22 | *Synergistetes* | 0 | 0 | 0.009713 | 0.024245 | Bacteria |
| 23 | *Ignavibacteriae* | 0 | 0.00161 | 0.014569 | 0.012123 | Bacteria |
| 24 | *Basidiomycota* | 0 | 0.00161 | 0 | 0.018184 | Eukaryota |
| 25 | *Thaumarchaeota* | 0 | 0 | 0 | 0.018184 | Archaea |
| 26 | *Euglenozoa* | 0 | 0 | 0.004856 | 0.012123 | Eukaryota |
| 27 | *Deferribacteres* | 0 | 0.00161 | 0 | 0.012123 | Bacteria |
| 28 | *Amoebozoa* | 0.001315 | 0 | 0 | 0.012123 | Eukaryota |
| 29 | *Elusimicrobia* | 0 | 0 | 0 | 0.012123 | Bacteria |
| 30 | *Bacillariophyta* | 0 | 0 | 0 | 0.012123 | Eukaryota |
| 31 | *Cryptophyta* | 0 | 0 | 0 | 0.012123 | Eukaryota |
| 32 | *Ciliophora* | 0 | 0.00161 | 0 | 0.006061 | Eukaryota |
| 33 | *Nitrospinae* | 0.001315 | 0 | 0 | 0.006061 | Bacteria |
| 34 | *Armatimonadetes* | 0 | 0 | 0 | 0.006061 | Bacteria |
| 35 | *Fibrobacteres* | 0 | 0 | 0 | 0.006061 | Bacteria |
| 36 | *Thermodesulfobacteria* | 0 | 0 | 0 | 0.006061 | Bacteria |
| 37 | *Crenarchaeota* | 0 | 0 | 0 | 0.006061 | Archaea |
| 38 | *Oomycota* | 0 | 0 | 0 | 0.006061 | Eukaryota |
| 39 | *Rhodophyta* | 0 | 0 | 0 | 0.006061 | Eukaryota |
| 40 | *Chrysiogenetes* | 0 | 0 | 0.004856 | 0 | Bacteria |
| 41 | *Balneolaeota* | 0 | 0.00161 | 0 | 0 | Bacteria |
| 42 | *Chlorophyta* | 0 | 0.00161 | 0 | 0 | Eukaryota |
| 43 | *Dictyoglomi* | 0.001315 | 0 | 0 | 0 | Bacteria |
| 44 | unclassified eukaryotic phyla | 0 | 0 | 0 | 0.018184 |  |
| 45 | unclassified archaeal phyla phyla | 0 | 0.00483 | 0 | 0.018184 |  |
| 46 | unclassified bacterial phyla | 0.085487 | 0.243109 | 0.403069 | 0.49703 |  |
