# Supplementary material for: Shotgun sequence-based metataxonomic and predictive functional profiles of Pe poke, a naturally fermented soybean food of Myanmar
Source: PLoS One. 2021 Dec 17;16(12):e0260777. doi: 10.1371/journal.pone.0260777 (PMC8682898; doi:10.1371/journal.pone.0260777)
Supplement: S2 Table — (DOCX) [file pone.0260777.s002.docx]

**Supplementary Table 2.** The minor families with a relative abundance of <1% detected in *pe poke*.

| Sl. No. | Family | Relative abundance (%) | | | | Domain |
| --- | --- | --- | --- | --- | --- | --- |
|  |  | 3ds | 4ds | 5ds | Sds |  |
| 1 | *Burkholderiaceae* | 0.009938 | 0.963109 | 1.274805 | 1.373015 | Bacteria |
| 2 | *Xanthomonadaceae* | 0.014907 | 0.210858 | 3.006615 | 0.383524 | Bacteria |
| 3 | *Paenibacillaceae* | 0.490269 | 0.349531 | 0.691521 | 1.902278 | Bacteria |
| 4 | *Comamonadaceae* | 0.004969 | 0.65537 | 1.316897 | 1.020173 | Bacteria |
| 5 | *Enterobacteriaceae* | 0.011594 | 0.290642 | 1.383043 | 1.173583 | Bacteria |
| 6 | *Moraxellaceae* | 0.023188 | 0.343832 | 1.202646 | 1.073867 | Bacteria |
| 7 | *Neisseriaceae* | 0.034783 | 0.243152 | 0.76368 | 0.95881 | Bacteria |
| 8 | *Carnobacteriaceae* | 0.750311 | 0.125375 | 0.108238 | 0.889775 | Bacteria |
| 9 | *Clostridiaceae* | 0.357764 | 0.167167 | 0.463019 | 0.767048 | Bacteria |
| 10 | *Staphylococcaceae* | 0.291511 | 0.216557 | 0.288635 | 0.81307 | Bacteria |
| 11 | *Myoviridae* | 0.978882 | 0.471107 | 0.01804 | 0.130398 | Viruses |
| 12 | *Alteromonadaceae* | 0.004969 | 0.241252 | 0.667468 | 0.521592 | Bacteria |
| 13 | *Oxalobacteraceae* | 0.004969 | 0.383724 | 0.529164 | 0.49091 | Bacteria |
| 14 | *Vibrionaceae* | 0.018219 | 0.191862 | 0.318701 | 0.828411 | Bacteria |
| 15 | *Siphoviridae* | 0.841408 | 0.163368 | 0.024053 | 0.32216 | Viruses |
| 16 | *Oceanospirillaceae* | 0.013251 | 0.233654 | 0.619363 | 0.460229 | Bacteria |
| 17 | *Pasteurellaceae* | 0.011594 | 0.121576 | 0.589296 | 0.521592 | Bacteria |
| 18 | *Halomonadaceae* | 0.008282 | 0.235553 | 0.463019 | 0.47557 | Bacteria |
| 19 | *Rhodobacteraceae* | 0.011594 | 0.30394 | 0.457005 | 0.406535 | Bacteria |
| 20 | *Streptococcaceae* | 0.425673 | 0.117777 | 0.090198 | 0.345171 | Bacteria |
| 21 | *Lactobacillaceae* | 0.286542 | 0.10258 | 0.102225 | 0.444888 | Bacteria |
| 22 | *Aerococcaceae* | 0.051346 | 0.018996 | 0.487072 | 0.283808 | Bacteria |
| 23 | *Listeriaceae* | 0.304762 | 0.15387 | 0.096212 | 0.276137 | Bacteria |
| 24 | *Chromobacteriaceae* | 0.006625 | 0.142472 | 0.37282 | 0.291478 | Bacteria |
| 25 | *Brucellaceae* | 0 | 0.011398 | 0.55923 | 0.130398 | Bacteria |
| 26 | *Rhodocyclaceae* | 0 | 0.159568 | 0.156344 | 0.375853 | Bacteria |
| 27 | *Phyllobacteriaceae* | 0 | 0.060788 | 0.396873 | 0.207103 | Bacteria |
| 28 | *Thermoactinomycetaceae* | 0.109317 | 0.081684 | 0.258569 | 0.199432 | Bacteria |
| 29 | *Lachnospiraceae* | 0.187164 | 0.081684 | 0.102225 | 0.276137 | Bacteria |
| 30 | *Sporolactobacillaceae* | 0.096066 | 0.041792 | 0.096212 | 0.398865 | Bacteria |
| 31 | *Orbaceae* | 0.009938 | 0.045591 | 0.066146 | 0.506251 | Bacteria |
| 32 | *Yersiniaceae* | 0.014907 | 0.070286 | 0.16837 | 0.268467 | Bacteria |
| 33 | *Rhizobiaceae* | 0.001656 | 0.094981 | 0.192423 | 0.214773 | Bacteria |
| 34 | *Ectothiorhodospiraceae* | 0 | 0.075985 | 0.18641 | 0.214773 | Bacteria |
| 35 | *Sphingomonadaceae* | 0.003313 | 0.075985 | 0.138304 | 0.214773 | Bacteria |
| 36 | *Erwiniaceae* | 0.006625 | 0.070286 | 0.072159 | 0.268467 | Bacteria |
| 37 | *Acetobacteraceae* | 0.001656 | 0.068386 | 0.120265 | 0.214773 | Bacteria |
| 38 | *Bradyrhizobiaceae* | 0 | 0.070286 | 0.198437 | 0.130398 | Bacteria |
| 39 | *Pectobacteriaceae* | 0.013251 | 0.075985 | 0.198437 | 0.092046 | Bacteria |
| 40 | *Piscirickettsiaceae* | 0.003313 | 0.077885 | 0.120265 | 0.16108 | Bacteria |
| 41 | *Rhodospirillaceae* | 0.004969 | 0.070286 | 0.108238 | 0.16875 | Bacteria |
| 42 | *Erysipelotrichaceae* | 0.048033 | 0.011398 | 0.024053 | 0.268467 | Bacteria |
| 43 | *Chitinophagaceae* | 0 | 0.003799 | 0.282622 | 0.061364 | Bacteria |
| 44 | *Streptomycetaceae* | 0.024845 | 0.055089 | 0.126278 | 0.130398 | Bacteria |
| 45 | *Chromatiaceae* | 0.003313 | 0.066487 | 0.102225 | 0.15341 | Bacteria |
| 46 | *Peptococcaceae* | 0.041408 | 0.04939 | 0.078172 | 0.15341 | Bacteria |
| 47 | *Methylococcaceae* | 0.001656 | 0.041792 | 0.120265 | 0.15341 | Bacteria |
| 48 | *Porphyromonadaceae* | 0 | 0.030394 | 0.222489 | 0.061364 | Bacteria |
| 49 | *Peptostreptococcaceae* | 0.026501 | 0.005699 | 0.174384 | 0.099716 | Bacteria |
| 50 | *Nitrosomonadaceae* | 0 | 0.094981 | 0.084185 | 0.122728 | Bacteria |
| 51 | *Ruminococcaceae* | 0.079503 | 0.036093 | 0.042093 | 0.138069 | Bacteria |
| 52 | *Aeromonadaceae* | 0.004969 | 0.056989 | 0.114251 | 0.107387 | Bacteria |
| 53 | *Cytophagaceae* | 0.003313 | 0.0019 | 0.210463 | 0.061364 | Bacteria |
| 54 | *Micrococcaceae* | 0.006625 | 0.018996 | 0.024053 | 0.222444 | Bacteria |
| 55 | *Sutterellaceae* | 0 | 0.043691 | 0.066146 | 0.15341 | Bacteria |
| 56 | *Thiotrichaceae* | 0.001656 | 0.060788 | 0.054119 | 0.145739 | Bacteria |
| 57 | *Bacteroidaceae* | 0.046377 | 0.017097 | 0.066146 | 0.122728 | Bacteria |
| 58 | *Alicyclobacillaceae* | 0 | 0.017097 | 0.102225 | 0.122728 | Bacteria |
| 59 | *Mycobacteriaceae* | 0.049689 | 0.05129 | 0.048106 | 0.084375 | Bacteria |
| 60 | *Shewanellaceae* | 0.006625 | 0.017097 | 0.090198 | 0.115057 | Bacteria |
| 61 | *Legionellaceae* | 0.001656 | 0.068386 | 0.030066 | 0.115057 | Bacteria |
| 62 | *Rhodanobacteraceae* | 0.001656 | 0.055089 | 0.066146 | 0.092046 | Bacteria |
| 63 | *Idiomarinaceae* | 0 | 0.036093 | 0.114251 | 0.053693 | Bacteria |
| 64 | *Methylophilaceae* | 0 | 0.05129 | 0.030066 | 0.115057 | Bacteria |
| 65 | *Heliobacteriaceae* | 0.008282 | 0.037992 | 0.024053 | 0.099716 | Bacteria |
| 66 | *Cellvibrionaceae* | 0 | 0.030394 | 0.042093 | 0.092046 | Bacteria |
| 67 | *Alcanivoracaceae* | 0.001656 | 0.024695 | 0.090198 | 0.046023 | Bacteria |
| 68 | *Podoviridae* | 0.066253 | 0.0019 | 0.024053 | 0.061364 | Viruses |
| 69 | *Corynebacteriaceae* | 0.004969 | 0.007598 | 0.024053 | 0.115057 | Bacteria |
| 70 | *Hyphomicrobiaceae* | 0.004969 | 0.036093 | 0.024053 | 0.084375 | Bacteria |
| 71 | *Methylobacteriaceae* | 0.004969 | 0.022795 | 0.072159 | 0.046023 | Bacteria |
| 72 | *Bartonellaceae* | 0 | 0.003799 | 0.084185 | 0.053693 | Bacteria |
| 73 | *Campylobacteraceae* | 0.013251 | 0.009498 | 0.01804 | 0.099716 | Bacteria |
| 74 | *Leuconostocaceae* | 0.056315 | 0.013297 | 0.024053 | 0.046023 | Bacteria |
| 75 | *Peptoniphilaceae* | 0.048033 | 0.007598 | 0.006013 | 0.076705 | Bacteria |
| 76 | *Cyclobacteriaceae* | 0.001656 | 0.0019 | 0.126278 | 0.00767 | Bacteria |
| 77 | *Caulobacteraceae* | 0.004969 | 0.026595 | 0.036079 | 0.061364 | Bacteria |
| 78 | *Veillonellaceae* | 0 | 0.032294 | 0.01804 | 0.076705 | Bacteria |
| 79 | *Pseudonocardiaceae* | 0.001656 | 0.013297 | 0.012026 | 0.099716 | Bacteria |
| 80 | *Desulfovibrionaceae* | 0 | 0.007598 | 0.024053 | 0.092046 | Bacteria |
| 81 | *Hymenobacteraceae* | 0 | 0 | 0.108238 | 0.015341 | Bacteria |
| 82 | *Eubacteriaceae* | 0.043064 | 0.011398 | 0 | 0.069034 | Bacteria |
| 83 | *Hydrogenophilaceae* | 0 | 0.04939 | 0.01804 | 0.053693 | Bacteria |
| 84 | *Sinobacteraceae* | 0 | 0.028494 | 0.006013 | 0.084375 | Bacteria |
| 85 | *Crocinitomicaceae* | 0 | 0.013297 | 0.096212 | 0.00767 | Bacteria |
| 86 | *Microbacteriaceae* | 0.009938 | 0.022795 | 0.012026 | 0.069034 | Bacteria |
| 87 | *Pseudoalteromonadaceae* | 0.004969 | 0.020896 | 0.006013 | 0.076705 | Bacteria |
| 88 | *Sporomusaceae* | 0.026501 | 0.007598 | 0.012026 | 0.061364 | Bacteria |
| 89 | *Aurantimonadaceae* | 0 | 0.037992 | 0.054119 | 0.015341 | Bacteria |
| 90 | *Cardiobacteriaceae* | 0 | 0.015197 | 0 | 0.092046 | Bacteria |
| 91 | *Spirochaetaceae* | 0.016563 | 0.011398 | 0.012026 | 0.061364 | Bacteria |
| 92 | *Prevotellaceae* | 0.006625 | 0.017097 | 0.030066 | 0.046023 | Bacteria |
| 93 | *Thermoanaerobacteraceae* | 0.006625 | 0.009498 | 0.012026 | 0.069034 | Bacteria |
| 94 | *Nocardiaceae* | 0 | 0.011398 | 0.030066 | 0.053693 | Bacteria |
| 95 | *Halieaceae* | 0.001656 | 0.007598 | 0.024053 | 0.061364 | Bacteria |
| 96 | *Colwelliaceae* | 0.001656 | 0.005699 | 0.01804 | 0.069034 | Bacteria |
| 97 | *Microbulbiferaceae* | 0.001656 | 0.013297 | 0.01804 | 0.061364 | Bacteria |
| 98 | *Coxiellaceae* | 0 | 0.007598 | 0.012026 | 0.069034 | Bacteria |
| 99 | *Actinomycetaceae* | 0.003313 | 0.013297 | 0.01804 | 0.053693 | Bacteria |
| 100 | *Psychromonadaceae* | 0.001656 | 0 | 0.036079 | 0.046023 | Bacteria |
| 101 | *Flammeovirgaceae* | 0 | 0.007598 | 0.066146 | 0.00767 | Bacteria |
| 102 | *Selenomonadaceae* | 0.001656 | 0.007598 | 0 | 0.069034 | Bacteria |
| 103 | *Succinivibrionaceae* | 0.006625 | 0.007598 | 0.006013 | 0.053693 | Bacteria |
| 104 | *Leptospiraceae* | 0.003313 | 0.007598 | 0.024053 | 0.038352 | Bacteria |
| 105 | *Deinococcaceae* | 0.003313 | 0.011398 | 0.012026 | 0.046023 | Bacteria |
| 106 | *Hahellaceae* | 0.003313 | 0.011398 | 0.012026 | 0.046023 | Bacteria |
| 107 | *Fusobacteriaceae* | 0.028157 | 0 | 0.006013 | 0.038352 | Bacteria |
| 108 | *Micromonosporaceae* | 0 | 0.013297 | 0.012026 | 0.046023 | Bacteria |
| 109 | *Chlorobiaceae* | 0 | 0.005699 | 0.042093 | 0.023011 | Bacteria |
| 110 | *Xanthobacteraceae* | 0 | 0.022795 | 0.024053 | 0.023011 | Bacteria |
| 111 | *Budviciaceae* | 0 | 0 | 0 | 0.069034 | Bacteria |
| 112 | *Gallionellaceae* | 0 | 0.022795 | 0 | 0.046023 | Bacteria |
| 113 | *Geobacteraceae* | 0 | 0.020896 | 0 | 0.046023 | Bacteria |
| 114 | *Prolixibacteraceae* | 0 | 0.005699 | 0.060132 | 0 | Bacteria |
| 115 | *Bifidobacteriaceae* | 0.023188 | 0.005699 | 0.012026 | 0.023011 | Bacteria |
| 116 | *Acidobacteriaceae* | 0 | 0.0019 | 0 | 0.061364 | Bacteria |
| 117 | *Nitrospiraceae* | 0.004969 | 0 | 0.012026 | 0.046023 | Bacteria |
| 118 | *Erythrobacteraceae* | 0 | 0.007598 | 0.006013 | 0.046023 | Bacteria |
| 119 | *Chlamydiaceae* | 0.003313 | 0.013297 | 0.012026 | 0.030682 | Bacteria |
| 120 | *Oscillatoriaceae* | 0 | 0.007598 | 0.012026 | 0.038352 | Bacteria |
| 121 | *Ferrimonadaceae* | 0 | 0.007598 | 0.01804 | 0.030682 | Bacteria |
| 122 | *Desulfobulbaceae* | 0 | 0.005699 | 0.012026 | 0.038352 | Bacteria |
| 123 | *Hafniaceae* | 0 | 0.003799 | 0.036079 | 0.015341 | Bacteria |
| 124 | *Desulfobacteraceae* | 0.004969 | 0.003799 | 0 | 0.046023 | Bacteria |
| 125 | *Marinilabiliaceae* | 0 | 0 | 0.024053 | 0.030682 | Bacteria |
| 126 | *Halanaerobiaceae* | 0.011594 | 0 | 0.012026 | 0.030682 | Bacteria |
| 127 | *Cryomorphaceae* | 0 | 0 | 0.030066 | 0.023011 | Bacteria |
| 128 | *Thermonemataceae* | 0 | 0 | 0.030066 | 0.023011 | Bacteria |
| 129 | *Helicobacteraceae* | 0 | 0.005699 | 0.006013 | 0.038352 | Bacteria |
| 130 | *Francisellaceae* | 0 | 0.011398 | 0 | 0.038352 | Bacteria |
| 131 | *Salinisphaeraceae* | 0 | 0.011398 | 0 | 0.038352 | Bacteria |
| 132 | *Planctomycetaceae* | 0 | 0.009498 | 0.024053 | 0.015341 | Bacteria |
| 133 | *Spongiibacteraceae* | 0 | 0.011398 | 0.006013 | 0.030682 | Bacteria |
| 134 | *Desulfurobacteriaceae* | 0 | 0 | 0.024053 | 0.023011 | Bacteria |
| 135 | *Hyphomonadaceae* | 0 | 0.003799 | 0.012026 | 0.030682 | Bacteria |
| 136 | *Competibacteraceae* | 0 | 0.0019 | 0.012026 | 0.030682 | Bacteria |
| 137 | *Saccharospirillaceae* | 0 | 0.007598 | 0.006013 | 0.030682 | Bacteria |
| 138 | *Ferrovaceae* | 0 | 0.013297 | 0 | 0.030682 | Bacteria |
| 139 | *Thermaceae* | 0 | 0.003799 | 0.024053 | 0.015341 | Bacteria |
| 140 | *Thorselliaceae* | 0 | 0.015197 | 0.012026 | 0.015341 | Bacteria |
| 141 | *Frankiaceae* | 0 | 0.013297 | 0.006013 | 0.023011 | Bacteria |
| 142 | *Acholeplasmataceae* | 0 | 0 | 0.01804 | 0.023011 | Bacteria |
| 143 | *Halothiobacillaceae* | 0 | 0.005699 | 0.012026 | 0.023011 | Bacteria |
| 144 | *Methanobacteriaceae* | 0.009938 | 0 | 0.006013 | 0.023011 | Archaea |
| 145 | *Desulfovibrionaceae* | 0.008282 | 0.030394 | 0 | 0 | Bacteria |
| 146 | *Brevibacteriaceae* | 0 | 0 | 0 | 0.038352 | Bacteria |
| 147 | *Intrasporangiaceae* | 0 | 0 | 0.006013 | 0.030682 | Bacteria |
| 148 | *Rhodobiaceae* | 0 | 0.005699 | 0 | 0.030682 | Bacteria |
| 149 | *Desulfuromonadaceae* | 0 | 0.013297 | 0 | 0.023011 | Bacteria |
| 150 | *Synergistaceae* | 0 | 0 | 0.012026 | 0.023011 | Bacteria |
| 151 | *Pseudanabaenaceae* | 0 | 0.003799 | 0 | 0.030682 | Bacteria |
| 152 | *Acidiferrobacteraceae* | 0 | 0.011398 | 0 | 0.023011 | Bacteria |
| 153 | *Nocardioidaceae* | 0 | 0.018996 | 0 | 0.015341 | Bacteria |
| 154 | *Halobacteroidaceae* | 0.003313 | 0.0019 | 0.006013 | 0.023011 | Bacteria |
| 155 | *Kangiellaceae* | 0 | 0 | 0.01804 | 0.015341 | Bacteria |
| 156 | *Budviciaceae* | 0.003313 | 0 | 0.030066 | 0 | Bacteria |
| 157 | *Porticoccaceae* | 0 | 0.007598 | 0.01804 | 0.00767 | Bacteria |
| 158 | *Leptotrichiaceae* | 0.008282 | 0.0019 | 0 | 0.023011 | Bacteria |
| 159 | *Beijerinckiaceae* | 0 | 0.011398 | 0.006013 | 0.015341 | Bacteria |
| 160 | *Acidaminococcaceae* | 0.001656 | 0.007598 | 0 | 0.023011 | Bacteria |
| 161 | *Atopobiaceae* | 0.008282 | 0 | 0 | 0.023011 | Bacteria |
| 162 | *Mucoraceae* | 0.001656 | 0.003799 | 0.01804 | 0.00767 | Eukaryota |
| 163 | *Aphanothecaceae* | 0 | 0.009498 | 0.006013 | 0.015341 | Bacteria |
| 164 | *Woeseiaceae* | 0 | 0.0019 | 0.012026 | 0.015341 | Bacteria |
| 165 | *Moritellaceae* | 0 | 0 | 0.006013 | 0.023011 | Bacteria |
| 166 | *Anaerolineaceae* | 0.001656 | 0 | 0.012026 | 0.015341 | Bacteria |
| 167 | *Sneathiellaceae* | 0 | 0 | 0.012026 | 0.015341 | Bacteria |
| 168 | *Synechococcaceae* | 0 | 0.005699 | 0.006013 | 0.015341 | Bacteria |
| 169 | *Tolypothrichaceae* | 0 | 0.0019 | 0 | 0.023011 | Bacteria |
| 170 | *Aspergillaceae* | 0.001656 | 0 | 0 | 0.023011 | Eukaryota |
| 171 | *Acidithiobacillaceae* | 0 | 0.003799 | 0.012026 | 0.00767 | Bacteria |
| 172 | *Rubritaleaceae* | 0 | 0.0019 | 0.006013 | 0.015341 | Bacteria |
| 173 | *Ventosimonadaceae* | 0 | 0.0019 | 0.006013 | 0.015341 | Bacteria |
| 174 | *Gemmatimonadaceae* | 0 | 0 | 0 | 0.023011 | Bacteria |
| 175 | *Rickettsiaceae* | 0 | 0 | 0 | 0.023011 | Bacteria |
| 176 | *Syntrophaceae* | 0 | 0.007598 | 0 | 0.015341 | Bacteria |
| 177 | *Archangiaceae* | 0 | 0 | 0.006013 | 0.015341 | Bacteria |
| 178 | *Trypanosomatidae* | 0 | 0 | 0.006013 | 0.015341 | Eukaryota |
| 179 | *Desulfarculaceae* | 0.009938 | 0.0019 | 0 | 0.00767 | Bacteria |
| 180 | *Natrialbaceae* | 0 | 0.003799 | 0 | 0.015341 | Archaea |
| 181 | *Desulfohalobiaceae* | 0 | 0.018996 | 0 | 0 | Bacteria |
| 182 | *Criblamydiaceae* | 0 | 0 | 0.01804 | 0 | Bacteria |
| 183 | *Deferribacteraceae* | 0 | 0.0019 | 0 | 0.015341 | Bacteria |
| 184 | *Spiroplasmataceae* | 0 | 0.0019 | 0 | 0.015341 | Bacteria |
| 185 | *Bryobacteraceae* | 0 | 0.0019 | 0.006013 | 0.00767 | Bacteria |
| 186 | *Kiloniellaceae* | 0 | 0.0019 | 0.006013 | 0.00767 | Bacteria |
| 187 | *Anaplasmataceae* | 0 | 0 | 0 | 0.015341 | Bacteria |
| 188 | *Bacteriovoracaceae* | 0 | 0 | 0 | 0.015341 | Bacteria |
| 189 | *Brachyspiraceae* | 0 | 0 | 0 | 0.015341 | Bacteria |
| 190 | *Cellulomonadaceae* | 0 | 0 | 0 | 0.015341 | Bacteria |
| 191 | *Oleiphilaceae* | 0 | 0 | 0 | 0.015341 | Bacteria |
| 192 | *Schleiferiaceae* | 0 | 0 | 0 | 0.015341 | Bacteria |
| 193 | *Geminigeraceae* | 0 | 0 | 0 | 0.015341 | Eukaryota |
| 194 | *Thalassiosiraceae* | 0 | 0 | 0 | 0.015341 | Eukaryota |
| 195 | *Petrotogaceae* | 0.001656 | 0 | 0.006013 | 0.00767 | Bacteria |
| 196 | *Nostocaceae* | 0 | 0.007598 | 0 | 0.00767 | Bacteria |
| 197 | *Microcoleaceae* | 0.004969 | 0.003799 | 0.006013 | 0 | Bacteria |
| 198 | *Coriobacteriaceae* | 0.006625 | 0.0019 | 0.006013 | 0 | Bacteria |
| 199 | *Immundisolibacteraceae* | 0 | 0.0019 | 0.012026 | 0 | Bacteria |
| 200 | *Propionibacteriaceae* | 0 | 0 | 0.006013 | 0.00767 | Bacteria |
| 201 | *Rikenellaceae* | 0.001656 | 0 | 0.012026 | 0 | Bacteria |
| 202 | *Nocardiopsaceae* | 0 | 0.005699 | 0 | 0.00767 | Bacteria |
| 203 | *Promicromonosporaceae* | 0 | 0.005699 | 0 | 0.00767 | Bacteria |
| 204 | *Opitutaceae* | 0.001656 | 0.003799 | 0 | 0.00767 | Bacteria |
| 205 | *Syntrophomonadaceae* | 0.003313 | 0.0019 | 0 | 0.00767 | Bacteria |
| 206 | *Defluviitaleaceae* | 0.004969 | 0 | 0 | 0.00767 | Bacteria |
| 207 | *Mycoplasmataceae* | 0.004969 | 0.007598 | 0 | 0 | Bacteria |
| 208 | *Lentimicrobiaceae* | 0 | 0 | 0.012026 | 0 | Bacteria |
| 209 | *Merismopediaceae* | 0 | 0 | 0.012026 | 0 | Bacteria |
| 210 | *Saprospiraceae* | 0 | 0 | 0.012026 | 0 | Bacteria |
| 211 | *Aquificaceae* | 0 | 0.003799 | 0 | 0.00767 | Bacteria |
| 212 | *Sulfuricellaceae* | 0 | 0.003799 | 0 | 0.00767 | Bacteria |
| 213 | *Eggerthellaceae* | 0.001656 | 0.0019 | 0 | 0.00767 | Bacteria |
| 214 | *Anaeromyxobacteraceae* | 0 | 0.0019 | 0 | 0.00767 | Bacteria |
| 215 | *Ardenticatenaceae* | 0 | 0.0019 | 0 | 0.00767 | Bacteria |
| 216 | *Dermabacteraceae* | 0 | 0.0019 | 0 | 0.00767 | Bacteria |
| 217 | *Desulfomicrobiaceae* | 0 | 0.0019 | 0 | 0.00767 | Bacteria |
| 218 | *Magnetococcaceae* | 0 | 0.0019 | 0 | 0.00767 | Bacteria |
| 219 | *Methylothermaceae* | 0 | 0.0019 | 0 | 0.00767 | Bacteria |
| 220 | *Haloarculaceae* | 0 | 0.0019 | 0 | 0.00767 | Archaea |
| 221 | *Gordoniaceae* | 0 | 0.009498 | 0 | 0 | Bacteria |
| 222 | *Thermithiobacillaceae* | 0 | 0.009498 | 0 | 0 | Bacteria |
| 223 | *Gonapodyaceae* | 0 | 0.009498 | 0 | 0 | Eukaryota |
| 224 | *Chroococcaceae* | 0.001656 | 0 | 0 | 0.00767 | Bacteria |
| 225 | *Glycomycetaceae* | 0.001656 | 0 | 0 | 0.00767 | Bacteria |
| 226 | *Nakamurellaceae* | 0.001656 | 0 | 0 | 0.00767 | Bacteria |
| 227 | *Streptosporangiaceae* | 0.001656 | 0 | 0 | 0.00767 | Bacteria |
| 228 | *Acidimicrobiaceae* | 0 | 0 | 0 | 0.00767 | Bacteria |
| 229 | *Actinopolysporaceae* | 0 | 0 | 0 | 0.00767 | Bacteria |
| 230 | *Bdellovibrionaceae* | 0 | 0 | 0 | 0.00767 | Bacteria |
| 231 | *Beutenbergiaceae* | 0 | 0 | 0 | 0.00767 | Bacteria |
| 232 | *Blattabacteriaceae* | 0 | 0 | 0 | 0.00767 | Bacteria |
| 233 | *Caldicoprobacteraceae* | 0 | 0 | 0 | 0.00767 | Bacteria |
| 234 | *Catabacteriaceae* | 0 | 0 | 0 | 0.00767 | Bacteria |
| 235 | *Chamaesiphonaceae* | 0 | 0 | 0 | 0.00767 | Bacteria |
| 236 | *Chitinispirillaceae* | 0 | 0 | 0 | 0.00767 | Bacteria |
| 237 | *Chloroflexaceae* | 0 | 0 | 0 | 0.00767 | Bacteria |
| 238 | *Conexibacteraceae* | 0 | 0 | 0 | 0.00767 | Bacteria |
| 239 | *Demequinaceae* | 0 | 0 | 0 | 0.00767 | Bacteria |
| 240 | *Dermacoccaceae* | 0 | 0 | 0 | 0.00767 | Bacteria |
| 241 | *Desulfurellaceae* | 0 | 0 | 0 | 0.00767 | Bacteria |
| 242 | *Dietziaceae* | 0 | 0 | 0 | 0.00767 | Bacteria |
| 243 | *Fervidobacteriaceae* | 0 | 0 | 0 | 0.00767 | Bacteria |
| 244 | *Herpetosiphonaceae* | 0 | 0 | 0 | 0.00767 | Bacteria |
| 245 | *Isosphaeraceae* | 0 | 0 | 0 | 0.00767 | Bacteria |
| 246 | *Kosmotogaceae* | 0 | 0 | 0 | 0.00767 | Bacteria |
| 247 | *Leptolyngbyaceae* | 0 | 0 | 0 | 0.00767 | Bacteria |
| 248 | *Methylocystaceae* | 0 | 0 | 0 | 0.00767 | Bacteria |
| 249 | *Microthrixaceae* | 0 | 0 | 0 | 0.00767 | Bacteria |
| 250 | *Oscillospiraceae* | 0 | 0 | 0 | 0.00767 | Bacteria |
| 251 | *Prochloraceae* | 0 | 0 | 0 | 0.00767 | Bacteria |
| 252 | *Sphaerobacteraceae* | 0 | 0 | 0 | 0.00767 | Bacteria |
| 253 | *Symbiobacteriaceae* | 0 | 0 | 0 | 0.00767 | Bacteria |
| 254 | *Thermodesulfobacteriaceae* | 0 | 0 | 0 | 0.00767 | Bacteria |
| 255 | *Trueperaceae* | 0 | 0 | 0 | 0.00767 | Bacteria |
| 256 | *Desulfurococcaceae* | 0 | 0 | 0 | 0.00767 | Archaea |
| 257 | *Halobacteriaceae* | 0 | 0 | 0 | 0.00767 | Archaea |
| 258 | *Bathycoccaceae* | 0 | 0 | 0 | 0.00767 | Eukaryota |
| 259 | *Choanephoraceae* | 0 | 0 | 0 | 0.00767 | Eukaryota |
| 260 | *Clavicipitaceae* | 0 | 0 | 0 | 0.00767 | Eukaryota |
| 261 | *Delesseriaceae* | 0 | 0 | 0 | 0.00767 | Eukaryota |
| 262 | *Glomeraceae* | 0 | 0 | 0 | 0.00767 | Eukaryota |
| 263 | *Malasseziaceae* | 0 | 0 | 0 | 0.00767 | Eukaryota |
| 264 | *Mixiaceae* | 0 | 0 | 0 | 0.00767 | Eukaryota |
| 265 | *Mycosphaerellaceae* | 0 | 0 | 0 | 0.00767 | Eukaryota |
| 266 | *Perkinsidae* | 0 | 0 | 0 | 0.00767 | Eukaryota |
| 267 | *Pseudeurotiaceae* | 0 | 0 | 0 | 0.00767 | Eukaryota |
| 268 | *Pucciniaceae* | 0 | 0 | 0 | 0.00767 | Eukaryota |
| 269 | *Saccharomycetaceae* | 0 | 0 | 0 | 0.00767 | Eukaryota |
| 270 | *Tetrahymenidae* | 0 | 0 | 0 | 0.00767 | Eukaryota |
| 271 | *Unikaryonidae* | 0 | 0 | 0 | 0.00767 | Eukaryota |
| 272 | *Caldicoprobacteraceae* | 0.001656 | 0 | 0.006013 | 0 | Bacteria |
| 273 | *Syntrophorhabdaceae* | 0 | 0.007598 | 0 | 0 | Bacteria |
| 274 | *Actinospicaceae* | 0 | 0 | 0.006013 | 0 | Bacteria |
| 275 | *Akkermansiaceae* | 0 | 0 | 0.006013 | 0 | Bacteria |
| 276 | *Algiphilaceae* | 0 | 0 | 0.006013 | 0 | Bacteria |
| 277 | *Bernardetiaceae* | 0 | 0 | 0.006013 | 0 | Bacteria |
| 278 | *Chrysiogenaceae* | 0 | 0 | 0.006013 | 0 | Bacteria |
| 279 | *Kordiimonadaceae* | 0 | 0 | 0.006013 | 0 | Bacteria |
| 280 | *Lentisphaeraceae* | 0 | 0 | 0.006013 | 0 | Bacteria |
| 281 | *Mariprofundaceae* | 0 | 0 | 0.006013 | 0 | Bacteria |
| 282 | *Microscillaceae* | 0 | 0 | 0.006013 | 0 | Bacteria |
| 283 | *Odoribacteraceae* | 0 | 0 | 0.006013 | 0 | Bacteria |
| 284 | *Rickettsiaceae* | 0 | 0 | 0.006013 | 0 | Bacteria |
| 285 | *Rivulariaceae* | 0 | 0 | 0.006013 | 0 | Bacteria |
| 286 | *Archaeoglobaceae* | 0 | 0 | 0.006013 | 0 | Archaea |
| 287 | *Methanocaldococcaceae* | 0 | 0 | 0.006013 | 0 | Archaea |
| 288 | *Verrucariaceae* | 0 | 0 | 0.006013 | 0 | Eukaryota |
| 289 | *Rubrobacteraceae* | 0.001656 | 0.003799 | 0 | 0 | Bacteria |
| 290 | *Dermocarpellaceae* | 0 | 0.003799 | 0 | 0 | Bacteria |
| 291 | *Geodermatophilaceae* | 0 | 0.003799 | 0 | 0 | Bacteria |
| 292 | *Parachlamydiaceae* | 0 | 0.003799 | 0 | 0 | Bacteria |
| 293 | *Pelagibacteraceae* | 0 | 0.003799 | 0 | 0 | Bacteria |
| 294 | *Coniochaetaceae* | 0 | 0.003799 | 0 | 0 | Eukaryota |
| 295 | *Halococcaceae* | 0.001656 | 0.0019 | 0 | 0 | Archaea |
| 296 | *Balneolaceae* | 0 | 0.0019 | 0 | 0 | Bacteria |
| 297 | *Cohaesibacteraceae* | 0 | 0.0019 | 0 | 0 | Bacteria |
| 298 | *Haliscomenobacteraceae* | 0 | 0.0019 | 0 | 0 | Bacteria |
| 299 | *Holophagaceae* | 0 | 0.0019 | 0 | 0 | Bacteria |
| 300 | *Kofleriaceae* | 0 | 0.0019 | 0 | 0 | Bacteria |
| 301 | *Ktedonobacteraceae* | 0 | 0.0019 | 0 | 0 | Bacteria |
| 302 | *Labilitrichaceae* | 0 | 0.0019 | 0 | 0 | Bacteria |
| 303 | *Melioribacteraceae* | 0 | 0.0019 | 0 | 0 | Bacteria |
| 304 | *Microcystaceae* | 0 | 0.0019 | 0 | 0 | Bacteria |
| 305 | *Polyangiaceae* | 0 | 0.0019 | 0 | 0 | Bacteria |
| 306 | *Sporichthyaceae* | 0 | 0.0019 | 0 | 0 | Bacteria |
| 307 | *Vulgatibacteraceae* | 0 | 0.0019 | 0 | 0 | Bacteria |
| 308 | *Haloferacaceae* | 0 | 0.0019 | 0 | 0 | Archaea |
| 309 | *Halorubraceae* | 0 | 0.0019 | 0 | 0 | Archaea |
| 310 | *Methanoregulaceae* | 0 | 0.0019 | 0 | 0 | Archaea |
| 311 | *Coccomyxaceae* | 0 | 0.0019 | 0 | 0 | Eukaryota |
| 312 | *Pseudocohnilembidae* | 0 | 0.0019 | 0 | 0 | Eukaryota |
| 313 | *sordariomyceta* | 0 | 0.0019 | 0 | 0 | Eukaryota |
| 314 | *Trichocomaceae* | 0 | 0.0019 | 0 | 0 | Eukaryota |
| 315 | *Jonesiaceae* | 0.001656 | 0 | 0 | 0 | Bacteria |
| 316 | *Nitrospinaceae* | 0.001656 | 0 | 0 | 0 | Bacteria |
| 317 | *Thermomicrobiaceae* | 0.001656 | 0 | 0 | 0 | Bacteria |
| 318 | *Tissierellaceae* | 0.001656 | 0 | 0 | 0 | Bacteria |
| 319 | *Thermococcaceae* | 0.001656 | 0 | 0 | 0 | Archaea |
| 320 | *Acanthamoebidae* | 0.001656 | 0 | 0 | 0 | Eukaryota |
| 321 | unclassified bacterial family | 0.1706 | 0.778846 | 4.39567 | 2.017335 |  |
| 322 | unclassified archaeal family | 0 | 0.0019 | 0.012026 | 0.00767 |  |
| 323 | unclassified eukaryotic family | 0.004969 | 0.0019 | 0 | 0.15341 |  |
| 324 | unclassified viral families | 0.003313 | 0.003799 | 0.192423 | 0 |  |
