# Supplementary material for: Shotgun sequence-based metataxonomic and predictive functional profiles of Pe poke, a naturally fermented soybean food of Myanmar
Source: PLoS One. 2021 Dec 17;16(12):e0260777. doi: 10.1371/journal.pone.0260777 (PMC8682898; doi:10.1371/journal.pone.0260777)
Supplement: S3 Table — (DOCX) [file pone.0260777.s003.docx]

**Supplementary Table 3.** The minor genera with a relative abundance of <1% detected in *pe poke*.

| Sl. No. | Genera | Relative abundance (%) | | | | Domain |
| --- | --- | --- | --- | --- | --- | --- |
|  |  | 3ds | 4ds | 5ds | Sds |  |
| 1 | *Sporosarcina* | 0.07738 | 0.0666 | 2.44493 | 1.34102 | Bacteria |
| 2 | *Bordetella* | 0 | 1.034 | 1.65577 | 0.8845 | Bacteria |
| 3 | *Enterococcus* | 1.806 | 0.99019 | 0.1404 | 0.51929 | Bacteria |
| 4 | *Pseudomonas* | 0.01976 | 0.76761 | 1.39434 | 1.11847 | Bacteria |
| 5 | *Virgibacillus* | 0.17286 | 0.14196 | 0.84725 | 2.13422 | Bacteria |
| 6 | *Pusillimonas* | 0 | 0.89029 | 1.37013 | 0.75325 | Bacteria |
| 7 | *Vagococcus* | 1.51954 | 0.80442 | 0.00968 | 0.50217 | Bacteria |
| 8 | *Oligella* | 0 | 0.55731 | 1.27814 | 0.39375 | Bacteria |
| 9 | *Alcaligenes* | 0 | 0.55906 | 1.32656 | 0.32527 | Bacteria |
| 10 | *Lysinibacillus* | 0.11689 | 0.08412 | 0.5955 | 1.38096 | Bacteria |
| 11 | *Luteimonas* | 0 | 0.01577 | 2.0673 | 0.00571 | Bacteria |
| 12 | *Paenibacillus* | 0.36877 | 0.25061 | 0.45025 | 0.78178 | Bacteria |
| 13 | *Castellaniella* | 0 | 0.57834 | 0.6778 | 0.47364 | Bacteria |
| 14 | *Geobacillus* | 0.34572 | 0.30845 | 0.7359 | 0.25679 | Bacteria |
| 15 | *Parageobacillus* | 0.12183 | 0.19278 | 1.09901 | 0.16549 | Bacteria |
| 16 | *Acinetobacter* | 0.01976 | 0.19278 | 0.64875 | 0.54782 | Bacteria |
| 17 | *Achromobacter* | 0 | 0.47844 | 0.47446 | 0.32527 | Bacteria |
| 18 | *Candidimonas* | 0 | 0.39432 | 0.48899 | 0.3538 | Bacteria |
| 19 | *Nit1virus* | 0.67828 | 0.3768 | 0 | 0.09701 | Bacteria |
| 20 | *Kerstersia* | 0.00329 | 0.32422 | 0.56645 | 0.25108 | Viruses |
| 21 | *Clostridium* | 0.28481 | 0.14897 | 0.2566 | 0.43369 | Bacteria |
| 22 | *Oblitimonas* | 0 | 0.26989 | 0.70685 | 0.13125 | Bacteria |
| 23 | *Myroides* | 0 | 0.00701 | 1.02154 | 0.05706 | Bacteria |
| 24 | *Staphylococcus* | 0.27329 | 0.18577 | 0.17913 | 0.38233 | Bacteria |
| 25 | *Advenella* | 0 | 0.27865 | 0.40184 | 0.30244 | Bacteria |
| 26 | *Aeribacillus* | 0.03787 | 0.06484 | 0.794 | 0.07989 | Bacteria |
| 27 | *Ornithinibacillus* | 0.02799 | 0.01577 | 0.24691 | 0.68478 | Bacteria |
| 28 | *Burkholderia* | 0.00823 | 0.2401 | 0.35827 | 0.30815 | Bacteria |
| 29 | *Marinobacter* | 0.00329 | 0.16649 | 0.47446 | 0.2682 | Bacteria |
| 30 | *Ureibacillus* | 0.01152 | 0.08062 | 0.78431 | 0.02853 | Bacteria |
| 31 | *Escherichia* | 0.00165 | 0.11216 | 0.397 | 0.3538 | Bacteria |
| 32 | *Neisseria* | 0.03128 | 0.09639 | 0.33406 | 0.35951 | Bacteria |
| 33 | *Gracilibacillus* | 0.02634 | 0.04557 | 0.2566 | 0.48505 | Bacteria |
| 34 | *Chryseobacterium* | 0 | 0.01052 | 0.71169 | 0.06848 | Bacteria |
| 35 | *Lentibacillus* | 0.05433 | 0.02979 | 0.16461 | 0.5307 | Bacteria |
| 36 | *Streptococcus* | 0.39182 | 0.09639 | 0.0581 | 0.22826 | Bacteria |
| 37 | *Anoxybacillus* | 0.16792 | 0.10515 | 0.2566 | 0.22826 | Bacteria |
| 38 | *Lactobacillus* | 0.28481 | 0.09113 | 0.0823 | 0.28532 | Bacteria |
| 39 | *Carnobacterium* | 0.4445 | 0.06309 | 0.03873 | 0.1769 | Bacteria |
| 40 | *Halomonas* | 0.00494 | 0.20329 | 0.23723 | 0.2682 | Bacteria |
| 41 | *Listeria* | 0.23707 | 0.14196 | 0.07746 | 0.18261 | Bacteria |
| 42 | *Vibrio* | 0.00494 | 0.12443 | 0.1404 | 0.35951 | Bacteria |
| 43 | *Providencia* | 0.00494 | 0.05608 | 0.17429 | 0.35951 | Bacteria |
| 44 | *Globicatella* | 0.00659 | 0.00351 | 0.34858 | 0.18261 | Bacteria |
| 45 | *Aneurinibacillus* | 0.05268 | 0.01227 | 0.05326 | 0.42228 | Bacteria |
| 46 | *Psychrobacter* | 0.00329 | 0.08938 | 0.28565 | 0.13125 | Bacteria |
| 47 | *Paucisalibacillus* | 0.01976 | 0.00876 | 0.08715 | 0.3538 | Bacteria |
| 48 | *Pelistega* | 0 | 0.14371 | 0.23723 | 0.0856 | Bacteria |
| 49 | *Amphibacillus* | 0.02634 | 0.01928 | 0.13072 | 0.27391 | Bacteria |
| 50 | *Gilliamella* | 0.00988 | 0.03155 | 0.04841 | 0.34809 | Bacteria |
| 51 | *Pedobacter* | 0 | 0.03155 | 0.36311 | 0.03424 | Bacteria |
| 52 | *Cupriavidus* | 0.00165 | 0.1402 | 0.06778 | 0.14837 | Bacteria |
| 53 | *Orrella* | 0 | 0.12794 | 0.09683 | 0.12554 | Bacteria |
| 54 | *Ochrobactrum* | 0 | 0.00351 | 0.29533 | 0.05136 | Bacteria |
| 55 | *Herbaspirillum* | 0.00165 | 0.08412 | 0.10167 | 0.15978 | Bacteria |
| 56 | *Parapedobacter* | 0 | 0 | 0.32922 | 0 | Bacteria |
| 57 | *Pontibacillus* | 0.01152 | 0.05608 | 0.04841 | 0.21114 | Bacteria |
| 58 | *Comamonas* | 0 | 0.05433 | 0.17913 | 0.0913 | Bacteria |
| 59 | *Caryophanon* | 0.14652 | 0.09113 | 0.03873 | 0.04565 | Bacteria |
| 60 | *Halobacillus* | 0.01482 | 0.01402 | 0.01452 | 0.2625 | Bacteria |
| 61 | *Planococcus* | 0.00823 | 0.01577 | 0.05326 | 0.22826 | Bacteria |
| 62 | *Xanthomonas* | 0.00494 | 0.06484 | 0.09199 | 0.13125 | Bacteria |
| 63 | *Anaerobacillus* | 0.04939 | 0.06134 | 0.05326 | 0.12554 | Bacteria |
| 64 | *Brevibacillus* | 0.04939 | 0.02454 | 0.05326 | 0.15407 | Bacteria |
| 65 | *Paraburkholderia* | 0 | 0.09639 | 0.0581 | 0.11984 | Bacteria |
| 66 | *Acidovorax* | 0.00329 | 0.07536 | 0.12588 | 0.06277 | Bacteria |
| 67 | *Streptomyces* | 0.02469 | 0.04907 | 0.10167 | 0.0913 | Bacteria |
| 68 | *Vitreoscilla* | 0.00165 | 0.02979 | 0.12588 | 0.10842 | Bacteria |
| 69 | *Massilia* | 0.00165 | 0.0701 | 0.13072 | 0.06277 | Bacteria |
| 70 | *Novibacillus* | 0.02634 | 0.03856 | 0.16945 | 0.02853 | Bacteria |
| 71 | *Marinospirillum* | 0 | 0.05433 | 0.16945 | 0.03424 | Bacteria |
| 72 | *Curvibacter* | 0 | 0.05082 | 0.13556 | 0.06848 | Bacteria |
| 73 | *Variovorax* | 0 | 0.04031 | 0.15977 | 0.05136 | Bacteria |
| 74 | *Pandoraea* | 0 | 0.06835 | 0.11135 | 0.06848 | Bacteria |
| 75 | *Psychrobacillus* | 0.02469 | 0.00351 | 0.06778 | 0.14837 | Bacteria |
| 76 | *Paracoccus* | 0 | 0.0666 | 0.13556 | 0.03424 | Bacteria |
| 77 | *Empedobacter* | 0 | 0.00701 | 0.22271 | 0.00571 | Bacteria |
| 78 | *Marinobacterium* | 0.00165 | 0.06134 | 0.10167 | 0.06848 | Bacteria |
| 79 | *Vaginella* | 0 | 0 | 0.22271 | 0 | Bacteria |
| 80 | *Azotobacter* | 0 | 0.0333 | 0.09199 | 0.09701 | Bacteria |
| 81 | *Paraliobacillus* | 0.03293 | 0.00876 | 0.02905 | 0.14837 | Bacteria |
| 82 | *Ralstonia* | 0 | 0.05082 | 0.11619 | 0.05136 | Bacteria |
| 83 | *Domibacillus* | 0.03128 | 0.01052 | 0.10651 | 0.06277 | Bacteria |
| 84 | *Fictibacillus* | 0.03951 | 0.01402 | 0.04841 | 0.10842 | Bacteria |
| 85 | *Bacteroides* | 0.0461 | 0.01577 | 0.05326 | 0.0913 | Bacteria |
| 86 | *Paenisporosarcina* | 0.00329 | 0.01753 | 0.0823 | 0.10272 | Bacteria |
| 87 | *Brackiella* | 0 | 0.09639 | 0.03873 | 0.06848 | Bacteria |
| 88 | *Salmonella* | 0 | 0.03505 | 0.09199 | 0.07418 | Bacteria |
| 89 | *Atopostipes* | 0.00494 | 0 | 0 | 0.19402 | Bacteria |
| 90 | *Mycobacterium* | 0.04939 | 0.04732 | 0.03873 | 0.06277 | Bacteria |
| 91 | *Caldibacillus* | 0.05927 | 0.02629 | 0.02905 | 0.07989 | Bacteria |
| 92 | *Janthinobacterium* | 0 | 0.08587 | 0.08715 | 0.01712 | Bacteria |
| 93 | *Pasteurella* | 0.00329 | 0.01753 | 0.15008 | 0.01712 | Bacteria |
| 94 | *Bhargavaea* | 0.01482 | 0.00351 | 0.07262 | 0.09701 | Bacteria |
| 95 | *Thauera* | 0 | 0.04907 | 0.06778 | 0.06848 | Bacteria |
| 96 | *Gulbenkiania* | 0 | 0.01928 | 0.12588 | 0.03995 | Bacteria |
| 97 | *Kurthia* | 0.00329 | 0.01052 | 0.03873 | 0.13125 | Bacteria |
| 98 | *Salinicoccus* | 0.01152 | 0.00701 | 0.04841 | 0.11413 | Bacteria |
| 99 | *Photobacterium* | 0.00659 | 0.02629 | 0.03389 | 0.11413 | Bacteria |
| 100 | *Serratia* | 0.01152 | 0.01753 | 0.07746 | 0.07418 | Bacteria |
| 101 | *Shewanella* | 0.00659 | 0.01577 | 0.07262 | 0.0856 | Bacteria |
| 102 | *Bradyrhizobium* | 0 | 0.04031 | 0.09683 | 0.03995 | Bacteria |
| 103 | *Caldalkalibacillus* | 0.00659 | 0.00175 | 0.15008 | 0.01712 | Bacteria |
| 104 | *Taylorella* | 0 | 0.08062 | 0.02905 | 0.06277 | Bacteria |
| 105 | *Alicyclobacillus* | 0 | 0.01577 | 0.0823 | 0.07418 | Bacteria |
| 106 | *Weeksella* | 0 | 0 | 0.16461 | 0.00571 | Bacteria |
| 107 | *Leclercia* | 0 | 0.00526 | 0.16461 | 0 | Bacteria |
| 108 | *Haemophilus* | 0.00494 | 0.03155 | 0.0581 | 0.07418 | Bacteria |
| 109 | *Pseudopedobacter* | 0 | 0.00351 | 0.16461 | 0 | Bacteria |
| 110 | *Rhizobium* | 0 | 0.04031 | 0.05326 | 0.07418 | Bacteria |
| 111 | *Lampropedia* | 0 | 0.04031 | 0.08715 | 0.03995 | Bacteria |
| 112 | *Enterobacter* | 0 | 0.02278 | 0.09199 | 0.05136 | Bacteria |
| 113 | *Thalassobacillus* | 0.00494 | 0.00351 | 0.01452 | 0.14266 | Bacteria |
| 114 | *Pantoea* | 0.00659 | 0.02454 | 0.04841 | 0.0856 | Bacteria |
| 115 | *Stenotrophomonas* | 0 | 0.05608 | 0.04357 | 0.06277 | Bacteria |
| 116 | *Snodgrassella* | 0 | 0.03155 | 0.0581 | 0.06848 | Bacteria |
| 117 | *Capnocytophaga* | 0 | 0.00175 | 0.15008 | 0.00571 | Bacteria |
| 118 | *Photorhabdus* | 0 | 0.00526 | 0.11135 | 0.03995 | Bacteria |
| 119 | *Tepidimicrobium* | 0.00329 | 0 | 0.14524 | 0.00571 | Bacteria |
| 120 | *Moraxella* | 0 | 0.03155 | 0.02421 | 0.09701 | Bacteria |
| 121 | *Legionella* | 0.00165 | 0.06309 | 0.02421 | 0.06277 | Bacteria |
| 122 | *Salinibacillus* | 0.00165 | 0 | 0.01452 | 0.13125 | Bacteria |
| 123 | *Aeromonas* | 0.00494 | 0.02103 | 0.06294 | 0.05706 | Bacteria |
| 124 | *Thermoactinomyces* | 0.07244 | 0.02103 | 0.02905 | 0.02283 | Bacteria |
| 125 | *Pseudochrobactrum* | 0 | 0.00526 | 0.11135 | 0.02853 | Bacteria |
| 126 | *Idiomarina* | 0 | 0.02454 | 0.09199 | 0.02853 | Bacteria |
| 127 | *Klebsiella* | 0.00823 | 0.02103 | 0.0581 | 0.05706 | Bacteria |
| 128 | *Marinomonas* | 0 | 0.01928 | 0.07262 | 0.05136 | Bacteria |
| 129 | *Thiomonas* | 0 | 0.06134 | 0.04357 | 0.03424 | Bacteria |
| 130 | *Yersinia* | 0.00165 | 0.01928 | 0.01452 | 0.10272 | Bacteria |
| 131 | *Terribacillus* | 0.01317 | 0.02103 | 0.02905 | 0.07418 | Bacteria |
| 132 | *Jeotgalibacillus* | 0.00494 | 0.01052 | 0.02421 | 0.09701 | Bacteria |
| 133 | *Sporolactobacillus* | 0.03128 | 0.01052 | 0.01452 | 0.07989 | Bacteria |
| 134 | *Polynucleobacter* | 0 | 0.0333 | 0.06294 | 0.03995 | Bacteria |
| 135 | *Sediminibacillus* | 0.00165 | 0.01227 | 0.01937 | 0.10272 | Bacteria |
| 136 | *Heliobacterium* | 0.00659 | 0.03505 | 0.01937 | 0.07418 | Bacteria |
| 137 | *Xenorhabdus* | 0.00329 | 0.00175 | 0.06778 | 0.05706 | Bacteria |
| 138 | *Mesorhizobium* | 0 | 0.01052 | 0.06778 | 0.05136 | Bacteria |
| 139 | *Alcanivorax* | 0.00165 | 0.02103 | 0.07262 | 0.03424 | Bacteria |
| 140 | *Basilea* | 0 | 0.04031 | 0.04357 | 0.04565 | Bacteria |
| 141 | *Trichococcus* | 0.06585 | 0.01577 | 0.02421 | 0.02283 | Bacteria |
| 142 | *Lautropia* | 0 | 0.04381 | 0.03873 | 0.04565 | Bacteria |
| 143 | *Caballeronia* | 0 | 0.02103 | 0.07262 | 0.03424 | Bacteria |
| 144 | *Sutterella* | 0 | 0.02278 | 0.02421 | 0.07989 | Bacteria |
| 145 | *Nitrosomonas* | 0 | 0.04206 | 0.03873 | 0.04565 | Bacteria |
| 146 | *Salimicrobium* | 0.00329 | 0.00701 | 0.02421 | 0.0913 | Bacteria |
| 147 | *Pectobacterium* | 0 | 0.0368 | 0.07746 | 0.01141 | Bacteria |
| 148 | *Thiothrix* | 0 | 0.0368 | 0.01452 | 0.07418 | Bacteria |
| 149 | *Exiguobacterium* | 0.0461 | 0 | 0.00484 | 0.07418 | Bacteria |
| 150 | *Lysobacter* | 0.00494 | 0.01577 | 0.08715 | 0.01712 | Bacteria |
| 151 | *Viridibacillus* | 0.00494 | 0.00175 | 0.04841 | 0.06848 | Bacteria |
| 152 | *Shigella* | 0 | 0.00175 | 0.09683 | 0.02283 | Bacteria |
| 153 | *Alteribacillus* | 0 | 0.00175 | 0.04357 | 0.07418 | Bacteria |
| 154 | *Corynebacterium* | 0.00494 | 0.00701 | 0.01937 | 0.0856 | Bacteria |
| 155 | *Natribacillus* | 0 | 0.01402 | 0.06294 | 0.03995 | Bacteria |
| 156 | *Tepidibacillus* | 0.00329 | 0.00876 | 0.03389 | 0.06848 | Bacteria |
| 157 | *Gluconobacter* | 0.00165 | 0.01928 | 0.06294 | 0.02853 | Bacteria |
| 158 | *Bartonella* | 0 | 0.00351 | 0.06778 | 0.03995 | Bacteria |
| 159 | *Mannheimia* | 0 | 0.01577 | 0.04841 | 0.04565 | Bacteria |
| 160 | *Halolactibacillus* | 0.00823 | 0.00876 | 0.02421 | 0.06848 | Bacteria |
| 161 | *Rodentibacter* | 0 | 0.01753 | 0.02905 | 0.06277 | Bacteria |
| 162 | *Duganella* | 0 | 0.02629 | 0.0581 | 0.02283 | Bacteria |
| 163 | *Erwinia* | 0 | 0.02278 | 0.00968 | 0.07418 | Bacteria |
| 164 | *Alteromonas* | 0.00165 | 0.02103 | 0.04357 | 0.03995 | Bacteria |
| 165 | *Tuberibacillus* | 0.00494 | 0.00351 | 0.02905 | 0.06848 | Bacteria |
| 166 | *Polaromonas* | 0 | 0.04031 | 0.03389 | 0.02853 | Bacteria |
| 167 | *Pseudoxanthomonas* | 0.00165 | 0.01577 | 0.04841 | 0.03424 | Eukaryota |
| 168 | *Thiomicrospira* | 0 | 0.03155 | 0.03389 | 0.03424 | Bacteria |
| 169 | *Aquaspirillum* | 0 | 0.01052 | 0.07746 | 0.01141 | Bacteria |
| 170 | *Sphingomonas* | 0.00329 | 0.03505 | 0.01452 | 0.04565 | Bacteria |
| 171 | *Batrachochytrium* | 0.00494 | 0.00175 | 0 | 0.0913 | Bacteria |
| 172 | *Hydrogenophaga* | 0 | 0.02804 | 0.02905 | 0.03995 | Bacteria |
| 173 | *Methylibium* | 0.00165 | 0.00701 | 0.04841 | 0.03995 | Bacteria |
| 174 | *Erysipelothrix* | 0.01646 | 0 | 0 | 0.07989 | Bacteria |
| 175 | *Halotalea* | 0 | 0.00351 | 0.0581 | 0.03424 | Bacteria |
| 176 | *Eisenbergiella* | 0.0214 | 0.00175 | 0.07262 | 0 | Bacteria |
| 177 | *Alkalibacillus* | 0.00165 | 0.00175 | 0.04357 | 0.04565 | Bacteria |
| 178 | *Chitinophaga* | 0 | 0.00175 | 0.07262 | 0.01712 | Bacteria |
| 179 | *Paramesorhizobium* | 0 | 0 | 0.07746 | 0.01141 | Bacteria |
| 180 | *Edaphobacillus* | 0.01152 | 0.00701 | 0.02905 | 0.03995 | Bacteria |
| 181 | *Jeotgalibaca* | 0.04445 | 0.00351 | 0.00484 | 0.03424 | Bacteria |
| 182 | *Aquibacillus* | 0.02634 | 0.01052 | 0.01937 | 0.02853 | Bacteria |
| 183 | *Pseudoalteromonas* | 0.00329 | 0.01928 | 0.00484 | 0.05706 | Bacteria |
| 184 | *Arthrobacter* | 0.00659 | 0.01227 | 0.01937 | 0.04565 | Bacteria |
| 185 | *Chelonobacter* | 0 | 0 | 0.0823 | 0 | Bacteria |
| 186 | *Nitrosospira* | 0 | 0.01753 | 0.02905 | 0.03424 | Bacteria |
| 187 | *Cosenzaea* | 0 | 0 | 0.02905 | 0.05136 | Bacteria |
| 188 | *Fluviicola* | 0 | 0.01227 | 0.06778 | 0 | Bacteria |
| 189 | *Conchiformibius* | 0 | 0.01753 | 0.03389 | 0.02853 | Bacteria |
| 190 | *Chromobacterium* | 0 | 0.01577 | 0.02421 | 0.03995 | Bacteria |
| 191 | *Aquimarina* | 0 | 0.00175 | 0.07746 | 0 | Bacteria |
| 192 | *Methylophaga* | 0 | 0.01227 | 0.00968 | 0.05706 | Bacteria |
| 193 | *Desulfosporosinus* | 0.0214 | 0.00526 | 0.02905 | 0.02283 | Bacteria |
| 194 | *Geomicrobium* | 0.00165 | 0.00351 | 0.00484 | 0.06848 | Bacteria |
| 195 | *Piscibacillus* | 0 | 0.00701 | 0 | 0.06848 | Bacteria |
| 196 | *Gallibacterium* | 0.00329 | 0.00701 | 0.05326 | 0.01141 | Bacteria |
| 197 | *Desulfovibrio* | 0.00823 | 0.02278 | 0.00968 | 0.03424 | Bacteria |
| 198 | *Microbulbifer* | 0.00165 | 0.01227 | 0.01452 | 0.04565 | Bacteria |
| 199 | *Oceanospirillum* | 0.00494 | 0.00526 | 0.0581 | 0.00571 | Bacteria |
| 200 | *Delftia* | 0 | 0.02278 | 0.03389 | 0.01712 | Bacteria |
| 201 | *Marinilactibacillus* | 0.02305 | 0.01052 | 0 | 0.03995 | Bacteria |
| 202 | *Ruminiclostridium* | 0.01152 | 0.01402 | 0.01937 | 0.02853 | Bacteria |
| 203 | *Zymomonas* | 0 | 0.01052 | 0.03873 | 0.02283 | Bacteria |
| 204 | *Morganella* | 0.01646 | 0.01402 | 0.02421 | 0.01712 | Bacteria |
| 205 | *Ectothiorhodospira* | 0 | 0.01227 | 0.01937 | 0.03995 | Bacteria |
| 206 | *Campylobacter* | 0.00659 | 0.00876 | 0.00484 | 0.05136 | Bacteria |
| 207 | *Nitratireductor* | 0 | 0.00701 | 0.02905 | 0.03424 | Bacteria |
| 208 | *Aliivibrio* | 0.00165 | 0.00351 | 0.05326 | 0.01141 | Bacteria |
| 209 | *Noviherbaspirillum* | 0.00165 | 0.02454 | 0.01452 | 0.02853 | Bacteria |
| 210 | *Methylobacillus* | 0 | 0.01402 | 0.01452 | 0.03995 | Bacteria |
| 211 | *Brenneria* | 0 | 0.00351 | 0.0581 | 0.00571 | Bacteria |
| 212 | *Lactococcus* | 0.02799 | 0.01227 | 0.00968 | 0.01712 | Bacteria |
| 213 | *Isobaculum* | 0.0214 | 0 | 0 | 0.04565 | Bacteria |
| 214 | *Sphingobium* | 0.00329 | 0.00701 | 0.03389 | 0.02283 | Bacteria |
| 215 | *Oceanimonas* | 0 | 0.02629 | 0.02905 | 0.01141 | Bacteria |
| 216 | *Pelagirhabdus* | 0.00165 | 0 | 0.01937 | 0.04565 | Bacteria |
| 217 | *Algoriphagus* | 0.00165 | 0.00175 | 0.06294 | 0 | Bacteria |
| 218 | *Dickeya* | 0 | 0.00526 | 0.00968 | 0.05136 | Bacteria |
| 219 | *Parabacteroides* | 0 | 0.01577 | 0.03873 | 0.01141 | Bacteria |
| 220 | *Citrobacter* | 0 | 0.00876 | 0 | 0.05706 | Bacteria |
| 221 | *Psychromonas* | 0.00165 | 0 | 0.02905 | 0.03424 | Bacteria |
| 222 | *Brochothrix* | 0.04774 | 0 | 0 | 0.01712 | Bacteria |
| 223 | *Gramella* | 0 | 0 | 0.05326 | 0.01141 | Bacteria |
| 224 | *Mangrovimonas* | 0 | 0 | 0.05326 | 0.01141 | Bacteria |
| 225 | *Thioalkalivibrio* | 0 | 0.02103 | 0.04357 | 0 | Bacteria |
| 226 | *Cellulophaga* | 0 | 0.00526 | 0.05326 | 0.00571 | Bacteria |
| 227 | *Fusicatenibacter* | 0 | 0.01052 | 0.05326 | 0 | Bacteria |
| 228 | *Olivibacter* | 0 | 0 | 0.06294 | 0 | Bacteria |
| 229 | *Dysgonomonas* | 0 | 0.00876 | 0.04841 | 0.00571 | Bacteria |
| 230 | *Tenuibacillus* | 0.00659 | 0.00175 | 0.01452 | 0.03995 | Bacteria |
| 231 | *Methylomonas* | 0 | 0.00701 | 0.00968 | 0.04565 | Bacteria |
| 232 | *Lachnobacterium* | 0.02963 | 0.00351 | 0 | 0.02853 | Bacteria |
| 233 | *Prevotella* | 0.00659 | 0.01227 | 0.01937 | 0.02283 | Bacteria |
| 234 | *Thiobacillus* | 0 | 0.02278 | 0.00968 | 0.02853 | Bacteria |
| 235 | *Polaribacter* | 0 | 0 | 0.04357 | 0.01712 | Bacteria |
| 236 | *Paucibacter* | 0 | 0.00701 | 0.01937 | 0.03424 | Bacteria |
| 237 | *Rhodoferax* | 0 | 0.01577 | 0.00484 | 0.03995 | Bacteria |
| 238 | *Solibacillus* | 0.00329 | 0.00175 | 0.00968 | 0.04565 | Bacteria |
| 239 | *Alicycliphilus* | 0 | 0.01577 | 0.03873 | 0.00571 | Bacteria |
| 240 | *Desulfotomaculum* | 0.00823 | 0.01052 | 0.02421 | 0.01712 | Bacteria |
| 241 | *Muricauda* | 0 | 0 | 0.04841 | 0.01141 | Bacteria |
| 242 | *Blautia* | 0.02799 | 0.00876 | 0 | 0.02283 | Bacteria |
| 243 | *Acetobacter* | 0 | 0.00876 | 0.00484 | 0.04565 | Bacteria |
| 244 | *Eubacterium* | 0.03457 | 0.00175 | 0 | 0.02283 | Bacteria |
| 245 | *Niabella* | 0 | 0 | 0.05326 | 0.00571 | Bacteria |
| 246 | *Nonlabens* | 0 | 0 | 0.05326 | 0.00571 | Bacteria |
| 247 | *Novosphingobium* | 0 | 0.01577 | 0.01452 | 0.02853 | Bacteria |
| 248 | *Azospirillum* | 0 | 0.01227 | 0.02905 | 0.01712 | Bacteria |
| 249 | *Pilibacter* | 0.04774 | 0 | 0.00484 | 0.00571 | Bacteria |
| 250 | *Methylobacterium* | 0.00494 | 0.00526 | 0.01937 | 0.02853 | Bacteria |
| 251 | *Hoeflea* | 0 | 0.01402 | 0.00968 | 0.03424 | Bacteria |
| 252 | *Deinococcus* | 0.00329 | 0.01052 | 0.00968 | 0.03424 | Bacteria |
| 253 | *Collimonas* | 0 | 0.01928 | 0.01452 | 0.02283 | Bacteria |
| 254 | *Eikenella* | 0 | 0.00175 | 0.01452 | 0.03995 | Bacteria |
| 255 | *Rheinheimera* | 0 | 0.01227 | 0.00968 | 0.03424 | Bacteria |
| 256 | *Mucilaginibacter* | 0 | 0.00175 | 0.04841 | 0.00571 | Bacteria |
| 257 | *Rhodanobacter* | 0 | 0.01052 | 0.03389 | 0.01141 | Bacteria |
| 258 | *Parasutterella* | 0 | 0.01402 | 0.02421 | 0.01712 | Bacteria |
| 259 | *Leuconostoc* | 0.02799 | 0.00175 | 0.01937 | 0.00571 | Bacteria |
| 260 | *Desulfitobacterium* | 0.01152 | 0.00351 | 0.00484 | 0.03424 | Bacteria |
| 261 | *Peptoniphilus* | 0.01811 | 0.00701 | 0 | 0.02853 | Bacteria |
| 262 | *Rhodococcus* | 0 | 0.01052 | 0.01452 | 0.02853 | Bacteria |
| 263 | *Massilibacterium* | 0.00823 | 0.00526 | 0 | 0.03995 | Bacteria |
| 264 | *Zobellia* | 0 | 0 | 0.05326 | 0 | Bacteria |
| 265 | *Salipaludibacillus* | 0 | 0.00526 | 0.01937 | 0.02853 | Bacteria |
| 266 | *Brachymonas* | 0.00165 | 0.01227 | 0.00484 | 0.03424 | Bacteria |
| 267 | *Oleispira* | 0.00165 | 0.02454 | 0.00968 | 0.01712 | Bacteria |
| 268 | *Aerococcus* | 0.01152 | 0 | 0.02421 | 0.01712 | Bacteria |
| 269 | *Anaerosalibacter* | 0.01317 | 0 | 0.03389 | 0.00571 | Bacteria |
| 270 | *Thioalkalimicrobium* | 0 | 0.01227 | 0.02905 | 0.01141 | Bacteria |
| 271 | *Leptospira* | 0.00329 | 0.00701 | 0.01937 | 0.02283 | Bacteria |
| 272 | *Veillonella* | 0 | 0.00876 | 0.01452 | 0.02853 | Bacteria |
| 273 | *Tenacibaculum* | 0.00329 | 0 | 0.04841 | 0 | Bacteria |
| 274 | *Bosea* | 0 | 0.00351 | 0.01937 | 0.02853 | Bacteria |
| 275 | *Alkalibacterium* | 0.01976 | 0.00351 | 0.00484 | 0.02283 | Bacteria |
| 276 | *Ideonella* | 0 | 0.00351 | 0.02421 | 0.02283 | Bacteria |
| 277 | *Microvirga* | 0 | 0.01577 | 0.02905 | 0.00571 | Bacteria |
| 278 | *Tetragenococcus* | 0.00988 | 0.00701 | 0.00484 | 0.02853 | Bacteria |
| 279 | *Sinorhizobium* | 0.00165 | 0 | 0.04841 | 0 | Bacteria |
| 280 | *Clostridioides* | 0.01811 | 0 | 0.01452 | 0.01712 | Bacteria |
| 281 | *Phi29virus* | 0.04939 | 0 | 0 | 0 | Bacteria |
| 282 | *Marinococcus* | 0 | 0.00701 | 0.01937 | 0.02283 | Bacteria |
| 283 | *Jeotgalicoccus* | 0 | 0.00351 | 0 | 0.04565 | Bacteria |
| 284 | *Proteiniborus* | 0.00494 | 0.00351 | 0.02905 | 0.01141 | Bacteria |
| 285 | *Aquamicrobium* | 0 | 0.01402 | 0.02905 | 0.00571 | Viruses |
| 286 | *Maribacter* | 0 | 0 | 0.04841 | 0 | Bacteria |
| 287 | *Fusobacterium* | 0.02634 | 0 | 0.00484 | 0.01712 | Bacteria |
| 288 | *Chlamydia* | 0.00329 | 0.01227 | 0.00968 | 0.02283 | Bacteria |
| 289 | *Gallaecimonas* | 0 | 0.01052 | 0.01452 | 0.02283 | Bacteria |
| 290 | *Tepidimonas* | 0 | 0.01928 | 0 | 0.02853 | Bacteria |
| 291 | *Riemerella* | 0.00329 | 0 | 0.03873 | 0.00571 | Bacteria |
| 292 | *Pediococcus* | 0 | 0.00175 | 0 | 0.04565 | Bacteria |
| 293 | *Rhizobacter* | 0 | 0.01402 | 0.00484 | 0.02853 | Bacteria |
| 294 | *Limnobacter* | 0 | 0.01227 | 0.02905 | 0.00571 | Bacteria |
| 295 | *Cellvibrio* | 0 | 0.00876 | 0.00968 | 0.02853 | Bacteria |
| 296 | *Parvimonas* | 0.02305 | 0 | 0 | 0.02283 | Bacteria |
| 297 | *Enteractinococcus* | 0 | 0 | 0 | 0.04565 | Bacteria |
| 298 | *Oceanicola* | 0 | 0.00876 | 0.01937 | 0.01712 | Bacteria |
| 299 | *Ottowia* | 0 | 0.01402 | 0.01937 | 0.01141 | Bacteria |
| 300 | *Hydrogenoanaerobacterium* | 0.04116 | 0.00351 | 0 | 0 | Bacteria |
| 301 | *Hymenobacter* | 0 | 0 | 0.03873 | 0.00571 | Bacteria |
| 302 | *Psychroflexus* | 0 | 0 | 0.03873 | 0.00571 | Bacteria |
| 303 | *Ahrensia* | 0 | 0.01052 | 0.03389 | 0 | Bacteria |
| 304 | *Ferrimonas* | 0 | 0.00701 | 0.01452 | 0.02283 | Bacteria |
| 305 | *Roseomonas* | 0 | 0.01753 | 0.00968 | 0.01712 | Bacteria |
| 306 | *Pseudaminobacter* | 0 | 0.00526 | 0.03873 | 0 | Bacteria |
| 307 | *Roseburia* | 0.00659 | 0.00876 | 0 | 0.02853 | Bacteria |
| 308 | *Leadbetterella* | 0 | 0 | 0.04357 | 0 | Bacteria |
| 309 | *Salinimicrobium* | 0 | 0 | 0.04357 | 0 | Bacteria |
| 310 | *Colwellia* | 0 | 0.00351 | 0 | 0.03995 | Bacteria |
| 311 | *Nitrincola* | 0 | 0.01052 | 0.00968 | 0.02283 | Bacteria |
| 312 | *Alkaliphilus* | 0.00165 | 0 | 0.02421 | 0.01712 | Bacteria |
| 313 | *Kushneria* | 0.00165 | 0 | 0.02421 | 0.01712 | Bacteria |
| 314 | *Brevundimonas* | 0.00165 | 0.01227 | 0.02905 | 0 | Bacteria |
| 315 | *Pisciglobus* | 0.0428 | 0 | 0 | 0 | Bacteria |
| 316 | *Neptuniibacter* | 0.00329 | 0.00526 | 0 | 0.03424 | Bacteria |
| 317 | *Caenibacillus* | 0.01317 | 0.00351 | 0.01452 | 0.01141 | Bacteria |
| 318 | *Turicibacter* | 0.00165 | 0.00175 | 0.00484 | 0.03424 | Bacteria |
| 319 | *Labrenzia* | 0 | 0.01052 | 0.01452 | 0.01712 | Bacteria |
| 320 | *Methyloversatilis* | 0 | 0.01928 | 0 | 0.02283 | Bacteria |
| 321 | *Leeia* | 0 | 0.01402 | 0.00484 | 0.02283 | Bacteria |
| 322 | *Melaminivora* | 0 | 0 | 0.02421 | 0.01712 | Bacteria |
| 323 | *Hyphomicrobium* | 0 | 0.00876 | 0.00968 | 0.02283 | Bacteria |
| 324 | *Roseovarius* | 0 | 0.01928 | 0.00484 | 0.01712 | Bacteria |
| 325 | *Sporomusa* | 0.01482 | 0.00526 | 0.00968 | 0.01141 | Bacteria |
| 326 | *Pseudorhodoferax* | 0 | 0.01577 | 0.01937 | 0.00571 | Bacteria |
| 327 | *Dechloromonas* | 0 | 0.01227 | 0 | 0.02853 | Bacteria |
| 328 | *Thermoanaerobacter* | 0.00329 | 0.00351 | 0.00484 | 0.02853 | Bacteria |
| 329 | *Salegentibacter* | 0 | 0.00351 | 0.01937 | 0.01712 | Bacteria |
| 330 | *Azoarcus* | 0 | 0.01227 | 0.00484 | 0.02283 | Bacteria |
| 331 | *Verminephrobacter* | 0 | 0.00701 | 0.00968 | 0.02283 | Bacteria |
| 332 | *Halanaerobium* | 0.01152 | 0 | 0.00484 | 0.02283 | Bacteria |
| 333 | *Aurantimonas* | 0 | 0.00526 | 0.03389 | 0 | Bacteria |
| 334 | *Chlorobaculum* | 0 | 0.00526 | 0.03389 | 0 | Bacteria |
| 335 | *Edwardsiella* | 0 | 0.00351 | 0.02421 | 0.01141 | Bacteria |
| 336 | *Gottschalkia* | 0 | 0.00351 | 0.02421 | 0.01141 | Bacteria |
| 337 | *Propionivibrio* | 0 | 0.00175 | 0.01452 | 0.02283 | Bacteria |
| 338 | *Microbacterium* | 0.00659 | 0.01052 | 0.00484 | 0.01712 | Bacteria |
| 339 | *Salinisphaera* | 0 | 0.01052 | 0 | 0.02853 | Bacteria |
| 340 | *Tepidiphilus* | 0 | 0.02278 | 0.00484 | 0.01141 | Bacteria |
| 341 | *Facklamia* | 0.01482 | 0.00701 | 0 | 0.01712 | Bacteria |
| 342 | *Formosa* | 0 | 0 | 0.03873 | 0 | Bacteria |
| 343 | *Mariniphaga* | 0 | 0 | 0.03873 | 0 | Bacteria |
| 344 | *Mesonia* | 0 | 0 | 0.03873 | 0 | Bacteria |
| 345 | *Siansivirga* | 0 | 0 | 0.03873 | 0 | Bacteria |
| 346 | *Zhouia* | 0 | 0 | 0.03873 | 0 | Bacteria |
| 347 | *Andreprevotia* | 0 | 0.01052 | 0.00484 | 0.02283 | Bacteria |
| 348 | *Xenophilus* | 0 | 0.00701 | 0.01937 | 0.01141 | Bacteria |
| 349 | *Rummeliibacillus* | 0.00659 | 0.00351 | 0.00484 | 0.02283 | Bacteria |
| 350 | *Desemzia* | 0.02634 | 0 | 0 | 0.01141 | Bacteria |
| 351 | *Bizionia* | 0 | 0.00351 | 0.03389 | 0 | Bacteria |
| 352 | *Teredinibacter* | 0 | 0 | 0.01452 | 0.02283 | Bacteria |
| 353 | *Caldimonas* | 0 | 0.01227 | 0.01937 | 0.00571 | Bacteria |
| 354 | *Derxia* | 0 | 0.01227 | 0.01937 | 0.00571 | Bacteria |
| 355 | *Cardiobacterium* | 0 | 0.00876 | 0 | 0.02853 | Bacteria |
| 356 | *Salinivibrio* | 0 | 0.00876 | 0 | 0.02853 | Bacteria |
| 357 | *Ruminococcus* | 0.00165 | 0.00701 | 0 | 0.02853 | Bacteria |
| 358 | *Gelidibacter* | 0 | 0.00526 | 0.01452 | 0.01712 | Bacteria |
| 359 | *Neptunomonas* | 0 | 0.00351 | 0.00484 | 0.02853 | Bacteria |
| 360 | *Brucella* | 0 | 0.00175 | 0.02905 | 0.00571 | Bacteria |
| 361 | *Thermovibrio* | 0 | 0 | 0.01937 | 0.01712 | Bacteria |
| 362 | *Nitrococcus* | 0 | 0.00701 | 0.02905 | 0 | Bacteria |
| 363 | *Microvirgula* | 0 | 0.00526 | 0.01937 | 0.01141 | Bacteria |
| 364 | *Nosocomiicoccus* | 0 | 0.00175 | 0 | 0.03424 | Bacteria |
| 365 | *Plesiomonas* | 0 | 0 | 0.02421 | 0.01141 | Bacteria |
| 366 | *Francisella* | 0 | 0.00701 | 0 | 0.02853 | Bacteria |
| 367 | *Geobacter* | 0 | 0.00701 | 0 | 0.02853 | Bacteria |
| 368 | *Solimonas* | 0 | 0.00701 | 0 | 0.02853 | Bacteria |
| 369 | *Thorsellia* | 0 | 0.01402 | 0.00968 | 0.01141 | Bacteria |
| 370 | *Ferrovum* | 0 | 0.01227 | 0 | 0.02283 | Bacteria |
| 371 | *Zunongwangia* | 0 | 0 | 0.02905 | 0.00571 | Bacteria |
| 372 | *Butyrivibrio* | 0 | 0.00701 | 0.00484 | 0.02283 | Bacteria |
| 373 | *Desulfuribacillus* | 0.00659 | 0.00526 | 0 | 0.02283 | Bacteria |
| 374 | *Pelosinus* | 0.01152 | 0 | 0 | 0.02283 | Bacteria |
| 375 | *Saccharomonospora* | 0 | 0 | 0 | 0.03424 | Bacteria |
| 376 | *Frankia* | 0 | 0.01227 | 0.00484 | 0.01712 | Bacteria |
| 377 | *Cruoricaptor* | 0 | 0 | 0.03389 | 0 | Bacteria |
| 378 | *Solitalea* | 0 | 0 | 0.03389 | 0 | Bacteria |
| 379 | *Albimonas* | 0 | 0.00876 | 0.01937 | 0.00571 | Bacteria |
| 380 | *Ramlibacter* | 0 | 0.00701 | 0.00968 | 0.01712 | Bacteria |
| 381 | *Rubeoparvulum* | 0.00165 | 0.00351 | 0 | 0.02853 | Bacteria |
| 382 | *Hahella* | 0.00329 | 0.00351 | 0.00968 | 0.01712 | Bacteria |
| 383 | *Treponema* | 0.00329 | 0.00351 | 0.00968 | 0.01712 | Bacteria |
| 384 | *Pseudospirillum* | 0 | 0.00351 | 0.02421 | 0.00571 | Bacteria |
| 385 | *Halalkalibacillus* | 0 | 0 | 0.00484 | 0.02853 | Bacteria |
| 386 | *Allofustis* | 0.01482 | 0.00701 | 0 | 0.01141 | Bacteria |
| 387 | *Succinatimonas* | 0.00659 | 0.00351 | 0 | 0.02283 | Bacteria |
| 388 | *Pseudogulbenkiania* | 0.00329 | 0.01402 | 0.00968 | 0.00571 | Bacteria |
| 389 | *Histophilus* | 0 | 0 | 0.00968 | 0.02283 | Bacteria |
| 390 | *Rubrivivax* | 0 | 0.01227 | 0.01452 | 0.00571 | Bacteria |
| 391 | *Halothiobacillus* | 0 | 0.00526 | 0.00968 | 0.01712 | Bacteria |
| 392 | *Thalassospira* | 0 | 0.00526 | 0.00968 | 0.01712 | Bacteria |
| 393 | *Aliihoeflea* | 0 | 0.00175 | 0.02421 | 0.00571 | Bacteria |
| 394 | *Moellerella* | 0.00659 | 0 | 0.01937 | 0.00571 | Bacteria |
| 395 | *Alloiococcus* | 0 | 0 | 0.01452 | 0.01712 | Bacteria |
| 396 | *Nitrosococcus* | 0 | 0 | 0.01452 | 0.01712 | Bacteria |
| 397 | *Bermanella* | 0 | 0.00876 | 0 | 0.02283 | Bacteria |
| 398 | *Frischella* | 0 | 0.00876 | 0 | 0.02283 | Bacteria |
| 399 | *Tatumella* | 0 | 0.01402 | 0 | 0.01712 | Bacteria |
| 400 | *Algoriella* | 0 | 0.00175 | 0.02905 | 0 | Bacteria |
| 401 | *Vulcanibacillus* | 0 | 0 | 0.01937 | 0.01141 | Bacteria |
| 402 | *Methylomicrobium* | 0 | 0.01052 | 0.01452 | 0.00571 | Bacteria |
| 403 | *Bifidobacterium* | 0.00988 | 0.00526 | 0.00968 | 0.00571 | Bacteria |
| 404 | *Caulobacter* | 0 | 0.00175 | 0 | 0.02853 | Bacteria |
| 405 | *Kingella* | 0.00165 | 0.00175 | 0.00968 | 0.01712 | Bacteria |
| 406 | *Saccharibacillus* | 0.00494 | 0.01928 | 0 | 0.00571 | Bacteria |
| 407 | *Cytophaga* | 0 | 0 | 0.02421 | 0.00571 | Bacteria |
| 408 | *Phyllobacterium* | 0 | 0 | 0.02421 | 0.00571 | Bacteria |
| 409 | *Amphritea* | 0 | 0.00701 | 0 | 0.02283 | Bacteria |
| 410 | *Alysiella* | 0 | 0.02979 | 0 | 0 | Bacteria |
| 411 | *Halorhodospira* | 0 | 0.00526 | 0.02421 | 0 | Bacteria |
| 412 | *Thiocapsa* | 0 | 0.00351 | 0.01452 | 0.01141 | Bacteria |
| 413 | *Planomicrobium* | 0 | 0.00175 | 0.00484 | 0.02283 | Bacteria |
| 414 | *Lawsonia* | 0 | 0.01227 | 0 | 0.01712 | Bacteria |
| 415 | *Uliginosibacterium* | 0 | 0.01227 | 0 | 0.01712 | Bacteria |
| 416 | *Arenibacter* | 0 | 0 | 0.02905 | 0 | Bacteria |
| 417 | *Gaetbulibacter* | 0 | 0 | 0.02905 | 0 | Bacteria |
| 418 | *Lacinutrix* | 0 | 0 | 0.02905 | 0 | Viruses |
| 419 | *Psychroserpens* | 0 | 0 | 0.02905 | 0 | Bacteria |
| 420 | *Pustulibacterium* | 0 | 0 | 0.02905 | 0 | Bacteria |
| 421 | *Ulvibacter* | 0 | 0 | 0.02905 | 0 | Bacteria |
| 422 | *Acidihalobacter* | 0 | 0.00701 | 0.00484 | 0.01712 | Bacteria |
| 423 | *Devosia* | 0 | 0.00701 | 0.00484 | 0.01712 | Bacteria |
| 424 | *Dyella* | 0 | 0.00175 | 0.00968 | 0.01712 | Bacteria |
| 425 | *Pseudovibrio* | 0 | 0.00175 | 0.00968 | 0.01712 | Bacteria |
| 426 | *Brevibacterium* | 0 | 0 | 0 | 0.02853 | Bacteria |
| 427 | *Coxiella* | 0 | 0 | 0 | 0.02853 | Bacteria |
| 428 | *Magnetovibrio* | 0 | 0 | 0 | 0.02853 | Bacteria |
| 429 | *Salibacterium* | 0 | 0 | 0 | 0.02853 | Bacteria |
| 430 | *Yaniella* | 0 | 0 | 0 | 0.02853 | Bacteria |
| 431 | *N4virus* | 0 | 0 | 0 | 0.02853 | Bacteria |
| 432 | *Sporanaerobacter* | 0.00494 | 0.00701 | 0.00484 | 0.01141 | Bacteria |
| 433 | *Nitrobacter* | 0 | 0.00876 | 0.01937 | 0 | Bacteria |
| 434 | *Pseudohongiella* | 0 | 0.00526 | 0 | 0.02283 | Bacteria |
| 435 | *Anaerobium* | 0.02799 | 0 | 0 | 0 | Bacteria |
| 436 | *Nitrospira* | 0.00494 | 0 | 0 | 0.02283 | Bacteria |
| 437 | *Bibersteinia* | 0 | 0.00175 | 0.01452 | 0.01141 | Bacteria |
| 438 | *Proteiniphilum* | 0 | 0.00175 | 0.01452 | 0.01141 | Bacteria |
| 439 | *Budvicia* | 0 | 0 | 0.00484 | 0.02283 | Bacteria |
| 440 | *Enterovibrio* | 0 | 0 | 0.00484 | 0.02283 | Bacteria |
| 441 | *Yangia* | 0 | 0.01227 | 0.00968 | 0.00571 | Bacteria |
| 442 | *Megasphaera* | 0 | 0.01052 | 0 | 0.01712 | Bacteria |
| 443 | *Weissella* | 0.00823 | 0.00175 | 0 | 0.01712 | Bacteria |
| 444 | *Grimontia* | 0.00329 | 0.00175 | 0.00484 | 0.01712 | Bacteria |
| 445 | *Meiothermus* | 0 | 0.00175 | 0.01937 | 0.00571 | Bacteria |
| 446 | *Shimwellia* | 0 | 0 | 0.00968 | 0.01712 | Bacteria |
| 447 | *Actinomyces* | 0 | 0.01052 | 0.00484 | 0.01141 | Bacteria |
| 448 | *Azohydromonas* | 0 | 0.01052 | 0.00484 | 0.01141 | Bacteria |
| 449 | *Oceanobacter* | 0 | 0.00351 | 0 | 0.02283 | Bacteria |
| 450 | *Stenoxybacter* | 0 | 0.00351 | 0 | 0.02283 | Bacteria |
| 451 | *Marinimicrobium* | 0 | 0.01577 | 0.00484 | 0.00571 | Bacteria |
| 452 | *Numidum* | 0.00329 | 0 | 0 | 0.02283 | Bacteria |
| 453 | *Fructobacillus* | 0.01152 | 0.00876 | 0 | 0.00571 | Bacteria |
| 454 | *Chishuiella* | 0 | 0 | 0.01452 | 0.01141 | Bacteria |
| 455 | *Kangiella* | 0 | 0 | 0.01452 | 0.01141 | Bacteria |
| 456 | *Tropicibacter* | 0 | 0 | 0.01452 | 0.01141 | Bacteria |
| 457 | *Chitinilyticum* | 0 | 0.01052 | 0.00968 | 0.00571 | Bacteria |
| 458 | *Buttiauxella* | 0 | 0.00876 | 0 | 0.01712 | Bacteria |
| 459 | *Aureimonas* | 0 | 0.02103 | 0.00484 | 0 | Bacteria |
| 460 | *Selenomonas* | 0.00165 | 0.00701 | 0 | 0.01712 | Bacteria |
| 461 | *Azorhizobium* | 0 | 0.00526 | 0.01452 | 0.00571 | Bacteria |
| 462 | *Mucor* | 0.00165 | 0.00351 | 0.01452 | 0.00571 | Bacteria |
| 463 | *Bergeyella* | 0 | 0 | 0.01937 | 0.00571 | Eukaryota |
| 464 | *Cyclobacterium* | 0 | 0 | 0.01937 | 0.00571 | Bacteria |
| 465 | *Leptolyngbya* | 0 | 0 | 0.01937 | 0.00571 | Bacteria |
| 466 | *Rufibacter* | 0 | 0 | 0.01937 | 0.00571 | Bacteria |
| 467 | *Ea92virus* | 0 | 0 | 0.01937 | 0.00571 | Bacteria |
| 468 | *Limnohabitans* | 0 | 0.00876 | 0.00484 | 0.01141 | Bacteria |
| 469 | *Micromonospora* | 0 | 0.00876 | 0.00484 | 0.01141 | Bacteria |
| 470 | *Cycloclasticus* | 0.00329 | 0.00701 | 0.01452 | 0 | Viruses |
| 471 | *Afipia* | 0 | 0.00351 | 0.00968 | 0.01141 | Bacteria |
| 472 | *Leucothrix* | 0 | 0.00351 | 0.00968 | 0.01141 | Bacteria |
| 473 | *Gardnerella* | 0.01317 | 0 | 0 | 0.01141 | Bacteria |
| 474 | *Cohnella* | 0.00165 | 0 | 0 | 0.02283 | Bacteria |
| 475 | *Leeuwenhoekiella* | 0 | 0 | 0.02421 | 0 | Bacteria |
| 476 | *Neorhizobium* | 0 | 0 | 0.02421 | 0 | Bacteria |
| 477 | *Thermonema* | 0 | 0 | 0.02421 | 0 | Bacteria |
| 478 | *Kaistia* | 0 | 0.00876 | 0.00968 | 0.00571 | Bacteria |
| 479 | *Endozoicomonas* | 0 | 0.00701 | 0 | 0.01712 | Bacteria |
| 480 | *Methylophilus* | 0 | 0.00701 | 0 | 0.01712 | Bacteria |
| 481 | *Fangia* | 0.00165 | 0.00526 | 0 | 0.01712 | Bacteria |
| 482 | *Garciella* | 0.00165 | 0.00526 | 0 | 0.01712 | Bacteria |
| 483 | *Actinobacillus* | 0 | 0.00175 | 0.00484 | 0.01712 | Bacteria |
| 484 | *Ectothiorhodosinus* | 0 | 0.00175 | 0.00484 | 0.01712 | Bacteria |
| 485 | *Caloramator* | 0.00659 | 0 | 0 | 0.01712 | Bacteria |
| 486 | *Gemella* | 0.00659 | 0 | 0 | 0.01712 | Bacteria |
| 487 | *Beggiatoa* | 0 | 0.01402 | 0.00968 | 0 | Bacteria |
| 488 | *Oxalobacter* | 0 | 0.01227 | 0 | 0.01141 | Bacteria |
| 489 | *Thalassotalea* | 0.00165 | 0.00175 | 0.01452 | 0.00571 | Bacteria |
| 490 | *Pragia* | 0.00329 | 0 | 0.01452 | 0.00571 | Bacteria |
| 491 | *Ensifer* | 0 | 0.00701 | 0.00484 | 0.01141 | Bacteria |
| 492 | *Carnimonas* | 0 | 0.00351 | 0.01937 | 0 | Bacteria |
| 493 | *Woeseia* | 0 | 0.00175 | 0.00968 | 0.01141 | Bacteria |
| 494 | *Hydrocarboniphaga* | 0 | 0.01227 | 0.00484 | 0.00571 | Bacteria |
| 495 | *Varibaculum* | 0.00165 | 0 | 0.00968 | 0.01141 | Bacteria |
| 496 | *Helicobacter* | 0 | 0.00526 | 0 | 0.01712 | Bacteria |
| 497 | *Phascolarctobacterium* | 0.00165 | 0.00351 | 0 | 0.01712 | Bacteria |
| 498 | *Salsuginibacillus* | 0.00329 | 0.00175 | 0 | 0.01712 | Bacteria |
| 499 | *Pseudorhodobacter* | 0 | 0.00175 | 0.01452 | 0.00571 | Bacteria |
| 500 | *Azospira* | 0 | 0 | 0.00484 | 0.01712 | Bacteria |
| 501 | *Moritella* | 0 | 0 | 0.00484 | 0.01712 | Bacteria |
| 502 | *Dialister* | 0 | 0.01052 | 0 | 0.01141 | Bacteria |
| 503 | *Methylobacter* | 0 | 0.00526 | 0.00484 | 0.01141 | Bacteria |
| 504 | *Methylotenera* | 0 | 0.00526 | 0.00484 | 0.01141 | Bacteria |
| 505 | *Pelomonas* | 0 | 0.00526 | 0.00484 | 0.01141 | Bacteria |
| 506 | *Rhodopseudomonas* | 0 | 0.00526 | 0.00484 | 0.01141 | Bacteria |
| 507 | *Hungatella* | 0.00988 | 0 | 0 | 0.01141 | Bacteria |
| 508 | *Arcobacter* | 0.00494 | 0 | 0.00484 | 0.01141 | Bacteria |
| 509 | *Methylococcus* | 0 | 0.00175 | 0.01937 | 0 | Bacteria |
| 510 | *Sinomicrobium* | 0 | 0.00175 | 0.01937 | 0 | Bacteria |
| 511 | *Tamlana* | 0 | 0.00175 | 0.01937 | 0 | Bacteria |
| 512 | *Bilophila* | 0 | 0 | 0.00968 | 0.01141 | Bacteria |
| 513 | *Flexilinea* | 0 | 0 | 0.00968 | 0.01141 | Bacteria |
| 514 | *Nocardia* | 0 | 0 | 0.00968 | 0.01141 | Bacteria |
| 515 | *Vogesella* | 0 | 0.00526 | 0.00968 | 0.00571 | Bacteria |
| 516 | *Enhydrobacter* | 0 | 0.00351 | 0 | 0.01712 | Bacteria |
| 517 | *Phormidium* | 0 | 0.00351 | 0 | 0.01712 | Bacteria |
| 518 | *Asticcacaulis* | 0.00329 | 0 | 0 | 0.01712 | Bacteria |
| 519 | *Flavisolibacter* | 0 | 0 | 0.01452 | 0.00571 | Bacteria |
| 520 | *Galbibacter* | 0 | 0 | 0.01452 | 0.00571 | Bacteria |
| 521 | *Paramaledivibacter* | 0 | 0 | 0.01452 | 0.00571 | Bacteria |
| 522 | *Sediminibacterium* | 0 | 0 | 0.01452 | 0.00571 | Bacteria |
| 523 | *Thermomonas* | 0 | 0 | 0.01452 | 0.00571 | Bacteria |
| 524 | *Chitinimonas* | 0 | 0.00876 | 0 | 0.01141 | Bacteria |
| 525 | *Spiribacter* | 0 | 0.00876 | 0 | 0.01141 | Bacteria |
| 526 | *Crenobacter* | 0 | 0.00526 | 0.01452 | 0 | Bacteria |
| 527 | *Lonsdalea* | 0.01317 | 0.00175 | 0.00484 | 0 | Bacteria |
| 528 | *Methylocaldum* | 0 | 0.00351 | 0.00484 | 0.01141 | Bacteria |
| 529 | *Catonella* | 0.01976 | 0 | 0 | 0 | Bacteria |
| 530 | *Atopobium* | 0.00823 | 0 | 0 | 0.01141 | Archaea |
| 531 | *Kallipyga* | 0.00823 | 0 | 0 | 0.01141 | Bacteria |
| 532 | *Leptotrichia* | 0.00823 | 0 | 0 | 0.01141 | Bacteria |
| 533 | *Methanobrevibacter* | 0.00823 | 0 | 0 | 0.01141 | Bacteria |
| 534 | *Adhaeribacter* | 0 | 0 | 0.01937 | 0 | Bacteria |
| 535 | *Aequorivita* | 0 | 0 | 0.01937 | 0 | Bacteria |
| 536 | *Flexithrix* | 0 | 0 | 0.01937 | 0 | Bacteria |
| 537 | *Imtechella* | 0 | 0 | 0.01937 | 0 | Bacteria |
| 538 | *Niastella* | 0 | 0 | 0.01937 | 0 | Bacteria |
| 539 | *Olleya* | 0 | 0 | 0.01937 | 0 | Bacteria |
| 540 | *Runella* | 0 | 0 | 0.01937 | 0 | Bacteria |
| 541 | *Sporocytophaga* | 0 | 0 | 0.01937 | 0 | Bacteria |
| 542 | *Winogradskyella* | 0 | 0 | 0.01937 | 0 | Bacteria |
| 543 | *Rhodovulum* | 0 | 0.00876 | 0.00484 | 0.00571 | Bacteria |
| 544 | *Ruegeria* | 0 | 0.00876 | 0.00484 | 0.00571 | Bacteria |
| 545 | *Acidithiobacillus* | 0 | 0.00351 | 0.00968 | 0.00571 | Bacteria |
| 546 | *Agrobacterium* | 0 | 0.00175 | 0 | 0.01712 | Bacteria |
| 547 | *Erythrobacter* | 0 | 0.00175 | 0 | 0.01712 | Bacteria |
| 548 | *Laribacter* | 0 | 0.00175 | 0 | 0.01712 | Bacteria |
| 549 | *Muribacter* | 0 | 0.00175 | 0 | 0.01712 | Bacteria |
| 550 | *Pelotomaculum* | 0 | 0.00175 | 0 | 0.01712 | Bacteria |
| 551 | *Rhodobacter* | 0 | 0.01402 | 0.00484 | 0 | Bacteria |
| 552 | *Oceanibaculum* | 0.00165 | 0.00175 | 0.00968 | 0.00571 | Bacteria |
| 553 | *Planifilum* | 0.00165 | 0 | 0 | 0.01712 | Bacteria |
| 554 | *Coprococcus* | 0.00659 | 0.00701 | 0.00484 | 0 | Bacteria |
| 555 | *Desulfuromonas* | 0 | 0.00701 | 0 | 0.01141 | Bacteria |
| 556 | *Desulfobacterium* | 0.00494 | 0.00175 | 0 | 0.01141 | Bacteria |
| 557 | *Thermoanaerobacterium* | 0.00494 | 0.00175 | 0 | 0.01141 | Bacteria |
| 558 | *Chania* | 0 | 0.00351 | 0.01452 | 0 | Bacteria |
| 559 | *Bergeriella* | 0 | 0.00175 | 0.00484 | 0.01141 | Bacteria |
| 560 | *Catenovulum* | 0 | 0.00175 | 0.00484 | 0.01141 | Bacteria |
| 561 | *Cobetia* | 0 | 0.00175 | 0.00484 | 0.01141 | Bacteria |
| 562 | *Desulfobulbus* | 0 | 0.00175 | 0.00484 | 0.01141 | Bacteria |
| 563 | *Moorella* | 0 | 0.00175 | 0.00484 | 0.01141 | Bacteria |
| 564 | *Pseudacidovorax* | 0 | 0.00175 | 0.00484 | 0.01141 | Bacteria |
| 565 | *Rubritalea* | 0 | 0.00175 | 0.00484 | 0.01141 | Bacteria |
| 566 | *Synechococcus* | 0 | 0.00175 | 0.00484 | 0.01141 | Bacteria |
| 567 | *Aquisalimonas* | 0 | 0.00701 | 0.00484 | 0.00571 | Bacteria |
| 568 | *Rickettsiella* | 0 | 0.00701 | 0.00484 | 0.00571 | Bacteria |
| 569 | *Sulfitobacter* | 0 | 0.00701 | 0.00484 | 0.00571 | Bacteria |
| 570 | *Chromohalobacter* | 0 | 0.00175 | 0.00968 | 0.00571 | Bacteria |
| 571 | *Lyngbya* | 0 | 0.00175 | 0.00968 | 0.00571 | Bacteria |
| 572 | *Planctomyces* | 0 | 0.00175 | 0.00968 | 0.00571 | Bacteria |
| 573 | *Pseudooceanicola* | 0 | 0.00175 | 0.00968 | 0.00571 | Bacteria |
| 574 | *Sedimenticola* | 0 | 0.00175 | 0.00968 | 0.00571 | Bacteria |
| 575 | *Sphingopyxis* | 0 | 0.00175 | 0.00968 | 0.00571 | Bacteria |
| 576 | *Tepidibacter* | 0 | 0.00175 | 0.00968 | 0.00571 | Bacteria |
| 577 | *Sodalis* | 0 | 0.01227 | 0.00484 | 0 | Bacteria |
| 578 | *Granulicatella* | 0.00988 | 0.00701 | 0 | 0 | Bacteria |
| 579 | *Eremococcus* | 0.00494 | 0.00701 | 0.00484 | 0 | Bacteria |
| 580 | *Allochromatium* | 0 | 0.00701 | 0.00968 | 0 | Bacteria |
| 581 | *Xylella* | 0 | 0.00701 | 0.00968 | 0 | Bacteria |
| 582 | *Amorphus* | 0 | 0.00526 | 0 | 0.01141 | Bacteria |
| 583 | *Donghicola* | 0 | 0.00526 | 0 | 0.01141 | Bacteria |
| 584 | *Ferriphaselus* | 0 | 0.00526 | 0 | 0.01141 | Bacteria |
| 585 | *Rappaport* | 0 | 0.00526 | 0 | 0.01141 | Bacteria |
| 586 | *Zoogloea* | 0 | 0.00526 | 0 | 0.01141 | Eukaryota |
| 587 | *Coprobacillus* | 0.00165 | 0.00351 | 0 | 0.01141 | Bacteria |
| 588 | *Anaerotruncus* | 0.01646 | 0 | 0 | 0 | Bacteria |
| 589 | *Spirochaeta* | 0.00494 | 0 | 0 | 0.01141 | Bacteria |
| 590 | *Angomonas* | 0 | 0 | 0.00484 | 0.01141 | Bacteria |
| 591 | *Dokdonella* | 0 | 0.01052 | 0 | 0.00571 | Bacteria |
| 592 | *Sideroxydans* | 0 | 0.01052 | 0 | 0.00571 | Bacteria |
| 593 | *Fulvimarina* | 0 | 0.00526 | 0.00484 | 0.00571 | Bacteria |
| 594 | *Xylophilus* | 0 | 0.01052 | 0.00484 | 0 | Bacteria |
| 595 | *Aquitalea* | 0 | 0.00351 | 0 | 0.01141 | Bacteria |
| 596 | *Beijerinckia* | 0 | 0.00351 | 0 | 0.01141 | Bacteria |
| 597 | *Belnapia* | 0 | 0.00351 | 0 | 0.01141 | Bacteria |
| 598 | *Pseudanabaena* | 0 | 0.00351 | 0 | 0.01141 | Bacteria |
| 599 | *Reinekea* | 0 | 0.00351 | 0 | 0.01141 | Bacteria |
| 600 | *Tolumonas* | 0 | 0.00351 | 0 | 0.01141 | Bacteria |
| 601 | *Thalassolituus* | 0.00165 | 0.00351 | 0.00968 | 0 | Bacteria |
| 602 | *Spirosoma* | 0.00165 | 0.00175 | 0 | 0.01141 | Bacteria |
| 603 | *Geosporobacter* | 0.00329 | 0 | 0 | 0.01141 | Bacteria |
| 604 | *Melissococcus* | 0.00329 | 0 | 0 | 0.01141 | Bacteria |
| 605 | *Risungbinella* | 0.00329 | 0 | 0 | 0.01141 | Bacteria |
| 606 | *Aeromicrobium* | 0 | 0.00876 | 0 | 0.00571 | Bacteria |
| 607 | *Methylovorus* | 0 | 0.00876 | 0 | 0.00571 | Bacteria |
| 608 | *Nocardioides* | 0 | 0.00876 | 0 | 0.00571 | Bacteria |
| 609 | *Rouxiella* | 0 | 0.00876 | 0 | 0.00571 | Bacteria |
| 610 | *Sulfuricaulis* | 0 | 0.00876 | 0 | 0.00571 | Bacteria |
| 611 | *Atopobacter* | 0.00494 | 0.00351 | 0 | 0.00571 | Bacteria |
| 612 | *Alkanindiges* | 0 | 0.00351 | 0.00484 | 0.00571 | Bacteria |
| 613 | *Paraglaciecola* | 0 | 0.00351 | 0.00484 | 0.00571 | Bacteria |
| 614 | *Roseivivax* | 0 | 0.00351 | 0.00484 | 0.00571 | Bacteria |
| 615 | *Exiguobacterium* | 0 | 0.01402 | 0 | 0 | Bacteria |
| 616 | *Carboxydocella* | 0 | 0.01402 | 0 | 0 | Bacteria |
| 617 | *Thermoflavimicrobium* | 0.00329 | 0 | 0.00484 | 0.00571 | Bacteria |
| 618 | *Sphaerochaeta* | 0.00659 | 0.00701 | 0 | 0 | Eukaryota |
| 619 | *Planktothrix* | 0.00494 | 0.00351 | 0.00484 | 0 | Bacteria |
| 620 | *Anaerocolumna* | 0.01152 | 0.00175 | 0 | 0 | Bacteria |
| 621 | *Aminobacter* | 0 | 0.00351 | 0.00968 | 0 | Bacteria |
| 622 | *Caloranaerobacter* | 0 | 0.00351 | 0.00968 | 0 | Bacteria |
| 623 | *Lamprocystis* | 0 | 0.00351 | 0.00968 | 0 | Bacteria |
| 624 | *Methylosarcina* | 0 | 0.00351 | 0.00968 | 0 | Bacteria |
| 625 | *Piscirickettsia* | 0 | 0.00351 | 0.00968 | 0 | Bacteria |
| 626 | *Celeribacter* | 0.00659 | 0.00175 | 0.00484 | 0 | Bacteria |
| 627 | *Spo1virus* | 0.01317 | 0 | 0 | 0 | Bacteria |
| 628 | *Aspergillus* | 0.00165 | 0 | 0 | 0.01141 | Viruses |
| 629 | *Glaciecola* | 0 | 0.00701 | 0 | 0.00571 | Bacteria |
| 630 | *Herminiimonas* | 0 | 0.00701 | 0 | 0.00571 | Bacteria |
| 631 | *Mitsuaria* | 0 | 0.00701 | 0 | 0.00571 | Bacteria |
| 632 | *Nitrosovibrio* | 0 | 0.00701 | 0 | 0.00571 | Bacteria |
| 633 | *Sphaerotilus* | 0 | 0.00701 | 0 | 0.00571 | Bacteria |
| 634 | *Azovibrio* | 0 | 0.01227 | 0 | 0 | Archaea |
| 635 | *Kroppenstedtia* | 0 | 0.01227 | 0 | 0 | Bacteria |
| 636 | *Thermobacillus* | 0 | 0.01227 | 0 | 0 | Bacteria |
| 637 | *Methanobacterium* | 0.00165 | 0 | 0.00484 | 0.00571 | Bacteria |
| 638 | *Gayadomonas* | 0 | 0.00701 | 0.00484 | 0 | Eukaryota |
| 639 | *Rhodoplanes* | 0 | 0.00701 | 0.00484 | 0 | Eukaryota |
| 640 | *Ignavigranum* | 0.01152 | 0 | 0 | 0 | Eukaryota |
| 641 | *Youngiibacter* | 0.01152 | 0 | 0 | 0 | Bacteria |
| 642 | *Acytostelium* | 0 | 0 | 0 | 0.01141 | Bacteria |
| 643 | *Guillardia* | 0 | 0 | 0 | 0.01141 | Bacteria |
| 644 | *Thalassiosira* | 0 | 0 | 0 | 0.01141 | Bacteria |
| 645 | *Desulfitibacter* | 0 | 0.00526 | 0 | 0.00571 | Bacteria |
| 646 | *Diaphorobacter* | 0 | 0.00526 | 0 | 0.00571 | Bacteria |
| 647 | *Giesbergeria* | 0 | 0.00526 | 0 | 0.00571 | Bacteria |
| 648 | *Desulfovermiculus* | 0 | 0.01052 | 0 | 0 | Bacteria |
| 649 | *Geoalkalibacter* | 0 | 0.01052 | 0 | 0 | Bacteria |
| 650 | *Mycoplasma* | 0.00329 | 0.00701 | 0 | 0 | Bacteria |
| 651 | *Acidisphaera* | 0 | 0.00526 | 0.00484 | 0 | Bacteria |
| 652 | *Aquincola* | 0 | 0.00526 | 0.00484 | 0 | Bacteria |
| 653 | *Arenimonas* | 0 | 0.00526 | 0.00484 | 0 | Bacteria |
| 654 | *Chitiniphilus* | 0 | 0.00526 | 0.00484 | 0 | Bacteria |
| 655 | *Necropsobacter* | 0 | 0.00526 | 0.00484 | 0 | Bacteria |
| 656 | *Twortvirus* | 0 | 0 | 0.00968 | 0 | Bacteria |
| 657 | *Gordonia* | 0 | 0.00876 | 0 | 0 | Bacteria |
| 658 | *Phenylobacterium* | 0 | 0.00876 | 0 | 0 | Bacteria |
| 659 | *Pseudoclavibacter* | 0 | 0.00876 | 0 | 0 | Bacteria |
| 660 | *Shimia* | 0 | 0.00876 | 0 | 0 | Bacteria |
| 661 | *Thermithiobacillus* | 0 | 0.00876 | 0 | 0 | Bacteria |
| 662 | *Gonapodya* | 0 | 0.00876 | 0 | 0 | Eukaryota |
| 663 | *Dethiosulfatarculus* | 0.00659 | 0.00175 | 0 | 0 | Bacteria |
| 664 | *Halapricum* | 0 | 0.00175 | 0 | 0.00571 | Archaea |
| 665 | *Natrialba* | 0 | 0.00175 | 0 | 0.00571 | Archaea |
| 666 | *Loktanella* | 0 | 0.00701 | 0 | 0 | Bacteria |
| 667 | *Nostoc* | 0 | 0.00701 | 0 | 0 | Bacteria |
| 668 | *Syntrophorhabdus* | 0 | 0.00701 | 0 | 0 | Bacteria |
| 669 | *Thiohalocapsa* | 0 | 0.00701 | 0 | 0 | Bacteria |
| 670 | *Tistlia* | 0 | 0.00701 | 0 | 0 | Bacteria |
| 671 | *Pseudobutyrivibrio* | 0.00494 | 0.00175 | 0 | 0 | Bacteria |
| 672 | *Acetivibrio* | 0.00659 | 0 | 0 | 0 | Bacteria |
| 673 | *Oenococcus* | 0.00659 | 0 | 0 | 0 | Bacteria |
| 674 | *Pedosphaera* | 0.00659 | 0 | 0 | 0 | Bacteria |
| 675 | *Halovenus* | 0 | 0 | 0 | 0.00571 | Archaea |
| 676 | *Ignisphaera* | 0 | 0 | 0 | 0.00571 | Archaea |
| 677 | *Bathycoccus* | 0 | 0 | 0 | 0.00571 | Eukaryota |
| 678 | *Choanephora* | 0 | 0 | 0 | 0.00571 | Eukaryota |
| 679 | *Claviceps* | 0 | 0 | 0 | 0.00571 | Eukaryota |
| 680 | *Encephalitozoon* | 0 | 0 | 0 | 0.00571 | Eukaryota |
| 681 | *Malassezia* | 0 | 0 | 0 | 0.00571 | Eukaryota |
| 682 | *Membranoptera* | 0 | 0 | 0 | 0.00571 | Eukaryota |
| 683 | *Mixia* | 0 | 0 | 0 | 0.00571 | Eukaryota |
| 684 | *Penicillium* | 0 | 0 | 0 | 0.00571 | Eukaryota |
| 685 | *Perkinsus* | 0 | 0 | 0 | 0.00571 | Eukaryota |
| 686 | *Phytophthora* | 0 | 0 | 0 | 0.00571 | Eukaryota |
| 687 | *Pseudocercospora* | 0 | 0 | 0 | 0.00571 | Eukaryota |
| 688 | *Pseudogymnoascus* | 0 | 0 | 0 | 0.00571 | Eukaryota |
| 689 | *Puccinia* | 0 | 0 | 0 | 0.00571 | Eukaryota |
| 690 | *Rhizophagus* | 0 | 0 | 0 | 0.00571 | Eukaryota |
| 691 | *Tetrahymena* | 0 | 0 | 0 | 0.00571 | Eukaryota |
| 692 | *Zygosaccharomyces* | 0 | 0 | 0 | 0.00571 | Eukaryota |
| 693 | *Rdjlvirus* | 0 | 0 | 0 | 0.00571 | Viruses |
| 694 | *Bc431virus* | 0.00494 | 0 | 0 | 0 | Viruses |
| 695 | *Archaeoglobus* | 0 | 0 | 0.00484 | 0 | Archaea |
| 696 | *Methanocaldococcus* | 0 | 0 | 0.00484 | 0 | Archaea |
| 697 | *Endocarpon* | 0 | 0 | 0.00484 | 0 | Eukaryota |
| 698 | *Halococcus* | 0.00165 | 0.00175 | 0 | 0 | Archaea |
| 699 | *Lambdavirus* | 0.00329 | 0 | 0 | 0 | Viruses |
| 700 | *Halolamina* | 0 | 0.00175 | 0 | 0 | Archaea |
| 701 | *Haloprofundus* | 0 | 0.00175 | 0 | 0 | Archaea |
| 702 | *Methanoregula* | 0 | 0.00175 | 0 | 0 | Archaea |
| 703 | *Natronorubrum* | 0 | 0.00175 | 0 | 0 | Archaea |
| 704 | *Coccomyxa* | 0 | 0.00175 | 0 | 0 | Eukaryota |
| 705 | *Coniochaeta* | 0 | 0.00175 | 0 | 0 | Eukaryota |
| 706 | *Dacryopinax* | 0 | 0.00175 | 0 | 0 | Eukaryota |
| 707 | *Pseudocohnilembus* | 0 | 0.00175 | 0 | 0 | Eukaryota |
| 708 | *Talaromyces* | 0 | 0.00175 | 0 | 0 | Eukaryota |
| 709 | *Pyrococcus* | 0.00165 | 0 | 0 | 0 | Archaea |
| 710 | *Acanthamoeba* | 0.00165 | 0 | 0 | 0 | Eukaryota |
| 711 | *Tsarbombavirus* | 0.00165 | 0 | 0 | 0 | Viruses |
| 712 | unclassified archaeal genera | 0 | 0.00175 | 0.00968 | 0.00571 |  |
| 713 | unclassified eukaryotic genera | 0 | 0.00175 | 0 | 0 |  |
| 714 | unclassified bacterial genera | 0.12183 | 0.32597 | 1.00702 | 0.82744 |  |
| 715 | unclassified viral genera | 1.12114 | 0.20855 | 0.02421 | 0.23967 |  |
