# Supplementary material for: Shotgun sequence-based metataxonomic and predictive functional profiles of Pe poke, a naturally fermented soybean food of Myanmar
Source: PLoS One. 2021 Dec 17;16(12):e0260777. doi: 10.1371/journal.pone.0260777 (PMC8682898; doi:10.1371/journal.pone.0260777)
Supplement: S4 Table — (DOCX) [file pone.0260777.s004.docx]

**Supplementary Table 4.** The minor species with a relative abundance of <1% detected in *pe poke*.

| Sl. No. | species | Relative abundance (%) | | |  | Domain |
| --- | --- | --- | --- | --- | --- | --- |
|  |  | 3ds | 4ds | 5ds | Sds |  |
| 1 | *Wohlfahrtiimonas chitiniclastica* | 0 | 0.383047 | 1.6515 | 1.652479 | Bacteria |
| 2 | *Bacillus glycinifermentans* | 0 | 2.726877 | 0 | 0.175263 | Bacteria |
| 3 | *Flavobacterium marinum* | 0 | 0.004141 | 2.858106 | 0.008346 | Bacteria |
| 4 | *Sporosarcina koreensis* | 0.057317 | 0.031058 | 2.123357 | 0.634285 | Bacteria |
| 5 | *Luteimonas* sp. JM171 | 0 | 0.006212 | 2.743512 | 0.008346 | Bacteria |
| 6 | *Alcaligenes faecalis* | 0 | 0.507278 | 1.469498 | 0.333834 | Bacteria |
| 7 | *Flavobacterium ummariense* | 0 | 0.037269 | 2.237951 | 0 | Bacteria |
| 8 | *Wohlfahrtiimonas* sp. 34C10-3-10 | 0.016858 | 0.178065 | 0.721267 | 1.335336 | Bacteria |
| 9 | *Pusillimonas* sp. T7-7 | 0 | 0.546618 | 1.024604 | 0.575864 | Bacteria |
| 10 | *Bacillus licheniformis* | 0.986193 | 0.592169 | 0.397708 | 0.108496 | Bacteria |
| 11 | *Pusillimonas noertemannii* | 0 | 0.501066 | 0.869565 | 0.525789 | Bacteria |
| 12 | *Candidimonas bauzanensis* | 0 | 0.465867 | 0.680822 | 0.517443 | Bacteria |
| 13 | *Bacillus andreraoultii* | 0.620375 | 0.703977 | 0.107853 | 0.216992 | Bacteria |
| 14 | *Wohlfahrtiimonas larvae* | 0.016858 | 0.043481 | 0.552747 | 0.959773 | Bacteria |
| 15 | *Lysinibacillus sphaericus* | 0.006743 | 0.024846 | 0.262892 | 1.26857 | Bacteria |
| 16 | *Kerstersia gyiorum* | 0.003372 | 0.383047 | 0.788675 | 0.367217 | Bacteria |
| 17 | *Oblitimonas alkaliphila* | 0 | 0.31886 | 0.984159 | 0.191955 | Bacteria |
| 18 | *Vagococcus lutrae* | 0.335474 | 0.942088 | 0.013482 | 0.091804 | Bacteria |
| 19 | *Aeribacillus pallidus* | 0.038773 | 0.076609 | 1.105494 | 0.116842 | Bacteria |
| 20 | *Parageobacillus toebii* | 0.045517 | 0.11802 | 1.031345 | 0.050075 | Bacteria |
| 21 | *Ureibacillus thermosphaericus* | 0.011801 | 0.095244 | 1.092012 | 0.041729 | Bacteria |
| 22 | *Sphingobacterium paucimobilis* | 0 | 0.004141 | 1.213347 | 0 | Bacteria |
| 23 | *Vagococcus fluvialis* | 0.787269 | 0 | 0 | 0.400601 | Bacteria |
| 24 | *Oligella ureolytica* | 0 | 0.262956 | 0.640377 | 0.267067 | Bacteria |
| 25 | *Castellaniella defragrans* | 0 | 0.302296 | 0.49208 | 0.325488 | Bacteria |
| 26 | *Bacillus fordii* | 0.062375 | 0.074539 | 0.653859 | 0.267067 | Bacteria |
| 27 | *Sphingobacterium nematocida* | 0 | 0 | 0.997641 | 0 | Bacteria |
| 28 | *Castellaniella caeni* | 0 | 0.333354 | 0.357263 | 0.300451 | Bacteria |
| 29 | *Escherichia coli* | 0 | 0.128372 | 0.424671 | 0.433984 | Bacteria |
| 30 | *Proteus mirabilis* | 0 | 0.010353 | 0.006741 | 0.901352 | Bacteria |
| 31 | *Bacillus* sp. OxB-1 | 0.006743 | 0.043481 | 0.566229 | 0.300451 | Bacteria |
| 32 | *Bacillus cereus* | 0.301758 | 0.213264 | 0.121335 | 0.191955 | Bacteria |
| 33 | *Oceanobacillus damuensis* | 0.025287 | 0.008282 | 0.020222 | 0.759473 | Bacteria |
| 34 | *Enterococcus faecalis* | 0.446737 | 0.219475 | 0.047186 | 0.075113 | Bacteria |
| 35 | *Bordetella petrii* | 0 | 0.227757 | 0.337041 | 0.216992 | Bacteria |
| 36 | *Geobacillus* sp. 8 | 0.131492 | 0.149078 | 0.431412 | 0.066767 | Bacteria |
| 37 | *Bacillus* phage PM1 | 0.566429 | 0.151148 | 0 | 0.041729 | Viruses |
| 38 | *Bacillus* sp. VT-16-64 | 0.057317 | 0.03934 | 0.337041 | 0.292105 | Bacteria |
| 39 | *Virgibacillus pantothenticus* | 0.050574 | 0.070398 | 0.323559 | 0.267067 | Bacteria |
| 40 | *Enterococcus faecium* | 0.094405 | 0.509348 | 0.006741 | 0.091804 | Bacteria |
| 41 | *Sphingobacterium wenxiniae* | 0 | 0 | 0.667341 | 0 | Bacteria |
| 42 | *Oceanobacillus manasiensis* | 0 | 0 | 0.040445 | 0.600901 | Bacteria |
| 43 | *Bordetella trematum* | 0 | 0.171853 | 0.296596 | 0.166917 | Bacteria |
| 44 | *Bacillus* phage phiNIT1 | 0.327045 | 0.19877 | 0 | 0.050075 | Viruses |
| 45 | *Bacillus methanolicus* | 0.025287 | 0.043481 | 0.397708 | 0.083459 | Bacteria |
| 46 | *Sporosarcina* sp. HYO08 | 0 | 0 | 0.289855 | 0.258721 | Bacteria |
| 47 | *Bacillus megaterium* | 0.033716 | 0.024846 | 0.195484 | 0.292105 | Bacteria |
| 48 | *Ornithinibacillus contaminans* | 0.008429 | 0 | 0.283114 | 0.208646 | Bacteria |
| 49 | *Sphingobacterium lactis* | 0 | 0.002071 | 0.471857 | 0 | Bacteria |
| 50 | *Orrella dioscoreae* | 0 | 0.151148 | 0.134816 | 0.183609 | Bacteria |
| 51 | *Bacillus alveayuensis* | 0.10452 | 0.033128 | 0.276373 | 0.050075 | Bacteria |
| 52 | *Advenella mimigardefordensis* | 0 | 0.126302 | 0.235929 | 0.10015 | Bacteria |
| 53 | *Sphingobacterium mizutaii* | 0 | 0.006212 | 0.444894 | 0.008346 | Bacteria |
| 54 | *Advenella kashmirensis* | 0 | 0.124231 | 0.134816 | 0.2003 | Bacteria |
| 55 | *Staphylococcus aureus* | 0.011801 | 0.095244 | 0.101112 | 0.250376 | Bacteria |
| 56 | *Gilliamella apicola* | 0.010115 | 0.033128 | 0.060667 | 0.350526 | Bacteria |
| 57 | *Sphingobacterium psychroaquaticum* | 0 | 0 | 0.444894 | 0.008346 | Bacteria |
| 58 | *Oceanobacillus* sp. Castelsardo | 0.015172 | 0.004141 | 0.013482 | 0.417293 | Bacteria |
| 59 | *Sphingobacterium* sp. CFCC 11742 | 0 | 0 | 0.444894 | 0 | Bacteria |
| 60 | *Oligella urethralis* | 0 | 0.051763 | 0.364004 | 0.025038 | Bacteria |
| 61 | *Listeria monocytogenes* | 0.155094 | 0.089032 | 0.053927 | 0.141879 | Bacteria |
| 62 | *Virgibacillus alimentarius* | 0.001686 | 0 | 0.074149 | 0.350526 | Bacteria |
| 63 | *Sporosarcina newyorkensis* | 0 | 0.016564 | 0.296596 | 0.108496 | Bacteria |
| 64 | *Bordetella* genomosp. 9 | 0 | 0.082821 | 0.229188 | 0.108496 | Bacteria |
| 65 | *Bacillus eiseniae* | 0.001686 | 0.004141 | 0.364004 | 0.041729 | Bacteria |
| 66 | *Sporosarcina psychrophila* | 0.001686 | 0 | 0.175261 | 0.233684 | Bacteria |
| 67 | *Virgibacillus proomii* | 0.006743 | 0.012423 | 0.101112 | 0.283759 | Bacteria |
| 68 | *Lentibacillus jeotgali* | 0.048888 | 0.028987 | 0.087631 | 0.233684 | Bacteria |
| 69 | *Oceanobacillus sojae* | 0.016858 | 0.012423 | 0.094371 | 0.275413 | Bacteria |
| 70 | *Bordetella ansorpii* | 0 | 0.091103 | 0.182002 | 0.125188 | Bacteria |
| 71 | *Oceanobacillus timonensis* | 0 | 0.014494 | 0.08089 | 0.300451 | Bacteria |
| 72 | *Pseudomonas aeruginosa* | 0.001686 | 0.082821 | 0.202224 | 0.108496 | Bacteria |
| 73 | *Sporosarcina ureae* | 0.001686 | 0.006212 | 0.141557 | 0.24203 | Bacteria |
| 74 | *Carnobacterium maltaromaticum* | 0.183752 | 0.074539 | 0.026963 | 0.10015 | Bacteria |
| 75 | *Virgibacillus soli* | 0 | 0 | 0.235929 | 0.141879 | Bacteria |
| 76 | *Sphingobacterium* sp. T2 | 0 | 0 | 0.350522 | 0.008346 | Bacteria |
| 77 | *Novibacillus thermophilus* | 0.026973 | 0.045551 | 0.235929 | 0.041729 | Bacteria |
| 78 | *Paucisalibacillus globulus* | 0.006743 | 0.004141 | 0.087631 | 0.250376 | Bacteria |
| 79 | *Bacillus amyloliquefaciens* | 0.097776 | 0.070398 | 0.020222 | 0.158571 | Bacteria |
| 80 | *Sphingobacterium spiritivorum* | 0 | 0.002071 | 0.343782 | 0 | Bacteria |
| 81 | *Oceanobacillus caeni* | 0.065746 | 0.024846 | 0.053927 | 0.2003 | Bacteria |
| 82 | *Sphingobacterium deserti* | 0 | 0 | 0.343782 | 0 | Bacteria |
| 83 | *Oceanobacillus picturae* | 0.026973 | 0 | 0.047186 | 0.267067 | Bacteria |
| 84 | *Ornithinibacillus halophilus* | 0.006743 | 0.010353 | 0.013482 | 0.308797 | Bacteria |
| 85 | *Oceanobacillus limi* | 0.003372 | 0.006212 | 0.033704 | 0.292105 | Bacteria |
| 86 | *Bordetella* genomosp. 13 | 0 | 0.060045 | 0.222447 | 0.050075 | Bacteria |
| 87 | *Bacillus* sp. X1(2014) | 0.075861 | 0.031058 | 0.202224 | 0.008346 | Bacteria |
| 88 | *Bacillus* phage vB_BanS-Tsamsa | 0.247813 | 0.026917 | 0 | 0.041729 | Viruses |
| 89 | *Oceanobacillus jeddahense* | 0.016858 | 0.026917 | 0.128075 | 0.141879 | Bacteria |
| 90 | *Bacillus* phage Grass | 0.173637 | 0.111808 | 0 | 0.025038 | Viruses |
| 91 | *Vaginella massiliensis* | 0 | 0 | 0.310078 | 0 | Bacteria |
| 92 | *Myroides odoratus* | 0 | 0 | 0.269633 | 0.033383 | Bacteria |
| 93 | *Virgibacillus halodenitrificans* | 0.03203 | 0.008282 | 0.053927 | 0.208646 | Bacteria |
| 94 | *Oceanobacillus oncorhynchi* | 0.008429 | 0.014494 | 0.020222 | 0.258721 | Bacteria |
| 95 | *Bacillus* phage SIOphi | 0.237698 | 0.060045 | 0 | 0 | Viruses |
| 96 | *Amphibacillus xylanus* | 0.003372 | 0.002071 | 0.121335 | 0.166917 | Bacteria |
| 97 | *Ornithinibacillus californiensis* | 0.001686 | 0.004141 | 0.026963 | 0.258721 | Bacteria |
| 98 | *Bacillus farraginis* | 0.187124 | 0.097315 | 0.006741 | 0 | Bacteria |
| 99 | *Bacillus sporothermodurans* | 0.067432 | 0.014494 | 0.175261 | 0.033383 | Bacteria |
| 100 | *Parageobacillus caldoxylosilyticus* | 0.015172 | 0.024846 | 0.074149 | 0.175263 | Bacteria |
| 101 | *Atopostipes suicloacalis* | 0.005057 | 0 | 0 | 0.283759 | Bacteria |
| 102 | *Paucisalibacillus* sp. EB02 | 0.013486 | 0.006212 | 0.033704 | 0.233684 | Bacteria |
| 103 | *Providencia stuartii* | 0 | 0.028987 | 0.074149 | 0.183609 | Bacteria |
| 104 | *Bacillus acidiproducens* | 0.121378 | 0.093173 | 0.013482 | 0.058421 | Bacteria |
| 105 | *Marinobacter* sp. T13-3 | 0 | 0.068327 | 0.114594 | 0.10015 | Bacteria |
| 106 | *Caryophanon latum* | 0.143293 | 0.107667 | 0.006741 | 0.025038 | Bacteria |
| 107 | *Bordetella* sp. N | 0 | 0.055904 | 0.134816 | 0.091804 | Bacteria |
| 108 | *Gracilibacillus boraciitolerans* | 0.003372 | 0 | 0.195484 | 0.083459 | Bacteria |
| 109 | *Parageobacillus thermantarcticus* | 0.021915 | 0.028987 | 0.222447 | 0.008346 | Bacteria |
| 110 | *Geobacillus* virus E3 | 0.038773 | 0 | 0.006741 | 0.233684 | Bacteria |
| 111 | *Virgibacillus siamensis* | 0 | 0.006212 | 0.047186 | 0.225338 | Bacteria |
| 112 | *Lysinibacillus massiliensis* | 0.021915 | 0.022776 | 0.155039 | 0.075113 | Bacteria |
| 113 | *Vitreoscilla* sp. SN6 | 0 | 0.031058 | 0.148298 | 0.091804 | Bacteria |
| 114 | *Pseudomonas fluorescens* | 0 | 0.010353 | 0.202224 | 0.058421 | Bacteria |
| 115 | *Brackiella oedipodis* | 0 | 0.113879 | 0.053927 | 0.10015 | Bacteria |
| 116 | *Myroides phaeus* | 0 | 0 | 0.262892 | 0 | Bacteria |
| 117 | *Vagococcus penaei* | 0.170266 | 0 | 0 | 0.091804 | Bacteria |
| 118 | *Gracilibacillus timonensis* | 0.016858 | 0.033128 | 0.026963 | 0.183609 | Bacteria |
| 119 | *Salmonella enterica* | 0 | 0.04141 | 0.107853 | 0.108496 | Bacteria |
| 120 | *Staphylococcus hominis* | 0.165208 | 0.07868 | 0.013482 | 0 | Bacteria |
| 121 | *Ornithinibacillus scapharcae* | 0.011801 | 0.004141 | 0.020222 | 0.216992 | Bacteria |
| 122 | *Bordetella holmesii* | 0 | 0.047622 | 0.16178 | 0.041729 | Bacteria |
| 123 | *Virgibacillus dokdonensis* | 0.005057 | 0.010353 | 0.026963 | 0.208646 | Bacteria |
| 124 | *Caldibacillus debilis* | 0.060689 | 0.031058 | 0.040445 | 0.116842 | Bacteria |
| 125 | *Myroides guanonis* | 0 | 0.002071 | 0.229188 | 0.016692 | Bacteria |
| 126 | *Anoxybacillus flavithermus* | 0.02023 | 0.020705 | 0.013482 | 0.191955 | Bacteria |
| 127 | *Caldalkalibacillus thermarum* | 0.006743 | 0.002071 | 0.208965 | 0.025038 | Bacteria |
| 128 | *Bacillus galactosidilyticus* | 0.003372 | 0.014494 | 0.155039 | 0.066767 | Bacteria |
| 129 | *Marinospirillum insulare* | 0 | 0.051763 | 0.175261 | 0.008346 | Bacteria |
| 130 | *Lentibacillus amyloliquefaciens* | 0.005057 | 0 | 0.087631 | 0.141879 | Bacteria |
| 131 | *Pseudopedobacter saltans* | 0 | 0.004141 | 0.229188 | 0 | Bacteria |
| 132 | *Bordetella avium* | 0 | 0.057975 | 0.114594 | 0.058421 | Bacteria |
| 133 | *Gulbenkiania indica* | 0 | 0.018635 | 0.16178 | 0.050075 | Bacteria |
| 134 | *Bacillus niameyensis* | 0.008429 | 0.033128 | 0.020222 | 0.166917 | Bacteria |
| 135 | *Leclercia* sp. LK8 | 0 | 0.006212 | 0.222447 | 0 | Bacteria |
| 136 | *Lentibacillus halodurans* | 0.001686 | 0 | 0 | 0.225338 | Bacteria |
| 137 | *Bacillus halodurans* | 0.175323 | 0.002071 | 0.006741 | 0.041729 | Bacteria |
| 138 | *Paenibacillus* sp. P22 | 0.015172 | 0.04141 | 0.026963 | 0.141879 | Bacteria |
| 139 | *Parapedobacter composti* | 0 | 0 | 0.222447 | 0 | Bacteria |
| 140 | *Vibrio cholerae* | 0 | 0.026917 | 0.013482 | 0.175263 | Bacteria |
| 141 | *Tepidimicrobium xylanilyticum* | 0.003372 | 0 | 0.202224 | 0.008346 | Bacteria |
| 142 | *Salinibacillus kushneri* | 0.001686 | 0 | 0.020222 | 0.191955 | Bacteria |
| 143 | *Sphingobacterium* sp. JB170 | 0 | 0.002071 | 0.202224 | 0.008346 | Bacteria |
| 144 | *Bacillus lentus* | 0.001686 | 0.004141 | 0.067408 | 0.133534 | Bacteria |
| 145 | *Bacillus cohnii* | 0.016858 | 0 | 0.067408 | 0.116842 | Bacteria |
| 146 | *Acinetobacter baumannii* | 0 | 0.04141 | 0.08089 | 0.075113 | Bacteria |
| 147 | *Empedobacter falsenii* | 0 | 0.006212 | 0.188743 | 0 | Bacteria |
| 148 | *Bordetella* sp. FB-8 | 0 | 0.051763 | 0.067408 | 0.075113 | Bacteria |
| 149 | *Geobacillus* sp. WCH70 | 0 | 0.020705 | 0.16178 | 0.008346 | Bacteria |
| 150 | *Globicatella sulfidifaciens* | 0.003372 | 0.002071 | 0.101112 | 0.083459 | Bacteria |
| 151 | *Bacillus pseudofirmus* | 0.072489 | 0.018635 | 0.040445 | 0.058421 | Bacteria |
| 152 | *Achromobacter* sp. DMS1 | 0 | 0.049693 | 0.08089 | 0.058421 | Bacteria |
| 153 | Paraliobacillus ryukyuensis | 0.003372 | 0.010353 | 0.040445 | 0.133534 | Bacteria |
| 154 | *Oceanobacillus iheyensis* | 0.005057 | 0.006212 | 0 | 0.175263 | Bacteria |
| 155 | *Pseudomonas brassicacearum* | 0 | 0.028987 | 0.148298 | 0.008346 | Bacteria |
| 156 | *Heliobacterium modesticaldum* | 0.006743 | 0.04141 | 0.026963 | 0.108496 | Bacteria |
| 157 | *Parageobacillus thermoglucosidans* | 0.035402 | 0.043481 | 0.087631 | 0.016692 | Bacteria |
| 158 | *Anoxybacillus* sp. UARK-01 | 0.030344 | 0.028987 | 0.114594 | 0.008346 | Bacteria |
| 159 | *Psychrobacillus* sp. OK032 | 0.008429 | 0.002071 | 0.053927 | 0.116842 | Bacteria |
| 160 | Sphingobacterium thalpophilum | 0 | 0.002071 | 0.175261 | 0 | Bacteria |
| 161 | Basilea psittacipulmonis | 0 | 0.047622 | 0.060667 | 0.066767 | Bacteria |
| 162 | Pasteurella testudinis | 0 | 0.002071 | 0.155039 | 0.016692 | Bacteria |
| 163 | *Virgibacillus* sp. SK37 | 0.003372 | 0 | 0.026963 | 0.141879 | Bacteria |
| 164 | *Pseudomonas linyingensis* | 0 | 0.062116 | 0.040445 | 0.066767 | Bacteria |
| 165 | *Myroides injenensis* | 0 | 0 | 0.16852 | 0 | Bacteria |
| 166 | *Lentibacillus persicus* | 0 | 0.004141 | 0.047186 | 0.116842 | Bacteria |
| 167 | *Bacillus shackletonii* | 0.026973 | 0.051763 | 0.020222 | 0.066767 | Bacteria |
| 168 | *Pontibacillus litoralis* | 0.001686 | 0.024846 | 0.020222 | 0.116842 | Bacteria |
| 169 | *Natribacillus halophilus* | 0 | 0.016564 | 0.087631 | 0.058421 | Bacteria |
| 170 | *Marinobacterium stanieri* | 0 | 0.047622 | 0.060667 | 0.050075 | Bacteria |
| 171 | *Gracilibacillus halophilus* | 0.001686 | 0.004141 | 0.026963 | 0.125188 | Bacteria |
| 172 | *Bacillus* sp. B14905 | 0.008429 | 0.060045 | 0.013482 | 0.075113 | Bacteria |
| 173 | *Bacillus stratosphericus* | 0 | 0 | 0.08089 | 0.075113 | Bacteria |
| 174 | *Bhargavaea cecembensis* | 0.015172 | 0.004141 | 0.094371 | 0.041729 | Bacteria |
| 175 | *Bordetella* sp. H567 | 0 | 0.053834 | 0.067408 | 0.033383 | Bacteria |
| 176 | *Gluconobacter frateurii* | 0.001686 | 0.022776 | 0.087631 | 0.041729 | Bacteria |
| 177 | *Sporosarcina* sp. ZBG7A | 0 | 0 | 0.094371 | 0.058421 | Bacteria |
| 178 | *Xanthomonas citri* | 0.001686 | 0.028987 | 0.013482 | 0.108496 | Bacteria |
| 179 | *Shigella flexneri* | 0 | 0 | 0.134816 | 0.016692 | Bacteria |
| 180 | *Bacillus timonensis* | 0.006743 | 0.002071 | 0.033704 | 0.108496 | Bacteria |
| 181 | *Flavobacterium psychrophilum* | 0 | 0 | 0.141557 | 0.008346 | Bacteria |
| 182 | *Bordetella flabilis* | 0 | 0.057975 | 0.047186 | 0.041729 | Bacteria |
| 183 | *Paenibacillus* sp. oral taxon 786 | 0.040459 | 0.089032 | 0 | 0.016692 | Bacteria |
| 184 | *Pectobacterium carotovorum* | 0 | 0.018635 | 0.107853 | 0.016692 | Bacteria |
| 185 | *Klebsiella pneumoniae* | 0.001686 | 0.014494 | 0.067408 | 0.058421 | Bacteria |
| 186 | *Bacillus pumilus* | 0.023601 | 0.010353 | 0.074149 | 0.033383 | Bacteria |
| 187 | *Lampropedia hyalina* | 0 | 0.020705 | 0.094371 | 0.025038 | Bacteria |
| 188 | *Batrachochytrium dendrobatidis* | 0.005057 | 0 | 0 | 0.133534 | Eukaryota |
| 189 | *Providencia rettgeri* | 0 | 0.022776 | 0.040445 | 0.075113 | Bacteria |
| 190 | *Bacillus* sp. OK048 | 0.018544 | 0.012423 | 0.047186 | 0.058421 | Bacteria |
| 191 | *Bacillus gottheilii* | 0.006743 | 0.002071 | 0.026963 | 0.10015 | Bacteria |
| 192 | *Geobacillus kaustophilus* | 0.038773 | 0.014494 | 0.074149 | 0.008346 | Bacteria |
| 193 | *Halotalea alkalilenta* | 0 | 0.004141 | 0.08089 | 0.050075 | Bacteria |
| 194 | *Anaerobacillus alkalilacustris* | 0.016858 | 0.031058 | 0.020222 | 0.066767 | Bacteria |
| 195 | *Acidovorax* sp. MR-S7 | 0 | 0.018635 | 0.107853 | 0.008346 | Bacteria |
| 196 | *Anaerobacillus* sp. NB2006 | 0.026973 | 0.033128 | 0.040445 | 0.033383 | Bacteria |
| 197 | *Gilliamella intestini* | 0 | 0 | 0 | 0.133534 | Bacteria |
| 198 | *Pseudomonas saponiphila* | 0 | 0.018635 | 0.114594 | 0 | Bacteria |
| 199 | *Bacillus azotoformans* | 0.008429 | 0.033128 | 0.040445 | 0.050075 | Bacteria |
| 200 | *Globicatella* sp. HMSC072A10 | 0 | 0 | 0.114594 | 0.016692 | Bacteria |
| 201 | *Alkalibacillus haloalkaliphilus* | 0.001686 | 0.002071 | 0.060667 | 0.066767 | Bacteria |
| 202 | *Sporosarcina* sp. D27 | 0 | 0.008282 | 0.08089 | 0.041729 | Bacteria |
| 203 | *Bacillus gobiensis* | 0.030344 | 0 | 0 | 0.10015 | Bacteria |
| 204 | *Geobacillus stearothermophilus* | 0.010115 | 0.047622 | 0.047186 | 0.025038 | Bacteria |
| 205 | *Globicatella sanguinis* | 0 | 0.002071 | 0.094371 | 0.033383 | Bacteria |
| 206 | *Streptococcus pneumoniae* | 0.037088 | 0.024846 | 0 | 0.066767 | Bacteria |
| 207 | *Myroides odoratimimus* | 0 | 0 | 0.128075 | 0 | Bacteria |
| 208 | *Sphingobacterium* sp. ML3W | 0 | 0 | 0.128075 | 0 | Bacteria |
| 209 | *Bacillus shacheensis* | 0.011801 | 0.010353 | 0.047186 | 0.058421 | Bacteria |
| 210 | *Bacillus badius* | 0.003372 | 0.020705 | 0.020222 | 0.083459 | Bacteria |
| 211 | *Bacillus* sp. FJAT-29814 | 0.026973 | 0.045551 | 0.013482 | 0.041729 | Bacteria |
| 212 | *Pseudomonas syringae* | 0 | 0.004141 | 0.006741 | 0.116842 | Bacteria |
| 213 | *Pseudomonas knackmussii* | 0 | 0.018635 | 0.107853 | 0 | Bacteria |
| 214 | *Azotobacter beijerinckii* | 0 | 0.014494 | 0.060667 | 0.050075 | Bacteria |
| 215 | *Bacillus rubiinfantis* | 0.042145 | 0.020705 | 0.020222 | 0.041729 | Bacteria |
| 216 | *Bacillus sonorensis* | 0.057317 | 0.037269 | 0.013482 | 0.016692 | Bacteria |
| 217 | *Paramesorhizobium deserti* | 0 | 0 | 0.107853 | 0.016692 | Bacteria |
| 218 | *Terribacillus halophilus* | 0.013486 | 0.018635 | 0.033704 | 0.058421 | Bacteria |
| 219 | *Bacillus* sp. TH008 | 0.038773 | 0.084891 | 0 | 0 | Bacteria |
| 220 | *Mycobacterium abscessus* | 0.011801 | 0.037269 | 0.006741 | 0.066767 | Bacteria |
| 221 | *Clostridium botulinum* | 0.026973 | 0.020705 | 0.013482 | 0.058421 | Bacteria |
| 222 | *Edaphobacillus lindanitolerans* | 0.011801 | 0.008282 | 0.040445 | 0.058421 | Bacteria |
| 223 | *Stenotrophomonas maltophilia* | 0 | 0.033128 | 0.060667 | 0.025038 | Bacteria |
| 224 | *Aeribacillus* phage AP45 | 0 | 0.004141 | 0.114594 | 0 | Viruses |
| 225 | *Bacillus thuringiensis* | 0.023601 | 0.037269 | 0.006741 | 0.050075 | Bacteria |
| 226 | *Streptococcus agalactiae* | 0.016858 | 0.016564 | 0 | 0.083459 | Bacteria |
| 227 | *Lysinibacillus sinduriensis* | 0.018544 | 0.006212 | 0.033704 | 0.058421 | Bacteria |
| 228 | *Weeksella* sp. FF8 | 0 | 0 | 0.107853 | 0.008346 | Bacteria |
| 229 | *Achromobacter piechaudii* | 0 | 0.045551 | 0.053927 | 0.016692 | Bacteria |
| 230 | *Acinetobacter* sp. MDS7A | 0 | 0.008282 | 0.107853 | 0 | Bacteria |
| 231 | *Amphibacillus sediminis* | 0.023601 | 0.010353 | 0.006741 | 0.075113 | Bacteria |
| 232 | *Cosenzaea myxofaciens* | 0 | 0 | 0.040445 | 0.075113 | Bacteria |
| 233 | *Flavobacterium columnare* | 0.003372 | 0.004141 | 0.107853 | 0 | Bacteria |
| 234 | *Bacillus aquimaris* | 0.018544 | 0.033128 | 0.013482 | 0.050075 | Bacteria |
| 235 | *Enterococcus pallens* | 0.072489 | 0.002071 | 0.006741 | 0.033383 | Bacteria |
| 236 | *Chelonobacter oris* | 0 | 0 | 0.114594 | 0 | Bacteria |
| 237 | *Lautropia mirabilis* | 0 | 0.037269 | 0.026963 | 0.050075 | Bacteria |
| 238 | *Thermoactinomyces* sp. DSM 45892 | 0.062375 | 0.016564 | 0.026963 | 0.008346 | Bacteria |
| 239 | *Bacillus tuaregi* | 0.038773 | 0.016564 | 0 | 0.058421 | Bacteria |
| 240 | *Bacillus caseinilyticus* | 0.015172 | 0.016564 | 0.006741 | 0.075113 | Bacteria |
| 241 | *Gracilibacillus ureilyticus* | 0.001686 | 0.012423 | 0.006741 | 0.091804 | Bacteria |
| 242 | *Empedobacter brevis* | 0 | 0.002071 | 0.101112 | 0.008346 | Bacteria |
| 243 | *Paenisporosarcina* sp. TG20 | 0.003372 | 0.016564 | 0.074149 | 0.016692 | Bacteria |
| 244 | *Tuberibacillus calidus* | 0.003372 | 0 | 0.040445 | 0.066767 | Bacteria |
| 245 | *Virgibacillus chiguensis* | 0 | 0 | 0.026963 | 0.083459 | Bacteria |
| 246 | *Bacillus dakarensis* | 0 | 0.006212 | 0.020222 | 0.083459 | Bacteria |
| 247 | *Enterococcus phoeniculicola* | 0.075861 | 0.002071 | 0.006741 | 0.025038 | Bacteria |
| 248 | *Geobacillus* sp. 12AMOR1 | 0.015172 | 0 | 0.094371 | 0 | Bacteria |
| 249 | *Comamonas composti* | 0 | 0.010353 | 0.074149 | 0.025038 | Bacteria |
| 250 | *Ignatzschineria indica* | 0 | 0.010353 | 0.040445 | 0.058421 | Bacteria |
| 251 | *Streptomyces rubidus* | 0.023601 | 0.024846 | 0.060667 | 0 | Bacteria |
| 252 | *Pelistega* sp. MC2 | 0 | 0.04141 | 0.067408 | 0 | Bacteria |
| 253 | *Amphibacillus jilinensis* | 0 | 0.008282 | 0.033704 | 0.066767 | Bacteria |
| 254 | *Streptococcus anginosus* | 0.079233 | 0.022776 | 0.006741 | 0 | Bacteria |
| 255 | *Piscibacillus halophilus* | 0 | 0.008282 | 0 | 0.10015 | Bacteria |
| 256 | *Aquibacillus* sp. Marseille-P3518 | 0.026973 | 0.012423 | 0.026963 | 0.041729 | Bacteria |
| 257 | *Bacillus plakortidis* | 0 | 0 | 0.107853 | 0 | Bacteria |
| 258 | *Flavobacterium fontis* | 0 | 0 | 0.107853 | 0 | Bacteria |
| 259 | *Bacillus cellulosilyticus* | 0.011801 | 0.004141 | 0 | 0.091804 | Bacteria |
| 260 | *Bacteroides fragilis* | 0.015172 | 0.018635 | 0.006741 | 0.066767 | Bacteria |
| 261 | *Bacillus niacini* | 0.001686 | 0.006212 | 0.006741 | 0.091804 | Bacteria |
| 262 | *Anoxybacillus* sp. BCO1 | 0.02023 | 0.018635 | 0.033704 | 0.033383 | Bacteria |
| 263 | *Marinobacter lutaoensis* | 0 | 0 | 0.08089 | 0.025038 | Bacteria |
| 264 | *Bacillus ginsengihumi* | 0.005057 | 0.006212 | 0.026963 | 0.066767 | Bacteria |
| 265 | *Sediminibacillus halophilus* | 0.001686 | 0.014494 | 0.013482 | 0.075113 | Bacteria |
| 266 | *Bacillus flexus* | 0.011801 | 0.002071 | 0.074149 | 0.016692 | Bacteria |
| 267 | *Aquaspirillum* sp. LM1 | 0 | 0.006212 | 0.08089 | 0.016692 | Bacteria |
| 268 | *Taylorella asinigenitalis* | 0 | 0.047622 | 0.013482 | 0.041729 | Bacteria |
| 269 | *Halobacillus hunanensis* | 0.006743 | 0.004141 | 0 | 0.091804 | Bacteria |
| 270 | *Bacillus circulans* | 0.016858 | 0 | 0.060667 | 0.025038 | Bacteria |
| 271 | *Enterococcus durans* | 0.005057 | 0.053834 | 0.033704 | 0.008346 | Bacteria |
| 272 | *Bacillus solani* | 0.005057 | 0.006212 | 0.08089 | 0.008346 | Bacteria |
| 273 | *Pseudomonas putida* | 0 | 0.024846 | 0.033704 | 0.041729 | Bacteria |
| 274 | *Fluviicola taffensis* | 0 | 0.012423 | 0.087631 | 0 | Bacteria |
| 275 | *Zymomonas mobilis* | 0 | 0.012423 | 0.053927 | 0.033383 | Bacteria |
| 276 | *Planococcus maritimus* | 0 | 0.002071 | 0.013482 | 0.083459 | Bacteria |
| 277 | *Flavobacterium soli* | 0 | 0.004141 | 0.094371 | 0 | Bacteria |
| 278 | *Flavobacterium suncheonense* | 0 | 0.004141 | 0.094371 | 0 | Bacteria |
| 279 | *Jeotgalibaca dankookensis* | 0.045517 | 0.004141 | 0.006741 | 0.041729 | Bacteria |
| 280 | *Comamonas testosteroni* | 0 | 0.002071 | 0.087631 | 0.008346 | Bacteria |
| 281 | *Providencia alcalifaciens* | 0 | 0 | 0.013482 | 0.083459 | Bacteria |
| 282 | *Domibacillus enclensis* | 0.008429 | 0.002071 | 0.060667 | 0.025038 | Bacteria |
| 283 | *Kurthia* sp. 11kri321 | 0.003372 | 0.004141 | 0.013482 | 0.075113 | Bacteria |
| 284 | *Bacillus altitudinis* | 0.042145 | 0.053834 | 0 | 0 | Bacteria |
| 285 | *Caryophanon tenue* | 0.006743 | 0 | 0.047186 | 0.041729 | Bacteria |
| 286 | *Bacillus ligniniphilus* | 0.011801 | 0.018635 | 0.006741 | 0.058421 | Bacteria |
| 287 | *Pelagirhabdus alkalitolerans* | 0.001686 | 0 | 0.026963 | 0.066767 | Bacteria |
| 288 | *Burkholderia pseudomallei* | 0 | 0.016564 | 0.020222 | 0.058421 | Bacteria |
| 289 | *Bacillus* sp. FJAT-27445 | 0.006743 | 0.004141 | 0.067408 | 0.016692 | Bacteria |
| 290 | *Bacillus bogoriensis* | 0.005057 | 0.004141 | 0.026963 | 0.058421 | Bacteria |
| 291 | *Flavobacterium filum* | 0 | 0 | 0.094371 | 0 | Bacteria |
| 292 | *Vagococcus* sp. D7T301 | 0.069118 | 0 | 0 | 0.025038 | Bacteria |
| 293 | *Bacillus* sp. Marseille-P2366 | 0.043831 | 0.016564 | 0 | 0.033383 | Bacteria |
| 294 | *Paenisporosarcina* sp. TG-14 | 0 | 0 | 0.026963 | 0.066767 | Bacteria |
| 295 | *Bacillus* sp. MB2021 | 0.028659 | 0.022776 | 0.033704 | 0.008346 | Bacteria |
| 296 | *Bacillus* sp. FJAT-27225 | 0.006743 | 0.010353 | 0 | 0.075113 | Bacteria |
| 297 | *Bacillus* sp. MKU004 | 0.06406 | 0.014494 | 0.013482 | 0 | Bacteria |
| 298 | *Neisseria shayeganii* | 0.006743 | 0.014494 | 0.053927 | 0.016692 | Bacteria |
| 299 | *Pedobacter glucosidilyticus* | 0 | 0.004141 | 0.087631 | 0 | Bacteria |
| 300 | *Pontibacillus yanchengensis* | 0.003372 | 0.014494 | 0.040445 | 0.033383 | Bacteria |
| 301 | *Bordetella pertussis* | 0 | 0.04141 | 0 | 0.050075 | Bacteria |
| 302 | *Bacillus marisflavi* | 0.018544 | 0.004141 | 0.026963 | 0.041729 | Bacteria |
| 303 | *Alicyclobacillus macrosporangiidus* | 0 | 0.008282 | 0.074149 | 0.008346 | Bacteria |
| 304 | *Bordetella bronchialis* | 0 | 0.024846 | 0.040445 | 0.025038 | Bacteria |
| 305 | *Vitreoscilla stercoraria* | 0.001686 | 0.002071 | 0.026963 | 0.058421 | Bacteria |
| 306 | *Gracilibacillus lacisalsi* | 0 | 0 | 0.047186 | 0.041729 | Bacteria |
| 307 | *Sphingobacterium* sp. HMSC13C05 | 0 | 0 | 0.047186 | 0.041729 | Bacteria |
| 308 | *Paraliobacillus* sp. PM-2 | 0.030344 | 0 | 0 | 0.058421 | Bacteria |
| 309 | *Isobaculum melis* | 0.021915 | 0 | 0 | 0.066767 | Bacteria |
| 310 | *Sediminibacillus albus* | 0 | 0 | 0.013482 | 0.075113 | Bacteria |
| 311 | *Azotobacter vinelandii* | 0 | 0.016564 | 0.013482 | 0.058421 | Bacteria |
| 312 | *Pontibacillus halophilus* | 0.005057 | 0.024846 | 0 | 0.058421 | Bacteria |
| 313 | *Flavobacterium flevense* | 0 | 0.012423 | 0.067408 | 0.008346 | Bacteria |
| 314 | *Olivibacter sitiensis* | 0 | 0 | 0.087631 | 0 | Bacteria |
| 315 | *Pedobacter soli* | 0 | 0 | 0.087631 | 0 | Bacteria |
| 316 | *Bacillus* phage Mgbh1 | 0 | 0 | 0.087631 | 0 | Viruses |
| 317 | *Tenuibacillus multivorans* | 0.006743 | 0.002071 | 0.020222 | 0.058421 | Bacteria |
| 318 | *Enterococcus saccharolyticus* | 0.025287 | 0 | 0.020222 | 0.041729 | Bacteria |
| 319 | *Bacillus mojavensis* | 0.048888 | 0.006212 | 0.006741 | 0.025038 | Bacteria |
| 320 | *Fusicatenibacter saccharivorans* | 0 | 0.012423 | 0.074149 | 0 | Bacteria |
| 321 | *Virgibacillus senegalensis* | 0.02023 | 0 | 0.006741 | 0.058421 | Bacteria |
| 322 | *Marinobacter* sp. X15-166B | 0 | 0 | 0.026963 | 0.058421 | Bacteria |
| 323 | *Paucibacter* sp. KCTC 42545 | 0 | 0.008282 | 0.026963 | 0.050075 | Bacteria |
| 324 | *Lysinibacillus xylanilyticus* | 0.008429 | 0.002071 | 0.040445 | 0.033383 | Bacteria |
| 325 | *Pseudomonas caeni* | 0 | 0.012423 | 0.013482 | 0.058421 | Bacteria |
| 326 | *Planococcus massiliensis* | 0 | 0.010353 | 0.006741 | 0.066767 | Bacteria |
| 327 | *Bordetella hinzii* | 0 | 0.012423 | 0.053927 | 0.016692 | Bacteria |
| 328 | *Acinetobacter haemolyticus* | 0 | 0.002071 | 0.08089 | 0 | Bacteria |
| 329 | *Alteribacillus bidgolensis* | 0 | 0.002071 | 0.047186 | 0.033383 | Bacteria |
| 330 | *Amphibacillus marinus* | 0 | 0.002071 | 0.013482 | 0.066767 | Bacteria |
| 331 | *Ralstonia solanacearum* | 0 | 0.024846 | 0.040445 | 0.016692 | Bacteria |
| 332 | *Listeria grayi* | 0.010115 | 0.033128 | 0.013482 | 0.025038 | Bacteria |
| 333 | *Bacillus massiliosenegalensis* | 0.015172 | 0.031058 | 0.026963 | 0.008346 | Bacteria |
| 334 | *Bacillus clausii* | 0.021915 | 0.012423 | 0.013482 | 0.033383 | Bacteria |
| 335 | *Bacillus fastidiosus* | 0.013486 | 0.004141 | 0.013482 | 0.050075 | Bacteria |
| 336 | *Parapedobacter indicus* | 0 | 0 | 0.08089 | 0 | Bacteria |
| 337 | *Fictibacillus gelatini* | 0.003372 | 0.002071 | 0.033704 | 0.041729 | Bacteria |
| 338 | *Bacillus mesonae* | 0.013486 | 0.008282 | 0.033704 | 0.025038 | Bacteria |
| 339 | *Halomonas gudaonensis* | 0 | 0.018635 | 0.060667 | 0 | Bacteria |
| 340 | *Bacillus salsus* | 0.013486 | 0.002071 | 0.013482 | 0.050075 | Bacteria |
| 341 | *Providencia heimbachae* | 0 | 0.002071 | 0.026963 | 0.050075 | Bacteria |
| 342 | *Bacillus pseudalcaliphilus* | 0 | 0.010353 | 0.026963 | 0.041729 | Bacteria |
| 343 | *Lampropedia cohaerens* | 0 | 0.026917 | 0.026963 | 0.025038 | Bacteria |
| 344 | *Halomonas xianhensis* | 0 | 0.035199 | 0.026963 | 0.016692 | Bacteria |
| 345 | *Bacillus bataviensis* | 0.016858 | 0.006212 | 0.047186 | 0.008346 | Bacteria |
| 346 | *Snodgrassella* sp. CFCC 13594 | 0 | 0.016564 | 0.020222 | 0.041729 | Bacteria |
| 347 | *Serratia marcescens* | 0 | 0.006212 | 0.013482 | 0.058421 | Bacteria |
| 348 | *Lysinibacillus manganicus* | 0.001686 | 0.004141 | 0.047186 | 0.025038 | Bacteria |
| 349 | *Bacillus firmus* | 0.015172 | 0.012423 | 0.033704 | 0.016692 | Bacteria |
| 350 | *Bacillus halmapalus* | 0 | 0.004141 | 0.006741 | 0.066767 | Bacteria |
| 351 | *Bacillus okhensis* | 0.003372 | 0.002071 | 0.013482 | 0.058421 | Bacteria |
| 352 | *Bacillus paralicheniformis* | 0.053946 | 0.016564 | 0.006741 | 0 | Bacteria |
| 353 | *Pseudomonas stutzeri* | 0 | 0.012423 | 0.013482 | 0.050075 | Bacteria |
| 354 | *Clostridium* sp. N3C | 0.057317 | 0.018635 | 0 | 0 | Bacteria |
| 355 | *Lysinibacillus contaminans* | 0 | 0.002071 | 0.040445 | 0.033383 | Bacteria |
| 356 | *Parasutterella excrementihominis* | 0 | 0.016564 | 0.033704 | 0.025038 | Bacteria |
| 357 | *Bacillus* sp. SJS | 0.001686 | 0 | 0.006741 | 0.066767 | Bacteria |
| 358 | *Burkholderia mallei* | 0 | 0.033128 | 0.033704 | 0.008346 | Bacteria |
| 359 | *Salipaludibacillus aurantiacus* | 0 | 0.006212 | 0.026963 | 0.041729 | Bacteria |
| 360 | *Tepidibacillus decaturensis* | 0 | 0.010353 | 0.047186 | 0.016692 | Bacteria |
| 361 | *Chryseobacterium* sp. FH1 | 0 | 0 | 0.074149 | 0 | Bacteria |
| 362 | *Flavobacterium* sp. 316 | 0 | 0 | 0.074149 | 0 | Bacteria |
| 363 | *Parapedobacter koreensis* | 0 | 0 | 0.074149 | 0 | Bacteria |
| 364 | *Bacillus fumarioli* | 0.018544 | 0.026917 | 0.020222 | 0.008346 | Bacteria |
| 365 | *Pasteurella multocida* | 0.003372 | 0.008282 | 0.053927 | 0.008346 | Bacteria |
| 366 | *Acinetobacter brisouii* | 0 | 0.006212 | 0.067408 | 0 | Bacteria |
| 367 | *Chryseobacterium* sp. Hurlbut01 | 0 | 0.006212 | 0.067408 | 0 | Bacteria |
| 368 | *Bacillus koreensis* | 0.015172 | 0.008282 | 0 | 0.050075 | Bacteria |
| 369 | *Alteribacillus iranensis* | 0 | 0 | 0.006741 | 0.066767 | Bacteria |
| 370 | *Anoxybacillus tepidamans* | 0 | 0 | 0.006741 | 0.066767 | Bacteria |
| 371 | *Massilibacterium senegalense* | 0.008429 | 0.006212 | 0 | 0.058421 | Bacteria |
| 372 | *Thalassobacillus devorans* | 0.001686 | 0.004141 | 0 | 0.066767 | Bacteria |
| 373 | *Bacillus massiliogorillae* | 0.006743 | 0.002071 | 0.013482 | 0.050075 | Bacteria |
| 374 | *Kurthia senegalensis* | 0 | 0.002071 | 0.020222 | 0.050075 | Bacteria |
| 375 | *Taylorella equigenitalis* | 0 | 0.018635 | 0.020222 | 0.033383 | Bacteria |
| 376 | *Bacillus simplex* | 0.006743 | 0.006212 | 0 | 0.058421 | Bacteria |
| 377 | *Lysinibacillus odysseyi* | 0.016858 | 0.004141 | 0 | 0.050075 | Bacteria |
| 378 | *Bacillus* sp. FJAT-14578 | 0 | 0.004141 | 0 | 0.066767 | Bacteria |
| 379 | *Carnobacterium viridans* | 0.062375 | 0 | 0 | 0.008346 | Bacteria |
| 380 | *Achromobacter* sp. RTa | 0 | 0.006212 | 0.013482 | 0.050075 | Bacteria |
| 381 | *Burkholderia ubonensis* | 0 | 0.004141 | 0.040445 | 0.025038 | Bacteria |
| 382 | *Marinomonas spartinae* | 0 | 0.012423 | 0.040445 | 0.016692 | Bacteria |
| 383 | *Bacillus oceanisediminis* | 0.011801 | 0.004141 | 0.020222 | 0.033383 | Bacteria |
| 384 | *Achromobacter* sp. DH1f | 0 | 0.020705 | 0.040445 | 0.008346 | Bacteria |
| 385 | *Bacillus alcalophilus* | 0.010115 | 0.004141 | 0.013482 | 0.041729 | Bacteria |
| 386 | *Bacillus krulwichiae* | 0.010115 | 0.002071 | 0.040445 | 0.016692 | Bacteria |
| 387 | *Oleispira antarctica* | 0.001686 | 0.028987 | 0.013482 | 0.025038 | Bacteria |
| 388 | *Staphylococcus pseudintermedius* | 0 | 0 | 0.060667 | 0.008346 | Bacteria |
| 389 | *Bacillus endophyticus* | 0.001686 | 0.002071 | 0.006741 | 0.058421 | Bacteria |
| 390 | *Proteus hauseri* | 0 | 0.002071 | 0 | 0.066767 | Bacteria |
| 391 | *Achromobacter xylosoxidans* | 0 | 0.060045 | 0 | 0.008346 | Bacteria |
| 392 | *Bacillus persicus* | 0.001686 | 0.014494 | 0.026963 | 0.025038 | Bacteria |
| 393 | *Marinilactibacillus piezotolerans* | 0.021915 | 0.012423 | 0 | 0.033383 | Bacteria |
| 394 | *Flavobacterium antarcticum* | 0 | 0 | 0.067408 | 0 | Bacteria |
| 395 | *Flavobacterium beibuense* | 0 | 0 | 0.067408 | 0 | Bacteria |
| 396 | *Flavobacterium daejeonense* | 0 | 0 | 0.067408 | 0 | Bacteria |
| 397 | *Flavobacterium enshiense* | 0 | 0 | 0.067408 | 0 | Bacteria |
| 398 | *Sphingobacterium faecium* | 0 | 0 | 0.067408 | 0 | Bacteria |
| 399 | *Sphingobacterium* sp. IITKGP-BTPF85 | 0 | 0 | 0.067408 | 0 | Bacteria |
| 400 | *Bacillus wakoensis* | 0.003372 | 0.010353 | 0.020222 | 0.033383 | Bacteria |
| 401 | *Carnobacterium* sp. CP1 | 0.050574 | 0 | 0 | 0.016692 | Bacteria |
| 402 | *Cupriavidus necator* | 0 | 0.016564 | 0.033704 | 0.016692 | Bacteria |
| 403 | *Ralstonia* sp. PBA | 0 | 0.016564 | 0.033704 | 0.016692 | Bacteria |
| 404 | *Bacillus* sp. Marseille-P2384 | 0.025287 | 0.016564 | 0 | 0.025038 | Bacteria |
| 405 | *Achromobacter xylosoxidans* | 0 | 0 | 0 | 0.066767 | Bacteria |
| 406 | *Enteractinococcus helveticum* | 0 | 0 | 0 | 0.066767 | Bacteria |
| 407 | *Marinobacter* sp. EN3 | 0.001686 | 0.014494 | 0.033704 | 0.016692 | Bacteria |
| 408 | *Bacillus* sp. NC2-31 | 0.021915 | 0.004141 | 0.006741 | 0.033383 | Bacteria |
| 409 | *Methylobacillus* sp. MM2 | 0 | 0.004141 | 0.020222 | 0.041729 | Bacteria |
| 410 | *Enterococcus gallinarum* | 0.055631 | 0.002071 | 0 | 0.008346 | Bacteria |
| 411 | *Bacillus* sp. LL01 | 0.001686 | 0.002071 | 0.053927 | 0.008346 | Bacteria |
| 412 | *Janthinobacterium* sp. Marseille | 0 | 0.018635 | 0.047186 | 0 | Bacteria |
| 413 | *Aneurinibacillus* sp. XH2 | 0.023601 | 0 | 0 | 0.041729 | Bacteria |
| 414 | *Paenibacillus macerans* | 0.02023 | 0.012423 | 0.006741 | 0.025038 | Bacteria |
| 415 | *Mannheimia haemolytica* | 0 | 0.002071 | 0.020222 | 0.041729 | Bacteria |
| 416 | *Pilibacter termitis* | 0.048888 | 0 | 0.006741 | 0.008346 | Bacteria |
| 417 | *Enterococcus casseliflavus* | 0.047202 | 0 | 0 | 0.016692 | Bacteria |
| 418 | *Paracoccus chinensis* | 0 | 0.016564 | 0.047186 | 0 | Bacteria |
| 419 | *Fictibacillus arsenicus* | 0 | 0 | 0.013482 | 0.050075 | Bacteria |
| 420 | *Pseudomonas thermotolerans* | 0 | 0.016564 | 0.013482 | 0.033383 | Bacteria |
| 421 | *Bacillus* sp. EB01 | 0.016858 | 0.022776 | 0.006741 | 0.016692 | Bacteria |
| 422 | *Thauera phenylacetica* | 0 | 0.012423 | 0.033704 | 0.016692 | Bacteria |
| 423 | *Alcanivorax hongdengensis* | 0 | 0.002071 | 0.060667 | 0 | Bacteria |
| 424 | *Flavobacterium rivuli* | 0 | 0.002071 | 0.060667 | 0 | Bacteria |
| 425 | *Vagococcus teuberi* | 0.043831 | 0.002071 | 0 | 0.016692 | Bacteria |
| 426 | *Bacillus velezensis* | 0.015172 | 0.002071 | 0.020222 | 0.025038 | Bacteria |
| 427 | *Bacillus subterraneus* | 0.001686 | 0.002071 | 0.033704 | 0.025038 | Bacteria |
| 428 | *Bacillus* sp. UNC41MFS5 | 0.006743 | 0.010353 | 0.020222 | 0.025038 | Bacteria |
| 429 | *Chryseobacterium takakiae* | 0 | 0 | 0.053927 | 0.008346 | Bacteria |
| 430 | *Massilia* sp. PDC64 | 0 | 0.008282 | 0.053927 | 0 | Bacteria |
| 431 | *Jeotgalibacillus* soli Cunha et al. 2012 | 0.001686 | 0.010353 | 0 | 0.050075 | Bacteria |
| 432 | *Bacillus ndiopicus* | 0.001686 | 0 | 0.026963 | 0.033383 | Bacteria |
| 433 | *Bacillus* sp. FJAT-27986 | 0.001686 | 0 | 0.026963 | 0.033383 | Bacteria |
| 434 | *Cupriavidus basilensis* | 0.001686 | 0.026917 | 0 | 0.033383 | Bacteria |
| 435 | *Clostridium* sp. Marseille-P299 | 0.003372 | 0.004141 | 0.053927 | 0 | Bacteria |
| 436 | *Salimicrobium halophilum* | 0 | 0.006212 | 0.013482 | 0.041729 | Bacteria |
| 437 | *Vibrio cyclitrophicus* | 0 | 0.022776 | 0.013482 | 0.025038 | Bacteria |
| 438 | *Aneurinibacillus tyrosinisolvens* | 0 | 0.002071 | 0.033704 | 0.025038 | Bacteria |
| 439 | *Ideonella sakaiensis* | 0 | 0.002071 | 0.033704 | 0.025038 | Bacteria |
| 440 | *Aliivibrio fischeri* | 0 | 0 | 0.060667 | 0 | Bacteria |
| 441 | *Flavobacterium gelidilacus* | 0 | 0 | 0.060667 | 0 | Bacteria |
| 442 | *Leadbetterella byssophila* | 0 | 0 | 0.060667 | 0 | Bacteria |
| 443 | *Polynucleobacter asymbioticus* | 0 | 0 | 0.060667 | 0 | Bacteria |
| 444 | *Bacillus* sp. MRMR6 | 0 | 0.002071 | 0 | 0.058421 | Bacteria |
| 445 | *Halolactibacillus halophilus* | 0 | 0.002071 | 0 | 0.058421 | Bacteria |
| 446 | *Acinetobacter larvae* | 0 | 0.008282 | 0.026963 | 0.025038 | Bacteria |
| 447 | *Anoxybacillus ayderensis* | 0.001686 | 0.004141 | 0.053927 | 0 | Bacteria |
| 448 | *Brenneria goodwinii* | 0 | 0.004141 | 0.047186 | 0.008346 | Bacteria |
| 449 | *Variovorax* sp. HW608 | 0 | 0.004141 | 0.047186 | 0.008346 | Bacteria |
| 450 | *Paenibacillus odorifer* | 0.006743 | 0.012423 | 0.040445 | 0 | Bacteria |
| 451 | *Thiomicrospira* sp. CG2_30_44_34 | 0 | 0.012423 | 0.047186 | 0 | Bacteria |
| 452 | *Pseudomonas litoralis* | 0.003372 | 0.012423 | 0.026963 | 0.016692 | Bacteria |
| 453 | *Enterococcus columbae* | 0.057317 | 0.002071 | 0 | 0 | Bacteria |
| 454 | *Marinomonas fungiae* | 0 | 0.002071 | 0.040445 | 0.016692 | Bacteria |
| 455 | *Melaminivora alkalimesophila* | 0 | 0 | 0.033704 | 0.025038 | Bacteria |
| 456 | *Massilia namucuonensis* | 0 | 0.024846 | 0.033704 | 0 | Bacteria |
| 457 | *Chromobacterium* sp. | 0 | 0 | 0 | 0.058421 | Bacteria |
| 458 | *Aneurinibacillus thermoaerophilus* | 0.015172 | 0.012423 | 0.013482 | 0.016692 | Bacteria |
| 459 | *Bacillus humi* | 0.02023 | 0.004141 | 0 | 0.033383 | Bacteria |
| 460 | *Gallaecimonas xiamenensis* | 0 | 0.012423 | 0.020222 | 0.025038 | Bacteria |
| 461 | *Caballeronia megalochromosomata* | 0 | 0.002071 | 0.047186 | 0.008346 | Bacteria |
| 462 | *Lactobacillus fermentum* | 0 | 0.002071 | 0.013482 | 0.041729 | Bacteria |
| 463 | *Bacillus* sp. HMSC76G11 | 0.006743 | 0 | 0 | 0.050075 | Bacteria |
| 464 | *Gracilibacillus massiliensis* | 0 | 0 | 0.006741 | 0.050075 | Bacteria |
| 465 | *Anaerobacillus alkalidiazotrophicus* | 0.006743 | 0.008282 | 0 | 0.041729 | Bacteria |
| 466 | *Leeia oryzae* | 0 | 0.016564 | 0.006741 | 0.033383 | Bacteria |
| 467 | *Herbaspirillum* sp. RV1423 | 0 | 0.024846 | 0.006741 | 0.025038 | Bacteria |
| 468 | *Azospirillum brasilense* | 0 | 0.006212 | 0.033704 | 0.016692 | Bacteria |
| 469 | *Bacillus vireti* | 0.006743 | 0.004141 | 0.020222 | 0.025038 | Bacteria |
| 470 | *Pseudomonas sabulinigri* | 0 | 0.004141 | 0.026963 | 0.025038 | Bacteria |
| 471 | *Anoxybacillus suryakundensis* | 0.035402 | 0.020705 | 0 | 0 | Bacteria |
| 472 | *Enterococcus aquimarinus* | 0.028659 | 0.010353 | 0 | 0.016692 | Bacteria |
| 473 | *Thauera* sp. ZV-1-C | 0 | 0.002071 | 0.020222 | 0.033383 | Bacteria |
| 474 | *Enterobacter cloacae* | 0 | 0.010353 | 0.020222 | 0.025038 | Bacteria |
| 475 | *Chryseobacterium chaponense* | 0 | 0 | 0.047186 | 0.008346 | Bacteria |
| 476 | *Ochrobactrum* sp. P6BS-III | 0 | 0 | 0.047186 | 0.008346 | Bacteria |
| 477 | *Bordetella* sp. SCN 67-23 | 0 | 0.006212 | 0.040445 | 0.008346 | Bacteria |
| 478 | *Fictibacillus phosphorivorans* | 0.003372 | 0.006212 | 0.020222 | 0.025038 | Bacteria |
| 479 | *Lactobacillus rhamnosus* | 0.010115 | 0.022776 | 0.013482 | 0.008346 | Bacteria |
| 480 | *Kurthia huakuii* | 0 | 0.006212 | 0.006741 | 0.041729 | Bacteria |
| 481 | *Caenibacillus caldisaponilyticus* | 0.013486 | 0.004141 | 0.020222 | 0.016692 | Bacteria |
| 482 | *Gracilibacillus kekensis* | 0 | 0.004141 | 0.033704 | 0.016692 | Bacteria |
| 483 | *Geobacillus thermoleovorans* | 0.026973 | 0.012423 | 0.006741 | 0.008346 | Bacteria |
| 484 | *Xanthomonas* sp. Mitacek01 | 0 | 0.012423 | 0.033704 | 0.008346 | Bacteria |
| 485 | *Bacillus weihaiensis* | 0.005057 | 0.004141 | 0.020222 | 0.025038 | Bacteria |
| 486 | *Brevibacillus laterosporus* | 0.025287 | 0.012423 | 0 | 0.016692 | Bacteria |
| 487 | *Bacillus anthracis* | 0.005057 | 0.012423 | 0.020222 | 0.016692 | Bacteria |
| 488 | *Listeria innocua* | 0.008429 | 0.012423 | 0 | 0.033383 | Bacteria |
| 489 | *Halobacillus massiliensis* | 0 | 0.004141 | 0 | 0.050075 | Bacteria |
| 490 | *Pantoea ananatis* | 0 | 0.004141 | 0 | 0.050075 | Bacteria |
| 491 | *Geobacillus* sp. BCO2 | 0.008429 | 0.020705 | 0 | 0.025038 | Bacteria |
| 492 | *Tepidimonas taiwanensis* | 0 | 0.012423 | 0 | 0.041729 | Bacteria |
| 493 | *Comamonas terrigena* | 0 | 0.010353 | 0.026963 | 0.016692 | Bacteria |
| 494 | *Oceanicola granulosus* | 0 | 0.010353 | 0.026963 | 0.016692 | Bacteria |
| 495 | *Flavobacterium* sp. MedPE-SWcel | 0 | 0 | 0.053927 | 0 | Bacteria |
| 496 | *Mariniphaga anaerophila* | 0 | 0 | 0.053927 | 0 | Bacteria |
| 497 | *Oceanospirillum multiglobuliferum* | 0 | 0 | 0.053927 | 0 | Bacteria |
| 498 | *Pedobacter africanus* | 0 | 0 | 0.053927 | 0 | Bacteria |
| 499 | *Psychrobacter* sp. SHUES1 | 0 | 0 | 0.053927 | 0 | Bacteria |
| 500 | *Siansivirga zeaxanthinifaciens* | 0 | 0 | 0.053927 | 0 | Bacteria |
| 501 | *Zhouia amylolytica* | 0 | 0 | 0.053927 | 0 | Bacteria |
| 502 | *Thiothrix disciformis* | 0 | 0.008282 | 0.020222 | 0.025038 | Bacteria |
| 503 | *Bacillus aryabhattai* | 0.011801 | 0 | 0 | 0.041729 | Bacteria |
| 504 | *Chlorobaculum limnaeum* | 0 | 0.006212 | 0.047186 | 0 | Bacteria |
| 505 | *Bacillus dielmoensis* | 0.038773 | 0.006212 | 0 | 0.008346 | Bacteria |
| 506 | *Bacillus acidicola* | 0.038773 | 0.014494 | 0 | 0 | Bacteria |
| 507 | *Caballeronia sordidicola* | 0 | 0.004141 | 0.040445 | 0.008346 | Bacteria |
| 508 | *Thiomonas* sp. CB2 | 0 | 0.031058 | 0.013482 | 0.008346 | Bacteria |
| 509 | *Thioalkalimicrobium aerophilum* | 0 | 0.012423 | 0.040445 | 0 | Bacteria |
| 510 | *Bacillus* sp. FJAT-25496 | 0.001686 | 0.004141 | 0.013482 | 0.033383 | Bacteria |
| 511 | *Psychrobacter* sp. DAB_AL43B | 0 | 0.012423 | 0.006741 | 0.033383 | Bacteria |
| 512 | *Paracoccus alcaliphilus* | 0 | 0.037269 | 0.006741 | 0.008346 | Bacteria |
| 513 | *Bacillus psychrosaccharolyticus* | 0.001686 | 0.002071 | 0.006741 | 0.041729 | Bacteria |
| 514 | *Providencia sneebia* | 0 | 0 | 0.026963 | 0.025038 | Bacteria |
| 515 | *Salinicoccus alkaliphilus* | 0 | 0 | 0.026963 | 0.025038 | Bacteria |
| 516 | *Thermovibrio ammonificans* | 0 | 0 | 0.026963 | 0.025038 | Bacteria |
| 517 | *Marinospirillum alkaliphilum* | 0 | 0.008282 | 0.026963 | 0.016692 | Bacteria |
| 518 | *Sporosarcina* sp. P37 | 0 | 0.008282 | 0.026963 | 0.016692 | Bacteria |
| 519 | *Xenophilus* *azovorans* | 0 | 0.008282 | 0.026963 | 0.016692 | Bacteria |
| 520 | *Psychrobacillus* sp. OK028 | 0.005057 | 0 | 0.013482 | 0.033383 | Bacteria |
| 521 | *Pontibacillus chungwhensis* | 0.001686 | 0 | 0 | 0.050075 | Bacteria |
| 522 | *Rodentibacter ratti* | 0 | 0.006212 | 0.020222 | 0.025038 | Bacteria |
| 523 | *Streptococcus salivarius* | 0.02023 | 0.022776 | 0 | 0.008346 | Bacteria |
| 524 | *Haemophilus haemolyticus* | 0 | 0.004141 | 0.047186 | 0 | Bacteria |
| 525 | *Rummeliibacillus stabekisii* | 0.006743 | 0.004141 | 0.006741 | 0.033383 | Bacteria |
| 526 | *Noviherbaspirillum massiliense* | 0 | 0.020705 | 0.013482 | 0.016692 | Bacteria |
| 527 | *Enterococcus mundtii* | 0.025287 | 0.002071 | 0.006741 | 0.016692 | Bacteria |
| 528 | *Enterococcus canintestini* | 0.023601 | 0 | 0.026963 | 0 | Bacteria |
| 529 | *Bacillus alkalitelluris* | 0.006743 | 0.002071 | 0 | 0.041729 | Bacteria |
| 530 | *Photobacterium damselae* | 0 | 0.010353 | 0.006741 | 0.033383 | Bacteria |
| 531 | *Enterococcus sulfureus* | 0.02023 | 0 | 0.013482 | 0.016692 | Bacteria |
| 532 | *Brevibacillus* sp. OK042 | 0 | 0.008282 | 0 | 0.041729 | Bacteria |
| 533 | *Microvirgula aerodenitrificans* | 0 | 0.006212 | 0.026963 | 0.016692 | Bacteria |
| 534 | *Sporolactobacillus terrae* | 0.005057 | 0.006212 | 0.013482 | 0.025038 | Bacteria |
| 535 | *Bacillus* sp. LF1 | 0.015172 | 0.004141 | 0.013482 | 0.016692 | Bacteria |
| 536 | *Bordetella* genomosp. 8 | 0 | 0.012423 | 0.020222 | 0.016692 | Bacteria |
| 537 | *Bacillus panaciterrae* | 0.005057 | 0.012423 | 0.006741 | 0.025038 | Bacteria |
| 538 | *Domibacillus robiginosus* | 0.006743 | 0.002071 | 0.040445 | 0 | Bacteria |
| 539 | *Bacillus enclensis* | 0 | 0.002071 | 0.047186 | 0 | Bacteria |
| 540 | *Flavobacterium frigidimaris* | 0 | 0.002071 | 0.047186 | 0 | Bacteria |
| 541 | *Paenibacillus* sp. FF9 | 0 | 0.002071 | 0.047186 | 0 | Bacteria |
| 542 | *Sphingobacterium* sp. 21 | 0 | 0.002071 | 0.047186 | 0 | Bacteria |
| 543 | *Clostridium acetireducens* | 0.005057 | 0.010353 | 0.033704 | 0 | Bacteria |
| 544 | *Paenibacillus* sp. GM2 | 0.023601 | 0.010353 | 0.006741 | 0.008346 | Bacteria |
| 545 | *Fictibacillus macauensis* | 0.021915 | 0.002071 | 0 | 0.025038 | Bacteria |
| 546 | *Oceanimonas* sp. GK1 | 0 | 0.018635 | 0.013482 | 0.016692 | Bacteria |
| 547 | *Anoxybacillus pushchinoensis* | 0 | 0 | 0.040445 | 0.008346 | Bacteria |
| 548 | *Flavobacterium branchiophilum* | 0 | 0 | 0.040445 | 0.008346 | Bacteria |
| 549 | *Nitrococcus mobilis* | 0 | 0.008282 | 0.040445 | 0 | Bacteria |
| 550 | *Morganella morganii* | 0.011801 | 0.016564 | 0.020222 | 0 | Bacteria |
| 551 | *Halalkalibacillus halophilus* | 0 | 0 | 0.006741 | 0.041729 | Bacteria |
| 552 | *Lactobacillus curvatus* | 0.043831 | 0.004141 | 0 | 0 | Bacteria |
| 553 | *Rubeoparvulum massiliense* | 0.001686 | 0.004141 | 0 | 0.041729 | Bacteria |
| 554 | *Conchiformibius steedae* | 0 | 0.002071 | 0.020222 | 0.025038 | Bacteria |
| 555 | *Halolactibacillus alkaliphilus* | 0 | 0.002071 | 0.020222 | 0.025038 | Bacteria |
| 556 | *Propionivibrio dicarboxylicus* | 0 | 0.002071 | 0.020222 | 0.025038 | Bacteria |
| 557 | *Anaerosalibacter* sp. Marseille-P3206 | 0 | 0 | 0.047186 | 0 | Bacteria |
| 558 | *Capnocytophaga canis* | 0 | 0 | 0.047186 | 0 | Bacteria |
| 559 | *Chryseobacterium hungaricum* | 0 | 0 | 0.047186 | 0 | Bacteria |
| 560 | *Cruoricaptor ignavus* | 0 | 0 | 0.047186 | 0 | Bacteria |
| 561 | *Elizabethkingia anophelis* | 0 | 0 | 0.047186 | 0 | Bacteria |
| 562 | *Flavobacterium* sp. 38-13 | 0 | 0 | 0.047186 | 0 | Bacteria |
| 563 | *Flavobacterium* sp. 40-81 | 0 | 0 | 0.047186 | 0 | Bacteria |
| 564 | *Flavobacterium* terrae | 0 | 0 | 0.047186 | 0 | Bacteria |
| 565 | *Niabella aurantiaca* | 0 | 0 | 0.047186 | 0 | Bacteria |
| 566 | *Ochrobactrum rhizosphaerae* | 0 | 0 | 0.047186 | 0 | Bacteria |
| 567 | *Pedobacter nyackensis* | 0 | 0 | 0.047186 | 0 | Bacteria |
| 568 | *Pseudaminobacter salicylatoxidans* | 0 | 0 | 0.047186 | 0 | Bacteria |
| 569 | *Pseudomonas veronii* | 0 | 0 | 0.047186 | 0 | Bacteria |
| 570 | *Sinorhizobium* sp. LM21 | 0 | 0 | 0.047186 | 0 | Bacteria |
| 571 | *Solitalea canadensis* | 0 | 0 | 0.047186 | 0 | Bacteria |
| 572 | *Bacillus lonarensis* | 0.006743 | 0.026917 | 0.013482 | 0 | Bacteria |
| 573 | *Brevibacillus* sp. CF112 | 0.001686 | 0 | 0.020222 | 0.025038 | Bacteria |
| 574 | *Enterococcus cecorum* | 0.030344 | 0.016564 | 0 | 0 | Bacteria |
| 575 | *Geobacillus* sp. WSUCF1 | 0.016858 | 0.016564 | 0.013482 | 0 | Bacteria |
| 576 | *Histophilus somni* | 0 | 0 | 0.013482 | 0.033383 | Bacteria |
| 577 | *Thorsellia anophelis* | 0 | 0.016564 | 0.013482 | 0.016692 | Bacteria |
| 578 | *Hydrogenoanaerobacterium saccharovorans* | 0.042145 | 0.004141 | 0 | 0 | Bacteria |
| 579 | *Clostridium kluyveri* | 0.001686 | 0.004141 | 0.040445 | 0 | Bacteria |
| 580 | *Herbaspirillum* sp. YR522 | 0 | 0.004141 | 0.033704 | 0.008346 | Bacteria |
| 581 | *Pseudospirillum japonicum* | 0 | 0.004141 | 0.033704 | 0.008346 | Bacteria |
| 582 | *Bacillus horneckiae* | 0.016858 | 0.004141 | 0 | 0.025038 | Bacteria |
| 583 | *Bacillus* sp. MUM 116 | 0.016858 | 0.012423 | 0 | 0.016692 | Bacteria |
| 584 | *Halobacillus aidingensis* | 0 | 0.004141 | 0 | 0.041729 | Bacteria |
| 585 | *Thiothrix eikelboomii* | 0 | 0.020705 | 0 | 0.025038 | Bacteria |
| 586 | *Lysinibacillus* sp. BF-4 | 0 | 0.002071 | 0.026963 | 0.016692 | Bacteria |
| 587 | *Variovorax paradoxus* | 0 | 0.010353 | 0.026963 | 0.008346 | Bacteria |
| 588 | *Clostridioides difficile* | 0.016858 | 0 | 0.020222 | 0.008346 | Bacteria |
| 589 | *Bacillus novalis* | 0.001686 | 0.018635 | 0 | 0.025038 | Bacteria |
| 590 | *Alloiococcus otitis* | 0 | 0 | 0.020222 | 0.025038 | Bacteria |
| 591 | *Burkholderia* sp. JS23 | 0 | 0.008282 | 0.020222 | 0.016692 | Bacteria |
| 592 | *Bacillus sinesaloumensis* | 0.005057 | 0.008282 | 0.006741 | 0.025038 | Bacteria |
| 593 | *Bacillus* sp. FJAT-22058 | 0.011801 | 0.024846 | 0 | 0.008346 | Bacteria |
| 594 | *Bacillus mycoides* | 0.001686 | 0.014494 | 0.020222 | 0.008346 | Bacteria |
| 595 | *Bordetella bronchiseptica* | 0 | 0.014494 | 0.013482 | 0.016692 | Bacteria |
| 596 | *Burkholderia* sp. TNe-862 | 0 | 0.004141 | 0.040445 | 0 | Bacteria |
| 597 | *Pseudomonas pseudoalcaligenes* | 0 | 0.004141 | 0.040445 | 0 | Bacteria |
| 598 | *Bacillus* sp. SA1-12 | 0.001686 | 0.004141 | 0.013482 | 0.025038 | Bacteria |
| 599 | *Hoeflea* sp. BAL378 | 0 | 0.012423 | 0.006741 | 0.025038 | Bacteria |
| 600 | *Nitrosospira multiformis* | 0 | 0.012423 | 0.006741 | 0.025038 | Bacteria |
| 601 | *Rhodoferax ferrireducens* | 0 | 0.012423 | 0.006741 | 0.025038 | Bacteria |
| 602 | *Aliihoeflea* sp. 2WW | 0 | 0.002071 | 0.033704 | 0.008346 | Bacteria |
| 603 | *Dysgonomonas* sp. BGC7 | 0 | 0.002071 | 0.033704 | 0.008346 | Bacteria |
| 604 | *Pseudomonas bauzanensis* | 0 | 0.002071 | 0.033704 | 0.008346 | Bacteria |
| 605 | *Sporosarcina globispora* | 0.016858 | 0.002071 | 0 | 0.025038 | Bacteria |
| 606 | *Pisciglobus halotolerans* | 0.043831 | 0 | 0 | 0 | Bacteria |
| 607 | *Riemerella anatipestifer* | 0.003372 | 0 | 0.040445 | 0 | Bacteria |
| 608 | *Pediococcus acidilactici* | 0 | 0.002071 | 0 | 0.041729 | Bacteria |
| 609 | *Bacillus* sp. 1NLA3E | 0 | 0.010353 | 0 | 0.033383 | Bacteria |
| 610 | *Bermanella marisrubri* | 0 | 0.010353 | 0 | 0.033383 | Bacteria |
| 611 | *Frischella perrara* | 0 | 0.010353 | 0 | 0.033383 | Bacteria |
| 612 | *Desemzia incerta* | 0.026973 | 0 | 0 | 0.016692 | Bacteria |
| 613 | *Vulcanibacillus modesticaldus* | 0 | 0 | 0.026963 | 0.016692 | Bacteria |
| 614 | *Marinococcus halophilus* | 0 | 0.008282 | 0.026963 | 0.008346 | Bacteria |
| 615 | *Conchiformibius kuhniae* | 0 | 0.016564 | 0.026963 | 0 | Bacteria |
| 616 | *Xanthomonas massiliensis* | 0 | 0.016564 | 0.026963 | 0 | Bacteria |
| 617 | *Clostridium perfringens* | 0.003372 | 0 | 0.006741 | 0.033383 | Bacteria |
| 618 | *Gracilibacillus orientalis* | 0.003372 | 0 | 0.006741 | 0.033383 | Bacteria |
| 619 | *Achromobacter* sp. 2789STDY5608625 | 0 | 0.022776 | 0.020222 | 0 | Bacteria |
| 620 | *Bacillus* sp. ES3 | 0.003372 | 0.006212 | 0 | 0.033383 | Bacteria |
| 621 | *Algoriella xinjiangensis* | 0 | 0.002071 | 0.040445 | 0 | Bacteria |
| 622 | *Acinetobacter rudis* | 0 | 0.002071 | 0.006741 | 0.033383 | Bacteria |
| 623 | *Paenisporosarcina indica* | 0 | 0.002071 | 0.006741 | 0.033383 | Bacteria |
| 624 | *Paenisporosarcina* sp. HGH0030 | 0 | 0.002071 | 0.006741 | 0.033383 | Bacteria |
| 625 | *Pandoraea thiooxydan*s | 0 | 0.010353 | 0.006741 | 0.025038 | Bacteria |
| 626 | *Bacillus* sp. NRRL B-41327 | 0.015172 | 0.026917 | 0 | 0 | Bacteria |
| 627 | *Moellerella wisconsensis* | 0.006743 | 0 | 0.026963 | 0.008346 | Bacteria |
| 628 | *Acinetobacter gerneri* | 0 | 0 | 0.033704 | 0.008346 | Bacteria |
| 629 | *Flavobacterium caeni* | 0 | 0 | 0.033704 | 0.008346 | Bacteria |
| 630 | *Gallibacterium salpingitidis* | 0 | 0 | 0.033704 | 0.008346 | Bacteria |
| 631 | *Gottschalkia acidurici* | 0 | 0 | 0.033704 | 0.008346 | Bacteria |
| 632 | *Kushneria aurantia* | 0 | 0 | 0.033704 | 0.008346 | Bacteria |
| 633 | *Limnobacter* sp. CACIAM 66H1 | 0 | 0 | 0.033704 | 0.008346 | Bacteria |
| 634 | *Paenibacillus durus* | 0 | 0 | 0.033704 | 0.008346 | Bacteria |
| 635 | *Pedobacter arcticus* | 0 | 0 | 0.033704 | 0.008346 | Bacteria |
| 636 | *Psychrobacter* sp. PRwf-1 | 0 | 0 | 0.033704 | 0.008346 | Bacteria |
| 637 | *Salinicoccus qingdaonensis* | 0 | 0 | 0.033704 | 0.008346 | Bacteria |
| 638 | *Lysinibacillus macroides* | 0.003372 | 0 | 0.013482 | 0.025038 | Bacteria |
| 639 | *Jeotgalibacillus malaysiensis* | 0.001686 | 0 | 0.006741 | 0.033383 | Bacteria |
| 640 | *Noviherbaspirillum* sp. Root189 | 0.001686 | 0.008282 | 0.006741 | 0.025038 | Bacteria |
| 641 | *Bacillus manliponensis* | 0 | 0 | 0 | 0.041729 | Bacteria |
| 642 | *Magnetovibrio blakemorei* | 0 | 0 | 0 | 0.041729 | Bacteria |
| 643 | *Salinicoccus albus* | 0 | 0 | 0 | 0.041729 | Bacteria |
| 644 | *Yaniella halotolerans* | 0 | 0 | 0 | 0.041729 | Bacteria |
| 645 | *Thiothrix flexilis* | 0 | 0.008282 | 0 | 0.033383 | Bacteria |
| 646 | *Chlamydia trachomatis* | 0.001686 | 0.014494 | 0 | 0.025038 | Bacteria |
| 647 | *Marinobacterium profundum* | 0.001686 | 0.004141 | 0.026963 | 0.008346 | Bacteria |
| 648 | *Psychrobacter* sp. 1501(2011) | 0 | 0.004141 | 0.020222 | 0.016692 | Bacteria |
| 649 | *Aquamicrobium defluvii* | 0 | 0.012423 | 0.020222 | 0.008346 | Bacteria |
| 650 | *Halomonas lutea* | 0 | 0.012423 | 0.020222 | 0.008346 | Bacteria |
| 651 | *Ottowia thiooxydans* | 0 | 0.012423 | 0.020222 | 0.008346 | Bacteria |
| 652 | *Enterococcus massiliensis* | 0.003372 | 0.012423 | 0 | 0.025038 | Bacteria |
| 653 | *Geobacillus icigianus* | 0.021915 | 0.002071 | 0 | 0.016692 | Bacteria |
| 654 | *Lysobacter concretionis* | 0.001686 | 0.010353 | 0.020222 | 0.008346 | Bacteria |
| 655 | *Marinobacterium georgiense* | 0 | 0.002071 | 0.013482 | 0.025038 | Bacteria |
| 656 | *Nitrincola nitratireducens* | 0 | 0.010353 | 0.013482 | 0.016692 | Bacteria |
| 657 | *Polynucleobacter* sp. MWH-Weng1-1 | 0 | 0.010353 | 0.013482 | 0.016692 | Bacteria |
| 658 | *Enterococcus termitis* | 0.040459 | 0 | 0 | 0 | Bacteria |
| 659 | *Achromobacter* sp. 2789STDY5608623 | 0 | 0 | 0.040445 | 0 | Bacteria |
| 660 | *Acinetobacter bereziniae* | 0 | 0 | 0.040445 | 0 | Bacteria |
| 661 | *Chryseobacterium* sp. IHB B 17019 | 0 | 0 | 0.040445 | 0 | Bacteria |
| 662 | *Flavobacterium akiainvivens* | 0 | 0 | 0.040445 | 0 | Bacteria |
| 663 | *Flavobacterium aquatile* | 0 | 0 | 0.040445 | 0 | Bacteria |
| 664 | *Flavobacterium haoranii* | 0 | 0 | 0.040445 | 0 | Bacteria |
| 665 | *Flavobacterium indicum* | 0 | 0 | 0.040445 | 0 | Bacteria |
| 666 | *Flavobacterium* sp. PK15 | 0 | 0 | 0.040445 | 0 | Bacteria |
| 667 | *Flavobacterium subsaxonicum* | 0 | 0 | 0.040445 | 0 | Bacteria |
| 668 | *Flavobacterium tegetincola* | 0 | 0 | 0.040445 | 0 | Bacteria |
| 669 | *Flavobacterium terrigena* | 0 | 0 | 0.040445 | 0 | Bacteria |
| 670 | *Gaetbulibacter saemankumensis* | 0 | 0 | 0.040445 | 0 | Bacteria |
| 671 | *Geobacillus* sp. 44C | 0 | 0 | 0.040445 | 0 | Bacteria |
| 672 | *Mesonia phycicola* | 0 | 0 | 0.040445 | 0 | Bacteria |
| 673 | *Ochrobactrum pseudogrignonense* | 0 | 0 | 0.040445 | 0 | Bacteria |
| 674 | *Pustulibacterium marinum* | 0 | 0 | 0.040445 | 0 | Bacteria |
| 675 | *Salinimicrobium catena* | 0 | 0 | 0.040445 | 0 | Bacteria |
| 676 | *Thioalkalivibrio* sp. ALJT | 0 | 0 | 0.040445 | 0 | Bacteria |
| 677 | *Enterococcus rivorum* | 0.023601 | 0 | 0 | 0.016692 | Bacteria |
| 678 | *Parvimonas micra* | 0.023601 | 0 | 0 | 0.016692 | Bacteria |
| 679 | *Providencia burhodogranariea* | 0.001686 | 0 | 0.013482 | 0.025038 | Bacteria |
| 680 | *Allofustis seminis* | 0.015172 | 0.008282 | 0 | 0.016692 | Bacteria |
| 681 | *Budvicia aquatica* | 0 | 0 | 0.006741 | 0.033383 | Bacteria |
| 682 | *Oceanobacillus* sp. E9 | 0 | 0 | 0.006741 | 0.033383 | Bacteria |
| 683 | *Paraburkholderia* sp. SOS3 | 0 | 0 | 0.006741 | 0.033383 | Bacteria |
| 684 | *Lactobacillus salivarius* | 0.006743 | 0.008282 | 0 | 0.025038 | Bacteria |
| 685 | *Snodgrassella alvi* | 0 | 0.008282 | 0.006741 | 0.025038 | Bacteria |
| 686 | *Enterobacter massiliensis* | 0 | 0.006212 | 0.033704 | 0 | Bacteria |
| 687 | *Janthinobacterium* sp. B9-8 | 0 | 0.033128 | 0.006741 | 0 | Bacteria |
| 688 | *Bacillus* sp. FJAT-44921 | 0.008429 | 0.006212 | 0 | 0.025038 | Bacteria |
| 689 | *Acinetobacter qingfengensis* | 0.001686 | 0.006212 | 0.006741 | 0.025038 | Bacteria |
| 690 | *Haemophilus parainfluenzae* | 0.001686 | 0.006212 | 0.006741 | 0.025038 | Bacteria |
| 691 | *Bacillus* sp. SG-1 | 0 | 0.006212 | 0 | 0.033383 | Bacteria |
| 692 | *Dechloromonas denitrificans* | 0 | 0.006212 | 0 | 0.033383 | Bacteria |
| 693 | *Lawsonia intracellularis* | 0 | 0.014494 | 0 | 0.025038 | Bacteria |
| 694 | *Uliginosibacterium gangwonense* | 0 | 0.014494 | 0 | 0.025038 | Bacteria |
| 695 | *Paraburkholderia andropogonis* | 0 | 0.012423 | 0.026963 | 0 | Bacteria |
| 696 | *Tuberibacillus* sp. Marseille-P3662 | 0.001686 | 0.004141 | 0 | 0.033383 | Bacteria |
| 697 | *Bacillus* sp. 491mf | 0.010115 | 0.028987 | 0 | 0 | Bacteria |
| 698 | *Bibersteinia trehalosi* | 0 | 0.002071 | 0.020222 | 0.016692 | Bacteria |
| 699 | *Burkholderia cepacia* | 0 | 0.002071 | 0.020222 | 0.016692 | Bacteria |
| 700 | *Tetragenococcus muriaticus* | 0.003372 | 0.002071 | 0 | 0.033383 | Bacteria |
| 701 | *Bacillus cytotoxicus* | 0.011801 | 0.018635 | 0 | 0.008346 | Bacteria |
| 702 | *Psychrobacillus psychrotolerans* | 0.008429 | 0 | 0.013482 | 0.016692 | Bacteria |
| 703 | *Bhargavaea ginsengi* | 0 | 0 | 0.013482 | 0.025038 | Bacteria |
| 704 | *Morganella psychrotolerans* | 0 | 0 | 0.013482 | 0.025038 | Bacteria |
| 705 | *Shimwellia blattae* | 0 | 0 | 0.013482 | 0.025038 | Bacteria |
| 706 | *Microbulbifer mangrovi* | 0 | 0.008282 | 0.013482 | 0.016692 | Bacteria |
| 707 | *Lactobacillus algidus* | 0.005057 | 0 | 0 | 0.033383 | Bacteria |
| 708 | *Achromobacter* sp. Root83 | 0 | 0.016564 | 0.013482 | 0.008346 | Bacteria |
| 709 | *Domibacillus antri* | 0.005057 | 0.006212 | 0.026963 | 0 | Bacteria |
| 710 | *Sporomusa malonica* | 0.015172 | 0.006212 | 0 | 0.016692 | Bacteria |
| 711 | *Bacillus oleronius* | 0.001686 | 0.006212 | 0.013482 | 0.016692 | Bacteria |
| 712 | *Bacillus testis* | 0.001686 | 0.006212 | 0.013482 | 0.016692 | Bacteria |
| 713 | *Thiomonas* sp. FB-6 | 0 | 0.014494 | 0.006741 | 0.016692 | Bacteria |
| 714 | *Aurantimonas* sp. 22II-16-19i | 0 | 0.004141 | 0.033704 | 0 | Bacteria |
| 715 | *Polaromonas* sp. C04 | 0 | 0.004141 | 0.033704 | 0 | Bacteria |
| 716 | *Achromobacter* sp. LC458 | 0 | 0.031058 | 0.006741 | 0 | Bacteria |
| 717 | *Oceanospirillum beijerinckii* | 0.005057 | 0.004141 | 0.020222 | 0.008346 | Bacteria |
| 718 | *Oceanobacter kriegii* | 0 | 0.004141 | 0 | 0.033383 | Bacteria |
| 719 | *Achromobacter arsenitoxydans* | 0 | 0.012423 | 0 | 0.025038 | Bacteria |
| 720 | *Deinococcus pimensis* | 0 | 0.012423 | 0 | 0.025038 | Bacteria |
| 721 | *Acidovorax* sp. RAC01 | 0 | 0.002071 | 0.026963 | 0.008346 | Bacteria |
| 722 | *Alteromonas macleodii* | 0 | 0.002071 | 0.026963 | 0.008346 | Bacteria |
| 723 | *Bacillus safensis* | 0.001686 | 0.002071 | 0 | 0.033383 | Bacteria |
| 724 | *Sporolactobacillus inulinus* | 0.001686 | 0.002071 | 0 | 0.033383 | Bacteria |
| 725 | *Desulfosporosinus* sp. OT | 0.021915 | 0 | 0.006741 | 0.008346 | Bacteria |
| 726 | *Streptococcus pyogenes* | 0.021915 | 0 | 0.006741 | 0.008346 | Bacteria |
| 727 | *Acinetobacter* sp. P8-3-8 | 0 | 0 | 0.020222 | 0.016692 | Bacteria |
| 728 | *Chishuiella changwenlii* | 0 | 0 | 0.020222 | 0.016692 | Bacteria |
| 729 | *Muricauda zhangzhouensis* | 0 | 0 | 0.020222 | 0.016692 | Bacteria |
| 730 | *Sphingobacterium* sp. CZ-UAM | 0 | 0 | 0.020222 | 0.016692 | Bacteria |
| 731 | *Numidum massiliense* | 0.003372 | 0 | 0 | 0.033383 | Bacteria |
| 732 | *Paenibacillus senegalensis* | 0.003372 | 0 | 0 | 0.033383 | Bacteria |
| 733 | *Thiomonas* sp. FB-Cd | 0 | 0.006212 | 0.013482 | 0.016692 | Bacteria |
| 734 | *Pseudomonas alcaligenes* | 0 | 0.014494 | 0.013482 | 0.008346 | Bacteria |
| 735 | *Yangia* sp. CCB-MM3 | 0 | 0.014494 | 0.013482 | 0.008346 | Bacteria |
| 736 | *Proteiniborus* sp. DW1 | 0.005057 | 0.004141 | 0.026963 | 0 | Bacteria |
| 737 | *Bacillus nakamurai* | 0.016858 | 0.004141 | 0.006741 | 0.008346 | Bacteria |
| 738 | *Halomonas elongata* | 0 | 0.012423 | 0.006741 | 0.016692 | Bacteria |
| 739 | *Halorhodospira halophila* | 0 | 0.002071 | 0.033704 | 0 | Bacteria |
| 740 | *Ochrobactrum intermedium* | 0 | 0.002071 | 0.033704 | 0 | Bacteria |
| 741 | *Escherichia vulneris* | 0.001686 | 0.002071 | 0.006741 | 0.025038 | Bacteria |
| 742 | *Clostridium* sp. Marseille-P2415 | 0 | 0.002071 | 0 | 0.033383 | Bacteria |
| 743 | *Nitrosomonas halophila* | 0 | 0.002071 | 0 | 0.033383 | Bacteria |
| 744 | *Pseudomonas yangmingensis* | 0 | 0.002071 | 0 | 0.033383 | Bacteria |
| 745 | *Salinisphaera shabanensis* | 0 | 0.002071 | 0 | 0.033383 | Bacteria |
| 746 | *Carnobacterium* sp. AT7 | 0.028659 | 0 | 0.006741 | 0 | Bacteria |
| 747 | *Paraburkholderia megapolitana* | 0 | 0.010353 | 0 | 0.025038 | Bacteria |
| 748 | *Azotobacter chroococcum* | 0 | 0 | 0.026963 | 0.008346 | Bacteria |
| 749 | *Bacillus* sp. Soil768D1 | 0 | 0 | 0.026963 | 0.008346 | Bacteria |
| 750 | *Bergeyella zoohelcum* | 0 | 0 | 0.026963 | 0.008346 | Bacteria |
| 751 | *Chryseobacterium* sp. SCN 40-13 | 0 | 0 | 0.026963 | 0.008346 | Bacteria |
| 752 | *Cytophaga hutchinsonii* | 0 | 0 | 0.026963 | 0.008346 | Bacteria |
| 753 | *Gramella* sp. LPB0144 | 0 | 0 | 0.026963 | 0.008346 | Bacteria |
| 754 | *Derxia lacustris* | 0 | 0.008282 | 0.026963 | 0 | Bacteria |
| 755 | *Alysiella crassa* | 0 | 0.035199 | 0 | 0 | Bacteria |
| 756 | *Thalassobacillus cyri* | 0.003372 | 0 | 0.006741 | 0.025038 | Bacteria |
| 757 | *Bacillus cihuensis* | 0.001686 | 0 | 0 | 0.033383 | Bacteria |
| 758 | *Halobacillus kuroshimensis* | 0.001686 | 0 | 0 | 0.033383 | Bacteria |
| 759 | *Vibrio* sp. M12-1144 | 0 | 0.014494 | 0.020222 | 0 | Bacteria |
| 760 | *Bacillus nealsonii* | 0.005057 | 0.006212 | 0.006741 | 0.016692 | Bacteria |
| 761 | *Paenibacillus macquariensis* | 0.010115 | 0.004141 | 0.020222 | 0 | Bacteria |
| 762 | *Brevibacillus parabrevis* | 0.001686 | 0.004141 | 0.020222 | 0.008346 | Bacteria |
| 763 | *Mucor ambiguus* | 0.001686 | 0.004141 | 0.020222 | 0.008346 | Eukaryota |
| 764 | *Staphylococcus epidermidis* | 0.006743 | 0.004141 | 0.006741 | 0.016692 | Bacteria |
| 765 | *Achromobacter denitrificans* | 0 | 0.004141 | 0.013482 | 0.016692 | Bacteria |
| 766 | *Leucothrix mucor* | 0 | 0.004141 | 0.013482 | 0.016692 | Bacteria |
| 767 | *Marinospirillum minutulum* | 0 | 0.004141 | 0.013482 | 0.016692 | Bacteria |
| 768 | *Methylibium* sp. CF059 | 0 | 0.004141 | 0.013482 | 0.016692 | Bacteria |
| 769 | *Comamonas* sp. B-9 | 0 | 0.012423 | 0.013482 | 0.008346 | Bacteria |
| 770 | *Bacillus* sp. J33 | 0.006743 | 0.002071 | 0 | 0.025038 | Bacteria |
| 771 | *Bacillus aidingensis* | 0 | 0.002071 | 0.006741 | 0.025038 | Bacteria |
| 772 | *Bacillus hemicellulosilyticus* | 0 | 0.002071 | 0.006741 | 0.025038 | Bacteria |
| 773 | *Ectothiorhodosinus mongolicus* | 0 | 0.002071 | 0.006741 | 0.025038 | Bacteria |
| 774 | *Halomonas muralis* | 0 | 0.002071 | 0.006741 | 0.025038 | Bacteria |
| 775 | *Herbaspirillum autotrophicum* | 0 | 0.010353 | 0.006741 | 0.016692 | Bacteria |
| 776 | *Moraxella caviae* | 0 | 0.010353 | 0.006741 | 0.016692 | Bacteria |
| 777 | *Trichococcus flocculiformis* | 0.013486 | 0 | 0.020222 | 0 | Bacteria |
| 778 | *Aquimarina agarivorans* | 0 | 0 | 0.033704 | 0 | Bacteria |
| 779 | *Brenneria* sp. EniD312 | 0 | 0 | 0.033704 | 0 | Bacteria |
| 780 | *Brevundimonas* sp. SH203 | 0 | 0 | 0.033704 | 0 | Bacteria |
| 781 | *Burkholderia latens* | 0 | 0 | 0.033704 | 0 | Bacteria |
| 782 | *Cellulophaga tyrosinoxydans* | 0 | 0 | 0.033704 | 0 | Bacteria |
| 783 | *Chitinophaga niabensis* | 0 | 0 | 0.033704 | 0 | Bacteria |
| 784 | *Chryseobacterium* sp. G972 | 0 | 0 | 0.033704 | 0 | Bacteria |
| 785 | *Chryseobacterium* sp. Leaf405 | 0 | 0 | 0.033704 | 0 | Bacteria |
| 786 | *Enterobacter asburiae* | 0 | 0 | 0.033704 | 0 | Bacteria |
| 787 | *Flavobacterium gilvum* | 0 | 0 | 0.033704 | 0 | Bacteria |
| 788 | *Flavobacterium limnosediminis* | 0 | 0 | 0.033704 | 0 | Bacteria |
| 789 | *Flavobacterium noncentrifugens* | 0 | 0 | 0.033704 | 0 | Bacteria |
| 790 | *Geobacillus* sp. Y412MC61 | 0 | 0 | 0.033704 | 0 | Bacteria |
| 791 | *Luteimonas huabeiensis* | 0 | 0 | 0.033704 | 0 | Bacteria |
| 792 | *Mangrovimonas* sp. TPBH4 | 0 | 0 | 0.033704 | 0 | Bacteria |
| 793 | *Neorhizobium galegae* | 0 | 0 | 0.033704 | 0 | Bacteria |
| 794 | *Nitratireductor pacificus* | 0 | 0 | 0.033704 | 0 | Bacteria |
| 795 | *Paenibacillus* sp. FSL R7-0337 | 0 | 0 | 0.033704 | 0 | Bacteria |
| 796 | *Serratia odorifera* | 0 | 0 | 0.033704 | 0 | Bacteria |
| 797 | *Snodgrassella* sp. R-53583 | 0 | 0 | 0.033704 | 0 | Bacteria |
| 798 | *Tenacibaculum maritimum* | 0 | 0 | 0.033704 | 0 | Bacteria |
| 799 | *Thermonema rossianum* | 0 | 0 | 0.033704 | 0 | Bacteria |
| 800 | *Listeria fleischmannii* | 0.025287 | 0 | 0 | 0.008346 | Bacteria |
| 801 | *Bacillus daliensis* | 0.008429 | 0 | 0 | 0.025038 | Bacteria |
| 802 | *Lysinibacillus saudimassiliensis* | 0.001686 | 0 | 0.006741 | 0.025038 | Bacteria |
| 803 | *Paenibacillus ihumii* | 0.001686 | 0 | 0.006741 | 0.025038 | Bacteria |
| 804 | *Acinetobacter pittii* | 0 | 0 | 0 | 0.033383 | Bacteria |
| 805 | *Aneurinibacillus migulanus* | 0 | 0 | 0 | 0.033383 | Bacteria |
| 806 | *Bacillus cecembensis* | 0 | 0 | 0 | 0.033383 | Bacteria |
| 807 | *Bacillus oryziterrae* | 0 | 0 | 0 | 0.033383 | Bacteria |
| 808 | *Bacillus* sp. FJAT-18017 | 0 | 0 | 0 | 0.033383 | Bacteria |
| 809 | *Clostridium collagenovorans* | 0 | 0 | 0 | 0.033383 | Bacteria |
| 810 | *Clostridium* sp. DL-VIII | 0 | 0 | 0 | 0.033383 | Bacteria |
| 811 | *Coxiella burnetii* | 0 | 0 | 0 | 0.033383 | Bacteria |
| 812 | *Erwinia billingiae* | 0 | 0 | 0 | 0.033383 | Bacteria |
| 813 | *Halomonas titanicae* | 0 | 0 | 0 | 0.033383 | Bacteria |
| 814 | *Pontibacillus marinus* | 0 | 0 | 0 | 0.033383 | Bacteria |
| 815 | *Saccharomonospora viridis* | 0 | 0 | 0 | 0.033383 | Bacteria |
| 816 | *Stenoxybacter acetivorans* | 0 | 0 | 0 | 0.033383 | Bacteria |
| 817 | *Virgibacillus salinus* | 0 | 0 | 0 | 0.033383 | Bacteria |
| 818 | *Burkholderia* sp. UYPR1.413 | 0 | 0.008282 | 0 | 0.025038 | Bacteria |
| 819 | *Cardiobacterium hominis* | 0 | 0.008282 | 0 | 0.025038 | Bacteria |
| 820 | *Comamonas* sp. SCN 65-56 | 0 | 0.008282 | 0 | 0.025038 | Bacteria |
| 821 | *Aquaspirillum serpens* | 0 | 0.006212 | 0.026963 | 0 | Bacteria |
| 822 | *Fangia hongkongensis* | 0.001686 | 0.006212 | 0 | 0.025038 | Bacteria |
| 823 | *Garciella nitratireducens* | 0.001686 | 0.006212 | 0 | 0.025038 | Bacteria |
| 824 | *Paenibacillus glucanolyticus* | 0.010115 | 0.022776 | 0 | 0 | Bacteria |
| 825 | *Bacillus* sp. URHB0009 | 0.001686 | 0.022776 | 0 | 0.008346 | Bacteria |
| 826 | *Lactococcus lactis* | 0.013486 | 0.004141 | 0.006741 | 0.008346 | Bacteria |
| 827 | *Aeromonas* sp. RU39B | 0 | 0.004141 | 0.020222 | 0.008346 | Bacteria |
| 828 | *Desulfosporosinus lacus* | 0 | 0.004141 | 0.020222 | 0.008346 | Bacteria |
| 829 | *Oceanimonas smirnovii* | 0 | 0.012423 | 0.020222 | 0 | Bacteria |
| 830 | *Bacillus* sp. FJAT-27916 | 0.003372 | 0.004141 | 0 | 0.025038 | Bacteria |
| 831 | *Geobacillus* sp. PA-3 | 0.003372 | 0.002071 | 0.026963 | 0 | Bacteria |
| 832 | *Lysinibacillus* sp. FJAT-14745 | 0.003372 | 0.002071 | 0.026963 | 0 | Bacteria |
| 833 | *Hahella chejuensis* | 0 | 0.002071 | 0.013482 | 0.016692 | Bacteria |
| 834 | *Proteiniphilum saccharofermentans* | 0 | 0.002071 | 0.013482 | 0.016692 | Bacteria |
| 835 | *Woeseia oceani* | 0 | 0.002071 | 0.013482 | 0.016692 | Bacteria |
| 836 | *Bacillus chagannorensis* | 0.005057 | 0.002071 | 0 | 0.025038 | Bacteria |
| 837 | *Mycobacterium asiaticum* | 0.03203 | 0 | 0 | 0 | Bacteria |
| 838 | *Enterococcus malodoratus* | 0.023601 | 0 | 0 | 0.008346 | Bacteria |
| 839 | *Pragia fontium* | 0.003372 | 0 | 0.020222 | 0.008346 | Bacteria |
| 840 | *Carnobacterium gallinarum* | 0.015172 | 0 | 0 | 0.016692 | Bacteria |
| 841 | *Paenibacillus elgii* | 0.015172 | 0 | 0 | 0.016692 | Bacteria |
| 842 | *Domibacillus* sp. SAB 38 | 0.008429 | 0 | 0.006741 | 0.016692 | Bacteria |
| 843 | *Varibaculum timonense* | 0.001686 | 0 | 0.013482 | 0.016692 | Bacteria |
| 844 | *Bacillus kribbensis* | 0.006743 | 0 | 0 | 0.025038 | Bacteria |
| 845 | *Azospira oryzae* | 0 | 0 | 0.006741 | 0.025038 | Bacteria |
| 846 | *Planococcus* sp. CAU13 | 0 | 0 | 0.006741 | 0.025038 | Bacteria |
| 847 | *Thalassobacillus* sp. C254 | 0 | 0 | 0.006741 | 0.025038 | Bacteria |
| 848 | *Vibrio parahaemolyticus* | 0 | 0 | 0.006741 | 0.025038 | Bacteria |
| 849 | *Acidihalobacter prosperus* | 0 | 0.008282 | 0.006741 | 0.016692 | Bacteria |
| 850 | *Brachymonas chironomi* | 0 | 0.008282 | 0.006741 | 0.016692 | Bacteria |
| 851 | *Halomonas pantelleriensis* | 0.003372 | 0.006212 | 0.013482 | 0.008346 | Bacteria |
| 852 | *Erwinia teleogrylli* | 0 | 0.006212 | 0 | 0.025038 | Bacteria |
| 853 | *Pseudomonas argentinensis* | 0 | 0.006212 | 0 | 0.025038 | Bacteria |
| 854 | *Roseomonas rosea* | 0 | 0.006212 | 0 | 0.025038 | Bacteria |
| 855 | *Trichococcus palustris* | 0 | 0.006212 | 0 | 0.025038 | Bacteria |
| 856 | *Oxalobacter formigenes* | 0 | 0.014494 | 0 | 0.016692 | Bacteria |
| 857 | *Bacillus encimensis* | 0 | 0.004141 | 0.026963 | 0 | Bacteria |
| 858 | *Carnimonas nigrificans* | 0 | 0.004141 | 0.026963 | 0 | Bacteria |
| 859 | *Ralstonia syzygii* | 0 | 0.004141 | 0.026963 | 0 | Bacteria |
| 860 | *Neptuniibacter caesariensis* | 0.001686 | 0.004141 | 0 | 0.025038 | Bacteria |
| 861 | *Bacillus* sp. FJAT-29937 | 0 | 0.002071 | 0.020222 | 0.008346 | Bacteria |
| 862 | *Massilia* sp. Leaf139 | 0 | 0.002071 | 0.020222 | 0.008346 | Bacteria |
| 863 | *Flavobacterium reichenbachii* | 0 | 0.010353 | 0.020222 | 0 | Bacteria |
| 864 | *Pandoraea* sp. SD6-2 | 0 | 0.010353 | 0.020222 | 0 | Bacteria |
| 865 | *Psychrobacillus psychrodurans* | 0.003372 | 0.002071 | 0 | 0.025038 | Bacteria |
| 866 | *Salsuginibacillus kocurii* | 0.003372 | 0.002071 | 0 | 0.025038 | Bacteria |
| 867 | *Bacillus* sp. 522_BSPC | 0.010115 | 0 | 0.020222 | 0 | Bacteria |
| 868 | *Enterococcus canis* | 0.021915 | 0 | 0 | 0.008346 | Bacteria |
| 869 | *Anaerobacillus arseniciselenatis* | 0 | 0 | 0.013482 | 0.016692 | Bacteria |
| 870 | *Bacillus coahuilensis* | 0 | 0 | 0.013482 | 0.016692 | Bacteria |
| 871 | *Brevibacillus massiliensis* | 0 | 0 | 0.013482 | 0.016692 | Bacteria |
| 872 | *Flexilinea flocculi* | 0 | 0 | 0.013482 | 0.016692 | Bacteria |
| 873 | *Kurthia massiliensis* | 0 | 0 | 0.013482 | 0.016692 | Bacteria |
| 874 | *Parabacteroides* sp. Marseille-P3160 | 0 | 0 | 0.013482 | 0.016692 | Bacteria |
| 875 | *Proteiniborus ethanoligenes* | 0 | 0 | 0.013482 | 0.016692 | Bacteria |
| 876 | *Pseudomonas agarici* | 0 | 0 | 0.013482 | 0.016692 | Bacteria |
| 877 | *Psychromonas* sp. SP041 | 0 | 0 | 0.013482 | 0.016692 | Bacteria |
| 878 | *Paenibacillus tianmuensis* | 0.005057 | 0 | 0 | 0.025038 | Bacteria |
| 879 | *Parabacteroides* sp. YL27 | 0 | 0.016564 | 0.013482 | 0 | Bacteria |
| 880 | *Sporanaerobacter* sp. PP17-6a | 0.005057 | 0.008282 | 0 | 0.016692 | Bacteria |
| 881 | *Geobacillus* sp. Sah69 | 0.001686 | 0.006212 | 0.013482 | 0.008346 | Bacteria |
| 882 | *Bacillus* sp. FJAT-25547 | 0 | 0.006212 | 0.006741 | 0.016692 | Bacteria |
| 883 | *Rhodopseudomonas palustris* | 0 | 0.006212 | 0.006741 | 0.016692 | Bacteria |
| 884 | *Verminephrobacter eiseniae* | 0 | 0.006212 | 0.006741 | 0.016692 | Bacteria |
| 885 | *Azovibrio restrictus* | 0 | 0.014494 | 0.006741 | 0.008346 | Bacteria |
| 886 | *Enhydrobacter aerosaccus* | 0 | 0.004141 | 0 | 0.025038 | Bacteria |
| 887 | *Gelidibacter mesophilus* | 0 | 0.004141 | 0 | 0.025038 | Bacteria |
| 888 | *Ramlibacter tataouinensis* | 0 | 0.004141 | 0 | 0.025038 | Bacteria |
| 889 | *Moraxella atlantae* | 0 | 0.012423 | 0 | 0.016692 | Bacteria |
| 890 | *Bradyrhizobium* sp. NFR13 | 0 | 0.002071 | 0.026963 | 0 | Bacteria |
| 891 | *Methylococcus capsulatus* | 0 | 0.002071 | 0.026963 | 0 | Bacteria |
| 892 | *Sinomicrobium oceani* | 0 | 0.002071 | 0.026963 | 0 | Bacteria |
| 893 | *Sphingobacterium* sp. B29 | 0 | 0.002071 | 0.026963 | 0 | Bacteria |
| 894 | *Vibrio crassostreae* | 0 | 0.002071 | 0.026963 | 0 | Bacteria |
| 895 | *Clostridium pasteurianum* | 0.018544 | 0.002071 | 0 | 0.008346 | Bacteria |
| 896 | *Staphylococcus massiliensis* | 0.018544 | 0.002071 | 0 | 0.008346 | Bacteria |
| 897 | *Lactobacillus casei* | 0.010115 | 0.010353 | 0 | 0.008346 | Bacteria |
| 898 | *Streptococcus gordonii* | 0.010115 | 0.010353 | 0 | 0.008346 | Bacteria |
| 899 | *Jeotgalibacillus campisalis* | 0.001686 | 0.002071 | 0 | 0.025038 | Bacteria |
| 900 | *Anaerobium acetethylicum* | 0.028659 | 0 | 0 | 0 | Bacteria |
| 901 | *Enterococcus thailandicus* | 0.02023 | 0 | 0 | 0.008346 | Bacteria |
| 902 | *Alteromonas* sp. Nap_26 | 0 | 0 | 0.020222 | 0.008346 | Bacteria |
| 903 | *Flavisolibacter ginsengisoli* | 0 | 0 | 0.020222 | 0.008346 | Bacteria |
| 904 | *Galbibacter marinus* | 0 | 0 | 0.020222 | 0.008346 | Bacteria |
| 905 | *Marinospirillum celere* | 0 | 0 | 0.020222 | 0.008346 | Bacteria |
| 906 | *Niabella drilacis* | 0 | 0 | 0.020222 | 0.008346 | Bacteria |
| 907 | *Paramaledivibacter caminithermalis* | 0 | 0 | 0.020222 | 0.008346 | Bacteria |
| 908 | *Pseudomonas cichorii* | 0 | 0 | 0.020222 | 0.008346 | Bacteria |
| 909 | *Tropicibacter litoreus* | 0 | 0 | 0.020222 | 0.008346 | Bacteria |
| 910 | *Neisseria dentiae* | 0 | 0.008282 | 0.020222 | 0 | Bacteria |
| 911 | *Neisseria lactamica* | 0 | 0.008282 | 0.020222 | 0 | Bacteria |
| 912 | *Pedobacter oryzae* | 0 | 0.008282 | 0.020222 | 0 | Bacteria |
| 913 | *Erysipelothrix rhusiopathiae* | 0.011801 | 0 | 0 | 0.016692 | Bacteria |
| 914 | *Oceanobacillus kimchii* | 0.003372 | 0 | 0 | 0.025038 | Bacteria |
| 915 | *Salimicrobium flavidum* | 0.003372 | 0 | 0 | 0.025038 | Bacteria |
| 916 | *Burkholderia* sp. JPY347 | 0 | 0.006212 | 0.013482 | 0.008346 | Bacteria |
| 917 | *Cupriavidus pauculus* | 0 | 0.006212 | 0.013482 | 0.008346 | Bacteria |
| 918 | *Neisseria bacilliformis* | 0 | 0.004141 | 0.006741 | 0.016692 | Bacteria |
| 919 | *Pseudomonas* sp. TTU2014-080ASC | 0 | 0.012423 | 0.006741 | 0.008346 | Bacteria |
| 920 | *Enterococcus devriesei* | 0.025287 | 0.002071 | 0 | 0 | Bacteria |
| 921 | *Alicyclobacillus* sp. USBA-503 | 0 | 0.002071 | 0 | 0.025038 | Bacteria |
| 922 | *Andreprevotia chitinilytica* | 0 | 0.002071 | 0 | 0.025038 | Bacteria |
| 923 | *Bacillus* sp. FJAT-27997 | 0 | 0.002071 | 0 | 0.025038 | Bacteria |
| 924 | *Burkholderia* sp. Leaf177 | 0 | 0.002071 | 0 | 0.025038 | Bacteria |
| 925 | *Dickeya paradisiaca* | 0 | 0.002071 | 0 | 0.025038 | Bacteria |
| 926 | *Hydrogenophaga* sp. LPB0072 | 0 | 0.002071 | 0 | 0.025038 | Bacteria |
| 927 | *Laribacter hongkongensis* | 0 | 0.002071 | 0 | 0.025038 | Bacteria |
| 928 | *Melghirimyces thermohalophilus* | 0 | 0.002071 | 0 | 0.025038 | Bacteria |
| 929 | *Moraxella bovoculi* | 0 | 0.002071 | 0 | 0.025038 | Bacteria |
| 930 | *Muribacter muris* | 0 | 0.002071 | 0 | 0.025038 | Bacteria |
| 931 | *Pelotomaculum thermopropionicum* | 0 | 0.002071 | 0 | 0.025038 | Bacteria |
| 932 | *Pseudomonas resinovorans* | 0 | 0.002071 | 0 | 0.025038 | Bacteria |
| 933 | *Pseudoxanthomonas* sp. GM95 | 0 | 0.002071 | 0 | 0.025038 | Bacteria |
| 934 | *Rhizobacter* sp. Root1221 | 0 | 0.002071 | 0 | 0.025038 | Bacteria |
| 935 | *Thioalkalivibrio denitrificans* | 0 | 0.002071 | 0 | 0.025038 | Bacteria |
| 936 | *Virgibacillus subterraneus* | 0 | 0.002071 | 0 | 0.025038 | Bacteria |
| 937 | *Ectothiorhodospira magna* | 0 | 0.010353 | 0 | 0.016692 | Bacteria |
| 938 | *Methylobacillus flagellatus* | 0 | 0.010353 | 0 | 0.016692 | Bacteria |
| 939 | *Carnobacterium divergens* | 0.026973 | 0 | 0 | 0 | Bacteria |
| 940 | *Carnobacterium mobile* | 0.013486 | 0 | 0.013482 | 0 | Bacteria |
| 941 | *Achromobacter* sp. NFACC18-2 | 0 | 0 | 0.026963 | 0 | Bacteria |
| 942 | *Acinetobacter* sp. NRRL B-65365 | 0 | 0 | 0.026963 | 0 | Bacteria |
| 943 | *Adhaeribacter aquaticus* | 0 | 0 | 0.026963 | 0 | Bacteria |
| 944 | *Ahrensia kielensis* | 0 | 0 | 0.026963 | 0 | Bacteria |
| 945 | *Albimonas donghaensis* | 0 | 0 | 0.026963 | 0 | Bacteria |
| 946 | *Algoriphagus* sp. NH1 | 0 | 0 | 0.026963 | 0 | Bacteria |
| 947 | *Bartonella koehlerae* | 0 | 0 | 0.026963 | 0 | Bacteria |
| 948 | *Capnocytophaga cynodegmi* | 0 | 0 | 0.026963 | 0 | Bacteria |
| 949 | *Capnocytophaga* sp. oral taxon 329 | 0 | 0 | 0.026963 | 0 | Bacteria |
| 950 | *Chitinophaga arvensicola* | 0 | 0 | 0.026963 | 0 | Bacteria |
| 951 | *Chryseobacterium gleum* | 0 | 0 | 0.026963 | 0 | Bacteria |
| 952 | *Chryseobacterium* sp. J200 | 0 | 0 | 0.026963 | 0 | Bacteria |
| 953 | *Flavobacterium cauense* | 0 | 0 | 0.026963 | 0 | Bacteria |
| 954 | *Flavobacterium phragmitis* | 0 | 0 | 0.026963 | 0 | Bacteria |
| 955 | *Flavobacterium saliperosum* | 0 | 0 | 0.026963 | 0 | Bacteria |
| 956 | *Flavobacterium seoulense* | 0 | 0 | 0.026963 | 0 | Bacteria |
| 957 | *Flavobacterium succinicans* | 0 | 0 | 0.026963 | 0 | Bacteria |
| 958 | *Flavobacterium urocaniciphilum* | 0 | 0 | 0.026963 | 0 | Bacteria |
| 959 | *Flexithrix dorotheae* | 0 | 0 | 0.026963 | 0 | Bacteria |
| 960 | *Formosa haliotis* | 0 | 0 | 0.026963 | 0 | Bacteria |
| 961 | *Imtechella halotolerans* | 0 | 0 | 0.026963 | 0 | Bacteria |
| 962 | *Luteimonas abyssi* | 0 | 0 | 0.026963 | 0 | Bacteria |
| 963 | *Lysobacter defluvii* | 0 | 0 | 0.026963 | 0 | Bacteria |
| 964 | *Lysobacter spongiicola* | 0 | 0 | 0.026963 | 0 | Bacteria |
| 965 | *Marinobacter subterrani* | 0 | 0 | 0.026963 | 0 | Bacteria |
| 966 | *Meiothermus rufus* | 0 | 0 | 0.026963 | 0 | Bacteria |
| 967 | *Mesorhizobium* sp. B7 | 0 | 0 | 0.026963 | 0 | Bacteria |
| 968 | *Microvirga vignae* | 0 | 0 | 0.026963 | 0 | Bacteria |
| 969 | *Niastella vici* | 0 | 0 | 0.026963 | 0 | Bacteria |
| 970 | *Paracoccus solventivorans* | 0 | 0 | 0.026963 | 0 | Bacteria |
| 971 | *Parageobacillus* genomosp. 1 | 0 | 0 | 0.026963 | 0 | Bacteria |
| 972 | *Parapedobacter luteus* | 0 | 0 | 0.026963 | 0 | Bacteria |
| 973 | *Plesiomonas shigelloides* | 0 | 0 | 0.026963 | 0 | Bacteria |
| 974 | *Psychroflexus halocasei* | 0 | 0 | 0.026963 | 0 | Bacteria |
| 975 | *Psychroflexus tropicus* | 0 | 0 | 0.026963 | 0 | Bacteria |
| 976 | *Rhizobium grahamii* | 0 | 0 | 0.026963 | 0 | Bacteria |
| 977 | *Sphingobacterium* sp. PM2-P1-29 | 0 | 0 | 0.026963 | 0 | Bacteria |
| 978 | *Sporocytophaga myxococcoides* | 0 | 0 | 0.026963 | 0 | Bacteria |
| 979 | *Variovorax* sp. Root318D1 | 0 | 0 | 0.026963 | 0 | Bacteria |
| 980 | *Enterococcus ureilyticus* | 0.018544 | 0 | 0 | 0.008346 | Bacteria |
| 981 | *Hungatella hathewayi* | 0.010115 | 0 | 0 | 0.016692 | Bacteria |
| 982 | *Bacillus jeotgali* | 0.003372 | 0 | 0.006741 | 0.016692 | Bacteria |
| 983 | *Bacillus* sp. JCM 19034 | 0.003372 | 0 | 0.006741 | 0.016692 | Bacteria |
| 984 | *Bacillus indicus* | 0.001686 | 0 | 0 | 0.025038 | Bacteria |
| 985 | *Brevibacillus panacihumi* | 0.001686 | 0 | 0 | 0.025038 | Bacteria |
| 986 | *Desulfuribacillus stibiiarsenatis* | 0.001686 | 0 | 0 | 0.025038 | Bacteria |
| 987 | *Domibacillus iocasae* | 0.001686 | 0 | 0 | 0.025038 | Bacteria |
| 988 | *Erysipelothrix larvae* | 0.001686 | 0 | 0 | 0.025038 | Bacteria |
| 989 | *Planifilum fulgidum* | 0.001686 | 0 | 0 | 0.025038 | Bacteria |
| 990 | *Crenobacter luteus* | 0 | 0.006212 | 0.020222 | 0 | Bacteria |
| 991 | *Rubrivivax* sp. SCN 70-15 | 0 | 0.006212 | 0.020222 | 0 | Bacteria |
| 992 | *Acinetobacter nosocomialis* | 0 | 0.004141 | 0.013482 | 0.008346 | Bacteria |
| 993 | *Duganella sacchari* | 0 | 0.004141 | 0.013482 | 0.008346 | Bacteria |
| 994 | *Lactobacillus parabrevis* | 0 | 0.004141 | 0.013482 | 0.008346 | Bacteria |
| 995 | *Pseudomonas monteilii* | 0 | 0.004141 | 0.013482 | 0.008346 | Bacteria |
| 996 | *Burkholderia* sp. yr281 | 0 | 0.012423 | 0.013482 | 0 | Bacteria |
| 997 | *Idiomarina* sp. A28L | 0 | 0.012423 | 0.013482 | 0 | Bacteria |
| 998 | *Janthinobacterium agaricidamnosum* | 0 | 0.012423 | 0.013482 | 0 | Bacteria |
| 999 | *Sporolactobacillus nakayamae* | 0.015172 | 0.002071 | 0 | 0.008346 | Bacteria |
| 1000 | *Bacillus* sp. J37 | 0.001686 | 0.002071 | 0.013482 | 0.008346 | Bacteria |
| 1001 | *Clostridium* sp. W14A | 0.015172 | 0.010353 | 0 | 0 | Bacteria |
| 1002 | *Bergeriella denitrificans* | 0 | 0.002071 | 0.006741 | 0.016692 | Bacteria |
| 1003 | *Planomicrobium glaciei* | 0 | 0.002071 | 0.006741 | 0.016692 | Bacteria |
| 1004 | *Xenorhabdus mauleonii* | 0 | 0.002071 | 0.006741 | 0.016692 | Bacteria |
| 1005 | *Andreprevotia lacus* | 0 | 0.010353 | 0.006741 | 0.008346 | Bacteria |
| 1006 | *Streptococcus mitis* | 0.025287 | 0 | 0 | 0 | Bacteria |
| 1007 | *Geobacillus jurassicus* | 0.011801 | 0 | 0.013482 | 0 | Bacteria |
| 1008 | *Atopobium fossor* | 0.008429 | 0 | 0 | 0.016692 | Bacteria |
| 1009 | *Paenibacillus* sp. JCM 10914 | 0.008429 | 0 | 0 | 0.016692 | Bacteria |
| 1010 | *Bacillus solimangrovi* | 0.001686 | 0 | 0.006741 | 0.016692 | Bacteria |
| 1011 | *Lysinibacillus* sp. ZYM-1 | 0.001686 | 0 | 0.006741 | 0.016692 | Bacteria |
| 1012 | *Massilia timonae* | 0.001686 | 0 | 0.006741 | 0.016692 | Bacteria |
| 1013 | *Achromobacter* phage phiAxp-3 | 0 | 0 | 0 | 0.025038 | Viruses |
| 1014 | *Amphritea atlantica* | 0 | 0.008282 | 0 | 0.016692 | Bacteria |
| 1015 | *Frankia* sp. EUN1f | 0 | 0.008282 | 0 | 0.016692 | Bacteria |
| 1016 | *Geobacter* sp. OR-1 | 0 | 0.008282 | 0 | 0.016692 | Bacteria |
| 1017 | *Hydrogenophaga taeniospiralis* | 0 | 0.008282 | 0 | 0.016692 | Bacteria |
| 1018 | *Paenibacillus amylolyticus* | 0 | 0.008282 | 0 | 0.016692 | Bacteria |
| 1019 | *Veillonella* sp. CAG:933 | 0 | 0.008282 | 0 | 0.016692 | Bacteria |
| 1020 | *Marinimicrobium* sp. LS-A18 | 0 | 0.016564 | 0 | 0.008346 | Bacteria |
| 1021 | *Paraburkholderia ribeironis* | 0 | 0.016564 | 0 | 0.008346 | Bacteria |
| 1022 | *Tepidiphilus thermophilus* | 0 | 0.016564 | 0 | 0.008346 | Bacteria |
| 1023 | *Achromobacter* sp. ATCC31444 | 0 | 0.024846 | 0 | 0 | Bacteria |
| 1024 | *Bacillus* sp. CDB3 | 0.018544 | 0.006212 | 0 | 0 | Bacteria |
| 1025 | *Achromobacter* sp. 2789STDY5663426 | 0 | 0.004141 | 0.020222 | 0 | Bacteria |
| 1026 | *Acinetobacter* sp. ANC 4149 | 0 | 0.004141 | 0.020222 | 0 | Bacteria |
| 1027 | *Aquamicrobium aerolatum* | 0 | 0.004141 | 0.020222 | 0 | Bacteria |
| 1028 | *Bacillus pseudomycoides* | 0 | 0.004141 | 0.020222 | 0 | Bacteria |
| 1029 | *Chania multitudinisentens* | 0 | 0.004141 | 0.020222 | 0 | Bacteria |
| 1030 | *Labrenzia* sp. OB1 | 0 | 0.004141 | 0.020222 | 0 | Bacteria |
| 1031 | *Lautropia* sp. SCN 66-9 | 0 | 0.004141 | 0.020222 | 0 | Bacteria |
| 1032 | *Massilia yuzhufengensis* | 0 | 0.004141 | 0.020222 | 0 | Bacteria |
| 1033 | *Thiomonas intermedia* | 0 | 0.004141 | 0.020222 | 0 | Bacteria |
| 1034 | *Burkholderia cenocepacia* | 0.003372 | 0.004141 | 0 | 0.016692 | Bacteria |
| 1035 | *Eisenbergiella tayi* | 0.021915 | 0.002071 | 0 | 0 | Bacteria |
| 1036 | *Paenibacillus sabinae* | 0.001686 | 0.002071 | 0.020222 | 0 | Bacteria |
| 1037 | *Paenibacillus* sp. IHBB 10380 | 0.013486 | 0.002071 | 0 | 0.008346 | Bacteria |
| 1038 | *Chromohalobacter japonicus* | 0 | 0.002071 | 0.013482 | 0.008346 | Bacteria |
| 1039 | *Ectothiorhodospira* sp. PHS-1 | 0 | 0.002071 | 0.013482 | 0.008346 | Bacteria |
| 1040 | *Lysobacter antibioticus* | 0 | 0.002071 | 0.013482 | 0.008346 | Bacteria |
| 1041 | *Planctomyces* sp. SH-PL14 | 0 | 0.002071 | 0.013482 | 0.008346 | Bacteria |
| 1042 | *Shewanella mangrovi* | 0 | 0.002071 | 0.013482 | 0.008346 | Bacteria |
| 1043 | *Acinetobacter* sp. 1564232 | 0 | 0.010353 | 0.013482 | 0 | Bacteria |
| 1044 | *Luteimonas* sp. FCS-9 | 0 | 0.010353 | 0.013482 | 0 | Bacteria |
| 1045 | *Saccharibacillus sacchari* | 0.005057 | 0.018635 | 0 | 0 | Bacteria |
| 1046 | *Enterococcus plantarum* | 0.023601 | 0 | 0 | 0 | Bacteria |
| 1047 | *Fusobacterium nucleatum* | 0.023601 | 0 | 0 | 0 | Bacteria |
| 1048 | *Streptococcus thoraltensis* | 0.023601 | 0 | 0 | 0 | Bacteria |
| 1049 | *Bacillus* virus G | 0.023601 | 0 | 0 | 0 | Viruses |
| 1050 | *Xenorhabdus bovienii* | 0.003372 | 0 | 0.020222 | 0 | Bacteria |
| 1051 | *Chlamydia abortus* | 0.001686 | 0 | 0.013482 | 0.008346 | Bacteria |
| 1052 | *Lactobacillus jensenii* | 0.015172 | 0.008282 | 0 | 0 | Bacteria |
| 1053 | *Bacillus gaemokensis* | 0.006743 | 0.008282 | 0 | 0.008346 | Bacteria |
| 1054 | *Aquisalimonas asiatica* | 0 | 0.008282 | 0.006741 | 0.008346 | Bacteria |
| 1055 | *Lautropia* sp. SCN 70-15 | 0 | 0.008282 | 0.006741 | 0.008346 | Bacteria |
| 1056 | *Pseudomonas* sp. StFLB209 | 0 | 0.008282 | 0.006741 | 0.008346 | Bacteria |
| 1057 | *Roseovarius pacificus* | 0 | 0.016564 | 0.006741 | 0 | Bacteria |
| 1058 | *Bacillus* sp. UNC438CL73TsuS30 | 0.003372 | 0.006212 | 0.013482 | 0 | Bacteria |
| 1059 | *Listeria seeligeri* | 0.001686 | 0.006212 | 0.006741 | 0.008346 | Bacteria |
| 1060 | *Acidovorax wautersii* | 0 | 0.006212 | 0 | 0.016692 | Bacteria |
| 1061 | *Amorphus coralli* | 0 | 0.006212 | 0 | 0.016692 | Bacteria |
| 1062 | *Cellvibrio japonicus* | 0 | 0.006212 | 0 | 0.016692 | Bacteria |
| 1063 | *Idiomarina woesei* | 0 | 0.006212 | 0 | 0.016692 | Bacteria |
| 1064 | *Methylophaga lonarensis* | 0 | 0.006212 | 0 | 0.016692 | Bacteria |
| 1065 | *Methylophaga thiooxydans* | 0 | 0.006212 | 0 | 0.016692 | Bacteria |
| 1066 | *Polaromonas* sp. JS666 | 0 | 0.006212 | 0 | 0.016692 | Bacteria |
| 1067 | *Rappaport israeli* | 0 | 0.006212 | 0 | 0.016692 | Bacteria |
| 1068 | *Zoogloea* sp. LCSB751 | 0 | 0.006212 | 0 | 0.016692 | Bacteria |
| 1069 | *Curvibacter delicatus* | 0 | 0.014494 | 0 | 0.008346 | Bacteria |
| 1070 | *Halomonas xinjiangensis* | 0 | 0.014494 | 0 | 0.008346 | Bacteria |
| 1071 | *Legionella geestiana* | 0 | 0.014494 | 0 | 0.008346 | Bacteria |
| 1072 | *Leuconostoc citreum* | 0.02023 | 0.002071 | 0 | 0 | Bacteria |
| 1073 | *Lonsdalea quercina* | 0.013486 | 0.002071 | 0.006741 | 0 | Bacteria |
| 1074 | *Flavobacterium* sp. ACAM 123 | 0.006743 | 0.002071 | 0.013482 | 0 | Bacteria |
| 1075 | *Aquimarina agarilytica* | 0 | 0.002071 | 0.020222 | 0 | Bacteria |
| 1076 | *Halomonas daqiaonensis* | 0 | 0.002071 | 0.020222 | 0 | Bacteria |
| 1077 | *Halomonas* sp. Marseille-P2426 | 0 | 0.002071 | 0.020222 | 0 | Bacteria |
| 1078 | *Nitrosomonas eutropha* | 0 | 0.002071 | 0.020222 | 0 | Bacteria |
| 1079 | *Enterococcus avium* | 0.011801 | 0.010353 | 0 | 0 | Bacteria |
| 1080 | *Enterococcus hirae* | 0.021915 | 0 | 0 | 0 | Bacteria |
| 1081 | *Lactobacillus sharpeae* | 0.021915 | 0 | 0 | 0 | Bacteria |
| 1082 | *Trichococcus collinsii* | 0.021915 | 0 | 0 | 0 | Bacteria |
| 1083 | *Paenibacillus wynnii* | 0.008429 | 0 | 0.013482 | 0 | Bacteria |
| 1084 | *Alkaliphilus oremlandii* | 0.001686 | 0 | 0.020222 | 0 | Bacteria |
| 1085 | *Anaerosalibacter massiliensis* | 0.013486 | 0 | 0 | 0.008346 | Bacteria |
| 1086 | *Carnobacterium inhibens* | 0.013486 | 0 | 0 | 0.008346 | Bacteria |
| 1087 | *Enterococcus asini* | 0.013486 | 0 | 0 | 0.008346 | Bacteria |
| 1088 | *Gardnerella vaginalis* | 0.013486 | 0 | 0 | 0.008346 | Bacteria |
| 1089 | *Pseudorhodoferax* sp. Leaf265 | 0 | 0.008282 | 0.013482 | 0 | Bacteria |
| 1090 | *Xylella fastidiosa* | 0 | 0.008282 | 0.013482 | 0 | Bacteria |
| 1091 | *Halobacillus* sp. BBL2006 | 0.005057 | 0 | 0 | 0.016692 | Bacteria |
| 1092 | *Paenibacillus fonticola* | 0.005057 | 0 | 0 | 0.016692 | Bacteria |
| 1093 | *Succinatimonas* sp. CAG:777 | 0.005057 | 0 | 0 | 0.016692 | Bacteria |
| 1094 | *Enterococcus dispar* | 0.005057 | 0.016564 | 0 | 0 | Bacteria |
| 1095 | *Lactobacillus plantarum* | 0.006743 | 0.006212 | 0 | 0.008346 | Bacteria |
| 1096 | *Acinetobacter albensis* | 0 | 0.006212 | 0.006741 | 0.008346 | Bacteria |
| 1097 | *Beggiatoa leptomitiformis* | 0 | 0.014494 | 0.006741 | 0 | Bacteria |
| 1098 | *Alteromonas lipolytica* | 0 | 0.004141 | 0 | 0.016692 | Bacteria |
| 1099 | *Bradyrhizobium erythrophlei* | 0 | 0.004141 | 0 | 0.016692 | Bacteria |
| 1100 | *Cupriavidus pinatubonensis* | 0 | 0.004141 | 0 | 0.016692 | Bacteria |
| 1101 | *Halomonas cupida* | 0 | 0.004141 | 0 | 0.016692 | Bacteria |
| 1102 | *Halomonas* sp. BC1 | 0 | 0.004141 | 0 | 0.016692 | Bacteria |
| 1103 | *Herbaspirillum chlorophenolicum* | 0 | 0.004141 | 0 | 0.016692 | Bacteria |
| 1104 | *Massilia* sp. Root418 | 0 | 0.004141 | 0 | 0.016692 | Bacteria |
| 1105 | *Pseudanabaena* sp. PCC 7367 | 0 | 0.004141 | 0 | 0.016692 | Bacteria |
| 1106 | *Reinekea blandensis* | 0 | 0.004141 | 0 | 0.016692 | Bacteria |
| 1107 | *Spiribacter curvatus* | 0 | 0.004141 | 0 | 0.016692 | Bacteria |
| 1108 | *Succinatimonas hippei* | 0 | 0.004141 | 0 | 0.016692 | Bacteria |
| 1109 | *Burkholderia* sp. GAS332 | 0 | 0.012423 | 0 | 0.008346 | Bacteria |
| 1110 | *Halomonas* sp. JB380 | 0 | 0.012423 | 0 | 0.008346 | Bacteria |
| 1111 | *Pseudomonas jinjuensis* | 0 | 0.012423 | 0 | 0.008346 | Bacteria |
| 1112 | *Sphingomonas mali* | 0 | 0.012423 | 0 | 0.008346 | Bacteria |
| 1113 | *Sutterella parvirubra* | 0 | 0.012423 | 0 | 0.008346 | Bacteria |
| 1114 | *Yersinia enterocolitica* | 0 | 0.012423 | 0 | 0.008346 | Bacteria |
| 1115 | *Polaromonas* sp. OV174 | 0 | 0.020705 | 0 | 0 | Bacteria |
| 1116 | *Enterococcus gilvus* | 0.010115 | 0.002071 | 0 | 0.008346 | Bacteria |
| 1117 | *Paenibacillus dendritiformis* | 0.003372 | 0.002071 | 0.006741 | 0.008346 | Bacteria |
| 1118 | *Brachymonas denitrificans* | 0.001686 | 0.002071 | 0 | 0.016692 | Bacteria |
| 1119 | *Bacillus* sp. BT1B_CT2 | 0.001686 | 0.010353 | 0 | 0.008346 | Bacteria |
| 1120 | *Catonella morbi* | 0.02023 | 0 | 0 | 0 | Bacteria |
| 1121 | *Clostridium* sp. BL8 | 0.02023 | 0 | 0 | 0 | Bacteria |
| 1122 | *Staphylococcus pasteuri* | 0.02023 | 0 | 0 | 0 | Bacteria |
| 1123 | *Bacillus manliponensis* | 0.013486 | 0 | 0.006741 | 0 | Bacteria |
| 1124 | *Bacillus okuhidensis* | 0.005057 | 0 | 0.006741 | 0.008346 | Bacteria |
| 1125 | *Bacillus massilioanorexius* | 0.005057 | 0.008282 | 0.006741 | 0 | Bacteria |
| 1126 | *Eremococcus coleocola* | 0.005057 | 0.008282 | 0.006741 | 0 | Bacteria |
| 1127 | *Erysipelothrix tonsillarum* | 0.003372 | 0 | 0 | 0.016692 | Bacteria |
| 1128 | *Melissococcus plutonius* | 0.003372 | 0 | 0 | 0.016692 | Bacteria |
| 1129 | *Paenibacillus* sp. TI45-13ar | 0.003372 | 0 | 0 | 0.016692 | Bacteria |
| 1130 | *Risungbinella massiliensis* | 0.003372 | 0 | 0 | 0.016692 | Bacteria |
| 1131 | *Staphylococcus equorum* | 0.003372 | 0 | 0 | 0.016692 | Bacteria |
| 1132 | *Halolactibacillus* sp. JCM 19043 | 0 | 0.006212 | 0.013482 | 0 | Bacteria |
| 1133 | *Halomonas* sp. HAL1 | 0 | 0.006212 | 0.013482 | 0 | Bacteria |
| 1134 | *Paenibacillus massiliensis* | 0 | 0.006212 | 0.013482 | 0 | Bacteria |
| 1135 | *Vogesella* sp. EB | 0 | 0.006212 | 0.013482 | 0 | Bacteria |
| 1136 | *Desulfuribacillus alkaliarsenatis* | 0.005057 | 0.006212 | 0 | 0.008346 | Bacteria |
| 1137 | *Tetragenococcus halophilus* | 0.005057 | 0.006212 | 0 | 0.008346 | Bacteria |
| 1138 | *Psychrobacter piscatorii* | 0.001686 | 0.004141 | 0.013482 | 0 | Bacteria |
| 1139 | *Hydrocarboniphaga effusa* | 0 | 0.004141 | 0.006741 | 0.008346 | Bacteria |
| 1140 | *Methylomonas koyamae* | 0 | 0.004141 | 0.006741 | 0.008346 | Bacteria |
| 1141 | *Ottowia* sp. oral taxon 894 | 0 | 0.004141 | 0.006741 | 0.008346 | Bacteria |
| 1142 | *Paraburkholderia caryophylli* | 0 | 0.004141 | 0.006741 | 0.008346 | Bacteria |
| 1143 | *Photobacterium halotolerans* | 0 | 0.004141 | 0.006741 | 0.008346 | Bacteria |
| 1144 | *Pseudomonas formosensis* | 0 | 0.004141 | 0.006741 | 0.008346 | Bacteria |
| 1145 | *Chitinilyticum litopenaei* | 0 | 0.012423 | 0.006741 | 0 | Bacteria |
| 1146 | *Psychrobacter* sp. CIP 110854 | 0 | 0.012423 | 0.006741 | 0 | Bacteria |
| 1147 | *Acetobacter syzygii* | 0 | 0.010353 | 0 | 0.008346 | Bacteria |
| 1148 | *Halothece* sp. PCC 7418 | 0 | 0.010353 | 0 | 0.008346 | Bacteria |
| 1149 | *Massilia* sp. CF038 | 0 | 0.010353 | 0 | 0.008346 | Bacteria |
| 1150 | *Megasphaera* sp. MJR8396C | 0 | 0.010353 | 0 | 0.008346 | Bacteria |
| 1151 | *Pseudomonas* sp. EGD-AK9 | 0 | 0.010353 | 0 | 0.008346 | Bacteria |
| 1152 | *Salinisphaera hydrothermalis* | 0 | 0.010353 | 0 | 0.008346 | Bacteria |
| 1153 | *Sulfuricaulis limicola* | 0 | 0.010353 | 0 | 0.008346 | Bacteria |
| 1154 | *Desulfovibrio desulfuricans* | 0 | 0.018635 | 0 | 0 | Bacteria |
| 1155 | *Geobacillus* sp. C56-T3 | 0 | 0.018635 | 0 | 0 | Bacteria |
| 1156 | *Clostridium intestinale* | 0.010115 | 0 | 0 | 0.008346 | Bacteria |
| 1157 | *Enterococcus* sp. TR | 0.010115 | 0 | 0 | 0.008346 | Bacteria |
| 1158 | *Enterococcus ureasiticus* | 0.010115 | 0 | 0 | 0.008346 | Bacteria |
| 1159 | *Paenibacillus* sp. SIT18 | 0.010115 | 0 | 0 | 0.008346 | Bacteria |
| 1160 | *Paenibacillus* sp. Soil787 | 0.010115 | 0 | 0 | 0.008346 | Bacteria |
| 1161 | *Thermoflavimicrobium dichotomicum* | 0.003372 | 0 | 0.006741 | 0.008346 | Bacteria |
| 1162 | *Viridibacillus arvi* | 0.003372 | 0 | 0.006741 | 0.008346 | Bacteria |
| 1163 | *Granulicatella balaenopterae* | 0.010115 | 0.008282 | 0 | 0 | Bacteria |
| 1164 | *Bacillus horikoshii* | 0 | 0.004141 | 0.013482 | 0 | Bacteria |
| 1165 | *Enterobacter cancerogenus* | 0 | 0.004141 | 0.013482 | 0 | Bacteria |
| 1166 | *Ferrimonas senticii* | 0 | 0.004141 | 0.013482 | 0 | Bacteria |
| 1167 | *Lamprocystis purpurea* | 0 | 0.004141 | 0.013482 | 0 | Bacteria |
| 1168 | *Methylosarcina fibrata* | 0 | 0.004141 | 0.013482 | 0 | Bacteria |
| 1169 | *Nitrobacter winogradskyi* | 0 | 0.004141 | 0.013482 | 0 | Bacteria |
| 1170 | *Polynucleobacter* sp. MWH-Adler-W8 | 0 | 0.004141 | 0.013482 | 0 | Bacteria |
| 1171 | *Pseudogulbenkiania subflava* | 0 | 0.004141 | 0.013482 | 0 | Bacteria |
| 1172 | *Pseudomonas panipatensis* | 0 | 0.004141 | 0.013482 | 0 | Bacteria |
| 1173 | *Pseudomonas* sp. NBRC 111123 | 0 | 0.004141 | 0.013482 | 0 | Bacteria |
| 1174 | *Pseudorhodoferax* sp. Leaf267 | 0 | 0.004141 | 0.013482 | 0 | Bacteria |
| 1175 | *Psychrobacter* sp. Rd 27.2 | 0 | 0.004141 | 0.013482 | 0 | Bacteria |
| 1176 | *Ramlibacter* sp. Leaf400 | 0 | 0.004141 | 0.013482 | 0 | Bacteria |
| 1177 | *Roseomonas* sp. M3 | 0 | 0.004141 | 0.013482 | 0 | Bacteria |
| 1178 | *Atopobacter phocae* | 0.005057 | 0.004141 | 0 | 0.008346 | Bacteria |
| 1179 | *Bacillus* sp. FJAT-27251 | 0.006743 | 0.002071 | 0 | 0.008346 | Bacteria |
| 1180 | *Bacillus weihenstephanensis* | 0.006743 | 0.010353 | 0 | 0 | Bacteria |
| 1181 | *Roseburia inulinivorans* | 0.006743 | 0.010353 | 0 | 0 | Bacteria |
| 1182 | *Vibrio* sp. EJY3 | 0 | 0.010353 | 0.006741 | 0 | Bacteria |
| 1183 | *Anaerotruncus rubiinfantis* | 0.016858 | 0 | 0 | 0 | Bacteria |
| 1184 | *Aerococcus viridans* | 0.003372 | 0 | 0.013482 | 0 | Bacteria |
| 1185 | *Methylobacterium* sp. AMS5 | 0.003372 | 0 | 0.013482 | 0 | Bacteria |
| 1186 | *Carnobacterium iners* | 0.008429 | 0 | 0 | 0.008346 | Bacteria |
| 1187 | *Streptococcus suis* | 0.008429 | 0 | 0 | 0.008346 | Bacteria |
| 1188 | *Methanobacterium formicicum* | 0.001686 | 0 | 0.006741 | 0.008346 | Archaea |
| 1189 | *Bacillus aurantiacus* | 0.001686 | 0.008282 | 0.006741 | 0 | Bacteria |
| 1190 | *Coprococcus comes* | 0.001686 | 0.008282 | 0.006741 | 0 | Bacteria |
| 1191 | *Acytostelium subglobosum* | 0 | 0 | 0 | 0.016692 | Eukaryota |
| 1192 | *Angomonas deanei* | 0 | 0 | 0 | 0.016692 | Eukaryota |
| 1193 | *Guillardia theta* | 0 | 0 | 0 | 0.016692 | Eukaryota |
| 1194 | *Geobacillus* phage GBK2 | 0 | 0 | 0 | 0.016692 | Viruses |
| 1195 | *Aeromicrobium choanae* | 0 | 0.008282 | 0 | 0.008346 | Bacteria |
| 1196 | *Bacillus marmarensis* | 0 | 0.008282 | 0 | 0.008346 | Bacteria |
| 1197 | *Clostridium grantii* | 0 | 0.008282 | 0 | 0.008346 | Bacteria |
| 1198 | *Collimonas pratensis* | 0 | 0.008282 | 0 | 0.008346 | Bacteria |
| 1199 | *Microvirga massiliensis* | 0 | 0.008282 | 0 | 0.008346 | Bacteria |
| 1200 | *Sideroxydans lithotrophicus* | 0 | 0.008282 | 0 | 0.008346 | Bacteria |
| 1201 | *Sphaerotilus natans* | 0 | 0.008282 | 0 | 0.008346 | Bacteria |
| 1202 | *Xanthomonas cassavae* | 0 | 0.008282 | 0 | 0.008346 | Bacteria |
| 1203 | *Acinetobacter* sp. 742879 | 0 | 0.016564 | 0 | 0 | Bacteria |
| 1204 | *Burkholderia* sp. RPE67 | 0 | 0.016564 | 0 | 0 | Bacteria |
| 1205 | *Carboxydocella* sp. ULO1 | 0 | 0.016564 | 0 | 0 | Bacteria |
| 1206 | *Pedobacter* sp. Leaf41 | 0 | 0.016564 | 0 | 0 | Bacteria |
| 1207 | *Variovorax* sp. PAMC 28711 | 0 | 0.016564 | 0 | 0 | Bacteria |
| 1208 | *Lactococcus piscium* | 0.003372 | 0.006212 | 0.006741 | 0 | Bacteria |
| 1209 | *Clostridium neonatale* | 0.001686 | 0.006212 | 0 | 0.008346 | Bacteria |
| 1210 | *Haemophilus influenzae* | 0.001686 | 0.006212 | 0 | 0.008346 | Bacteria |
| 1211 | *Legionella jordanis* | 0.001686 | 0.006212 | 0 | 0.008346 | Bacteria |
| 1212 | *Planococcus* sp. L10.15 | 0.003372 | 0.004141 | 0 | 0.008346 | Bacteria |
| 1213 | *Lactobacillus ruminis* | 0.003372 | 0.012423 | 0 | 0 | Bacteria |
| 1214 | *Lactobacillus graminis* | 0.015172 | 0 | 0 | 0 | Bacteria |
| 1215 | *Lactobacillus helveticus* | 0.015172 | 0 | 0 | 0 | Bacteria |
| 1216 | *Bacillus* virus B103 | 0.015172 | 0 | 0 | 0 | Viruses |
| 1217 | *Exiguobacterium antarcticum* | 0.008429 | 0 | 0.006741 | 0 | Bacteria |
| 1218 | *Clostridium baratii* | 0.006743 | 0 | 0 | 0.008346 | Bacteria |
| 1219 | *Exiguobacterium sibiricum* | 0.006743 | 0 | 0 | 0.008346 | Bacteria |
| 1220 | *Sphaerochaeta globosa* | 0.006743 | 0.008282 | 0 | 0 | Bacteria |
| 1221 | *Acidovorax* sp. JHL-3 | 0 | 0.008282 | 0.006741 | 0 | Bacteria |
| 1222 | *Burkholderia* sp. lig30 | 0 | 0.008282 | 0.006741 | 0 | Bacteria |
| 1223 | *Collimonas fungivorans* | 0 | 0.008282 | 0.006741 | 0 | Bacteria |
| 1224 | *Gayadomonas joobiniege* | 0 | 0.008282 | 0.006741 | 0 | Bacteria |
| 1225 | *Janthinobacterium* sp. CG23_2 | 0 | 0.008282 | 0.006741 | 0 | Bacteria |
| 1226 | *Marinobacterium lutimaris* | 0 | 0.008282 | 0.006741 | 0 | Bacteria |
| 1227 | *Micromonospora rifamycinica* | 0 | 0.008282 | 0.006741 | 0 | Bacteria |
| 1228 | *Pseudoalteromonas tunicata* | 0 | 0.008282 | 0.006741 | 0 | Bacteria |
| 1229 | *Pseudoxanthomonas suwonensis* | 0 | 0.008282 | 0.006741 | 0 | Bacteria |
| 1230 | *Rhodoplanes* sp. Z2-YC6860 | 0 | 0.008282 | 0.006741 | 0 | Bacteria |
| 1231 | *Staphylococcus* sp. MB371 | 0.008429 | 0.006212 | 0 | 0 | Bacteria |
| 1232 | *Herbaspirillum frisingense* | 0.001686 | 0.006212 | 0.006741 | 0 | Bacteria |
| 1233 | *Azorhizobium doebereinerae* | 0 | 0.006212 | 0 | 0.008346 | Bacteria |
| 1234 | *Blautia obeum* | 0 | 0.006212 | 0 | 0.008346 | Bacteria |
| 1235 | *Campylobacter rectus* | 0 | 0.006212 | 0 | 0.008346 | Bacteria |
| 1236 | *Citrobacter freundii* | 0 | 0.006212 | 0 | 0.008346 | Bacteria |
| 1237 | *Comamonas aquatica* | 0 | 0.006212 | 0 | 0.008346 | Bacteria |
| 1238 | *Diaphorobacter* sp. J5-51 | 0 | 0.006212 | 0 | 0.008346 | Bacteria |
| 1239 | *Erwinia* *amylovora* | 0 | 0.006212 | 0 | 0.008346 | Bacteria |
| 1240 | *Giesbergeria anulus* | 0 | 0.006212 | 0 | 0.008346 | Bacteria |
| 1241 | *Glaciecola nitratireducens* | 0 | 0.006212 | 0 | 0.008346 | Bacteria |
| 1242 | *Lactobacillus shenzhenensis* | 0 | 0.006212 | 0 | 0.008346 | Bacteria |
| 1243 | *Leptospira kirschneri* | 0 | 0.006212 | 0 | 0.008346 | Bacteria |
| 1244 | *Methylobacter tundripaludum* | 0 | 0.006212 | 0 | 0.008346 | Bacteria |
| 1245 | *Methyloversatilis thermotolerans* | 0 | 0.006212 | 0 | 0.008346 | Bacteria |
| 1246 | *Paraburkholderia oxyphila* | 0 | 0.006212 | 0 | 0.008346 | Bacteria |
| 1247 | *Paraburkholderia sacchari* | 0 | 0.006212 | 0 | 0.008346 | Bacteria |
| 1248 | *Pseudomonas salegens* | 0 | 0.006212 | 0 | 0.008346 | Bacteria |
| 1249 | *Rhizobacter gummiphilus* | 0 | 0.006212 | 0 | 0.008346 | Bacteria |
| 1250 | *Rouxiella badensis* | 0 | 0.006212 | 0 | 0.008346 | Bacteria |
| 1251 | *Salinicoccus halodurans* | 0 | 0.006212 | 0 | 0.008346 | Bacteria |
| 1252 | *Sphingomonas sanxanigenens* | 0 | 0.006212 | 0 | 0.008346 | Bacteria |
| 1253 | *Kroppenstedtia eburnea* | 0 | 0.014494 | 0 | 0 | Bacteria |
| 1254 | *Pantoea* sp. PSNIH1 | 0 | 0.014494 | 0 | 0 | Bacteria |
| 1255 | *Rhizobium gallicum* | 0 | 0.014494 | 0 | 0 | Bacteria |
| 1256 | *Tatumella morbirosei* | 0 | 0.014494 | 0 | 0 | Bacteria |
| 1257 | *Thermobacillus composti* | 0 | 0.014494 | 0 | 0 | Bacteria |
| 1258 | *Facklamia ignava* | 0.010115 | 0.004141 | 0 | 0 | Bacteria |
| 1259 | *Acidovorax citrulli* | 0.003372 | 0.004141 | 0.006741 | 0 | Bacteria |
| 1260 | *Coprobacillus* sp. 8_1_38FAA | 0.001686 | 0.004141 | 0 | 0.008346 | Bacteria |
| 1261 | *Phascolarctobacterium* sp. CAG:207 | 0.001686 | 0.004141 | 0 | 0.008346 | Bacteria |
| 1262 | *Anaerocolumna aminovalerica* | 0.011801 | 0.002071 | 0 | 0 | Bacteria |
| 1263 | *Fictibacillus enclensis* | 0.011801 | 0.002071 | 0 | 0 | Bacteria |
| 1264 | *Salinicoccus carnicancri* | 0.011801 | 0.002071 | 0 | 0 | Bacteria |
| 1265 | *Thermoactinomyces vulgaris* | 0.011801 | 0.002071 | 0 | 0 | Bacteria |
| 1266 | *Clostridium* sp. CAG:510 | 0.003372 | 0.002071 | 0 | 0.008346 | Bacteria |
| 1267 | *Desulfotomaculum hydrothermale* | 0.003372 | 0.002071 | 0 | 0.008346 | Bacteria |
| 1268 | *Hahella ganghwensis* | 0.003372 | 0.002071 | 0 | 0.008346 | Bacteria |
| 1269 | *Aneurinibacillus aneurinilyticus* | 0.013486 | 0 | 0 | 0 | Bacteria |
| 1270 | *Enterococcus pseudoavium* | 0.013486 | 0 | 0 | 0 | Bacteria |
| 1271 | *Eubacterium limosum* | 0.013486 | 0 | 0 | 0 | Bacteria |
| 1272 | *Lactobacillus mali* | 0.013486 | 0 | 0 | 0 | Bacteria |
| 1273 | *Streptococcus didelphis* | 0.013486 | 0 | 0 | 0 | Bacteria |
| 1274 | *Streptococcus plurextorum* | 0.013486 | 0 | 0 | 0 | Bacteria |
| 1275 | *Bacteroides xylanisolvens* | 0.006743 | 0 | 0.006741 | 0 | Bacteria |
| 1276 | *Halanaerobium praevalens* | 0.006743 | 0 | 0.006741 | 0 | Bacteria |
| 1277 | *Rhizobium* phage RHEph10 | 0 | 0 | 0.013482 | 0 | Viruses |
| 1278 | *Staphylococcus* phage Twort | 0 | 0 | 0.013482 | 0 | Viruses |
| 1279 | *Alkalibacter saccharofermentans* | 0.005057 | 0 | 0 | 0.008346 | Bacteria |
| 1280 | *Bacillus soli* | 0.005057 | 0 | 0 | 0.008346 | Bacteria |
| 1281 | *Carnobacterium* sp. 17-4 | 0.005057 | 0 | 0 | 0.008346 | Bacteria |
| 1282 | *Defluviitalea phaphyphila* | 0.005057 | 0 | 0 | 0.008346 | Bacteria |
| 1283 | *Dethiosulfatibacter aminovorans* | 0.005057 | 0 | 0 | 0.008346 | Bacteria |
| 1284 | *Enterococcus* sp. RIT-PI-f | 0.005057 | 0 | 0 | 0.008346 | Bacteria |
| 1285 | *Lactobacillus xiangfangensis* | 0.005057 | 0 | 0 | 0.008346 | Bacteria |
| 1286 | *Leuconostoc mesenteroides* | 0.005057 | 0 | 0 | 0.008346 | Bacteria |
| 1287 | *Photobacterium piscicola* | 0.005057 | 0 | 0 | 0.008346 | Bacteria |
| 1288 | *Enterococcus ratti* | 0.005057 | 0.008282 | 0 | 0 | Bacteria |
| 1289 | *Acidisphaera rubrifaciens* | 0 | 0.006212 | 0.006741 | 0 | Bacteria |
| 1290 | *Aquincola tertiaricarbonis* | 0 | 0.006212 | 0.006741 | 0 | Bacteria |
| 1291 | *Azospirillum lipoferum* | 0 | 0.006212 | 0.006741 | 0 | Bacteria |
| 1292 | *Chitiniphilus shinanonensis* | 0 | 0.006212 | 0.006741 | 0 | Bacteria |
| 1293 | *Flavobacterium johnsoniae* | 0 | 0.006212 | 0.006741 | 0 | Bacteria |
| 1294 | *Fulvimarina pelagi* | 0 | 0.006212 | 0.006741 | 0 | Bacteria |
| 1295 | *Gallibacterium anatis* | 0 | 0.006212 | 0.006741 | 0 | Bacteria |
| 1296 | *Halomonas* sp. PR-M31 | 0 | 0.006212 | 0.006741 | 0 | Bacteria |
| 1297 | *Herbaspirillum* sp. TSA66 | 0 | 0.006212 | 0.006741 | 0 | Bacteria |
| 1298 | *Legionella quinlivanii* | 0 | 0.006212 | 0.006741 | 0 | Bacteria |
| 1299 | *Limnobacter* sp. MED105 | 0 | 0.006212 | 0.006741 | 0 | Bacteria |
| 1300 | *Paraburkholderia sartisoli* | 0 | 0.006212 | 0.006741 | 0 | Bacteria |
| 1301 | *Paracoccus halophilus* | 0 | 0.006212 | 0.006741 | 0 | Bacteria |
| 1302 | *Pseudaminobacter manganicus* | 0 | 0.006212 | 0.006741 | 0 | Bacteria |
| 1303 | *Pseudomonas graminis* | 0 | 0.006212 | 0.006741 | 0 | Bacteria |
| 1304 | *Pseudomonas savastanoi* | 0 | 0.006212 | 0.006741 | 0 | Bacteria |
| 1305 | *Sulfitobacter brevis* | 0 | 0.006212 | 0.006741 | 0 | Bacteria |
| 1306 | *Tepidiphilus margaritifer* | 0 | 0.006212 | 0.006741 | 0 | Bacteria |
| 1307 | *Aureimonas* sp. AU12 | 0 | 0.012423 | 0 | 0 | Bacteria |
| 1308 | *Desulfovermiculus halophilus* | 0 | 0.012423 | 0 | 0 | Bacteria |
| 1309 | *Geoalkalibacter ferrihydriticus* | 0 | 0.012423 | 0 | 0 | Bacteria |
| 1310 | *Halomonas* sp. 54_146 | 0 | 0.012423 | 0 | 0 | Bacteria |
| 1311 | *Legionella parisiensis* | 0 | 0.012423 | 0 | 0 | Bacteria |
| 1312 | *Marinobacter antarcticus* | 0 | 0.012423 | 0 | 0 | Bacteria |
| 1313 | *Novosphingobium tardaugens* | 0 | 0.012423 | 0 | 0 | Bacteria |
| 1314 | *Paenibacillus* sp. yr247 | 0 | 0.012423 | 0 | 0 | Bacteria |
| 1315 | *Bacillus vietnamensis* | 0.010115 | 0.002071 | 0 | 0 | Bacteria |
| 1316 | *Listeria aquatica* | 0.001686 | 0.010353 | 0 | 0 | Bacteria |
| 1317 | *Clostridium* sp. USBA 49 | 0.011801 | 0 | 0 | 0 | Bacteria |
| 1318 | *Ignavigranum ruoffiae* | 0.011801 | 0 | 0 | 0 | Bacteria |
| 1319 | *Lysinibacillus fusiformis* | 0.011801 | 0 | 0 | 0 | Bacteria |
| 1320 | *Youngiibacter fragilis* | 0.011801 | 0 | 0 | 0 | Bacteria |
| 1321 | *Bacillus* phage Nf | 0.011801 | 0 | 0 | 0 | Viruses |
| 1322 | *Anoxybacillus amylolyticus* | 0.003372 | 0.008282 | 0 | 0 | Bacteria |
| 1323 | *Paenibacillus popilliae* | 0.003372 | 0.008282 | 0 | 0 | Bacteria |
| 1324 | *Trichococcus pasteurii* | 0.006743 | 0.004141 | 0 | 0 | Bacteria |
| 1325 | *Bacillus* phage Mater | 0.006743 | 0.004141 | 0 | 0 | Viruses |
| 1326 | *Halapricum salinum* | 0 | 0.002071 | 0 | 0.008346 | Archaea |
| 1327 | *Albimonas donghaensis* | 0 | 0.010353 | 0 | 0 | Bacteria |
| 1328 | *Azohydromonas lata* | 0 | 0.010353 | 0 | 0 | Bacteria |
| 1329 | *Burkholderia calidae* | 0 | 0.010353 | 0 | 0 | Bacteria |
| 1330 | *Caballeronia grimmiae* | 0 | 0.010353 | 0 | 0 | Bacteria |
| 1331 | *Ferrovum* sp. PN-J185 | 0 | 0.010353 | 0 | 0 | Bacteria |
| 1332 | *Fructobacillus fructosus* | 0 | 0.010353 | 0 | 0 | Bacteria |
| 1333 | *Methyloversatilis discipulorum* | 0 | 0.010353 | 0 | 0 | Bacteria |
| 1334 | *Microbacterium esteraromaticum* | 0 | 0.010353 | 0 | 0 | Bacteria |
| 1335 | *Nitrosomonas nitrosa* | 0 | 0.010353 | 0 | 0 | Bacteria |
| 1336 | *Polynucleobacter* sp. GWA2_45_21 | 0 | 0.010353 | 0 | 0 | Bacteria |
| 1337 | *Pseudomonas psychrotolerans* | 0 | 0.010353 | 0 | 0 | Bacteria |
| 1338 | *Psychrobacter* sp. JB385 | 0 | 0.010353 | 0 | 0 | Bacteria |
| 1339 | *Ruegeria* sp. ZGT108 | 0 | 0.010353 | 0 | 0 | Bacteria |
| 1340 | *Shimia haliotis* | 0 | 0.010353 | 0 | 0 | Bacteria |
| 1341 | *Thermithiobacillus tepidarius* | 0 | 0.010353 | 0 | 0 | Bacteria |
| 1342 | *Vibrio hangzhouensis* | 0 | 0.010353 | 0 | 0 | Bacteria |
| 1343 | *Vibrio harveyi* | 0 | 0.010353 | 0 | 0 | Bacteria |
| 1344 | *Xylophilus* sp. Leaf220 | 0 | 0.010353 | 0 | 0 | Bacteria |
| 1345 | *Gonapodya prolifera* | 0 | 0.010353 | 0 | 0 | Eukaryota |
| 1346 | *Bacillus* sp. FJAT-27245 | 0.010115 | 0 | 0 | 0 | Bacteria |
| 1347 | *Geobacillus* sp. LEMMY01 | 0.010115 | 0 | 0 | 0 | Bacteria |
| 1348 | *Listeria floridensis* | 0.010115 | 0 | 0 | 0 | Bacteria |
| 1349 | *Paenibacillus beijingensis* | 0.010115 | 0 | 0 | 0 | Bacteria |
| 1350 | *Paenibacillus gorillae* | 0.010115 | 0 | 0 | 0 | Bacteria |
| 1351 | *Paenibacillus naphthalenovorans* | 0.010115 | 0 | 0 | 0 | Bacteria |
| 1352 | *Staphylococcus saprophyticus* | 0.010115 | 0 | 0 | 0 | Bacteria |
| 1353 | *Bacillus* phage CampHawk | 0.010115 | 0 | 0 | 0 | Viruses |
| 1354 | *Bacillus* virus phi29 | 0.010115 | 0 | 0 | 0 | Viruses |
| 1355 | *Lysobacter arseniciresistens* | 0.003372 | 0.006212 | 0 | 0 | Bacteria |
| 1356 | *Bacillus* phage SPG24 | 0.005057 | 0.004141 | 0 | 0 | Viruses |
| 1357 | *Dethiosulfatarculus sandiegensis* | 0.006743 | 0.002071 | 0 | 0 | Bacteria |
| 1358 | *Weissella oryzae* | 0.006743 | 0.002071 | 0 | 0 | Bacteria |
| 1359 | *Bacillus* phage Shbh1 | 0.006743 | 0.002071 | 0 | 0 | Viruses |
| 1360 | *Alkalibacterium putridalgicola* | 0.008429 | 0 | 0 | 0 | Bacteria |
| 1361 | *Alkalibacterium subtropicum* | 0.008429 | 0 | 0 | 0 | Bacteria |
| 1362 | *Anoxybacillus gonensis* | 0.008429 | 0 | 0 | 0 | Bacteria |
| 1363 | *Clostridium amylolyticum* | 0.008429 | 0 | 0 | 0 | Bacteria |
| 1364 | *Enterococcus caccae* | 0.008429 | 0 | 0 | 0 | Bacteria |
| 1365 | *Fructobacillus* sp. EFB-N1 | 0.008429 | 0 | 0 | 0 | Bacteria |
| 1366 | *Lachnobacterium* *bovis* | 0.008429 | 0 | 0 | 0 | Bacteria |
| 1367 | *Lactobacillus coryniformis* | 0.008429 | 0 | 0 | 0 | Bacteria |
| 1368 | *Paenibacillus* sp. OV219 | 0.008429 | 0 | 0 | 0 | Bacteria |
| 1369 | *Streptococcus infantis* | 0.008429 | 0 | 0 | 0 | Bacteria |
| 1370 | *Methanobrevibacter cuticularis* | 0.008429 | 0 | 0 | 0 | Archaea |
| 1371 | *Bacillus* phage MG-B1 | 0.008429 | 0 | 0 | 0 | Viruses |
| 1372 | *Halovenus aranensis* | 0 | 0 | 0 | 0.008346 | Archaea |
| 1373 | *Ignisphaera aggregans* | 0 | 0 | 0 | 0.008346 | Archaea |
| 1374 | *Methanobrevibacter filiformis* | 0 | 0 | 0 | 0.008346 | Archaea |
| 1375 | *Natrialba asiatica* | 0 | 0 | 0 | 0.008346 | Archaea |
| 1376 | *Aspergillus fumigatus* | 0 | 0 | 0 | 0.008346 | Eukaryota |
| 1377 | *Bathycoccus prasinos* | 0 | 0 | 0 | 0.008346 | Eukaryota |
| 1378 | *Choanephora cucurbitarum* | 0 | 0 | 0 | 0.008346 | Eukaryota |
| 1379 | *Claviceps purpurea* | 0 | 0 | 0 | 0.008346 | Eukaryota |
| 1380 | *Encephalitozoon cuniculi* | 0 | 0 | 0 | 0.008346 | Eukaryota |
| 1381 | *Malassezia pachydermatis* | 0 | 0 | 0 | 0.008346 | Eukaryota |
| 1382 | *Mixia osmundae* | 0 | 0 | 0 | 0.008346 | Eukaryota |
| 1383 | *Perkinsus marinus* | 0 | 0 | 0 | 0.008346 | Eukaryota |
| 1384 | *Phytophthora nicotianae* | 0 | 0 | 0 | 0.008346 | Eukaryota |
| 1385 | *Pseudocercospora musae* | 0 | 0 | 0 | 0.008346 | Eukaryota |
| 1386 | *Puccinia striiformis* | 0 | 0 | 0 | 0.008346 | Eukaryota |
| 1387 | *Rhizophagus irregularis* | 0 | 0 | 0 | 0.008346 | Eukaryota |
| 1388 | *Tetrahymena thermophila* | 0 | 0 | 0 | 0.008346 | Eukaryota |
| 1389 | *Thalassiosira pseudonana* | 0 | 0 | 0 | 0.008346 | Eukaryota |
| 1390 | *Thalassiosira weissflogii* | 0 | 0 | 0 | 0.008346 | Eukaryota |
| 1391 | *Zygosaccharomyces rouxii* | 0 | 0 | 0 | 0.008346 | Eukaryota |
| 1392 | *Delftia* phage RG-2014 | 0 | 0 | 0 | 0.008346 | Viruses |
| 1393 | *Enterococcus* phage vB_EfaP_IME199 | 0 | 0 | 0 | 0.008346 | Viruses |
| 1394 | *Erwinia* phage Ea9-2 | 0 | 0 | 0 | 0.008346 | Viruses |
| 1395 | *Escherichia* phage N4 | 0 | 0 | 0 | 0.008346 | Viruses |
| 1396 | *Ahrensia marina* | 0 | 0.008282 | 0 | 0 | Bacteria |
| 1397 | *Arthrobacter* sp. UCD-GKA | 0 | 0.008282 | 0 | 0 | Bacteria |
| 1398 | *Brevundimonas* sp. Root1279 | 0 | 0.008282 | 0 | 0 | Bacteria |
| 1399 | *Chitinimonas* *taiwanensis* | 0 | 0.008282 | 0 | 0 | Bacteria |
| 1400 | *Cupriavidus* sp. BIS7 | 0 | 0.008282 | 0 | 0 | Bacteria |
| 1401 | *Cupriavidus* sp. YR651 | 0 | 0.008282 | 0 | 0 | Bacteria |
| 1402 | *Cupriavidus taiwanensis* | 0 | 0.008282 | 0 | 0 | Bacteria |
| 1403 | *Desulfuromonas acetoxidans* | 0 | 0.008282 | 0 | 0 | Bacteria |
| 1404 | *Dokdonella immobilis* | 0 | 0.008282 | 0 | 0 | Bacteria |
| 1405 | *Halomonas chromatireducens* | 0 | 0.008282 | 0 | 0 | Bacteria |
| 1406 | *Halomonas* sp. BC04 | 0 | 0.008282 | 0 | 0 | Bacteria |
| 1407 | *Hydrocarboniphaga daqingensis* | 0 | 0.008282 | 0 | 0 | Bacteria |
| 1408 | *Kaistia adipata* | 0 | 0.008282 | 0 | 0 | Bacteria |
| 1409 | *Pandoraea sputorum* | 0 | 0.008282 | 0 | 0 | Bacteria |
| 1410 | *Pectobacterium parmentieri* | 0 | 0.008282 | 0 | 0 | Bacteria |
| 1411 | *Pseudoclavibacter faecalis* | 0 | 0.008282 | 0 | 0 | Bacteria |
| 1412 | *Pseudomonas japonica* | 0 | 0.008282 | 0 | 0 | Bacteria |
| 1413 | *Pseudomonas marincola* | 0 | 0.008282 | 0 | 0 | Bacteria |
| 1414 | *Pseudomonas saudimassiliensis* | 0 | 0.008282 | 0 | 0 | Bacteria |
| 1415 | *Psychrobacter cryohalolentis* | 0 | 0.008282 | 0 | 0 | Bacteria |
| 1416 | *Psychrobacter urativorans* | 0 | 0.008282 | 0 | 0 | Bacteria |
| 1417 | *Rhodobacter* sp. 24-YEA-8 | 0 | 0.008282 | 0 | 0 | Bacteria |
| 1418 | *Stenotrophomonas daejeonensis* | 0 | 0.008282 | 0 | 0 | Bacteria |
| 1419 | *Stenotrophomonas panacihumi* | 0 | 0.008282 | 0 | 0 | Bacteria |
| 1420 | *Syntrophorhabdus aromaticivorans* | 0 | 0.008282 | 0 | 0 | Bacteria |
| 1421 | *Thauera* sp. 28 | 0 | 0.008282 | 0 | 0 | Bacteria |
| 1422 | *Thiohalocapsa* sp. ML1 | 0 | 0.008282 | 0 | 0 | Bacteria |
| 1423 | *Thiomicrospira arctica* | 0 | 0.008282 | 0 | 0 | Bacteria |
| 1424 | *Tistlia consotensis* | 0 | 0.008282 | 0 | 0 | Bacteria |
| 1425 | *Bacillus* sp. NRRL B-14911 | 0.001686 | 0.006212 | 0 | 0 | Bacteria |
| 1426 | *Mycoplasma meleagridis* | 0.001686 | 0.006212 | 0 | 0 | Bacteria |
| 1427 | *Enterococcus silesiacus* | 0.003372 | 0.004141 | 0 | 0 | Bacteria |
| 1428 | *Desulfotomaculum aeronauticum* | 0.005057 | 0.002071 | 0 | 0 | Bacteria |
| 1429 | *Pseudobutyrivibrio xylanivorans* | 0.005057 | 0.002071 | 0 | 0 | Bacteria |
| 1430 | *Acetivibrio ethanolgignens* | 0.006743 | 0 | 0 | 0 | Bacteria |
| 1431 | *Blautia hydrogenotrophica* | 0.006743 | 0 | 0 | 0 | Bacteria |
| 1432 | *Caloramator australicus* | 0.006743 | 0 | 0 | 0 | Bacteria |
| 1433 | *Campylobacter lari* | 0.006743 | 0 | 0 | 0 | Bacteria |
| 1434 | *Carnobacterium pleistocenium* | 0.006743 | 0 | 0 | 0 | Bacteria |
| 1435 | *Celeribacter baekdonensis* | 0.006743 | 0 | 0 | 0 | Bacteria |
| 1436 | *Klebsiella* *variicola* | 0.006743 | 0 | 0 | 0 | Bacteria |
| 1437 | *Lactobacillus cacaonum* | 0.006743 | 0 | 0 | 0 | Bacteria |
| 1438 | *Lactobacillus dextrinicus* | 0.006743 | 0 | 0 | 0 | Bacteria |
| 1439 | *Leptotrichia trevisanii* | 0.006743 | 0 | 0 | 0 | Bacteria |
| 1440 | *Oenococcus oeni* | 0.006743 | 0 | 0 | 0 | Bacteria |
| 1441 | *Pedosphaera parvula* | 0.006743 | 0 | 0 | 0 | Bacteria |
| 1442 | *Ruminiclostridium* sp. KB18 | 0.006743 | 0 | 0 | 0 | Bacteria |
| 1443 | *Streptococcus* sp. 'caviae' | 0.006743 | 0 | 0 | 0 | Bacteria |
| 1444 | *Streptococcus uberis* | 0.006743 | 0 | 0 | 0 | Bacteria |
| 1445 | *Archaeoglobus fulgidus* | 0 | 0 | 0.006741 | 0 | Archaea |
| 1446 | *Methanocaldococcus* sp. FS406-22 | 0 | 0 | 0.006741 | 0 | Archaea |
| 1447 | *Angomonas desouzai* | 0 | 0 | 0.006741 | 0 | Eukaryota |
| 1448 | *Endocarpon pusillum* | 0 | 0 | 0.006741 | 0 | Eukaryota |
| 1449 | *Acinetobacter* phage IME-AB2 | 0 | 0 | 0.006741 | 0 | Viruses |
| 1450 | *Bacillus* phage SP-10 | 0.001686 | 0.002071 | 0 | 0 | Viruses |
| 1451 | *Bacillus* phage BSNPO1 | 0.003372 | 0 | 0 | 0 | Viruses |
| 1452 | *Bacillus* virus GA1 | 0.003372 | 0 | 0 | 0 | Viruses |
| 1453 | *Halococcus salifodinae* | 0 | 0.002071 | 0 | 0 | Archaea |
| 1454 | *Halolamina pelagica* | 0 | 0.002071 | 0 | 0 | Archaea |
| 1455 | *Haloprofundus marisrubri* | 0 | 0.002071 | 0 | 0 | Archaea |
| 1456 | *Methanoregula formicica* | 0 | 0.002071 | 0 | 0 | Archaea |
| 1457 | *Natrialba* sp. SSL1 | 0 | 0.002071 | 0 | 0 | Archaea |
| 1458 | *Coccomyxa subellipsoidea* | 0 | 0.002071 | 0 | 0 | Eukaryota |
| 1459 | *Coniochaeta ligniaria* | 0 | 0.002071 | 0 | 0 | Eukaryota |
| 1460 | *Dacryopinax primogenitus* | 0 | 0.002071 | 0 | 0 | Eukaryota |
| 1461 | *Pseudocohnilembus persalinus* | 0 | 0.002071 | 0 | 0 | Eukaryota |
| 1462 | *Talaromyces marneffei* | 0 | 0.002071 | 0 | 0 | Eukaryota |
| 1463 | *Halococcus hamelinensis* | 0.001686 | 0 | 0 | 0 | Archaea |
| 1464 | *Pyrococcus horikoshii* | 0.001686 | 0 | 0 | 0 | Archaea |
| 1465 | *Acanthamoeba castellanii* | 0.001686 | 0 | 0 | 0 | Eukaryota |
| 1466 | *Aspergillus calidoustus* | 0.001686 | 0 | 0 | 0 | Eukaryota |
| 1467 | *Bacillus* phage Harambe | 0.001686 | 0 | 0 | 0 | Viruses |
| 1468 | *Bacillus* phage phi3T | 0.001686 | 0 | 0 | 0 | Viruses |
| 1469 | *Bacillus* phage SPP1 | 0.001686 | 0 | 0 | 0 | Viruses |
| 1470 | *Ralstonia* phage RSP15 | 0.001686 | 0 | 0 | 0 | Viruses |
| 1471 | unclassified archaeal species | 0 | 0.002071 | 0.013482 | 0.008346 |  |
| 1472 | unclassified eukaryotic species | 0 | 0.002071 | 0 | 0 |  |
| 1473 | unclassified viral species | 0 | 0 | 0.020222 | 0 |  |
| 1474 | unclassified bacterial species | 0.087662 | 0.430669 | 1.415571 | 1.084961 |  |
