# Supplementary material for: Shotgun sequence-based metataxonomic and predictive functional profiles of Pe poke, a naturally fermented soybean food of Myanmar
Source: PLoS One. 2021 Dec 17;16(12):e0260777. doi: 10.1371/journal.pone.0260777 (PMC8682898; doi:10.1371/journal.pone.0260777)
Supplement: S5 Table — (DOCX) [file pone.0260777.s005.docx]

**Supplementary Table 5.** The overall species of *Bacillus* detected in *pe poke*

| Sl. No. | species | Occurrence (%) | | | |
| --- | --- | --- | --- | --- | --- |
|  |  | 3ds | 4ds | 5ds | Sds |
| 1 | *Bacillus thermoamylovorans* | 74.80571 | 63.3704 | 0.613414 | 4.06443 |
| 2 | *Bacillus subtilis* | 7.053389 | 7.708553 | 1.247051 | 29.44417 |
| 3 | *Bacillus smithii* | 0.251184 | 0.538336 | 0.903269 | 4.974128 |
| 4 | *Bacillus coagulans* | 2.830459 | 0.565253 | 0.869565 | 1.952929 |
| 5 | *Bacillus glycinifermentans* | 0 | 2.726877 | 0 | 0.175263 |
| 6 | *Bacillus licheniformis* | 0.986193 | 0.592169 | 0.397708 | 0.108496 |
| 7 | *Bacillus andreraoultii* | 0.620375 | 0.703977 | 0.107853 | 0.216992 |
| 8 | *Bacillus fordii* | 0.062375 | 0.074539 | 0.653859 | 0.267067 |
| 9 | *Bacillus* sp. OxB-1 | 0.006743 | 0.043481 | 0.566229 | 0.300451 |
| 10 | *Bacillus cereus* | 0.301758 | 0.213264 | 0.121335 | 0.191955 |
| 11 | *Bacillus* sp. VT-16-64 | 0.057317 | 0.03934 | 0.337041 | 0.292105 |
| 12 | *Bacillus methanolicus* | 0.025287 | 0.043481 | 0.397708 | 0.083459 |
| 13 | *Bacillus megaterium* | 0.033716 | 0.024846 | 0.195484 | 0.292105 |
| 14 | *Bacillus alveayuensis* | 0.10452 | 0.033128 | 0.276373 | 0.050075 |
| 15 | *Bacillus eiseniae* | 0.001686 | 0.004141 | 0.364004 | 0.041729 |
| 16 | *Bacillus amyloliquefaciens* | 0.097776 | 0.070398 | 0.020222 | 0.158571 |
| 17 | *Bacillus* sp. X1(2014) | 0.075861 | 0.031058 | 0.202224 | 0.008346 |
| 18 | *Bacillus farraginis* | 0.187124 | 0.097315 | 0.006741 | 0 |
| 19 | *Bacillus sporothermodurans* | 0.067432 | 0.014494 | 0.175261 | 0.033383 |
| 20 | *Bacillus acidiproducens* | 0.121378 | 0.093173 | 0.013482 | 0.058421 |
| 21 | *Bacillus galactosidilyticus* | 0.003372 | 0.014494 | 0.155039 | 0.066767 |
| 22 | *Bacillus niameyensis* | 0.008429 | 0.033128 | 0.020222 | 0.166917 |
| 23 | *Bacillus halodurans* | 0.175323 | 0.002071 | 0.006741 | 0.041729 |
| 24 | *Bacillus lentus* | 0.001686 | 0.004141 | 0.067408 | 0.133534 |
| 25 | *Bacillus cohnii* | 0.016858 | 0 | 0.067408 | 0.116842 |
| 26 | *Bacillus pseudofirmus* | 0.072489 | 0.018635 | 0.040445 | 0.058421 |
| 27 | *Bacillus shackletonii* | 0.026973 | 0.051763 | 0.020222 | 0.066767 |
| 28 | *Bacillus* sp. B14905 | 0.008429 | 0.060045 | 0.013482 | 0.075113 |
| 29 | *Bacillus stratosphericus* | 0 | 0 | 0.08089 | 0.075113 |
| 30 | *Bacillus timonensis* | 0.006743 | 0.002071 | 0.033704 | 0.108496 |
| 31 | *Bacillus pumilus* | 0.023601 | 0.010353 | 0.074149 | 0.033383 |
| 32 | *Bacillus* sp. OK048 | 0.018544 | 0.012423 | 0.047186 | 0.058421 |
| 33 | *Bacillus gottheilii* | 0.006743 | 0.002071 | 0.026963 | 0.10015 |
| 34 | *Bacillus azotoformans* | 0.008429 | 0.033128 | 0.040445 | 0.050075 |
| 35 | *Bacillus gobiensis* | 0.030344 | 0 | 0 | 0.10015 |
| 36 | *Bacillus shacheensis* | 0.011801 | 0.010353 | 0.047186 | 0.058421 |
| 37 | *Bacillus badius* | 0.003372 | 0.020705 | 0.020222 | 0.083459 |
| 38 | *Bacillus* sp. FJAT-29814 | 0.026973 | 0.045551 | 0.013482 | 0.041729 |
| 39 | *Bacillus rubiinfantis* | 0.042145 | 0.020705 | 0.020222 | 0.041729 |
| 40 | *Bacillus sonorensis* | 0.057317 | 0.037269 | 0.013482 | 0.016692 |
| 41 | *Bacillus* sp. TH008 | 0.038773 | 0.084891 | 0 | 0 |
| 42 | *Bacillus thuringiensis* | 0.023601 | 0.037269 | 0.006741 | 0.050075 |
| 43 | *Bacillus aquimaris* | 0.018544 | 0.033128 | 0.013482 | 0.050075 |
| 44 | *Bacillus tuaregi* | 0.038773 | 0.016564 | 0 | 0.058421 |
| 45 | *Bacillus caseinilyticus* | 0.015172 | 0.016564 | 0.006741 | 0.075113 |
| 46 | *Bacillus dakarensis* | 0 | 0.006212 | 0.020222 | 0.083459 |
| 47 | *Bacillus plakortidis* | 0 | 0 | 0.107853 | 0 |
| 48 | *Bacillus cellulosilyticus* | 0.011801 | 0.004141 | 0 | 0.091804 |
| 49 | *Bacillus niacini* | 0.001686 | 0.006212 | 0.006741 | 0.091804 |
| 50 | *Bacillus ginsengihumi* | 0.005057 | 0.006212 | 0.026963 | 0.066767 |
| 51 | *Bacillus flexus* | 0.011801 | 0.002071 | 0.074149 | 0.016692 |
| 52 | *Bacillus circulans* | 0.016858 | 0 | 0.060667 | 0.025038 |
| 53 | *Bacillus solani* | 0.005057 | 0.006212 | 0.08089 | 0.008346 |
| 54 | *Bacillus altitudinis* | 0.042145 | 0.053834 | 0 | 0 |
| 55 | *Bacillus ligniniphilus* | 0.011801 | 0.018635 | 0.006741 | 0.058421 |
| 56 | *Bacillus* sp. FJAT-27445 | 0.006743 | 0.004141 | 0.067408 | 0.016692 |
| 57 | *Bacillus bogoriensis* | 0.005057 | 0.004141 | 0.026963 | 0.058421 |
| 58 | *Bacillus* sp. Marseille-P2366 | 0.043831 | 0.016564 | 0 | 0.033383 |
| 59 | *Bacillus* sp. MB2021 | 0.028659 | 0.022776 | 0.033704 | 0.008346 |
| 60 | *Bacillus* sp. FJAT-27225 | 0.006743 | 0.010353 | 0 | 0.075113 |
| 61 | *Bacillus* sp. MKU004 | 0.06406 | 0.014494 | 0.013482 | 0 |
| 62 | *Bacillus marisflavi* | 0.018544 | 0.004141 | 0.026963 | 0.041729 |
| 63 | *Bacillus mojavensis* | 0.048888 | 0.006212 | 0.006741 | 0.025038 |
| 64 | *Bacillus massiliosenegalensis* | 0.015172 | 0.031058 | 0.026963 | 0.008346 |
| 65 | *Bacillus clausii* | 0.021915 | 0.012423 | 0.013482 | 0.033383 |
| 66 | *Bacillus fastidiosus* | 0.013486 | 0.004141 | 0.013482 | 0.050075 |
| 67 | *Bacillus mesonae* | 0.013486 | 0.008282 | 0.033704 | 0.025038 |
| 68 | *Bacillus salsus* | 0.013486 | 0.002071 | 0.013482 | 0.050075 |
| 69 | *Bacillus pseudalcaliphilus* | 0 | 0.010353 | 0.026963 | 0.041729 |
| 70 | *Bacillus bataviensis* | 0.016858 | 0.006212 | 0.047186 | 0.008346 |
| 71 | *Bacillus firmus* | 0.015172 | 0.012423 | 0.033704 | 0.016692 |
| 72 | *Bacillus halmapalus* | 0 | 0.004141 | 0.006741 | 0.066767 |
| 73 | *Bacillus okhensis* | 0.003372 | 0.002071 | 0.013482 | 0.058421 |
| 74 | *Bacillus paralicheniformis* | 0.053946 | 0.016564 | 0.006741 | 0 |
| 75 | *Bacillus* sp. SJS | 0.001686 | 0 | 0.006741 | 0.066767 |
| 76 | *Bacillus fumarioli* | 0.018544 | 0.026917 | 0.020222 | 0.008346 |
| 77 | *Bacillus koreensis* | 0.015172 | 0.008282 | 0 | 0.050075 |
| 78 | *Bacillus massiliogorillae* | 0.006743 | 0.002071 | 0.013482 | 0.050075 |
| 79 | *Bacillus simplex* | 0.006743 | 0.006212 | 0 | 0.058421 |
| 80 | *Bacillus* sp. FJAT-14578 | 0 | 0.004141 | 0 | 0.066767 |
| 81 | *Bacillus oceanisediminis* | 0.011801 | 0.004141 | 0.020222 | 0.033383 |
| 82 | *Bacillus alcalophilus* | 0.010115 | 0.004141 | 0.013482 | 0.041729 |
| 83 | *Bacillus krulwichiae* | 0.010115 | 0.002071 | 0.040445 | 0.016692 |
| 84 | *Bacillus endophyticus* | 0.001686 | 0.002071 | 0.006741 | 0.058421 |
| 85 | *Bacillus persicus* | 0.001686 | 0.014494 | 0.026963 | 0.025038 |
| 86 | *Bacillus wakoensis* | 0.003372 | 0.010353 | 0.020222 | 0.033383 |
| 87 | *Bacillus* sp. Marseille-P2384 | 0.025287 | 0.016564 | 0 | 0.025038 |
| 88 | *Bacillus* sp. NC2-31 | 0.021915 | 0.004141 | 0.006741 | 0.033383 |
| 89 | *Bacillus* sp. LL01 | 0.001686 | 0.002071 | 0.053927 | 0.008346 |
| 90 | *Bacillus* sp. EB01 | 0.016858 | 0.022776 | 0.006741 | 0.016692 |
| 91 | *Bacillus velezensis* | 0.015172 | 0.002071 | 0.020222 | 0.025038 |
| 92 | *Bacillus subterraneus* | 0.001686 | 0.002071 | 0.033704 | 0.025038 |
| 93 | *Bacillus* sp. UNC41MFS5 | 0.006743 | 0.010353 | 0.020222 | 0.025038 |
| 94 | *Bacillus ndiopicus* | 0.001686 | 0 | 0.026963 | 0.033383 |
| 95 | *Bacillus* sp. FJAT-27986 | 0.001686 | 0 | 0.026963 | 0.033383 |
| 96 | *Bacillus* sp. MRMR6 | 0 | 0.002071 | 0 | 0.058421 |
| 97 | *Bacillus humi* | 0.02023 | 0.004141 | 0 | 0.033383 |
| 98 | *Bacillus* sp. HMSC76G11 | 0.006743 | 0 | 0 | 0.050075 |
| 99 | *Bacillus vireti* | 0.006743 | 0.004141 | 0.020222 | 0.025038 |
| 100 | *Bacillus weihaiensis* | 0.005057 | 0.004141 | 0.020222 | 0.025038 |
| 101 | *Bacillus anthracis* | 0.005057 | 0.012423 | 0.020222 | 0.016692 |
| 102 | *Bacillus aryabhattai* | 0.011801 | 0 | 0 | 0.041729 |
| 103 | *Bacillus dielmoensis* | 0.038773 | 0.006212 | 0 | 0.008346 |
| 104 | *Bacillus acidicola* | 0.038773 | 0.014494 | 0 | 0 |
| 105 | *Bacillus* sp. FJAT-25496 | 0.001686 | 0.004141 | 0.013482 | 0.033383 |
| 106 | *Bacillus psychrosaccharolyticus* | 0.001686 | 0.002071 | 0.006741 | 0.041729 |
| 107 | *Bacillus alkalitelluris* | 0.006743 | 0.002071 | 0 | 0.041729 |
| 108 | *Bacillus* sp. LF1 | 0.015172 | 0.004141 | 0.013482 | 0.016692 |
| 109 | *Bacillus panaciterrae* | 0.005057 | 0.012423 | 0.006741 | 0.025038 |
| 110 | *Bacillus enclensis* | 0 | 0.002071 | 0.047186 | 0 |
| 111 | *Bacillus lonarensis* | 0.006743 | 0.026917 | 0.013482 | 0 |
| 112 | *Bacillus horneckiae* | 0.016858 | 0.004141 | 0 | 0.025038 |
| 113 | *Bacillus* sp. MUM 116 | 0.016858 | 0.012423 | 0 | 0.016692 |
| 114 | *Bacillus novalis* | 0.001686 | 0.018635 | 0 | 0.025038 |
| 115 | *Bacillus sinesaloumensis* | 0.005057 | 0.008282 | 0.006741 | 0.025038 |
| 116 | *Bacillus* sp. FJAT-22058 | 0.011801 | 0.024846 | 0 | 0.008346 |
| 117 | *Bacillus mycoides* | 0.001686 | 0.014494 | 0.020222 | 0.008346 |
| 118 | *Bacillus* sp. SA1-12 | 0.001686 | 0.004141 | 0.013482 | 0.025038 |
| 119 | *Bacillus* sp. 1NLA3E | 0 | 0.010353 | 0 | 0.033383 |
| 120 | *Bacillus* sp. ES3 | 0.003372 | 0.006212 | 0 | 0.033383 |
| 121 | *Bacillus* sp. NRRL B-41327 | 0.015172 | 0.026917 | 0 | 0 |
| 122 | *Bacillus manliponensis* | 0 | 0 | 0 | 0.041729 |
| 123 | *Bacillus* sp. FJAT-44921 | 0.008429 | 0.006212 | 0 | 0.025038 |
| 124 | *Bacillus* sp. SG-1 | 0 | 0.006212 | 0 | 0.033383 |
| 125 | *Bacillus* sp. 491mf | 0.010115 | 0.028987 | 0 | 0 |
| 126 | *Bacillus cytotoxicus* | 0.011801 | 0.018635 | 0 | 0.008346 |
| 127 | *Bacillus oleronius* | 0.001686 | 0.006212 | 0.013482 | 0.016692 |
| 128 | *Bacillus testis* | 0.001686 | 0.006212 | 0.013482 | 0.016692 |
| 129 | *Bacillus safensis* | 0.001686 | 0.002071 | 0 | 0.033383 |
| 130 | *Bacillus nakamurai* | 0.016858 | 0.004141 | 0.006741 | 0.008346 |
| 131 | *Bacillus* sp. Soil768D1 | 0 | 0 | 0.026963 | 0.008346 |
| 132 | *Bacillus cihuensis* | 0.001686 | 0 | 0 | 0.033383 |
| 133 | *Bacillus nealsonii* | 0.005057 | 0.006212 | 0.006741 | 0.016692 |
| 134 | *Bacillus* sp. J33 | 0.006743 | 0.002071 | 0 | 0.025038 |
| 135 | *Bacillus aidingensis* | 0 | 0.002071 | 0.006741 | 0.025038 |
| 136 | *Bacillus hemicellulosilyticus* | 0 | 0.002071 | 0.006741 | 0.025038 |
| 137 | *Bacillus daliensis* | 0.008429 | 0 | 0 | 0.025038 |
| 138 | *Bacillus cecembensis* | 0 | 0 | 0 | 0.033383 |
| 139 | *Bacillus oryziterrae* | 0 | 0 | 0 | 0.033383 |
| 140 | *Bacillus* sp. FJAT-18017 | 0 | 0 | 0 | 0.033383 |
| 141 | *Bacillus* sp. URHB0009 | 0.001686 | 0.022776 | 0 | 0.008346 |
| 142 | *Bacillus* sp. FJAT-27916 | 0.003372 | 0.004141 | 0 | 0.025038 |
| 143 | *Bacillus chagannorensis* | 0.005057 | 0.002071 | 0 | 0.025038 |
| 144 | *Bacillus kribbensis* | 0.006743 | 0 | 0 | 0.025038 |
| 145 | *Bacillus encimensis* | 0 | 0.004141 | 0.026963 | 0 |
| 146 | *Bacillus* sp. FJAT-29937 | 0 | 0.002071 | 0.020222 | 0.008346 |
| 147 | *Bacillus* sp. 522_BSPC | 0.010115 | 0 | 0.020222 | 0 |
| 148 | *Bacillus coahuilensis* | 0 | 0 | 0.013482 | 0.016692 |
| 149 | *Bacillus* sp. FJAT-25547 | 0 | 0.006212 | 0.006741 | 0.016692 |
| 150 | *Bacillus* sp. FJAT-27997 | 0 | 0.002071 | 0 | 0.025038 |
| 151 | *Bacillus jeotgali* | 0.003372 | 0 | 0.006741 | 0.016692 |
| 152 | *Bacillus* sp. JCM 19034 | 0.003372 | 0 | 0.006741 | 0.016692 |
| 153 | *Bacillus indicus* | 0.001686 | 0 | 0 | 0.025038 |
| 154 | *Bacillus* sp. J37 | 0.001686 | 0.002071 | 0.013482 | 0.008346 |
| 155 | *Bacillus solimangrovi* | 0.001686 | 0 | 0.006741 | 0.016692 |
| 156 | *Bacillus* sp. CDB3 | 0.018544 | 0.006212 | 0 | 0 |
| 157 | *Bacillus pseudomycoides* | 0 | 0.004141 | 0.020222 | 0 |
| 158 | *Bacillus gaemokensis* | 0.006743 | 0.008282 | 0 | 0.008346 |
| 159 | *Bacillus* sp. UNC438CL73TsuS30 | 0.003372 | 0.006212 | 0.013482 | 0 |
| 160 | *Bacillus* sp. BT1B_CT2 | 0.001686 | 0.010353 | 0 | 0.008346 |
| 161 | *Bacillus manliponensis* | 0.013486 | 0 | 0.006741 | 0 |
| 162 | *Bacillus okuhidensis* | 0.005057 | 0 | 0.006741 | 0.008346 |
| 163 | *Bacillus massilioanorexius* | 0.005057 | 0.008282 | 0.006741 | 0 |
| 164 | *Bacillus horikoshii* | 0 | 0.004141 | 0.013482 | 0 |
| 165 | *Bacillus* sp. FJAT-27251 | 0.006743 | 0.002071 | 0 | 0.008346 |
| 166 | *Bacillus weihenstephanensis* | 0.006743 | 0.010353 | 0 | 0 |
| 167 | *Bacillus aurantiacus* | 0.001686 | 0.008282 | 0.006741 | 0 |
| 168 | *Bacillus marmarensis* | 0 | 0.008282 | 0 | 0.008346 |
| 169 | *Bacillus soli* | 0.005057 | 0 | 0 | 0.008346 |
| 170 | *Bacillus vietnamensis* | 0.010115 | 0.002071 | 0 | 0 |
| 171 | *Bacillus* sp. FJAT-27245 | 0.010115 | 0 | 0 | 0 |
| 172 | *Bacillus* sp. NRRL B-14911 | 0.001686 | 0.006212 | 0 | 0 |
