# Supplementary material for: Shotgun sequence-based metataxonomic and predictive functional profiles of Pe poke, a naturally fermented soybean food of Myanmar
Source: PLoS One. 2021 Dec 17;16(12):e0260777. doi: 10.1371/journal.pone.0260777 (PMC8682898; doi:10.1371/journal.pone.0260777)
Supplement: S6 Table — (DOCX) [file pone.0260777.s006.docx]

**Supplementary Table 6.** The species of lactic acid bacteria detected in *pe poke*

| Sl. No. | Lactic acid bacteria | Occurrence (%) | | | |
| --- | --- | --- | --- | --- | --- |
|  |  | 3ds | 4ds | 5ds | Sds |
| 1 | *Vagococcus lutrae* | 0.335474 | 0.942088 | 0.013482 | 0.091804 |
| 2 | *Vagococcus fluvialis* | 0.787269 | 0 | 0 | 0.400601 |
| 3 | *Enterococcus faecalis* | 0.446737 | 0.219475 | 0.047186 | 0.075113 |
| 4 | *Enterococcus faecium* | 0.094405 | 0.509348 | 0.006741 | 0.091804 |
| 5 | *Carnobacterium maltaromaticum* | 0.183752 | 0.074539 | 0.026963 | 0.10015 |
| 6 | *Vagococcus penaei* | 0.170266 | 0 | 0 | 0.091804 |
| 7 | *Streptococcus pneumoniae* | 0.037088 | 0.024846 | 0 | 0.066767 |
| 8 | *Streptococcus agalactiae* | 0.016858 | 0.016564 | 0 | 0.083459 |
| 9 | *Enterococcus pallens* | 0.072489 | 0.002071 | 0.006741 | 0.033383 |
| 10 | *Enterococcus phoeniculicola* | 0.075861 | 0.002071 | 0.006741 | 0.025038 |
| 11 | *Streptococcus anginosus* | 0.079233 | 0.022776 | 0.006741 | 0 |
| 12 | *Enterococcus durans* | 0.005057 | 0.053834 | 0.033704 | 0.008346 |
| 13 | *Vagococcus* sp. D7T301 | 0.069118 | 0 | 0 | 0.025038 |
| 14 | *Enterococcus saccharolyticus* | 0.025287 | 0 | 0.020222 | 0.041729 |
| 15 | *Carnobacterium viridans* | 0.062375 | 0 | 0 | 0.008346 |
| 16 | *Carnobacterium* sp. CP1 | 0.050574 | 0 | 0 | 0.016692 |
| 17 | *Enterococcus gallinarum* | 0.055631 | 0.002071 | 0 | 0.008346 |
| 18 | *Enterococcus casseliflavus* | 0.047202 | 0 | 0 | 0.016692 |
| 19 | *Vagococcus teuberi* | 0.043831 | 0.002071 | 0 | 0.016692 |
| 20 | *Enterococcus columbae* | 0.057317 | 0.002071 | 0 | 0 |
| 21 | *Lactobacillus fermentum* | 0 | 0.002071 | 0.013482 | 0.041729 |
| 22 | *Enterococcus aquimarinus* | 0.028659 | 0.010353 | 0 | 0.016692 |
| 23 | *Lactobacillus rhamnosus* | 0.010115 | 0.022776 | 0.013482 | 0.008346 |
| 24 | *Streptococcus salivarius* | 0.02023 | 0.022776 | 0 | 0.008346 |
| 25 | *Enterococcus mundtii* | 0.025287 | 0.002071 | 0.006741 | 0.016692 |
| 26 | *Enterococcus canintestini* | 0.023601 | 0 | 0.026963 | 0 |
| 27 | *Enterococcus sulfureus* | 0.02023 | 0 | 0.013482 | 0.016692 |
| 28 | *Lactobacillus curvatus* | 0.043831 | 0.004141 | 0 | 0 |
| 29 | *Enterococcus cecorum* | 0.030344 | 0.016564 | 0 | 0 |
| 30 | *Pediococcus acidilactici* | 0 | 0.002071 | 0 | 0.041729 |
| 31 | *Enterococcus massiliensis* | 0.003372 | 0.012423 | 0 | 0.025038 |
| 32 | *Enterococcus termitis* | 0.040459 | 0 | 0 | 0 |
| 33 | *Enterococcus rivorum* | 0.023601 | 0 | 0 | 0.016692 |
| 34 | *Lactobacillus salivarius* | 0.006743 | 0.008282 | 0 | 0.025038 |
| 35 | *Tetragenococcus muriaticus* | 0.003372 | 0.002071 | 0 | 0.033383 |
| 36 | *Lactobacillus algidus* | 0.005057 | 0 | 0 | 0.033383 |
| 37 | *Streptococcus pyogenes* | 0.021915 | 0 | 0.006741 | 0.008346 |
| 38 | *Carnobacterium* sp. AT7 | 0.028659 | 0 | 0.006741 | 0 |
| 39 | *Lactococcus lactis* | 0.013486 | 0.004141 | 0.006741 | 0.008346 |
| 40 | *Enterococcus malodoratus* | 0.023601 | 0 | 0 | 0.008346 |
| 41 | *Carnobacterium gallinarum* | 0.015172 | 0 | 0 | 0.016692 |
| 42 | *Enterococcus canis* | 0.021915 | 0 | 0 | 0.008346 |
| 43 | *Lactobacillus casei* | 0.010115 | 0.010353 | 0 | 0.008346 |
| 44 | *Streptococcus gordonii* | 0.010115 | 0.010353 | 0 | 0.008346 |
| 45 | *Enterococcus thailandicus* | 0.02023 | 0 | 0 | 0.008346 |
| 46 | *Enterococcus devriesei* | 0.025287 | 0.002071 | 0 | 0 |
| 47 | *Carnobacterium divergens* | 0.026973 | 0 | 0 | 0 |
| 48 | *Carnobacterium mobile* | 0.013486 | 0 | 0.013482 | 0 |
| 49 | *Enterococcus ureilyticus* | 0.018544 | 0 | 0 | 0.008346 |
| 50 | *Lactobacillus parabrevis* | 0 | 0.004141 | 0.013482 | 0.008346 |
| 51 | *Streptococcus mitis* | 0.025287 | 0 | 0 | 0 |
| 52 | *Enterococcus plantarum* | 0.023601 | 0 | 0 | 0 |
| 53 | *Streptococcus thoraltensis* | 0.023601 | 0 | 0 | 0 |
| 54 | *Lactobacillus jensenii* | 0.015172 | 0.008282 | 0 | 0 |
| 55 | *Leuconostoc citreum* | 0.02023 | 0.002071 | 0 | 0 |
| 56 | *Enterococcus avium* | 0.011801 | 0.010353 | 0 | 0 |
| 57 | *Enterococcus hirae* | 0.021915 | 0 | 0 | 0 |
| 58 | *Lactobacillus sharpeae* | 0.021915 | 0 | 0 | 0 |
| 59 | *Carnobacterium inhibens* | 0.013486 | 0 | 0 | 0.008346 |
| 60 | *Enterococcus asini* | 0.013486 | 0 | 0 | 0.008346 |
| 61 | *Enterococcus dispar* | 0.005057 | 0.016564 | 0 | 0 |
| 62 | *Lactobacillus plantarum* | 0.006743 | 0.006212 | 0 | 0.008346 |
| 63 | *Enterococcus gilvus* | 0.010115 | 0.002071 | 0 | 0.008346 |
| 64 | *Eremococcus coleocola* | 0.005057 | 0.008282 | 0.006741 | 0 |
| 65 | *Tetragenococcus halophilus* | 0.005057 | 0.006212 | 0 | 0.008346 |
| 66 | *Enterococcus* sp. TR | 0.010115 | 0 | 0 | 0.008346 |
| 67 | *Enterococcus ureasiticus* | 0.010115 | 0 | 0 | 0.008346 |
| 68 | *Aerococcus viridans* | 0.003372 | 0 | 0.013482 | 0 |
| 69 | *Carnobacterium iners* | 0.008429 | 0 | 0 | 0.008346 |
| 70 | *Streptococcus suis* | 0.008429 | 0 | 0 | 0.008346 |
| 71 | *Lactococcus piscium* | 0.003372 | 0.006212 | 0.006741 | 0 |
| 72 | *Lactobacillus ruminis* | 0.003372 | 0.012423 | 0 | 0 |
| 73 | *Lactobacillus graminis* | 0.015172 | 0 | 0 | 0 |
| 74 | *Lactobacillus helveticus* | 0.015172 | 0 | 0 | 0 |
| 75 | *Lactobacillus shenzhenensis* | 0 | 0.006212 | 0 | 0.008346 |
| 76 | *Enterococcus pseudoavium* | 0.013486 | 0 | 0 | 0 |
| 77 | *Lactobacillus mali* | 0.013486 | 0 | 0 | 0 |
| 78 | *Streptococcus didelphis* | 0.013486 | 0 | 0 | 0 |
| 79 | *Streptococcus plurextorum* | 0.013486 | 0 | 0 | 0 |
| 80 | *Carnobacterium* sp. 17-4 | 0.005057 | 0 | 0 | 0.008346 |
| 81 | *Enterococcus* sp. RIT-PI-f | 0.005057 | 0 | 0 | 0.008346 |
| 82 | *Lactobacillus xiangfangensis* | 0.005057 | 0 | 0 | 0.008346 |
| 83 | *Leuconostoc mesenteroides* | 0.005057 | 0 | 0 | 0.008346 |
| 84 | *Enterococcus ratti* | 0.005057 | 0.008282 | 0 | 0 |
| 85 | *Weissella oryzae* | 0.006743 | 0.002071 | 0 | 0 |
| 86 | *Enterococcus caccae* | 0.008429 | 0 | 0 | 0 |
| 87 | *Lactobacillus coryniformis* | 0.008429 | 0 | 0 | 0 |
| 88 | *Streptococcus infantis* | 0.008429 | 0 | 0 | 0 |
| 89 | *Enterococcus silesiacus* | 0.003372 | 0.004141 | 0 | 0 |
| 90 | *Carnobacterium pleistocenium* | 0.006743 | 0 | 0 | 0 |
| 91 | *Lactobacillus cacaonum* | 0.006743 | 0 | 0 | 0 |
| 92 | *Lactobacillus dextrinicus* | 0.006743 | 0 | 0 | 0 |
| 93 | *Oenococcus oeni* | 0.006743 | 0 | 0 | 0 |
| 94 | *Streptococcus* sp. 'caviae' | 0.006743 | 0 | 0 | 0 |
