# Supplementary material for: Shotgun sequence-based metataxonomic and predictive functional profiles of Pe poke, a naturally fermented soybean food of Myanmar
Source: PLoS One. 2021 Dec 17;16(12):e0260777. doi: 10.1371/journal.pone.0260777 (PMC8682898; doi:10.1371/journal.pone.0260777)
Supplement: S7 Table — (DOCX) [file pone.0260777.s007.docx]

**Supplementary Table 7.** Archaeal species detected in samples of *pe poke.*

| Sl. No. | Species | Relative abundance (%) | | | |
| --- | --- | --- | --- | --- | --- |
|  |  | 3ds | 4ds | 5ds | Sds |
| 1 | *Archaeoglobus fulgidus* | 0 | 0 | 0.006741 | 0 |
| 2 | *Halapricum salinum* | 0 | 0.001842 | 0 | 0.008346 |
| 3 | *Halococcus hamelinensis* | 0.001686 | 0 | 0 | 0 |
| 4 | *Halococcus salifodinae* | 0 | 0.001842 | 0 | 0 |
| 5 | *Halolamina pelagica* | 0 | 0.001842 | 0 | 0 |
| 6 | *Haloprofundus marisrubri* | 0 | 0.001842 | 0 | 0 |
| 7 | *Halovenus aranensis* | 0 | 0 | 0 | 0.008346 |
| 8 | *Ignisphaera aggregans* | 0 | 0 | 0 | 0.008346 |
| 9 | *Methanobacterium formicicum* | 0.001686 | 0 | 0.006741 | 0.008346 |
| 10 | *Methanobrevibacter cuticularis* | 0.008429 | 0 | 0 | 0 |
| 11 | *Methanobrevibacter filiformis* | 0 | 0 | 0 | 0.008346 |
| 12 | *Methanocaldococcus* sp. FS406-22 | 0 | 0 | 0.006741 | 0 |
| 13 | *Methanoregula formicica* | 0 | 0.001842 | 0 | 0 |
| 14 | *Natrialba asiatica* | 0 | 0 | 0 | 0.008346 |
| 15 | *Natrialba* sp. SSL1 | 0 | 0.001842 | 0 | 0 |
| 16 | *Pyrococcus horikoshii* | 0.001686 | 0 | 0 | 0 |
| 17 | unclassified archaeal species | 0 | 0.001842 | 0.013482 | 0.008346 |
