# Supplementary material for: Shotgun sequence-based metataxonomic and predictive functional profiles of Pe poke, a naturally fermented soybean food of Myanmar
Source: PLoS One. 2021 Dec 17;16(12):e0260777. doi: 10.1371/journal.pone.0260777 (PMC8682898; doi:10.1371/journal.pone.0260777)
Supplement: S8 Table — (DOCX) [file pone.0260777.s008.docx]

**Supplementary Table 8.** Eukaryotic species including yeasts, moulds, algae, protozoa and parasites detected in *pe poke*

| Sl. No. | Species | Relative abundance (%) | | | |
| --- | --- | --- | --- | --- | --- |
|  |  | 3ds | 4ds | 5ds | Sds |
| **Yeasts** | | | | | |
| 1 | *Malassezia pachydermatis* | 0 | 0 | 0 | 0.008346 |
| 2 | *Zygosaccharomyces rouxii* | 0 | 0 | 0 | 0.008346 |
| 3 | *Coniochaeta ligniaria* | 0 | 0.001842 | 0 | 0 |
| **Molds** | | | | | |
| 1 | *Batrachochytrium dendrobatidis* | 0.005057 | 0 | 0 | 0.133534 |
| 2 | *Mucor ambiguus* | 0.001686 | 0.003683 | 0.020222 | 0.008346 |
| 3 | *Gonapodya prolifera* | 0 | 0.009209 | 0 | 0 |
| 4 | *Acytostelium subglobosum* | 0 | 0 | 0 | 0.016692 |
| 5 | *Aspergillus fumigatus* | 0 | 0 | 0 | 0.008346 |
| 6 | *Choanephora cucurbitarum* | 0 | 0 | 0 | 0.008346 |
| 7 | *Claviceps purpurea* | 0 | 0 | 0 | 0.008346 |
| 8 | *Mixia osmundae* | 0 | 0 | 0 | 0.008346 |
| 9 | *Pseudocercospora musae* | 0 | 0 | 0 | 0.008346 |
| 10 | *Puccinia striiformis* | 0 | 0 | 0 | 0.008346 |
| 11 | *Rhizophagus irregularis* | 0 | 0 | 0 | 0.008346 |
| 12 | *Endocarpon pusillum* | 0 | 0 | 0.006741 | 0 |
| 13 | *Aspergillus calidoustus* | 0.001686 | 0 | 0 | 0 |
| 14 | *Dacryopinax primogenitus* | 0 | 0.001842 | 0 | 0 |
| 15 | *Talaromyces marneffei* | 0 | 0.001842 | 0 | 0 |
| **Other microbial eukaryotes (algae, protozoa, parasites)** | | | | | |
| 1 | *Guillardia theta* | 0 | 0 | 0 | 0.016692 |
| 2 | *Bathycoccus prasinos* | 0 | 0 | 0 | 0.008346 |
| 3 | *Phytophthora nicotianae* | 0 | 0 | 0 | 0.008346 |
| 4 | *Tetrahymena thermophila* | 0 | 0 | 0 | 0.008346 |
| 5 | *Thalassiosira pseudonana* | 0 | 0 | 0 | 0.008346 |
| 6 | *Thalassiosira weissflogii* | 0 | 0 | 0 | 0.008346 |
| 7 | *Coccomyxa subellipsoidea* | 0 | 0.001842 | 0 | 0 |
| 8 | *Angomonas deanei* | 0 | 0 | 0 | 0.016692 |
| 9 | *Encephalitozoon cuniculi* | 0 | 0 | 0 | 0.008346 |
| 10 | *Perkinsus marinus* | 0 | 0 | 0 | 0.008346 |
| 11 | *Angomonas desouzai* | 0 | 0 | 0.006741 | 0 |
| 12 | *Acanthamoeba castellanii* | 0.001686 | 0 | 0 | 0 |
| 13 | *Pseudocohnilembus persalinus* | 0 | 0.001842 | 0 | 0 |
|  | | | | | |
|  | unclassified eukaryotic species | 0 | 0.001842 | 0 | 0 |
