# Supplementary material for: Shotgun sequence-based metataxonomic and predictive functional profiles of Pe poke, a naturally fermented soybean food of Myanmar
Source: PLoS One. 2021 Dec 17;16(12):e0260777. doi: 10.1371/journal.pone.0260777 (PMC8682898; doi:10.1371/journal.pone.0260777)
Supplement: S9 Table — (DOCX) [file pone.0260777.s009.docx]

**Supplementary Table 9.** Viral species detected in samples of *pe poke*.

| Sl. No. | Species | Relative abundance (%) | | | |
| --- | --- | --- | --- | --- | --- |
|  |  | 3ds | 4ds | 5ds | Sds |
| 1 | *Achromobacter* phage phiAxp-3 | 0 | 0 | 0 | 0.025038 |
| 2 | *Acinetobacter* phage IME-AB2 | 0 | 0 | 0.006741 | 0 |
| 3 | *Aeribacillus* phage AP45 | 0 | 0.003683 | 0.114594 | 0 |
| 4 | *Bacillus* phage BSNPO1 | 0.003372 | 0 | 0 | 0 |
| 5 | *Bacillus* phage CampHawk | 0.010115 | 0 | 0 | 0 |
| 6 | *Bacillus* phage Grass | 0.173637 | 0.099453 | 0 | 0.025038 |
| 7 | *Bacillus* phage Harambe | 0.001686 | 0 | 0 | 0 |
| 8 | *Bacillus* phage Mater | 0.006743 | 0.003683 | 0 | 0 |
| 9 | *Bacillus* phage MG-B1 | 0.008429 | 0 | 0 | 0 |
| 10 | *Bacillus* phage Mgbh1 | 0 | 0 | 0.087631 | 0 |
| 11 | *Bacillus* phage Nf | 0.011801 | 0 | 0 | 0 |
| 12 | *Bacillus* phage phi3T | 0.001686 | 0 | 0 | 0 |
| 13 | *Bacillus* phage phiNIT1 | 0.327045 | 0.176805 | 0 | 0.050075 |
| 14 | *Bacillus* phage PM1 | 0.566429 | 0.134446 | 0 | 0.041729 |
| 15 | *Bacillus* phage Shbh1 | 0.006743 | 0.001842 | 0 | 0 |
| 16 | *Bacillus* phage SIOphi | 0.237698 | 0.05341 | 0 | 0 |
| 17 | *Bacillus* phage SP-10 | 0.001686 | 0.001842 | 0 | 0 |
| 18 | *Bacillus* phage SPG24 | 0.005057 | 0.003683 | 0 | 0 |
| 19 | *Bacillus* phage SPP1 | 0.001686 | 0 | 0 | 0 |
| 20 | *Bacillus* phage vB_BanS-Tsamsa | 0.247813 | 0.023942 | 0 | 0.041729 |
| 21 | *Bacillus* virus B103 | 0.015172 | 0 | 0 | 0 |
| 22 | *Bacillus* virus G | 0.023601 | 0 | 0 | 0 |
| 23 | *Bacillus* virus GA1 | 0.003372 | 0 | 0 | 0 |
| 24 | *Bacillus* virus phi29 | 0.010115 | 0 | 0 | 0 |
| 25 | *Delftia* phage RG-2014 | 0 | 0 | 0 | 0.008346 |
| 26 | *Enterococcus* phage vB_EfaP_IME199 | 0 | 0 | 0 | 0.008346 |
| 27 | *Erwinia* phage Ea9-2 | 0 | 0 | 0 | 0.008346 |
| 28 | *Escherichia* phage N4 | 0 | 0 | 0 | 0.008346 |
| 29 | *Geobacillus* phage GBK2 | 0 | 0 | 0 | 0.016692 |
| 30 | *Geobacillus* virus E3 | 0.038773 | 0 | 0.006741 | 0.233684 |
| 31 | *Ralstonia* phage RSP15 | 0.001686 | 0 | 0 | 0 |
| 32 | *Rhizobium* phage RHEph10 | 0 | 0 | 0.013482 | 0 |
| 33 | *Staphylococcus* phage Twort | 0 | 0 | 0.013482 | 0 |
|  | unclassified viral species | 0 | 0 | 0.020222 | 0 |
