# Supplementary material for: Shotgun sequence-based metataxonomic and predictive functional profiles of Pe poke, a naturally fermented soybean food of Myanmar
Source: PLoS One. 2021 Dec 17;16(12):e0260777. doi: 10.1371/journal.pone.0260777 (PMC8682898; doi:10.1371/journal.pone.0260777)
Supplement: S10 Table — (DOCX) [file pone.0260777.s010.docx]

**Supplementary Table 10.** Shared and unique bacterial species detected in *pe poke*

| Samples Code | Number of species | Bacterial species |
| --- | --- | --- |
| 3ds, 4ds, 5ds and Sds | 204 | *Bacillus clausii* |
|  |  | *Parageobacillus thermoglucosidans* |
|  |  | *Bacillus subterraneus* |
|  |  | *Enterococcus faecium* |
|  |  | *Oceanospirillum beijerinckii* |
|  |  | *Bacillus bogoriensis* |
|  |  | *Parageobacillus toebii* |
|  |  | *Paenisporosarcina* sp. TG20 |
|  |  | *Bacillus gottheilii* |
|  |  | *Gracilibacillus timonensis* |
|  |  | *Brevibacillus parabrevis* |
|  |  | *Gluconobacter frateurii* |
|  |  | *Virgibacillus pantothenticus* |
|  |  | *Bacillus psychrosaccharolyticus* |
|  |  | *Bacillus caseinilyticus* |
|  |  | *Xanthomonas citri* |
|  |  | *Bacillus ligniniphilus* |
|  |  | *Lysinibacillus massiliensis* |
|  |  | *Pontibacillus litoralis* |
|  |  | *Bacillus salsus* |
|  |  | *Bacillus krulwichiae* |
|  |  | *Sediminibacillus halophilus* |
|  |  | *Bacteroides fragilis* |
|  |  | *Gracilibacillus halophilus* |
|  |  | *Bacillus* sp. J37 |
|  |  | *Bacillus nakamurai* |
|  |  | *Ignatzschineria larvae* |
|  |  | *Bacillus oleronius* |
|  |  | *Wohlfahrtiimonas* sp. 34C10-3-10 |
|  |  | *Vagococcus lutrae* |
|  |  | *Aeribacillus pallidus* |
|  |  | *Bacillus* sp. UNC41MFS5 |
|  |  | *Marinobacter* sp. EN3 |
|  |  | *Fictibacillus phosphorivorans* |
|  |  | *Thermoactinomyces* sp. DSM 45892 |
|  |  | *Carnobacterium maltaromaticum* |
|  |  | *Bacillus acidiproducens* |
|  |  | *Psychrobacillus* sp. OK032 |
|  |  | *Oceanobacillus sojae* |
|  |  | *Anoxybacillus flavithermus* |
|  |  | *Geobacillus* sp. Sah69 |
|  |  | *Bacillus niacini* |
|  |  | *Bacillus galactosidilyticus* |
|  |  | *Edaphobacillus lindanitolerans* |
|  |  | *Bacillus subtilis* |
|  |  | *Globicatella sulfidifaciens* |
|  |  | *Bacillus badius* |
|  |  | *Bacillus shackletonii* |
|  |  | *Bacillus pumilus* |
|  |  | *Bacillus andreraoultii* |
|  |  | *Aneurinibacillus thermoaerophilus* |
|  |  | *Bacillus licheniformis* |
|  |  | *Oceanobacillus damuensis* |
|  |  | *Bacillus fumarioli* |
|  |  | *Jeotgalibaca dankookensis* |
|  |  | *Bacillus* sp. VT-16-64 |
|  |  | *Anoxybacillus* sp. BCO1 |
|  |  | *Bacillus* sp. EB01 |
|  |  | *Bacillus panaciterrae* |
|  |  | *Enterococcus faecalis* |
|  |  | *Caryophanon latum* |
|  |  | *Fictibacillus gelatini* |
|  |  | *Bacillus* sp. MB2021 |
|  |  | *Kurthia* sp. 11kri321 |
|  |  | *Bacillus amyloliquefaciens* |
|  |  | *Paenibacillus macerans* |
|  |  | *Wohlfahrtiimonas larvae* |
|  |  | *Parageobacillus thermantarcticus* |
|  |  | *Enterococcus phoeniculicola* |
|  |  | *Paucisalibacillus globulus* |
|  |  | *Bacillus okhensis* |
|  |  | *Bacillus marisflavi* |
|  |  | *Ureibacillus thermosphaericus* |
|  |  | *Caldalkalibacillus thermarum* |
|  |  | *Staphylococcus aureus* |
|  |  | *Geobacillus* sp. 8 |
|  |  | *Bacillus smithii* |
|  |  | *Bacillus mycoides* |
|  |  | *Listeria grayi* |
|  |  | *Bacillus* sp. FJAT-27445 |
|  |  | *Ornithinibacillus californiensis* |
|  |  | *Noviherbaspirillum* sp. Root189 |
|  |  | *Amphibacillus xylanus* |
|  |  | *Aquibacillus* sp. Marseille-P3518 |
|  |  | *Bacillus massiliosenegalensis* |
|  |  | *Bacillus fordii* |
|  |  | *Escherichia vulneris* |
|  |  | *Bacillus alcalophilus* |
|  |  | *Geobacillus thermoleovorans* |
|  |  | *Bacillus ginsengihumi* |
|  |  | *Lentibacillus jeotgali* |
|  |  | *Lactobacillus rhamnosus* |
|  |  | *Pontibacillus yanchengensis* |
|  |  | *Bacillus firmus* |
|  |  | *Anaerobacillus alkalilacustris* |
|  |  | *Bacillus coagulans* |
|  |  | *Pseudomonas litoralis* |
|  |  | *Sporosarcina ureae* |
|  |  | *Bacillus sporothermodurans* |
|  |  | *Bacillus* sp. OK048 |
|  |  | *Bacillus anthracis* |
|  |  | *Paenibacillus* sp. GM2 |
|  |  | *Gilliamella apicola* |
|  |  | *Bacillus velezensis* |
|  |  | *Bhargavaea cecembensis* |
|  |  | *Bacillus lentus* |
|  |  | *Parageobacillus caldoxylosilyticus* |
|  |  | *Bacillus alveayuensis* |
|  |  | *Paenibacillus* sp. P22 |
|  |  | *Bacillus thermoamylovorans* |
|  |  | *Bacillus wakoensis* |
|  |  | *Vitreoscilla stercoraria* |
|  |  | *Bacillus bataviensis* |
|  |  | *Bacillus* sp. X1(2014) |
|  |  | *Heliobacterium modesticaldum* |
|  |  | *Alkalibacillus haloalkaliphilus* |
|  |  | *Oleispira antarctica* |
|  |  | *Bacillus megaterium* |
|  |  | *Bacillus testis* |
|  |  | *Bacillus thuringiensis* |
|  |  | *Lysinibacillus xylanilyticus* |
|  |  | *Bacillus weihaiensis* |
|  |  | *Lactococcus lactis* |
|  |  | *Klebsiella pneumoniae* |
|  |  | *Bacillus persicus* |
|  |  | *Anaerobacillus* sp. NB2006 |
|  |  | *Bacillus* sp. LF1 |
|  |  | *Oceanobacillus* sp. Castelsardo |
|  |  | *Bacillus niameyensis* |
|  |  | *Listeria monocytogenes* |
|  |  | *Bacillus vireti* |
|  |  | *Bacillus* sp. NC2-31 |
|  |  | *Pasteurella multocida* |
|  |  | *Pseudomonas aeruginosa* |
|  |  | *Lysinibacillus sphaericus* |
|  |  | *Bacillus* sp. LL01 |
|  |  | *Ornithinibacillus scapharcae* |
|  |  | *Caenibacillus caldisaponilyticus* |
|  |  | *Gracilibacillus ureilyticus* |
|  |  | *Bacillus timonensis* |
|  |  | *Bacillus cereus* |
|  |  | *Kerstersia gyiorum* |
|  |  | *Enterococcus durans* |
|  |  | *Lysinibacillus manganicus* |
|  |  | *Bacillus methanolicus* |
|  |  | *Neisseria shayeganii* |
|  |  | *Bacillus halodurans* |
|  |  | *Sporosarcina koreensis* |
|  |  | *Bacillus endophyticus* |
|  |  | *Clostridium botulinum* |
|  |  | *Lysinibacillus sinduriensis* |
|  |  | *Listeria seeligeri* |
|  |  | *Paucisalibacillus* sp. EB02 |
|  |  | *Bacillus* sp. SA1-12 |
|  |  | *Haemophilus parainfluenzae* |
|  |  | *Tenuibacillus multivorans* |
|  |  | *Bacillus aquimaris* |
|  |  | *Ornithinibacillus halophilus* |
|  |  | *Bacillus flexus* |
|  |  | *Paraliobacillus ryukyuensis* |
|  |  | *Bacillus sinesaloumensis* |
|  |  | *Virgibacillus proomii* |
|  |  | *Bacillus* sp. OxB-1 |
|  |  | *Terribacillus halophilus* |
|  |  | *Oceanobacillus caeni* |
|  |  | *Virgibacillus dokdonensis* |
|  |  | *Lysobacter concretionis* |
|  |  | *Virgibacillus halodenitrificans* |
|  |  | *Bacillus solani* |
|  |  | *Bacillus* sp. FJAT-29814 |
|  |  | *Caldibacillus debilis* |
|  |  | *Oceanobacillus oncorhynchi* |
|  |  | *Bacillus shacheensis* |
|  |  | *Bacillus mojavensis* |
|  |  | *Mycobacterium abscessus* |
|  |  | *Bacillus sonorensis* |
|  |  | *Bacillus massiliogorillae* |
|  |  | *Geobacillus kaustophilus* |
|  |  | *Rummeliibacillus stabekisii* |
|  |  | *Amphibacillus sediminis* |
|  |  | *Sporolactobacillus terrae* |
|  |  | *Paenibacillus dendritiformis* |
|  |  | *Oceanobacillus jeddahense* |
|  |  | *Novibacillus thermophilus* |
|  |  | *Bacillus nealsonii* |
|  |  | *Bacillus mesonae* |
|  |  | *Bacillus* sp. B14905 |
|  |  | *Anoxybacillus* sp. UARK-01 |
|  |  | *Bacillus azotoformans* |
|  |  | *Domibacillus enclensis* |
|  |  | *Bacillus fastidiosus* |
|  |  | *Bacillus oceanisediminis* |
|  |  | *Bacillus rubiinfantis* |
|  |  | *Oceanobacillus limi* |
|  |  | *Bacillus eiseniae* |
|  |  | *Staphylococcus epidermidis* |
|  |  | *Bacillus pseudofirmus* |
|  |  | *Marinobacterium profundum* |
|  |  | *Enterococcus pallens* |
|  |  | *Enterococcus mundtii* |
|  |  | *Bacillus* sp. FJAT-25496 |
|  |  | *Acinetobacter* *qingfengensis* |
|  |  | *Geobacillus stearothermophilus* |
|  |  | *Halomonas pantelleriensis* |
| 3ds, 4ds, 5ds | 33 | *Flavobacterium* sp. ACAM 123 |
|  |  | *Morganella morganii* |
|  |  | *Bacillus paralicheniformis* |
|  |  | *Bacillus* sp. MKU004 |
|  |  | *Lactococcus piscium* |
|  |  | *Domibacillus robiginosus* |
|  |  | *Lonsdalea quercina* |
|  |  | *Clostridium acetireducens* |
|  |  | *Anoxybacillus ayderensis* |
|  |  | *Bacillus massilioanorexius* |
|  |  | *Paenibacillus sabinae* |
|  |  | *Herbaspirillum frisingense* |
|  |  | *Streptococcus anginosus* |
|  |  | *Psychrobacter piscatorii* |
|  |  | Geobacillus sp. WSUCF1 |
|  |  | *Geobacillus* sp. PA-3 |
|  |  | *Bacillus* sp. UNC438CL73TsuS30 |
|  |  | *Bacillus aurantiacus* |
|  |  | *Lysinibacillus* sp. FJAT-14745 |
|  |  | *Bacillus farraginis* |
|  |  | *Acidovorax citrulli* |
|  |  | *Coprococcus comes* |
|  |  | *Staphylococcus hominis* |
|  |  | *Flavobacterium columnare* |
|  |  | *Paenibacillus macquariensis* |
|  |  | *Paenibacillus odorifer* |
|  |  | *Proteiniborus* sp. DW1 |
|  |  | *Bacillus lonarensis* |
|  |  | *Clostridium kluyveri* |
|  |  | *Domibacillus antri* |
|  |  | *Streptomyces rubidus* |
|  |  | *Clostridium* sp. Marseille-P299 |
|  |  | *Eremococcus coleocola* |
| 3ds, 4ds, Sds | 86 | *Lactobacillus casei* |
|  |  | *Thalassobacillus devorans* |
|  |  | *Chlamydia trachomatis* |
|  |  | *Allofustis seminis* |
|  |  | *Burkholderia cenocepacia* |
|  |  | *Paenibacillus* sp. oral taxon 786 |
|  |  | *Sporolactobacillus inulinus* |
|  |  | *Bacillus safensis* |
|  |  | *Listeria innocua* |
|  |  | *Geobacillus* sp. BCO2 |
|  |  | *Neptuniibacter caesariensis* |
|  |  | *Bacillus gaemokensis* |
|  |  | *Geobacillus icigianus* |
|  |  | *Streptococcus pneumoniae* |
|  |  | *Sporolactobacillus nakayamae* |
|  |  | *Hahella ganghwensis* |
|  |  | *Tetragenococcus halophilus* |
|  |  | *Desulfuribacillus alkaliarsenatis* |
|  |  | *Pontibacillus halophilus* |
|  |  | *Jeotgalibacillus campisalis* |
|  |  | *Garciella nitratireducens* |
|  |  | *Bacillus novalis* |
|  |  | *Bacillus koreensis* |
|  |  | *Lysinibacillus odysseyi* |
|  |  | *Enterococcus aquimarinus* |
|  |  | *Sporosarcina globispora* |
|  |  | *Salsuginibacillus kocurii* |
|  |  | *Fictibacillus macauensis* |
|  |  | *Bacillus cellulosilyticus* |
|  |  | *Tuberibacillus* sp. Marseille-P3662 |
|  |  | *Lactobacillus plantarum* |
|  |  | *Enterococcus gallinarum* |
|  |  | *Marinilactibacillus piezotolerans* |
|  |  | *Lactobacillus salivarius* |
|  |  | *Bacillus* sp. Marseille-P2384 |
|  |  | *Bacillus* sp. URHB0009 |
|  |  | *Sporomusa malonica* |
|  |  | *Phascolarctobacterium* sp. CAG:207 |
|  |  | *Enterococcus massiliensis* |
|  |  | *Bacillus* sp. FJAT-27916 |
|  |  | *Bacillus* sp. FJAT-22058 |
|  |  | *Halobacillus hunanensis* |
|  |  | *Bacillus tuaregi* |
|  |  | *Bacillus* sp. J33 |
|  |  | *Bacillus* sp. ES3 |
|  |  | *Desulfotomaculum hydrothermale* |
|  |  | *Legionella jordanis* |
|  |  | *Bacillus* sp. BT1B_CT2 |
|  |  | *Clostridium neonatale* |
|  |  | *Brachymonas denitrificans* |
|  |  | *Rubeoparvulum massiliense* |
|  |  | *Streptococcus agalactiae* |
|  |  | *Fangia hongkongensis* |
|  |  | *Psychrobacillus psychrodurans* |
|  |  | *Bacillus* sp. FJAT-27225 |
|  |  | *Haemophilus influenzae* |
|  |  | *Bacillus alkalitelluris* |
|  |  | *Bacillus chagannorensis* |
|  |  | *Anaerobacillus alkalidiazotrophicus* |
|  |  | *Clostridium* sp. CAG:510 |
|  |  | *Sporanaerobacter* sp. PP17-6a |
|  |  | *Atopobacter* *phocae* |
|  |  | *Massilibacterium senegalense* |
|  |  | *Bacillus horneckiae* |
|  |  | *Coprobacillus* sp. 8_1_38FAA |
|  |  | *Bacillus dielmoensis* |
|  |  | *Bacillus* sp. FJAT-27251 |
|  |  | *Streptococcus salivarius* |
|  |  | *Streptococcus gordonii* |
|  |  | *Bacillus* sp. FJAT-44921 |
|  |  | *Paenibacillus* sp. IHBB 10380 |
|  |  | *Enterococcus gilvus* |
|  |  | *Brevibacillus laterosporus* |
|  |  | *Oceanobacillus iheyensis* |
|  |  | *Jeotgalibacillus* soli Cunha et al. 2012 |
|  |  | *Bacillus cytotoxicus* |
|  |  | *Bacillus* sp. MUM 116 |
|  |  | *Planococcus* sp. L10.15 |
|  |  | *Bacillus* sp. Marseille-P2366 |
|  |  | *Staphylococcus massiliensis* |
|  |  | *Cupriavidus basilensis* |
|  |  | *Bacillus simplex* |
|  |  | *Vagococcus teuberi* |
|  |  | *Tetragenococcus muriaticus* |
|  |  | *Bacillus humi* |
|  |  | *Clostridium pasteurianum* |
| 3ds, 5ds, Sds | 49 | *Bacillus* sp. SJS |
|  |  | *Pilibacter termitis* |
|  |  | *Moellerella wisconsensis* |
|  |  | *Pelagirhabdus alkalitolerans* |
|  |  | *Bacillus okuhidensis* |
|  |  | *Varibaculum timonense* |
|  |  | *Chlamydia abortus* |
|  |  | *Bacillus jeotgali* |
|  |  | *Bacillus solimangrovi* |
|  |  | *Ornithinibacillus contaminans* |
|  |  | *Lentibacillus amyloliquefaciens* |
|  |  | *Jeotgalibacillus malaysiensis* |
|  |  | *Gracilibacillus boraciitolerans* |
|  |  | *Bacillus* sp. JCM 19034 |
|  |  | *Virgibacillus* sp. SK37 |
|  |  | *Caryophanon tenue* |
|  |  | *Sporosarcina psychrophila* |
|  |  | *Viridibacillus arvi* |
|  |  | *Psychrobacillus* sp. OK028 |
|  |  | *Enterococcus saccharolyticus* |
|  |  | *Providencia burhodogranariea* |
|  |  | *Domibacillus* sp. SAB 38 |
|  |  | *Massilia timonae* |
|  |  | *Paenibacillus ihumii* |
|  |  | *Enterococcus sulfureus* |
|  |  | *Tuberibacillus calidus* |
|  |  | *Thalassobacillus cyri* |
|  |  | *Bacillus circulans* |
|  |  | *Pragia fontium* |
|  |  | *Bacillus manliponensis* |
|  |  | *Psychrobacillus psychrotolerans* |
|  |  | *Bacillus* sp. FJAT-27986 |
|  |  | *Lysinibacillus saudimassiliensis* |
|  |  | *Bacillus ndiopicus* |
|  |  | *Virgibacillus senegalensis* |
|  |  | *Streptococcus pyogenes* |
|  |  | *Lysinibacillus* sp. ZYM-1 |
|  |  | *Lysinibacillus macroides* |
|  |  | *Virgibacillus alimentarius* |
|  |  | *Oceanobacillus picturae* |
|  |  | *Clostridioides difficile* |
|  |  | *Salinibacillus kushneri* |
|  |  | *Clostridium perfringens* |
|  |  | *Tepidimicrobium xylanilyticum* |
|  |  | *Bacillus cohnii* |
|  |  | *Brevibacillus* sp. CF112 |
|  |  | *Thermoflavimicrobium dichotomicum* |
|  |  | *Desulfosporosinus* sp. OT |
|  |  | *Gracilibacillus orientalis* |
| 4ds, 5ds, Sds | 241 | *Microvirgula aerodenitrificans* |
|  |  | *Candidimonas bauzanensis* |
|  |  | *Rhodopseudomonas palustris* |
|  |  | *Burkholderia* sp. JS23 |
|  |  | *Bordetella* genomosp. 9 |
|  |  | *Halomonas xianhensis* |
|  |  | *Bordetella* sp. SCN 67-23 |
|  |  | *Thiomonas* sp. CB2 |
|  |  | *Pseudomonas sabulinigri* |
|  |  | *Rhodoferax ferrireducens* |
|  |  | *Achromobacter piechaudii* |
|  |  | *Azotobacter vinelandii* |
|  |  | *Bordetella trematum* |
|  |  | *Marinospirillum minutulum* |
|  |  | *Bordetella avium* |
|  |  | *Marinococcus halophilus* |
|  |  | *Bordetella* sp. H567 |
|  |  | *Ectothiorhodospira* sp. PHS-1 |
|  |  | *Sporosarcina* sp. P37 |
|  |  | *Oblitimonas alkaliphila* |
|  |  | *Lautropia mirabilis* |
|  |  | *Xenophilus azovorans* |
|  |  | *Burkholderia* sp. JPY347 |
|  |  | *Oceanicola granulosus* |
|  |  | *Duganella sacchari* |
|  |  | *Pseudomonas bauzanensis* |
|  |  | *Dysgonomonas* sp. BGC7 |
|  |  | *Pusillimonas noertemannii* |
|  |  | *Sphingobacterium mizutaii* |
|  |  | *Amphibacillus jilinensis* |
|  |  | *Halomonas muralis* |
|  |  | *Halomonas elongata* |
|  |  | *Brackiella oedipodis* |
|  |  | *Azotobacter beijerinckii* |
|  |  | *Pseudomonas stutzeri* |
|  |  | *Caballeronia sordidicola* |
|  |  | *Comamonas terrigena* |
|  |  | *Chromohalobacter japonicus* |
|  |  | *Geobacillus* sp. WCH70 |
|  |  | *Empedobacter brevis* |
|  |  | *Alteribacillus bidgolensis* |
|  |  | *Achromobacter denitrificans* |
|  |  | *Acinetobacter rudis* |
|  |  | *Pseudomonas brassicacearum* |
|  |  | *Aquisalimonas asiatica* |
|  |  | *Ottowia thiooxydans* |
|  |  | *Proteiniphilum saccharofermentans* |
|  |  | *Hydrocarboniphaga effusa* |
|  |  | *Cupriavidus pauculus* |
|  |  | *Hahella chejuensis* |
|  |  | *Serratia marcescens* |
|  |  | *Pseudomonas formosensis* |
|  |  | *Planococcus massiliensis* |
|  |  | *Providencia rettgeri* |
|  |  | *Ottowia* sp. oral taxon 894 |
|  |  | *Xenorhabdus mauleonii* |
|  |  | *Xanthomonas* sp. Mitacek01 |
|  |  | *Bordetella hinzii* |
|  |  | *Comamonas composti* |
|  |  | *Achromobacter* sp. RTa |
|  |  | *Thiothrix disciformis* |
|  |  | *Taylorella asinigenitalis* |
|  |  | *Bordetella bronchiseptica* |
|  |  | *Oceanobacillus timonensis* |
|  |  | *Herbaspirillum* sp. YR522 |
|  |  | *Castellaniella caeni* |
|  |  | *Flavobacterium marinum* |
|  |  | *Providencia heimbachae* |
|  |  | *Virgibacillus siamensis* |
|  |  | *Snodgrassella alvi* |
|  |  | *Noviherbaspirillum massiliense* |
|  |  | *Zymomonas mobilis* |
|  |  | *Aquamicrobium defluvii* |
|  |  | *Bordetella petrii* |
|  |  | *Neisseria bacilliformis* |
|  |  | *Bacillus* sp. FJAT-25547 |
|  |  | *Gracilibacillus kekensis* |
|  |  | *Nitrincola nitratireducens* |
|  |  | *Bacillus aidingensis* |
|  |  | *Proteus mirabilis* |
|  |  | *Woeseia oceani* |
|  |  | *Paracoccus alcaliphilus* |
|  |  | *Yangia* sp. CCB-MM3 |
|  |  | *Photobacterium halotolerans* |
|  |  | *Rodentibacter ratti* |
|  |  | *Advenella kashmirensis* |
|  |  | *Pseudomonas monteilii* |
|  |  | *Thorsellia anophelis* |
|  |  | *Natribacillus halophilus* |
|  |  | *Lactobacillus fermentum* |
|  |  | *Marinomonas spartinae* |
|  |  | *Bordetella* sp. N |
|  |  | *Methylibium* sp. CF059 |
|  |  | *Bordetella ansorpii* |
|  |  | *Psychrobacter* sp. DAB_AL43B |
|  |  | *Marinospirillum alkaliphilum* |
|  |  | *Paraburkholderia caryophylli* |
|  |  | *Lautropia* sp. SCN 70-15 |
|  |  | *Burkholderia pseudomallei* |
|  |  | *Pseudomonas syringae* |
|  |  | *Stenotrophomonas maltophilia* |
|  |  | *Castellaniella defragrans* |
|  |  | *Pusillimonas* sp. T7-7 |
|  |  | *Psychrobacter* sp. 1501(2011) |
|  |  | *Pseudomonas* sp. StFLB209 |
|  |  | *Mannheimia haemolytica* |
|  |  | *Azospirillum brasilense* |
|  |  | *Moraxella caviae* |
|  |  | *Kurthia senegalensis* |
|  |  | *Snodgrassella* sp. CFCC 13594 |
|  |  | *Aeromonas* sp. RU39B |
|  |  | *Parasutterella excrementihominis* |
|  |  | *Microbulbifer mangrovi* |
|  |  | *Desulfosporosinus lacus* |
|  |  | *Comamonas testosteroni* |
|  |  | *Flavobacterium flevense* |
|  |  | *Photobacterium damselae* |
|  |  | *Halolactibacillus alkaliphilus* |
|  |  | *Bordetella holmesii* |
|  |  | *Thiomonas* sp. FB-Cd |
|  |  | *Globicatella sanguinis* |
|  |  | *Lentibacillus persicus* |
|  |  | *Lactobacillus parabrevis* |
|  |  | *Thauera phenylacetica* |
|  |  | *Nitrosospira multiformis* |
|  |  | *Pseudomonas thermotolerans* |
|  |  | *Conchiformibius steedae* |
|  |  | *Halomonas lutea* |
|  |  | *Lampropedia cohaerens* |
|  |  | *Kurthia huakuii* |
|  |  | *Oceanimonas* sp. GK1 |
|  |  | *Lampropedia hyalina* |
|  |  | *Comamonas* sp. B-9 |
|  |  | *Alteromonas macleodii* |
|  |  | *Variovorax paradoxus* |
|  |  | *Caballeronia megalochromosomata* |
|  |  | *Wohlfahrtiimonas chitiniclastica* |
|  |  | *Bacillus* sp. FJAT-29937 |
|  |  | *Thiomonas* sp. FB-6 |
|  |  | *Bordetella* genomosp. 13 |
|  |  | *Marinobacterium stanieri* |
|  |  | *Ideonella sakaiensis* |
|  |  | *Amphibacillus marinus* |
|  |  | *Bacillus hemicellulosilyticus* |
|  |  | *Providencia stuartii* |
|  |  | *Achromobacter* sp. Root83 |
|  |  | *Pseudospirillum japonicum* |
|  |  | *Alcaligenes faecalis* |
|  |  | *Polynucleobacter* sp. MWH-Weng1-1 |
|  |  | *Massilia* sp. Leaf139 |
|  |  | *Acinetobacter albensis* |
|  |  | *Basilea psittacipulmonis* |
|  |  | *Lysobacter antibioticus* |
|  |  | *Vitreoscilla* sp. SN6 |
|  |  | *Cupriavidus necator* |
|  |  | *Marinomonas fungiae* |
|  |  | *Myroides guanonis* |
|  |  | *Burkholderia ubonensis* |
|  |  | *Pseudomonas caeni* |
|  |  | *Orrella dioscoreae* |
|  |  | *Paenisporosarcina indica* |
|  |  | *Planococcus maritimus* |
|  |  | *Oligella urethralis* |
|  |  | *Lysinibacillus* sp. BF-4 |
|  |  | *Andreprevotia lacus* |
|  |  | *Advenella mimigardefordensis* |
|  |  | *Leeia oryzae* |
|  |  | *Burkholderia mallei* |
|  |  | *Taylorella equigenitalis* |
|  |  | *Aquaspirillum* sp. LM1 |
|  |  | *Gallaecimonas xiamenensis* |
|  |  | *Bacillus dakarensis* |
|  |  | *Vibrio cyclitrophicus* |
|  |  | *Marinobacter* sp. T13-3 |
|  |  | *Aneurinibacillus tyrosinisolvens* |
|  |  | *Ralstonia solanacearum* |
|  |  | *Variovorax* sp. HW608 |
|  |  | *Leucothrix mucor* |
|  |  | *Pectobacterium carotovorum* |
|  |  | *Halotalea alkalilenta* |
|  |  | *Bordetella* sp. FB-8 |
|  |  | *Methylomonas koyamae* |
|  |  | *Bergeriella denitrificans* |
|  |  | *Brenneria goodwinii* |
|  |  | *Shewanella mangrovi* |
|  |  | *Pandoraea thiooxydans* |
|  |  | *Bordetella bronchialis* |
|  |  | *Escherichia coli* |
|  |  | *Aliihoeflea* sp. 2WW |
|  |  | *Pseudomonas putida* |
|  |  | *Verminephrobacter eiseniae* |
|  |  | *Marinobacterium georgiense* |
|  |  | *Thauera* sp. ZV-1-C |
|  |  | *Achromobacter* sp. DMS1 |
|  |  | *Paucibacter* sp. KCTC 42545 |
|  |  | *Ectothiorhodosinus mongolicus* |
|  |  | *Burkholderia cepacia* |
|  |  | *Pseudomonas alcaligenes* |
|  |  | *Ignatzschineria indica* |
|  |  | *Vibrio cholerae* |
|  |  | *Bibersteinia trehalosi* |
|  |  | *Tepidibacillus decaturensis* |
|  |  | *Bacillus halmapalus* |
|  |  | *Sporosarcina* sp. D27 |
|  |  | *Bacillus pseudalcaliphilus* |
|  |  | *Salimicrobium halophilum* |
|  |  | *Planomicrobium glaciei* |
|  |  | *Planctomyces* sp. SH-PL14 |
|  |  | *Herbaspirillum autotrophicum* |
|  |  | *Bordetella* genomosp. 8 |
|  |  | *Acidihalobacter prosperus* |
|  |  | *Pseudomonas* sp. TTU2014-080ASC |
|  |  | *Enterobacter cloacae* |
|  |  | *Achromobacter* sp. DH1f |
|  |  | *Acinetobacter nosocomialis* |
|  |  | *Luteimonas* sp. JM171 |
|  |  | *Pseudomonas linyingensis* |
|  |  | *Methylobacillus* sp. MM2 |
|  |  | *Paenisporosarcina* sp. HGH0030 |
|  |  | *Salipaludibacillus aurantiacus* |
|  |  | *Propionivibrio dicarboxylicus* |
|  |  | *Pasteurella testudinis* |
|  |  | *Pseudomonas fluorescens* |
|  |  | *Ralstonia* sp. PBA |
|  |  | *Acidovorax* sp. MR-S7 |
|  |  | *Gulbenkiania indica* |
|  |  | *Herbaspirillum* sp. RV1423 |
|  |  | *Lysinibacillus contaminans* |
|  |  | *Sphingobacterium* sp. JB170 |
|  |  | *Alicyclobacillus macrosporangiidus* |
|  |  | *Sporosarcina newyorkensis* |
|  |  | *Acidovorax* sp. RAC01 |
|  |  | *Marinospirillum insulare* |
|  |  | *Hoeflea* sp. BAL378 |
|  |  | *Oligella ureolytica* |
|  |  | *Salmonella enterica* |
|  |  | *Acinetobacter baumannii* |
|  |  | *Acinetobacter larvae* |
|  |  | *Bordetella flabilis* |
|  |  | *Azovibrio restrictus* |
|  |  | *Brachymonas chironomi* |
| 3ds, 4ds | 46 | *Enterococcus dispar* |
|  |  | *Clostridium* sp. N3C |
|  |  | *Bacillus* sp. 491mf |
|  |  | *Trichococcus pasteurii* |
|  |  | *Lysobacter arseniciresistens* |
|  |  | *Enterococcus cecorum* |
|  |  | *Staphylococcus* sp. MB371 |
|  |  | *Mycoplasma meleagridis* |
|  |  | *Lactobacillus curvatus* |
|  |  | *Hydrogenoanaerobacterium saccharovorans* |
|  |  | *Anoxybacillus suryakundensis* |
|  |  | *Bacillus weihenstephanensis* |
|  |  | *Facklamia ignava* |
|  |  | *Leuconostoc citreum* |
|  |  | *Bacillus* sp. CDB3 |
|  |  | *Enterococcus avium* |
|  |  | *Roseburia inulinivorans* |
|  |  | *Enterococcus devriesei* |
|  |  | *Paenibacillus glucanolyticus* |
|  |  | *Fictibacillus enclensis* |
|  |  | *Bacillus altitudinis* |
|  |  | *Anoxybacillus amylolyticus* |
|  |  | *Bacillus acidicola* |
|  |  | *Bacillus* sp. NRRL B-41327 |
|  |  | *Salinicoccus carnicancri* |
|  |  | *Enterococcus columbae* |
|  |  | *Dethiosulfatarculus sandiegensis* |
|  |  | *Enterococcus ratti* |
|  |  | *Weissella oryzae* |
|  |  | *Pseudobutyrivibrio xylanivorans* |
|  |  | *Paenibacillus popilliae* |
|  |  | *Saccharibacillus sacchari* |
|  |  | *Granulicatella balaenopterae* |
|  |  | *Enterococcus silesiacus* |
|  |  | *Bacillus* sp. NRRL B-14911 |
|  |  | *Lactobacillus jensenii* |
|  |  | *Lactobacillus ruminis* |
|  |  | *Anaerocolumna aminovalerica* |
|  |  | *Thermoactinomyces vulgaris* |
|  |  | *Bacillus* sp. TH008 |
|  |  | *Bacillus vietnamensis* |
|  |  | *Clostridium* sp. W14A |
|  |  | *Sphaerochaeta globosa* |
|  |  | *Listeria aquatica* |
|  |  | *Desulfotomaculum aeronauticum* |
|  |  | *Eisenbergiella tayi* |
| 3ds, 5ds | 16 | *Carnobacterium* sp. AT7 |
|  |  | *Exiguobacterium antarcticum* |
|  |  | *Bacteroides xylanisolvens* |
|  |  | *Riemerella anatipestifer* |
|  |  | *Aerococcus viridans* |
|  |  | *Paenibacillus wynnii* |
|  |  | *Xenorhabdus bovienii* |
|  |  | *Geobacillus* sp. 12AMOR1 |
|  |  | *Enterococcus canintestini* |
|  |  | *Trichococcus flocculiformis* |
|  |  | *Geobacillus jurassicus* |
|  |  | *Carnobacterium mobile* |
|  |  | *Halanaerobium praevalens* |
|  |  | *Bacillus* sp. 522_BSPC |
|  |  | *Methylobacterium* sp. AMS5 |
|  |  | *Alkaliphilus oremlandii* |
| 3ds, Sds | 75 | *Halobacillus* sp. BBL2006 |
|  |  | *Erysipelothrix rhusiopathiae* |
|  |  | *Carnobacterium viridans* |
|  |  | *Lactobacillus xiangfangensis* |
|  |  | *Bacillus kribbensis* |
|  |  | *Exiguobacterium sibiricum* |
|  |  | *Paenibacillus* sp. Soil787 |
|  |  | *Halobacillus kuroshimensis* |
|  |  | *Vagococcus fluvialis* |
|  |  | *Lentibacillus halodurans* |
|  |  | *Paenibacillus* sp. TI45-13ar |
|  |  | *Staphylococcus equorum* |
|  |  | *Anaerosalibacter massiliensis* |
|  |  | *Oceanobacillus kimchii* |
|  |  | *Alkalibacter saccharofermentans* |
|  |  | *Vagococcus* sp. D7T301 |
|  |  | *Paenibacillus fonticola* |
|  |  | *Gardnerella vaginalis* |
|  |  | *Paraliobacillus* sp. PM-2 |
|  |  | *Carnobacterium iners* |
|  |  | *Bacillus* sp. HMSC76G11 |
|  |  | *Parvimonas micra* |
|  |  | *Enterococcus ureasiticus* |
|  |  | *Succinatimonas* sp. CAG:777 |
|  |  | *Dethiosulfatibacter aminovorans* |
|  |  | *Paenibacillus* sp. SIT18 |
|  |  | *Vagococcus penaei* |
|  |  | *Leuconostoc mesenteroides* |
|  |  | *Enterococcus ureilyticus* |
|  |  | *Streptococcus suis* |
|  |  | *Bacillus indicus* |
|  |  | *Bacillus aryabhattai* |
|  |  | *Bacillus cihuensis* |
|  |  | *Carnobacterium* sp. CP1 |
|  |  | *Isobaculum melis* |
|  |  | *Enterococcus casseliflavus* |
|  |  | *Bacillus gobiensis* |
|  |  | *Photobacterium piscicola* |
|  |  | *Atopostipes suicloacalis* |
|  |  | *Defluviitalea phaphyphila* |
|  |  | *Paenibacillus* sp. JCM 10914 |
|  |  | *Enterococcus* sp. RIT-PI-f |
|  |  | *Bacillus daliensis* |
|  |  | *Lactobacillus algidus* |
|  |  | *Pontibacillus chungwhensis* |
|  |  | *Clostridium baratii* |
|  |  | *Enterococcus asini* |
|  |  | *Paenibacillus senegalensis* |
|  |  | *Enterococcus rivorum* |
|  |  | *Paenibacillus elgii* |
|  |  | *Carnobacterium inhibens* |
|  |  | *Melissococcus plutonius* |
|  |  | *Enterococcus* sp. TR |
|  |  | *Carnobacterium gallinarum* |
|  |  | *Atopobium fossor* |
|  |  | *Carnobacterium* sp. 17-4 |
|  |  | *Salimicrobium flavidum* |
|  |  | *Erysipelothrix tonsillarum* |
|  |  | *Enterococcus canis* |
|  |  | *Listeria fleischmannii* |
|  |  | *Brevibacillus panacihumi* |
|  |  | *Paenibacillus tianmuensis* |
|  |  | *Desulfuribacillus stibiiarsenatis* |
|  |  | *Planifilum fulgidum* |
|  |  | *Enterococcus thailandicus* |
|  |  | *Erysipelothrix larvae* |
|  |  | *Desemzia incerta* |
|  |  | *Clostridium intestinale* |
|  |  | *Hungatella hathewayi* |
|  |  | *Domibacillus iocasae* |
|  |  | *Aneurinibacillus* sp. XH2 |
|  |  | *Risungbinella massiliensis* |
|  |  | *Enterococcus malodoratus* |
|  |  | *Bacillus soli* |
|  |  | *Numidum massiliense* |
| 4ds, 5ds | 142 | *Aquamicrobium aerolatum* |
|  |  | *Pseudomonas graminis* |
|  |  | *Ramlibacter* sp. Leaf400 |
|  |  | *Chryseobacterium* sp. Hurlbut01 |
|  |  | *Pseudorhodoferax* sp. Leaf265 |
|  |  | *Acinetobacter brisouii* |
|  |  | *Flavobacterium reichenbachii* |
|  |  | *Paraburkholderia andropogonis* |
|  |  | *Limnobacter* sp. MED105 |
|  |  | *Derxia lacustris* |
|  |  | *Lautropia* sp. SCN 66-9 |
|  |  | *Algoriella xinjiangensis* |
|  |  | *Halomonas* sp. Marseille-P2426 |
|  |  | *Sphingobacterium spiritivorum* |
|  |  | *Acidisphaera rubrifaciens* |
|  |  | *Bacillus encimensis* |
|  |  | *Vogesella* sp. EB |
|  |  | *Pedobacter glucosidilyticus* |
|  |  | *Halolactibacillus* sp. JCM 19043 |
|  |  | *Leclercia* sp. LK8 |
|  |  | *Halomonas daqiaonensis* |
|  |  | *Sulfitobacter brevis* |
|  |  | *Fluviicola taffensis* |
|  |  | *Pseudaminobacter manganicus* |
|  |  | *Albimonas donghaensis* |
|  |  | *Sphingobacterium paucimobilis* |
|  |  | *Rubrivivax* sp. SCN 70-15 |
|  |  | *Legionella quinlivanii* |
|  |  | *Gallibacterium anatis* |
|  |  | *Nitrosomonas eutropha* |
|  |  | *Lamprocystis purpurea* |
|  |  | *Labrenzia* sp. OB1 |
|  |  | *Herbaspirillum* sp. TSA66 |
|  |  | *Pandoraea* sp. SD6-2 |
|  |  | *Chlorobaculum limnaeum* |
|  |  | *Beggiatoa leptomitiformis* |
|  |  | *Flavobacterium frigidimaris* |
|  |  | *Nitrobacter winogradskyi* |
|  |  | *Halomonas gudaonensis* |
|  |  | *Pedobacter oryzae* |
|  |  | *Marinobacterium lutimaris* |
|  |  | *Thioalkalimicrobium aerophilum* |
|  |  | *Acinetobacter* sp. MDS7A |
|  |  | *Enterobacter cancerogenus* |
|  |  | *Ralstonia syzygii* |
|  |  | *Fulvimarina pelagi* |
|  |  | *Bacillus enclensis* |
|  |  | *Chitinilyticum litopenaei* |
|  |  | *Psychrobacter* sp. CIP 110854 |
|  |  | *Methylosarcina fibrata* |
|  |  | *Acinetobacter* sp. 1564232 |
|  |  | *Vibrio* sp. M12-1144 |
|  |  | *Oceanimonas smirnovii* |
|  |  | *Acinetobacter* sp. ANC 4149 |
|  |  | *Vibrio crassostreae* |
|  |  | *Aquaspirillum serpens* |
|  |  | *Burkholderia* sp. TNe-862 |
|  |  | *Roseovarius pacificus* |
|  |  | *Pseudomonas saponiphila* |
|  |  | *Achromobacter* sp. LC458 |
|  |  | *Pseudomonas savastanoi* |
|  |  | *Alcanivorax hongdengensis* |
|  |  | *Sphingobacterium thalpophilum* |
|  |  | *Gayadomonas joobiniege* |
|  |  | *Pseudogulbenkiania subflava* |
|  |  | *Crenobacter luteus* |
|  |  | *Haemophilus haemolyticus* |
|  |  | *Xanthomonas massiliensis* |
|  |  | *Vibrio* sp. EJY3 |
|  |  | *Flavobacterium johnsoniae* |
|  |  | *Luteimonas* sp. FCS-9 |
|  |  | *Micromonospora rifamycinica* |
|  |  | *Janthinobacterium* sp. CG23_2 |
|  |  | *Pseudopedobacter saltans* |
|  |  | *Sphingobacterium* sp. B29 |
|  |  | *Pseudomonas knackmussii* |
|  |  | *Massilia yuzhufengensis* |
|  |  | *Acidovorax* sp. JHL-3 |
|  |  | *Halomonas* sp. PR-M31 |
|  |  | *Thiomonas intermedia* |
|  |  | *Azospirillum lipoferum* |
|  |  | *Flavobacterium ummariense* |
|  |  | *Halomonas* sp. HAL1 |
|  |  | *Paracoccus halophilus* |
|  |  | *Achromobacter* sp. 2789STDY5663426 |
|  |  | *Massilia namucuonensis* |
|  |  | *Nitrococcus mobilis* |
|  |  | *Massilia* sp. PDC64 |
|  |  | *Rhodoplanes* sp. Z2-YC6860 |
|  |  | *Pseudomonas panipatensis* |
|  |  | *Pseudomonas pseudoalcaligenes* |
|  |  | *Polaromonas* sp. C04 |
|  |  | *Sinomicrobium oceani* |
|  |  | *Pelistega* sp. MC2 |
|  |  | *Xylella fastidiosa* |
|  |  | *Aquimarina agarilytica* |
|  |  | *Sphingobacterium* sp. 21 |
|  |  | *Polynucleobacter* sp. MWH-Adler-W8 |
|  |  | *Neisseria dentiae* |
|  |  | *Parabacteroides* sp. YL27 |
|  |  | *Psychrobacter* sp. Rd 27.2 |
|  |  | *Aurantimonas* sp. 22II-16-19i |
|  |  | *Janthinobacterium* sp. Marseille |
|  |  | *Roseomonas* sp. M3 |
|  |  | *Acinetobacter haemolyticus* |
|  |  | *Paenibacillus massiliensis* |
|  |  | *Tepidiphilus margaritifer* |
|  |  | *Janthinobacterium* sp. B9-8 |
|  |  | *Bacillus pseudomycoides* |
|  |  | *Ochrobactrum intermedium* |
|  |  | *Flavobacterium suncheonense* |
|  |  | *Bradyrhizobium* sp. NFR13 |
|  |  | *Pseudomonas* sp. NBRC 111123 |
|  |  | *Bacillus horikoshii* |
|  |  | *Fusicatenibacter saccharivorans* |
|  |  | *Collimonas fungivorans* |
|  |  | *Chania multitudinisentens* |
|  |  | *Pseudoxanthomonas suwonensis* |
|  |  | *Sphingobacterium lactis* |
|  |  | *Paracoccus chinensis* |
|  |  | *Enterobacter massiliensis* |
|  |  | *Halorhodospira halophila* |
|  |  | *Methylococcus capsulatus* |
|  |  | *Pseudoalteromonas tunicata* |
|  |  | *Paenibacillus* sp. FF9 |
|  |  | *Ferrimonas senticii* |
|  |  | *Conchiformibius kuhniae* |
|  |  | *Chitiniphilus shinanonensis* |
|  |  | *Burkholderia* sp. yr281 |
|  |  | *Idiomarina* sp. A28L |
|  |  | *Empedobacter falsenii* |
|  |  | *Carnimonas nigrificans* |
|  |  | *Thiomicrospira* sp. CG2_30_44_34 |
|  |  | *Flavobacterium soli* |
|  |  | *Paraburkholderia sartisoli* |
|  |  | *Aquincola tertiaricarbonis* |
|  |  | *Flavobacterium rivuli* |
|  |  | *Burkholderia* sp. lig30 |
|  |  | *Janthinobacterium agaricidamnosum* |
|  |  | *Neisseria lactamica* |
|  |  | *Achromobacter* sp. 2789STDY5608625 |
|  |  | *Pseudorhodoferax* sp. Leaf267 |
| 4ds, Sds | 134 | *Herbaspirillum chlorophenolicum* |
|  |  | *Xanthomonas cassavae* |
|  |  | *Pseudomonas argentinensis* |
|  |  | *Succinatimonas hippei* |
|  |  | *Halomonas xinjiangensis* |
|  |  | *Megasphaera* sp. MJR8396C |
|  |  | *Alteromonas lipolytica* |
|  |  | *Hydrogenophaga* sp. LPB0072 |
|  |  | *Campylobacter rectus* |
|  |  | *Idiomarina woesei* |
|  |  | *Pantoea ananatis* |
|  |  | *Halobacillus aidingensis* |
|  |  | *Bacillus glycinifermentans* |
|  |  | *Ramlibacter tataouinensis* |
|  |  | *Methylobacillus flagellatus* |
|  |  | *Pseudanabaena* sp. PCC 7367 |
|  |  | *Collimonas pratensis* |
|  |  | *Aeromicrobium choanae* |
|  |  | *Sphingomonas sanxanigenens* |
|  |  | *Spiribacter curvatus* |
|  |  | *Roseomonas rosea* |
|  |  | *Enhydrobacter aerosaccus* |
|  |  | *Citrobacter freundii* |
|  |  | *Comamonas* sp. SCN 65-56 |
|  |  | *Paenibacillus amylolyticus* |
|  |  | *Deinococcus pimensis* |
|  |  | *Methylophaga lonarensis* |
|  |  | *Cardiobacterium hominis* |
|  |  | *Diaphorobacter* sp. J5-51 |
|  |  | *Burkholderia* sp. UYPR1.413 |
|  |  | *Massilia* sp. Root418 |
|  |  | *Andreprevotia chitinilytica* |
|  |  | *Rappaport israeli* |
|  |  | *Blautia obeum* |
|  |  | *Sphaerotilus natans* |
|  |  | *Dechloromonas denitrificans* |
|  |  | *Bacillus* sp. SG-1 |
|  |  | *Clostridium grantii* |
|  |  | *Salinisphaera hydrothermalis* |
|  |  | *Clostridium* sp. Marseille-P2415 |
|  |  | *Curvibacter delicatus* |
|  |  | *Nitrosomonas halophila* |
|  |  | *Bacillus* sp. FJAT-27997 |
|  |  | *Pseudoxanthomonas* sp. GM95 |
|  |  | *Halobacillus massiliensis* |
|  |  | *Halothece* sp. PCC 7418 |
|  |  | *Pseudomonas jinjuensis* |
|  |  | *Rhizobacter* sp. Root1221 |
|  |  | *Thiothrix eikelboomii* |
|  |  | *Laribacter hongkongensis* |
|  |  | *Halomonas* sp. JB380 |
|  |  | *Moraxella atlantae* |
|  |  | *Microvirga massiliensis* |
|  |  | *Sutterella parvirubra* |
|  |  | *Achromobacter xylosoxidans* |
|  |  | *Glaciecola nitratireducens* |
|  |  | *Methylophaga thiooxydans* |
|  |  | *Paraburkholderia oxyphila* |
|  |  | *Erwinia teleogrylli* |
|  |  | *Amorphus coralli* |
|  |  | *Pseudomonas resinovorans* |
|  |  | *Methylobacter tundripaludum* |
|  |  | *Acetobacter syzygii* |
|  |  | *Sphingomonas mali* |
|  |  | *Cellvibrio japonicus* |
|  |  | *Trichococcus palustris* |
|  |  | *Pseudomonas* sp. EGD-AK9 |
|  |  | *Paraburkholderia ribeironis* |
|  |  | *Rhizobacter gummiphilus* |
|  |  | *Burkholderia* sp. Leaf177 |
|  |  | *Gelidibacter mesophilus* |
|  |  | *Melghirimyces thermohalophilus* |
|  |  | *Oceanobacter kriegii* |
|  |  | *Acidovorax wautersii* |
|  |  | *Pelotomaculum thermopropionicum* |
|  |  | *Pseudomonas salegens* |
|  |  | *Burkholderia* sp. GAS332 |
|  |  | *Lawsonia intracellularis* |
|  |  | *Giesbergeria anulus* |
|  |  | *Lactobacillus shenzhenensis* |
|  |  | *Methyloversatilis thermotolerans* |
|  |  | *Tepidimonas taiwanensis* |
|  |  | *Polaromonas* sp. JS666 |
|  |  | *Bradyrhizobium erythrophlei* |
|  |  | *Amphritea atlantica* |
|  |  | *Halomonas* sp. BC1 |
|  |  | *Dickeya paradisiaca* |
|  |  | *Sulfuricaulis limicola* |
|  |  | *Ectothiorhodospira magna* |
|  |  | *Sideroxydans lithotrophicus* |
|  |  | *Rouxiella badensis* |
|  |  | *Brevibacillus* sp. OK042 |
|  |  | *Massilia* sp. CF038 |
|  |  | *Proteus hauseri* |
|  |  | *Legionella geestiana* |
|  |  | *Oxalobacter formigenes* |
|  |  | *Moraxella bovoculi* |
|  |  | *Thioalkalivibrio denitrificans* |
|  |  | *Uliginosibacterium gangwonense* |
|  |  | *Paraburkholderia sacchari* |
|  |  | *Alicyclobacillus* sp. USBA-503 |
|  |  | *Erwinia amylovora* |
|  |  | *Tepidiphilus thermophilus* |
|  |  | *Azorhizobium doebereinerae* |
|  |  | *Salinicoccus halodurans* |
|  |  | *Hydrogenophaga taeniospiralis* |
|  |  | *Marinimicrobium* sp. LS-A18 |
|  |  | *Bordetella pertussis* |
|  |  | *Comamonas aquatica* |
|  |  | *Yersinia enterocolitica* |
|  |  | *Halolactibacillus halophilus* |
|  |  | *Bermanella marisrubri* |
|  |  | *Halomonas cupida* |
|  |  | *Paraburkholderia megapolitana* |
|  |  | *Geobacter* sp. OR-1 |
|  |  | *Frischella perrara* |
|  |  | *Muribacter muris* |
|  |  | *Frankia* sp. EUN1f |
|  |  | *Leptospira kirschneri* |
|  |  | *Salinisphaera shabanensis* |
|  |  | *Bacillus* sp. FJAT-14578 |
|  |  | *Piscibacillus halophilus* |
|  |  | *Veillonella* sp. CAG:933 |
|  |  | *Pseudomonas yangmingensis* |
|  |  | *Bacillus marmarensis* |
|  |  | *Pediococcus acidilactici* |
|  |  | *Zoogloea* sp. LCSB751 |
|  |  | *Thiothrix flexilis* |
|  |  | *Achromobacter arsenitoxydans* |
|  |  | *Cupriavidus pinatubonensis* |
|  |  | *Bacillus* sp. MRMR6 |
|  |  | *Reinekea blandensis* |
|  |  | *Bacillus* sp. 1NLA3E |
|  |  | *Virgibacillus subterraneus* |
| 5ds, Sds | 87 | *Vibrio parahaemolyticus* |
|  |  | *Azospira oryzae* |
|  |  | *Shimwellia blattae* |
|  |  | *Myroides odoratus* |
|  |  | *Flavisolibacter ginsengisoli* |
|  |  | *Planococcus* sp. CAU13 |
|  |  | *Psychrobacter* sp. PRwf-1 |
|  |  | *Pseudomonas cichorii* |
|  |  | *Psychromonas* sp. SP041 |
|  |  | *Pseudomonas agarici* |
|  |  | *Providencia alcalifaciens* |
|  |  | *Salinicoccus alkaliphilus* |
|  |  | *Acinetobacter gerneri* |
|  |  | *Bhargavaea ginsengi* |
|  |  | *Sporosarcina* sp. ZBG7A |
|  |  | *Gallibacterium salpingitidis* |
|  |  | *Alloiococcus otitis* |
|  |  | *Bacillus coahuilensis* |
|  |  | *Bergeyella zoohelcum* |
|  |  | *Gracilibacillus massiliensis* |
|  |  | *Alteromonas* sp. Nap_26 |
|  |  | *Galbibacter marinus* |
|  |  | *Vulcanibacillus modesticaldus* |
|  |  | *Paenisporosarcina* sp. TG-14 |
|  |  | *Tropicibacter litoreus* |
|  |  | *Virgibacillus soli* |
|  |  | *Staphylococcus pseudintermedius* |
|  |  | *Sphingobacterium* sp. T2 |
|  |  | *Providencia sneebia* |
|  |  | *Morganella psychrotolerans* |
|  |  | *Gracilibacillus lacisalsi* |
|  |  | *Limnobacter* sp. CACIAM 66H1 |
|  |  | *Flavobacterium branchiophilum* |
|  |  | *Globicatella* sp. HMSC072A10 |
|  |  | *Halalkalibacillus halophilus* |
|  |  | *Azotobacter chroococcum* |
|  |  | *Fictibacillus arsenicus* |
|  |  | *Pedobacter arcticus* |
|  |  | *Proteiniborus ethanoligenes* |
|  |  | *Sphingobacterium* sp. HMSC13C05 |
|  |  | *Kurthia massiliensis* |
|  |  | *Chishuiella changwenlii* |
|  |  | *Sphingobacterium* sp. CZ-UAM |
|  |  | *Sporosarcina* sp. HYO08 |
|  |  | *Histophilus somni* |
|  |  | *Bacillus* sp. Soil768D1 |
|  |  | *Shigella flexneri* |
|  |  | *Gottschalkia acidurici* |
|  |  | *Sphingobacterium psychroaquaticum* |
|  |  | *Paramaledivibacter caminithermalis* |
|  |  | *Alteribacillus iranensis* |
|  |  | *Niabella drilacis* |
|  |  | *Marinobacter* sp. X15-166B |
|  |  | *Parabacteroides* sp. Marseille-P3160 |
|  |  | *Flavobacterium caeni* |
|  |  | *Acinetobacter* sp. P8-3-8 |
|  |  | *Marinospirillum celere* |
|  |  | *Chryseobacterium* sp. SCN 40-13 |
|  |  | *Chryseobacterium takakiae* |
|  |  | *Anaerobacillus arseniciselenatis* |
|  |  | *Oceanobacillus* sp. E9 |
|  |  | *Chryseobacterium chaponense* |
|  |  | *Cytophaga hutchinsonii* |
|  |  | *Gramella* sp. LPB0144 |
|  |  | *Budvicia aquatica* |
|  |  | *Salinicoccus qingdaonensis* |
|  |  | *Kushneria aurantia* |
|  |  | *Paenibacillus durus* |
|  |  | *Bacillus stratosphericus* |
|  |  | *Sediminibacillus albus* |
|  |  | *Melaminivora alkalimesophila* |
|  |  | *Cosenzaea myxofaciens* |
|  |  | *Anoxybacillus tepidamans* |
|  |  | *Anoxybacillus pushchinoensis* |
|  |  | *Thermovibrio ammonificans* |
|  |  | *Marinobacter lutaoensis* |
|  |  | *Muricauda zhangzhouensis* |
|  |  | *Flavobacterium psychrophilum* |
|  |  | *Oceanobacillus manasiensis* |
|  |  | *Thalassobacillus* sp. C254 |
|  |  | *Ochrobactrum* sp. P6BS-III |
|  |  | *Paraburkholderia* sp. SOS3 |
|  |  | *Virgibacillus chiguensis* |
|  |  | *Brevibacillus massiliensis* |
|  |  | *Weeksella* sp. FF8 |
|  |  | *Flexilinea flocculi* |
|  |  | *Paramesorhizobium deserti* |
| 3ds | 60 | *Paenibacillus gorillae* |
|  |  | *Streptococcus thoraltensis* |
|  |  | *Geobacillus* sp. LEMMY01 |
|  |  | *Youngiibacter fragilis* |
|  |  | *Streptococcus plurextorum* |
|  |  | *Enterococcus caccae* |
|  |  | *Clostridium amylolyticum* |
|  |  | *Lactobacillus sharpeae* |
|  |  | *Fructobacillus* sp. EFB-N1 |
|  |  | *Fusobacterium nucleatum* |
|  |  | *Streptococcus uberis* |
|  |  | *Alkalibacterium putridalgicola* |
|  |  | *Lactobacillus mali* |
|  |  | *Paenibacillus* sp. OV219 |
|  |  | *Mycobacterium asiaticum* |
|  |  | *Listeria floridensis* |
|  |  | *Staphylococcus pasteuri* |
|  |  | *Paenibacillus naphthalenovorans* |
|  |  | *Enterococcus plantarum* |
|  |  | *Acetivibrio ethanolgignens* |
|  |  | *Catonella morbi* |
|  |  | *Carnobacterium pleistocenium* |
|  |  | *Ruminiclostridium* sp. KB18 |
|  |  | *Staphylococcus saprophyticus* |
|  |  | *Lysinibacillus fusiformis* |
|  |  | *Streptococcus didelphis* |
|  |  | *Klebsiella variicola* |
|  |  | *Clostridium* sp. USBA 49 |
|  |  | *Carnobacterium divergens* |
|  |  | *Celeribacter baekdonensis* |
|  |  | *Lactobacillus coryniformis* |
|  |  | *Ignavigranum ruoffiae* |
|  |  | *Lactobacillus graminis* |
|  |  | *Enterococcus pseudoavium* |
|  |  | *Aneurinibacillus aneurinilyticus* |
|  |  | *Trichococcus collinsii* |
|  |  | *Lactobacillus cacaonum* |
|  |  | *Anaerotruncus rubiinfantis* |
|  |  | *Clostridium* sp. BL8 |
|  |  | *Enterococcus termitis* |
|  |  | *Streptococcus mitis* |
|  |  | *Streptococcus* sp. 'caviae' |
|  |  | *Pisciglobus halotolerans* |
|  |  | *Lactobacillus helveticus* |
|  |  | *Blautia hydrogenotrophica* |
|  |  | *Alkalibacterium subtropicum* |
|  |  | *Eubacterium limosum* |
|  |  | *Enterococcus hirae* |
|  |  | *Oenococcus oeni* |
|  |  | *Caloramator australicus* |
|  |  | *Campylobacter lari* |
|  |  | *Bacillus* sp. FJAT-27245 |
|  |  | *Leptotrichia trevisanii* |
|  |  | *Pedosphaera parvula* |
|  |  | *Anaerobium acetethylicum* |
|  |  | *Lactobacillus dextrinicus* |
|  |  | *Paenibacillus beijingensis* |
|  |  | *Anoxybacillus gonensis* |
|  |  | *Streptococcus infantis* |
|  |  | *Lachnobacterium bovis* |
| 4ds | 69 | *Cupriavidus* sp. YR651 |
|  |  | *Pseudoclavibacter faecalis* |
|  |  | *Vibrio harveyi* |
|  |  | *Stenotrophomonas daejeonensis* |
|  |  | *Ruegeria* sp. ZGT108 |
|  |  | *Ahrensia marina* |
|  |  | *Methyloversatilis discipulorum* |
|  |  | *Cupriavidus* sp. BIS7 |
|  |  | *Pseudomonas psychrotolerans* |
|  |  | *Psychrobacter* sp. JB385 |
|  |  | *Variovorax* sp. PAMC 28711 |
|  |  | *Halomonas* sp. 54_146 |
|  |  | *Thermobacillus composti* |
|  |  | *Thiomicrospira arctica* |
|  |  | *Thiohalocapsa* sp. ML1 |
|  |  | *Polaromonas* sp. OV174 |
|  |  | *Pantoea* sp. PSNIH1 |
|  |  | *Pseudomonas japonica* |
|  |  | *Novosphingobium tardaugens* |
|  |  | *Arthrobacter* sp. UCD-GKA |
|  |  | *Cupriavidus taiwanensis* |
|  |  | *Tatumella morbirosei* |
|  |  | *Legionella parisiensis* |
|  |  | *Rhodobacter* sp. 24-YEA-8 |
|  |  | *Vibrio hangzhouensis* |
|  |  | *Paenibacillus* sp. yr247 |
|  |  | *Polynucleobacter* sp. GWA2_45_21 |
|  |  | *Psychrobacter urativorans* |
|  |  | *Kaistia adipata* |
|  |  | *Burkholderia calidae* |
|  |  | *Tistlia consotensis* |
|  |  | *Burkholderia* sp. RPE67 |
|  |  | *Pedobacter* sp. Leaf41 |
|  |  | *Desulfuromonas acetoxidans* |
|  |  | *Azohydromonas lata* |
|  |  | *Halomonas chromatireducens* |
|  |  | *Marinobacter antarcticus* |
|  |  | *Microbacterium esteraromaticum* |
|  |  | *Geobacillus* sp. C56-T3 |
|  |  | *Aureimonas* sp. AU12 |
|  |  | *Pectobacterium parmentieri* |
|  |  | *Desulfovibrio desulfuricans* |
|  |  | *Hydrocarboniphaga daqingensis* |
|  |  | *Achromobacter* sp. ATCC31444 |
|  |  | *Acinetobacter* sp. 742879 |
|  |  | *Thauera* sp. 28 |
|  |  | *Rhizobium gallicum* |
|  |  | *Carboxydocella* sp. ULO1 |
|  |  | *Ferrovum* sp. PN-J185 |
|  |  | *Fructobacillus fructosus* |
|  |  | *Brevundimonas* sp. Root1279 |
|  |  | *Geoalkalibacter ferrihydriticus* |
|  |  | *Psychrobacter cryohalolentis* |
|  |  | *Halomonas* sp. BC04 |
|  |  | *Xylophilus* sp. Leaf220 |
|  |  | *Kroppenstedtia eburnea* |
|  |  | *Stenotrophomonas panacihumi* |
|  |  | *Thermithiobacillus tepidarius* |
|  |  | *Pseudomonas marincola* |
|  |  | *Pseudomonas saudimassiliensis* |
|  |  | *Shimia haliotis* |
|  |  | *Desulfovermiculus halophilus* |
|  |  | *Chitinimonas taiwanensis* |
|  |  | *Caballeronia grimmiae* |
|  |  | *Pandoraea sputorum* |
|  |  | *Alysiella crassa* |
|  |  | *Dokdonella immobilis* |
|  |  | *Nitrosomonas nitrosa* |
|  |  | *Syntrophorhabdus aromaticivorans* |
| 5ds | 130 | *Thioalkalivibrio* sp. ALJT |
|  |  | *Sphingobacterium* sp. ML3W |
|  |  | *Luteimonas abyssi* |
|  |  | *Nitratireductor pacificus* |
|  |  | *Flexithrix dorotheae* |
|  |  | *Sphingobacterium nematocida* |
|  |  | *Anaerosalibacter* sp. Marseille-P3206 |
|  |  | *Capnocytophaga* sp. oral taxon 329 |
|  |  | *Luteimonas huabeiensis* |
|  |  | *Lysobacter spongiicola* |
|  |  | *Sporocytophaga myxococcoides* |
|  |  | *Salinimicrobium catena* |
|  |  | *Serratia odorifera* |
|  |  | *Vaginella massiliensis* |
|  |  | *Myroides injenensis* |
|  |  | *Flavobacterium phragmitis* |
|  |  | *Flavobacterium daejeonense* |
|  |  | *Pedobacter africanus* |
|  |  | *Flavobacterium* sp. 40-81 |
|  |  | *Sinorhizobium* sp. LM21 |
|  |  | *Flavobacterium cauense* |
|  |  | *Niabella aurantiaca* |
|  |  | *Chitinophaga arvensicola* |
|  |  | *Acinetobacter* sp. NRRL B-65365 |
|  |  | *Flavobacterium* sp. 38-13 |
|  |  | *Flavobacterium antarcticum* |
|  |  | *Geobacillus* sp. 44C |
|  |  | *Pseudomonas veronii* |
|  |  | *Flavobacterium enshiense* |
|  |  | *Achromobacter* sp. NFACC18-2 |
|  |  | *Lysobacter defluvii* |
|  |  | *Adhaeribacter aquaticus* |
|  |  | *Flavobacterium fontis* |
|  |  | *Flavobacterium indicum* |
|  |  | *Pseudaminobacter salicylatoxidans* |
|  |  | *Mesonia phycicola* |
|  |  | *Thermonema rossianum* |
|  |  | *Bartonella koehlerae* |
|  |  | *Flavobacterium* sp. 316 |
|  |  | *Cellulophaga tyrosinoxydans* |
|  |  | *Oceanospirillum multiglobuliferum* |
|  |  | *Parapedobacter composti* |
|  |  | *Myroides phaeus* |
|  |  | *Solitalea canadensis* |
|  |  | *Flavobacterium* sp. MedPE-SWcel |
|  |  | *Mangrovimonas* sp. TPBH4 |
|  |  | *Parapedobacter koreensis* |
|  |  | *Psychroflexus halocasei* |
|  |  | *Parapedobacter indicus* |
|  |  | *Variovorax* sp. Root318D1 |
|  |  | *Snodgrassella* sp. R-53583 |
|  |  | *Sphingobacterium wenxiniae* |
|  |  | *Pedobacter soli* |
|  |  | *Ochrobactrum rhizosphaerae* |
|  |  | *Parageobacillus* genomosp. 1 |
|  |  | *Flavobacterium haoranii* |
|  |  | *Burkholderia latens* |
|  |  | *Ahrensia kielensis* |
|  |  | *Imtechella halotolerans* |
|  |  | *Rhizobium grahamii* |
|  |  | *Paracoccus solventivorans* |
|  |  | *Zhouia amylolytica* |
|  |  | *Sphingobacterium* sp. PM2-P1-29 |
|  |  | *Chryseobacterium* sp. J200 |
|  |  | *Myroides odoratimimus* |
|  |  | *Siansivirga zeaxanthinifaciens* |
|  |  | *Aliivibrio fischeri* |
|  |  | *Capnocytophaga canis* |
|  |  | *Formosa haliotis* |
|  |  | *Flavobacterium* sp. PK15 |
|  |  | *Psychroflexus tropicus* |
|  |  | *Flavobacterium succinicans* |
|  |  | *Pedobacter nyackensis* |
|  |  | *Chryseobacterium* sp. IHB B 17019 |
|  |  | *Sphingobacterium faecium* |
|  |  | *Ochrobactrum pseudogrignonense* |
|  |  | *Flavobacterium tegetincola* |
|  |  | *Geobacillus* sp. Y412MC61 |
|  |  | *Marinobacter subterrani* |
|  |  | *Chryseobacterium* sp. FH1 |
|  |  | *Mesorhizobium* sp. B7 |
|  |  | *Olivibacter sitiensis* |
|  |  | *Plesiomonas shigelloides* |
|  |  | *Achromobacter* sp. 2789STDY5608623 |
|  |  | *Aquimarina agarivorans* |
|  |  | *Psychrobacter* sp. SHUES1 |
|  |  | *Acinetobacter bereziniae* |
|  |  | *Flavobacterium akiainvivens* |
|  |  | *Meiothermus rufus* |
|  |  | *Cruoricaptor ignavus* |
|  |  | *Flavobacterium terrigena* |
|  |  | *Neorhizobium galegae* |
|  |  | *Flavobacterium seoulense* |
|  |  | *Tenacibaculum maritimum* |
|  |  | *Flavobacterium terrae* |
|  |  | *Flavobacterium gilvum* |
|  |  | *Pustulibacterium marinum* |
|  |  | *Niastella vici* |
|  |  | *Sphingobacterium* sp. IITKGP-BTPF85 |
|  |  | *Chryseobacterium* sp. G972 |
|  |  | *Gaetbulibacter saemankumensis* |
|  |  | *Flavobacterium aquatile* |
|  |  | *Flavobacterium limnosediminis* |
|  |  | *Chryseobacterium* sp. Leaf405 |
|  |  | *Chelonobacter oris* |
|  |  | *Elizabethkingia anophelis* |
|  |  | *Polynucleobacter asymbioticus* |
|  |  | *Flavobacterium noncentrifugens* |
|  |  | *Flavobacterium filum* |
|  |  | *Parapedobacter luteus* |
|  |  | *Algoriphagus* sp. NH1 |
|  |  | *Sphingobacterium* sp. CFCC 11742 |
|  |  | *Chryseobacterium gleum* |
|  |  | *Microvirga vignae* |
|  |  | *Chryseobacterium hungaricum* |
|  |  | *Flavobacterium subsaxonicum* |
|  |  | *Sphingobacterium deserti* |
|  |  | *Flavobacterium urocaniciphilum* |
|  |  | *Enterobacter asburiae* |
|  |  | *Paenibacillus* sp. FSL R7-0337 |
|  |  | *Flavobacterium beibuense* |
|  |  | *Chitinophaga niabensis* |
|  |  | *Capnocytophaga cynodegmi* |
|  |  | *Brenneria* sp. EniD312 |
|  |  | *Flavobacterium gelidilacus* |
|  |  | *Flavobacterium saliperosum* |
|  |  | *Brevundimonas* sp. SH203 |
|  |  | *Mariniphaga anaerophila* |
|  |  | *Bacillus plakortidis* |
|  |  | *Leadbetterella byssophila* |
| Sds | 20 | *Clostridium collagenovorans* |
|  |  | *Yaniella halotolerans* |
|  |  | *Enteractinococcus helveticum* |
|  |  | *Salinicoccus albus* |
|  |  | *Chromobacterium* sp. |
|  |  | *Gilliamella intestini* |
|  |  | *Bacillus cecembensis* |
|  |  | *Stenoxybacter acetivorans* |
|  |  | *Saccharomonospora viridis* |
|  |  | *Bacillus oryziterrae* |
|  |  | *Erwinia billingiae* |
|  |  | *Acinetobacter pittii* |
|  |  | *Virgibacillus salinus* |
|  |  | *Halomonas titanicae* |
|  |  | *Magnetovibrio blakemorei* |
|  |  | *Aneurinibacillus migulanus* |
|  |  | *Pontibacillus marinus* |
|  |  | *Bacillus* sp. FJAT-18017 |
|  |  | *Clostridium* sp. DL-VIII |
|  |  | *Coxiella burnetii* |
