# Supplementary material for: Shotgun sequence-based metataxonomic and predictive functional profiles of Pe poke, a naturally fermented soybean food of Myanmar
Source: PLoS One. 2021 Dec 17;16(12):e0260777. doi: 10.1371/journal.pone.0260777 (PMC8682898; doi:10.1371/journal.pone.0260777)
Supplement: S11 Table — (DOCX) [file pone.0260777.s011.docx]

**Supplementary Table 11.** Shared and unique archaeal species detected in *pe poke*.

| Sample Code | Number of species | Archaeal species |
| --- | --- | --- |
| 3ds, 5ds, Sds | 1 | *Methanobacterium formicicum* |
| 4ds, Sds | 1 | *Halapricum salinum* |
| 3ds | 3 | *Methanobrevibacter cuticularis* |
|  |  | *Halococcus hamelinensis* |
|  |  | *Pyrococcus horikoshii* |
| 4ds | 5 | *Natrialba* sp. SSL1 |
|  |  | *Methanoregula formicica* |
|  |  | *Halolamina pelagica* |
|  |  | *Halococcus salifodinae* |
|  |  | *Haloprofundus marisrubri* |
| 5ds | 2 | *Archaeoglobus fulgidus* |
|  |  | *Methanocaldococcus* sp. FS406-22 |
| Sds | 4 | *Natrialba asiatica* |
|  |  | *Ignisphaera aggregans* |
|  |  | *Methanobrevibacter filiformis* |
|  |  | *Halovenus aranensis* |
