# Supplementary material for: Shotgun sequence-based metataxonomic and predictive functional profiles of Pe poke, a naturally fermented soybean food of Myanmar
Source: PLoS One. 2021 Dec 17;16(12):e0260777. doi: 10.1371/journal.pone.0260777 (PMC8682898; doi:10.1371/journal.pone.0260777)
Supplement: S12 Table — (DOCX) [file pone.0260777.s012.docx]

**Supplementary Table 12.** Shared and unique eukaryotic species detected in *pe poke*.

| Sample Code | Number of species | Eukaryotic species |
| --- | --- | --- |
| 3ds, 4ds, 5ds, Sds | 1 | *Mucor ambiguus* |
| 3ds, Sds | 1 | *Batrachochytrium dendrobatidis* |
| 3ds | 2 | *Aspergillus calidoustus* |
|  |  | *Acanthamoeba castellanii* |
| 4ds | 6 | *Coniochaeta ligniaria* |
|  |  | *Talaromyces marneffei* |
|  |  | *Pseudocohnilembus persalinus* |
|  |  | *Dacryopinax primogenitus* |
|  |  | *Gonapodya prolifera* |
|  |  | *Coccomyxa subellipsoidea* |
| 5ds | 2 | *Angomonas desouzai* |
|  |  | *Endocarpon pusillum* |
| Sds | 19 | *Angomonas deanei* |
|  |  | *Phytophthora nicotianae* |
|  |  | *Mixia osmundae* |
|  |  | *Malassezia pachydermatis* |
|  |  | *Choanephora cucurbitarum* |
|  |  | *Zygosaccharomyces rouxii* |
|  |  | *Pseudocercospora musae* |
|  |  | *Aspergillus fumigatus* |
|  |  | *Thalassiosira pseudonana* |
|  |  | *Claviceps purpurea* |
|  |  | *Perkinsus marinus* |
|  |  | *Thalassiosira weissflogii* |
|  |  | *Rhizophagus irregularis* |
|  |  | *Guillardia theta* |
|  |  | *Puccinia striiformis* |
|  |  | *Acytostelium subglobosum* |
|  |  | *Tetrahymena thermophila* |
|  |  | *Encephalitozoon cuniculi* |
|  |  | *Bathycoccus prasinos* |
