# Supplementary material for: Shotgun sequence-based metataxonomic and predictive functional profiles of Pe poke, a naturally fermented soybean food of Myanmar
Source: PLoS One. 2021 Dec 17;16(12):e0260777. doi: 10.1371/journal.pone.0260777 (PMC8682898; doi:10.1371/journal.pone.0260777)
Supplement: S13 Table — (DOCX) [file pone.0260777.s013.docx]

**Supplementary Table 13.** Shared and unique viral species detected in *pe poke* samples.

| Sample Code | Number of species | Viral species |
| --- | --- | --- |
| 3ds, 4ds, Sds | 4 | *Bacillus* phage vB_BanS-Tsamsa |
|  |  | *Bacillus* phage Grass |
|  |  | *Bacillus* phage phiNIT1 |
|  |  | *Bacillus* phage PM1 |
| 3ds, 5ds, Sds | 1 | *Geobacillus* virus E3 |
| 3ds, 4ds | 5 | *Bacillus* phage Shbh1 |
|  |  | *Bacillus* phage SP-10 |
|  |  | *Bacillus* phage Mater |
|  |  | *Bacillus* phage SPG24 |
|  |  | *Bacillus* phage SIOphi |
| 4ds, 5ds | 1 | *Aeribacillus* phage AP45 |
| 3ds | 12 | *Bacillus* virus B103 |
|  |  | *Bacillus* phage BSNPO1 |
|  |  | *Bacillus* phage CampHawk |
|  |  | *Bacillus* phage Nf |
|  |  | *Bacillus* virus GA1 |
|  |  | *Bacillus* phage MG-B1 |
|  |  | *Bacillus* virus G |
|  |  | *Bacillus* phage SPP1 |
|  |  | *Ralstonia* phage RSP15 |
|  |  | *Bacillus* phage phi3T |
|  |  | *Bacillus* virus phi29 |
|  |  | *Bacillus* phage Harambe |
| 5ds | 4 | *Bacillus* phage Mgbh1 |
|  |  | *Staphylococcus* phage Twort |
|  |  | *Acinetobacter* phage IME-AB2 |
|  |  | *Rhizobium* phage RHEph10 |
| Sds | 6 | *Erwinia* phage Ea9-2 |
|  |  | *Escherichia* phage N4 |
|  |  | *Geobacillus* phage GBK2 |
|  |  | *Achromobacter* phage phiAxp-3 |
|  |  | *Delftia* phage RG-2014 |
|  |  | *Enterococcus* phage vB_EfaP_IME199 |
