# Supplementary material for: Shotgun sequence-based metataxonomic and predictive functional profiles of Pe poke, a naturally fermented soybean food of Myanmar
Source: PLoS One. 2021 Dec 17;16(12):e0260777. doi: 10.1371/journal.pone.0260777 (PMC8682898; doi:10.1371/journal.pone.0260777)
Supplement: S14 Table — (DOCX) [file pone.0260777.s014.docx]

**Supplementary Table 14.** Clusters of Orthologous Groups (COGs) detected in *pe poke* metagenome.

| Sl. No. | Level-2 | Relative abundance (%) | | | |
| --- | --- | --- | --- | --- | --- |
|  |  | 3ds | 4ds | 5ds | Sds |
| 1 | General function prediction only | 13.71316 | 13.16007 | 13.0551 | 12.93608 |
| 2 | DNA replication, recombination and repair | 11.04274 | 11.67102 | 10.57641 | 10.49457 |
| 3 | Amino acid transport and metabolism | 8.288296 | 8.766315 | 9.281035 | 9.454772 |
| 4 | Carbohydrate transport and metabolism | 9.232935 | 7.954451 | 6.849302 | 6.800326 |
| 5 | Transcription | 8.066897 | 7.472478 | 6.939039 | 6.855708 |
| 6 | Translation, ribosomal structure and biogenesis | 6.879286 | 7.113835 | 7.245447 | 7.383763 |
| 7 | Inorganic ion transport and metabolism | 6.695354 | 6.616729 | 6.952058 | 6.967329 |
| 8 | Energy production and conversion | 5.590626 | 6.340559 | 7.360757 | 7.520714 |
| 9 | Cell envelope biogenesis, outer membrane | 5.376039 | 5.555177 | 6.193246 | 6.140042 |
| 10 | Posttranslational modification, protein turnover, chaperones | 4.129388 | 4.172058 | 4.262274 | 4.288842 |
| 11 | Signal transduction mechanisms | 4.479086 | 4.259827 | 3.579715 | 3.512214 |
| 12 | Coenzyme metabolism | 3.310777 | 3.584913 | 4.246 | 4.262654 |
| 13 | Nucleotide transport and metabolism | 3.460648 | 3.204328 | 2.957135 | 2.992745 |
| 14 | Lipid metabolism | 3.056451 | 3.048462 | 3.116151 | 3.106083 |
| 15 | Replication, recombination and repair | 1.817748 | 2.028525 | 1.722671 | 1.691925 |
| 16 | Secondary metabolites biosynthesis, transport and catabolism | 1.167174 | 1.570764 | 2.183445 | 2.172326 |
| 17 | Cell division and chromosome partitioning | 1.536173 | 1.516286 | 1.479963 | 1.457519 |
| 18 | Cell motility and secretion | 1.337481 | 1.212878 | 1.043367 | 1.034646 |
| 19 | Cell wall/membrane/envelope biogenesis | 0.34175 | 0.314758 | 0.411488 | 0.394968 |
| 20 | Defense mechanisms | 0.244107 | 0.20429 | 0.196212 | 0.189327 |
| 21 | Lipid transport and metabolism | 0.133975 | 0.111981 | 0.135303 | 0.13137 |
| 22 | Intracellular trafficking, secretion, and vesicular transport | 0.063581 | 0.080203 | 0.136233 | 0.135234 |
| 23 | Cell motility | 0.036332 | 0.027995 | 0.020458 | 0.021036 |
| 24 | Coenzyme transport and metabolism | 0 | 0.00454 | 0.027898 | 0.027905 |
| 25 | Cell cycle control, cell division, chromosome partitioning | 0 | 0.007566 | 0.021388 | 0.020607 |
| 26 | Chromatin structure and dynamics | 0 | 0 | 0.007904 | 0.007298 |
