# Supplementary material for: Shotgun sequence-based metataxonomic and predictive functional profiles of Pe poke, a naturally fermented soybean food of Myanmar
Source: PLoS One. 2021 Dec 17;16(12):e0260777. doi: 10.1371/journal.pone.0260777 (PMC8682898; doi:10.1371/journal.pone.0260777)
Supplement: S15 Table — (DOCX) [file pone.0260777.s015.docx]

**Supplementary Table 15.** The relative abundance of <1% mapped against KEGG database at level-2 (Super-pathways).

| Sl. No. | Level-2 (Super-pathways) | Relative abundance | | | |
| --- | --- | --- | --- | --- | --- |
|  |  | 3ds | 4ds | 5ds | Sds |
| 1 | Transcription | 0.751055 | 0.85656 | 0.679423 | 0.707742 |
| 2 | Drug resistance: Antimicrobial | 0.368286 | 0.466349 | 0.630777 | 0.659368 |
| 3 | Infectious diseases: Bacterial | 0.331044 | 0.305914 | 0.354305 | 0.381035 |
| 4 | Aging | 0.142763 | 0.156356 | 0.160532 | 0.165214 |
| 5 | Transport and catabolism | 0.146901 | 0.154997 | 0.145938 | 0.139167 |
| 6 | Biosynthesis of other secondary metabolites | 0.10552 | 0.10605 | 0.134587 | 0.140655 |
| 7 | Endocrine system | 0.134486 | 0.10877 | 0.074591 | 0.071444 |
| 8 | Immune system | 0.111727 | 0.089735 | 0.088374 | 0.084096 |
| 9 | Neurodegenerative diseases | 0.120003 | 0.101971 | 0.06324 | 0.058792 |
| 10 | Digestive system | 0.04138 | 0.027192 | 0.044592 | 0.040931 |
| 11 | Cancers: Overview | 0 | 0.004079 | 0.030809 | 0.030513 |
| 12 | Environmental adaptation | 0.004138 | 0.008158 | 0.023512 | 0.021582 |
| 13 | Cellular community - eukaryotes | 0.004138 | 0.002719 | 0.001622 | 0.002233 |
| 14 | Nervous system | 0.002069 | 0.00136 | 0.001622 | 0.002233 |
| 15 | Infectious diseases: Parasitic | 0 | 0 | 0.003243 | 0.002977 |
