# Supplementary material for: Shotgun sequence-based metataxonomic and predictive functional profiles of Pe poke, a naturally fermented soybean food of Myanmar
Source: PLoS One. 2021 Dec 17;16(12):e0260777. doi: 10.1371/journal.pone.0260777 (PMC8682898; doi:10.1371/journal.pone.0260777)
Supplement: S16 Table — (DOCX) [file pone.0260777.s016.docx]

**Supplementary Table 16.** The relative abundance of <1% mapped against KEGG database at level-3 (Sub-pathways).

| Sl. No | Level-3 (Sub-pathways) | Relative abundance | | | |
| --- | --- | --- | --- | --- | --- |
|  |  | 3ds | 4ds | 5ds | Sds |
| 1 | Phenylalanine, tyrosine and tryptophan biosynthesis | 0.860607 | 0.910871 | 1.008488 | 1.016475 |
| 2 | Glycerolipid metabolism | 1.119202 | 0.921747 | 0.811492 | 0.812585 |
| 3 | Arginine and proline metabolism | 0.862675 | 0.920387 | 0.907963 | 0.910065 |
| 4 | Nicotinate and nicotinamide metabolism | 0.7551 | 0.765403 | 0.99957 | 0.991175 |
| 5 | Base excision repair | 0.802681 | 0.85649 | 0.929041 | 0.915274 |
| 6 | Fructose and mannose metabolism | 1.278497 | 0.977487 | 0.634764 | 0.60795 |
| 7 | Nitrogen metabolism | 0.904051 | 0.933982 | 0.796089 | 0.797702 |
| 8 | Ubiquinone and other terpenoid-quinone biosynthesis | 0.678555 | 0.789874 | 0.943633 | 0.932389 |
| 9 | Riboflavin metabolism | 0.802681 | 0.765403 | 0.716642 | 0.683107 |
| 10 | RNA polymerase | 0.688899 | 0.815705 | 0.655031 | 0.685339 |
| 11 | Mismatch repair | 0.674418 | 0.701506 | 0.646113 | 0.64218 |
| 12 | Pantothenate and CoA biosynthesis | 0.697174 | 0.625374 | 0.64287 | 0.627297 |
| 13 | Pentose and glucuronate interconversions | 0.773718 | 0.705585 | 0.55937 | 0.551397 |
| 14 | Phenylalanine metabolism | 0.208945 | 0.474468 | 0.940391 | 0.928668 |
| 15 | Selenocompound metabolism | 0.558567 | 0.598184 | 0.566666 | 0.565535 |
| 16 | Histidine metabolism | 0.461335 | 0.492142 | 0.620171 | 0.657807 |
| 17 | Lipopolysaccharide biosynthesis | 0.039307 | 0.243352 | 0.903099 | 0.913042 |
| 18 | Inositol phosphate metabolism | 0.647524 | 0.532927 | 0.415069 | 0.400339 |
| 19 | Benzoate degradation | 0.229633 | 0.345315 | 0.715021 | 0.690548 |
| 20 | Methane metabolism | 0.426166 | 0.488064 | 0.493705 | 0.500796 |
| 21 | Arginine biosynthesis | 0.432372 | 0.463592 | 0.501812 | 0.505261 |
| 22 | Bacterial secretion system | 0.126195 | 0.305889 | 0.573152 | 0.586371 |
| 23 | Sulfur relay system | 0.409616 | 0.373865 | 0.357511 | 0.346762 |
| 24 | Polyketide sugar unit biosynthesis | 0.36824 | 0.383381 | 0.374535 | 0.35718 |
| 25 | Biotin metabolism | 0.211014 | 0.269183 | 0.412637 | 0.407036 |
| 26 | Bacterial chemotaxis | 0.38479 | 0.342596 | 0.263472 | 0.261188 |
| 27 | RNA transport | 0.349621 | 0.345315 | 0.275632 | 0.261188 |
| 28 | Tyrosine metabolism | 0.047582 | 0.163141 | 0.500191 | 0.497076 |
| 29 | Cationic antimicrobial peptide (CAMP) resistance | 0.331003 | 0.300451 | 0.284549 | 0.289465 |
| 30 | Glutathione metabolism | 0.097232 | 0.225678 | 0.426418 | 0.45094 |
| 31 | D-Glutamine and D-glutamate metabolism | 0.295834 | 0.305889 | 0.269146 | 0.270862 |
| 32 | Valine, leucine and isoleucine degradation | 0.182051 | 0.271902 | 0.332379 | 0.325183 |
| 33 | Fatty acid degradation | 0.208945 | 0.209364 | 0.302384 | 0.311044 |
| 34 | Biofilm formation - Escherichia coli | 0.107576 | 0.188972 | 0.335622 | 0.320718 |
| 35 | Vitamin B6 metabolism | 0.190326 | 0.203926 | 0.268336 | 0.267141 |
| 36 | beta-Lactam resistance | 0.037238 | 0.16586 | 0.346161 | 0.36983 |
| 37 | Protein processing in endoplasmic reticulum | 0.198602 | 0.241992 | 0.209967 | 0.209843 |
| 38 | Lipoic acid metabolism | 0.21722 | 0.218881 | 0.201049 | 0.20389 |
| 39 | D-Alanine metabolism | 0.219289 | 0.218881 | 0.192942 | 0.192728 |
| 40 | C5-Branched dibasic acid metabolism | 0.148951 | 0.19305 | 0.229423 | 0.247794 |
| 41 | Carbon fixation pathways in prokaryotes | 0.219289 | 0.183534 | 0.200238 | 0.20389 |
| 42 | Aminobenzoate degradation | 0.109645 | 0.153624 | 0.269957 | 0.267885 |
| 43 | Phosphotransferase system (PTS) | 0.215152 | 0.183534 | 0.172675 | 0.168916 |
| 44 | Biofilm formation - Pseudomonas aeruginosa | 0.144814 | 0.175377 | 0.207534 | 0.201658 |
| 45 | Valine, leucine and isoleucine biosynthesis | 0.148951 | 0.160422 | 0.187267 | 0.198681 |
| 46 | MAPK signaling pathway - plant | 0.159295 | 0.176736 | 0.162947 | 0.162219 |
| 47 | Apoptosis | 0.153089 | 0.134591 | 0.17835 | 0.172637 |
| 48 | Longevity regulating pathway - multiple species | 0.142745 | 0.156343 | 0.160515 | 0.165196 |
| 49 | Biosynthesis of siderophore group nonribosomal peptides | 0.064132 | 0.125075 | 0.211588 | 0.203146 |
| 50 | Phosphonate and phosphinate metabolism | 0.103438 | 0.133232 | 0.179161 | 0.176358 |
| 51 | One carbon pool by folate | 0.142745 | 0.13731 | 0.149165 | 0.154034 |
| 52 | Arabinogalactan biosynthesis - Mycobacterium | 0.159295 | 0.164501 | 0.120792 | 0.118316 |
| 53 | Epithelial cell signaling in Helicobacter pylori infection | 0.177914 | 0.141389 | 0.10701 | 0.116828 |
| 54 | Ribosome biogenesis in eukaryotes | 0.111713 | 0.133232 | 0.138627 | 0.145849 |
| 55 | beta-Alanine metabolism | 0.078613 | 0.112839 | 0.162947 | 0.159243 |
| 56 | Ethylbenzene degradation | 0 | 0.063897 | 0.225369 | 0.217285 |
| 57 | Legionellosis | 0.113782 | 0.129153 | 0.127277 | 0.130222 |
| 58 | Other glycan degradation | 0.182051 | 0.13731 | 0.08269 | 0.08111 |
| 59 | Lysine degradation | 0.053788 | 0.076132 | 0.158083 | 0.171893 |
| 60 | Primary bile acid biosynthesis | 0.124126 | 0.108761 | 0.107821 | 0.109386 |
| 61 | Secondary bile acid biosynthesis | 0.113782 | 0.106042 | 0.112685 | 0.111619 |
| 62 | Biofilm formation - Vibrio cholerae | 0.109645 | 0.104682 | 0.111063 | 0.107898 |
| 63 | Zeatin biosynthesis | 0.095163 | 0.106042 | 0.103767 | 0.110131 |
| 64 | Nonribosomal peptide structures | 0.13447 | 0.129153 | 0.077015 | 0.07218 |
| 65 | Taurine and hypotaurine metabolism | 0.068269 | 0.092447 | 0.124034 | 0.121292 |
| 66 | Steroid degradation | 0.04965 | 0.069335 | 0.14268 | 0.133943 |
| 67 | Carotenoid biosynthesis | 0.059994 | 0.063897 | 0.134573 | 0.129478 |
| 68 | NOD-like receptor signaling pathway | 0.107576 | 0.087009 | 0.086743 | 0.082598 |
| 69 | Caprolactam degradation | 0.119988 | 0.108761 | 0.065665 | 0.065483 |
| 70 | Alzheimer disease | 0.119988 | 0.101963 | 0.063233 | 0.058786 |
| 71 | MAPK signaling pathway - fly | 0.084819 | 0.081571 | 0.085122 | 0.092272 |
| 72 | Autophagy - yeast | 0.078613 | 0.092447 | 0.068908 | 0.066227 |
| 73 | Biosynthesis of unsaturated fatty acids | 0.008275 | 0.042145 | 0.104578 | 0.102689 |
| 74 | Peroxisome | 0.064132 | 0.059818 | 0.067287 | 0.063251 |
| 75 | D-Arginine and D-ornithine metabolism | 0.082751 | 0.077492 | 0.046209 | 0.043903 |
| 76 | Insulin signaling pathway | 0.093094 | 0.067975 | 0.042966 | 0.042415 |
| 77 | Ascorbate and aldarate metabolism | 0.059994 | 0.051661 | 0.064855 | 0.062507 |
| 78 | Salmonella infection | 0.018619 | 0.021752 | 0.089175 | 0.093016 |
| 79 | Tryptophan metabolism | 0 | 0.004079 | 0.105389 | 0.102689 |
| 80 | Steroid hormone biosynthesis | 0.055857 | 0.044864 | 0.055937 | 0.052089 |
| 81 | cAMP signaling pathway | 0.072407 | 0.057099 | 0.034049 | 0.031253 |
| 82 | Tropane, piperidine and pyridine alkaloid biosynthesis | 0.055857 | 0.053021 | 0.032427 | 0.033486 |
| 83 | Glycosaminoglycan degradation | 0.057925 | 0.043504 | 0.029995 | 0.029021 |
| 84 | Chlorocyclohexane and chlorobenzene degradation | 0 | 0.038066 | 0.057558 | 0.058042 |
| 85 | Spliceosome | 0.062063 | 0.040785 | 0.02432 | 0.022324 |
| 86 | Renin-angiotensin system | 0.041375 | 0.040785 | 0.031617 | 0.029021 |
| 87 | Penicillin and cephalosporin biosynthesis | 0.031031 | 0.031269 | 0.038913 | 0.040183 |
| 88 | Styrene degradation | 0 | 0.021752 | 0.055937 | 0.055065 |
| 89 | Limonene and pinene degradation | 0.04965 | 0.036707 | 0.021888 | 0.020091 |
| 90 | Furfural degradation | 0 | 0.021752 | 0.050262 | 0.049856 |
| 91 | Dioxin degradation | 0 | 0.012236 | 0.051884 | 0.049112 |
| 92 | Mineral absorption | 0.041375 | 0.02719 | 0.017024 | 0.015627 |
| 93 | N-Glycan biosynthesis | 0.035169 | 0.023112 | 0.020267 | 0.018603 |
| 94 | Fluorobenzoate degradation | 0 | 0.013595 | 0.041345 | 0.040927 |
| 95 | Chloroalkane and chloroalkene degradation | 0.039307 | 0.025831 | 0.015403 | 0.014883 |
| 96 | Arachidonic acid metabolism | 0.031031 | 0.02855 | 0.017835 | 0.017115 |
| 97 | Pertussis | 0.012413 | 0.008157 | 0.025942 | 0.033486 |
| 98 | Nitrotoluene degradation | 0.010344 | 0.006798 | 0.027563 | 0.033486 |
| 99 | Betalain biosynthesis | 0 | 0.004079 | 0.033238 | 0.037206 |
| 100 | Biosynthesis of secondary metabolites - unclassified | 0.018619 | 0.016314 | 0.018646 | 0.018603 |
| 101 | Xylene degradation | 0 | 0.012236 | 0.029995 | 0.027533 |
| 102 | Viral carcinogenesis | 0 | 0.004079 | 0.030806 | 0.030509 |
| 103 | Photosynthesis | 0.018619 | 0.019033 | 0.012971 | 0.011906 |
| 104 | Thermogenesis | 0.004138 | 0.008157 | 0.02351 | 0.02158 |
| 105 | Biosynthesis of vancomycin group antibiotics | 0 | 0.008157 | 0.02432 | 0.022324 |
| 106 | Protein digestion and absorption | 0 | 0 | 0.027563 | 0.0253 |
| 107 | Type I polyketide structures | 0.01655 | 0.012236 | 0.01216 | 0.011162 |
| 108 | Sphingolipid metabolism | 0.018619 | 0.012236 | 0.008917 | 0.008185 |
| 109 | Ubiquitin mediated proteolysis | 0 | 0.006798 | 0.021078 | 0.019347 |
| 110 | Biosynthesis of type II polyketide products | 0.004138 | 0.002719 | 0.019456 | 0.017859 |
| 111 | Polycyclic aromatic hydrocarbon degradation | 0.004138 | 0.008157 | 0.015403 | 0.014883 |
| 112 | Necroptosis | 0.008275 | 0.005438 | 0.008917 | 0.00893 |
| 113 | Bacterial invasion of epithelial cells | 0.008275 | 0.005438 | 0.004864 | 0.007441 |
| 114 | Phenylpropanoid biosynthesis | 0 | 0.00136 | 0.010539 | 0.010418 |
| 115 | Retinol metabolism | 0 | 0.002719 | 0.009728 | 0.00893 |
| 116 | Lysosome | 0.002069 | 0.00136 | 0.008917 | 0.00893 |
| 117 | Geraniol degradation | 0 | 0.005438 | 0.007296 | 0.007441 |
| 118 | Bisphenol degradation | 0 | 0 | 0.007296 | 0.006697 |
| 119 | Signaling pathways regulating pluripotency of stem cells | 0.004138 | 0.002719 | 0.001621 | 0.002232 |
| 120 | RIG-I-like receptor signaling pathway | 0.004138 | 0.002719 | 0.001621 | 0.001488 |
| 121 | Glutamatergic synapse | 0.002069 | 0.00136 | 0.001621 | 0.002232 |
| 122 | Chagas disease (American trypanosomiasis) | 0 | 0 | 0.003243 | 0.002977 |
| 123 | Steroid biosynthesis | 0 | 0 | 0.003243 | 0.002977 |
| 124 | Endocytosis | 0.002069 | 0.00136 | 0.000811 | 0.000744 |
| 125 | Biosynthesis of enediyne antibiotics | 0 | 0.00136 | 0.001621 | 0.001488 |
| 126 | Drug metabolism - other enzymes | 0 | 0 | 0.001621 | 0.001488 |
| 127 | Plant hormone signal transduction | 0 | 0 | 0.000811 | 0.000744 |
| 128 | Proteasome | 0 | 0 | 0.000811 | 0.000744 |
| 129 | Phenazine biosynthesis | 0 | 0 | 0.000811 | 0.000744 |
| 130 | Lipoarabinomannan (LAM) biosynthesis | 0 | 0 | 0.000811 | 0.000744 |
| 131 | Ether lipid metabolism | 0 | 0 | 0.000811 | 0.000744 |
| 132 | Biosynthesis of type II polyketide backbone | 0 | 0 | 0.000811 | 0.000744 |
| 133 | Atrazine degradation | 0 | 0 | 0.000811 | 0.000744 |
| 134 | Apelin signaling pathway | 0 | 0 | 0 | 0.000744 |
| 135 | Calcium signaling pathway | 0 | 0 | 0 | 0.000744 |
| 136 | Carbon fixation in photosynthetic organisms | 0 | 0 | 0 | 0.000744 |
