# Supplementary material for: Shotgun sequence-based metataxonomic and predictive functional profiles of Pe poke, a naturally fermented soybean food of Myanmar
Source: PLoS One. 2021 Dec 17;16(12):e0260777. doi: 10.1371/journal.pone.0260777 (PMC8682898; doi:10.1371/journal.pone.0260777)
Supplement: S17 Table — (DOCX) [file pone.0260777.s017.docx]

**Supplementary Table 17.** Predictive enzyme classification detected in *pe poke*

| **Protease** | | | | | | | | | | |
| --- | --- | --- | --- | --- | --- | --- | --- | --- | --- | --- |
| KO IDs | Functions | | Occurrence (%) | | | | | | | |
|  |  |  | 3ds | | | 4ds | | | 5ds | Sds |
| K03544 | ATP-dependent Clp protease ATP-binding subunit ClpX | | 0.098312 | | | 0.111601 | | | 0.085712 | 0.088612 |
| K01338 | ATP-dependent Lon protease [EC:3.4.21.53] | | 0.091758 | | | 0.146273 | | | 0.135438 | 0.141056 |
| K11749 | regulator of sigma E protease [EC:3.4.24.-] | | 0.077011 | | | 0.080179 | | | 0.071972 | 0.074145 |
| K01358 | ATP-dependent Clp protease, protease subunit [EC:3.4.21.92] | | 0.311322 | | | 0.252457 | | | 0.157029 | 0.15251 |
| K12536 | ATP-binding cassette, subfamily C, bacterial exporter for protease/lipase | | 0 | | | 0 | | | 0.000654 | 0.000603 |
| K10913 | TetR/AcrR family transcriptional regulator, hemagglutinin/protease regulatory protein | | 0.001639 | | | 0.001084 | | | 0.000654 | 0.000603 |
| K08303 | putative protease [EC:3.4.-.-] | | 0.140914 | | | 0.112685 | | | 0.086366 | 0.09464 |
| K06891 | ATP-dependent Clp protease adaptor protein ClpS | | 0 | | | 0.015169 | | | 0.032714 | 0.031346 |
| K04088 | membrane protease subunit HflK [EC:3.4.-.-] | | 0.036048 | | | 0.044424 | | | 0.043183 | 0.044608 |
| K04087 | membrane protease subunit HflC [EC:3.4.-.-] | | 0.031132 | | | 0.039006 | | | 0.03206 | 0.03014 |
| K21511 | capsid assembly protease [EC:3.4.21.-] | | 0 | | | 0 | | | 0.002617 | 0.002411 |
| K03695 | ATP-dependent Clp protease ATP-binding subunit ClpB | | 0.113059 | | | 0.124603 | | | 0.129549 | 0.133823 |
| **Serine Protease** | | | | | | | | | | |
| KO IDs | Functions | Occurrence (%) | | | | | | | | |
|  |  | 3ds | | | | 4ds | | | 5ds | Sds |
| K14645 | serine protease [EC:3.4.21.-] | 0.016385 | | | | 0.016253 | | | 0.009814 | 0.009042 |
| K04771 | serine protease Do [EC:3.4.21.107] | 0.155661 | | | | 0.151691 | | | 0.110575 | 0.106697 |
| K07403 | membrane-bound serine protease (ClpP class) | 0.037686 | | | | 0.039006 | | | 0.028134 | 0.028935 |
| **Lipase** | | | | | | | | | | |
| KO IDs | Functions | Occurrence (%) | | | | | | | | |
|  |  | 3ds | | | | 4ds | | | 5ds | Sds |
| K12536 | ATP-binding cassette, subfamily C, bacterial exporter for protease/lipase | 0 | | | | 0 | | | 0.000654 | 0.000603 |
| K01114 | phospholipase C [EC:3.1.4.3] | 0 | | | | 0.014086 | | | 0.0229 | 0.023509 |
| K01046 | triacylglycerol lipase [EC:3.1.1.3] | 0.036048 | | | | 0.031422 | | | 0.018974 | 0.018687 |
| K01054 | acylglycerol lipase [EC:3.1.1.23] | 0.013108 | | | | 0.008668 | | | 0.005234 | 0.004822 |
| K01048 | lysophospholipase [EC:3.1.1.5] | 0.031132 | | | | 0.026004 | | | 0.015703 | 0.01507 |
| K01058 | phospholipase A1/A2 [EC:3.1.1.32 3.1.1.4] | 0 | | | | 0 | | | 0.01374 | 0.012659 |
| K19311 | lipase [EC:3.1.1.-] | 0 | | | | 0 | | | 0.000654 | 0.000603 |
| K06999 | phospholipase/carboxylesterase | 0.037686 | | | | 0.042257 | | | 0.060194 | 0.059678 |
| **Amylase** | | | | | | | | | | |
| KO IDs | Functions | Occurrence (%) | | | | | | | | |
|  |  | 3ds | | | | 4ds | | | 5ds | Sds |
| K01208 | cyclomaltodextrinase / maltogenic alpha-amylase / neopullulanase [EC:3.2.1.54 3.2.1.133 3.2.1.135] | 0.049156 | | | | 0.047674 | | | 0.028789 | 0.026523 |
| K01178 | glucoamylase [EC:3.2.1.3] | 0 | | | | 0 | | | 0.001963 | 0.001808 |
| K01176 | alpha-amylase [EC:3.2.1.1] | 0.034409 | | | | 0.022754 | | | 0.015049 | 0.013865 |
| **γ-PGA protein** | | | | | | | | | | |
| KO IDs | Functions | | | | Occurrence (%) | | | | | |
|  |  |  |  |  | 3ds | 4ds | | | 5ds | Sds |
| K22116 | gamma-polyglutamate biosynthesis protein CapC | | | | 0.001639 | 0.001084 | | | 0.000654 | 0.000603 |
| K07282 | gamma-polyglutamate biosynthesis protein CapA | | | | 0.008193 | 0.007585 | | | 0.00458 | 0.006028 |
| K01932 | gamma-polyglutamate synthase [EC:6.3.2.-] | | | | 0.006554 | 0.004334 | | | 0.002617 | 0.002411 |
| **Glucosidase** | | | | | | | | | | |
| KO IDs | Functions | | | | Occurrence (%) | | | | | |
|  |  |  |  |  | 3ds | | 4ds | | 5ds | Sds |
| K01187 | alpha-glucosidase [EC:3.2.1.20] | | | | 0.085204 | | 0.068261 | | 0.044492 | 0.043402 |
| K01182 | oligo-1,6-glucosidase [EC:3.2.1.10] | | | | 0.057349 | | 0.054175 | | 0.032714 | 0.033154 |
| K01223; K01222 | 6-phospho-beta-glucosidase [EC:3.2.1.86] | | | | 0.444044 | | 0.352139 | | 0.212643 | 0.203748 |
| K01232 | maltose-6'-phosphate glucosidase [EC:3.2.1.122] | | | | 0.032771 | | 0.02167 | | 0.013086 | 0.014467 |
| K05349; K05350 | beta-glucosidase [EC:3.2.1.21] | | | | 0.152384 | | 0.133271 | | 0.081132 | 0.077159 |
| K01215 | glucan 1,6-alpha-glucosidase [EC:3.2.1.70] | | | | 0.014747 | | 0.009752 | | 0.005889 | 0.005425 |
| **Galactosidase** | | | | | | | | | | |
| KO IDs | Functions | | | Occurrence (%) | | | | | | |
|  |  |  |  | 3ds | | | | 4ds | 5ds | Sds |
| K07407; K07406 | alpha-galactosidase [EC:3.2.1.22] | | | 0.306407 | | | | 0.277377 | 0.167498 | 0.158538 |
| K12308 | beta-galactosidase [EC:3.2.1.23] | | | 0.178601 | | | | 0.121353 | 0.075897 | 0.072337 |
| K12111 | evolved beta-galactosidase subunit alpha [EC:3.2.1.23] | | | 0.040963 | | | | 0.027088 | 0.016357 | 0.01507 |
| K01224 | arabinogalactan endo-1,4-beta-galactosidase [EC:3.2.1.89] | | | 0.049156 | | | | 0.036839 | 0.022246 | 0.020495 |
| **Glutamate decarboxylase** | | | | | | | | | | |
| KO IDs | Functions | | | Occurrence (%) | | | | | | |
|  |  |  |  | 3ds | | | | 4ds | 5ds | Sds |
| K01580 | glutamate decarboxylase [EC:4.1.1.15] | | | 0 | | | | 0.010835 | 0.029443 | 0.028332 |
