# Supplementary material for: Shotgun sequence-based metataxonomic and predictive functional profiles of Pe poke, a naturally fermented soybean food of Myanmar
Source: PLoS One. 2021 Dec 17;16(12):e0260777. doi: 10.1371/journal.pone.0260777 (PMC8682898; doi:10.1371/journal.pone.0260777)
Supplement: S18 Table — (DOCX) [file pone.0260777.s018.docx]

**Supplementary Table 18.** Predictive enzymes classification involved in different pathways in *pe poke*: (a) Lysine biosynthesis; (b) Biosynthesis of alanine, aspartate and glutamate metabolism and 4-aminobutanoic acid (GABA); (c) Glycine, serine, threonine metabolism and ectoine biosynthesis; (d) Pentose phosphate pathways and (e) Galactose metabolism

| **(a**) **Lysine biosynthesis** | | | | | |
| --- | --- | --- | --- | --- | --- |
| KO IDs | Functions | 3ds | 4ds | 5ds | Sds |
| K01439 | succinyl-diaminopimelate desuccinylase [EC:3.5.1.18] | 0.14583 | 0.127854 | 0.077206 | 0.072337 |
| K01714 | 4-hydroxy-tetrahydrodipicolinate synthase [EC:4.3.3.7] | 0.057349 | 0.078012 | 0.10992 | 0.110916 |
| K00003 | homoserine dehydrogenase [EC:1.1.1.3] | 0.085204 | 0.081263 | 0.073934 | 0.075953 |
| K01928 | UDP-N-acetylmuramoyl-L-alanyl-D-glutamate--2,6-diaminopimelate ligase [EC:6.3.2.13] | 0.093397 | 0.088847 | 0.066083 | 0.063897 |
| K01928 | UDP-N-acetylmuramoyl-L-alanyl-D-glutamate--2,6-diaminopimelate ligase [EC:6.3.2.13] | 0.093397 | 0.088847 | 0.066083 | 0.063897 |
| K00821 | acetylornithine/N-succinyldiaminopimelate aminotransferase [EC:2.6.1.11 2.6.1.17] | 0.054072 | 0.056342 | 0.088983 | 0.086201 |
| K01929 | UDP-N-acetylmuramoyl-tripeptide--D-alanyl-D-alanine ligase [EC:6.3.2.10] | 0.075373 | 0.071511 | 0.05954 | 0.060883 |
| K00841 | aminotransferase [EC:2.6.1.-] | 0.07865 | 0.063927 | 0.04122 | 0.040388 |
| K01586 | diaminopimelate decarboxylase [EC:4.1.1.20] | 0.042602 | 0.055259 | 0.049726 | 0.050636 |
| K00215 | 4-hydroxy-tetrahydrodipicolinate reductase [EC:1.17.1.8] | 0.039325 | 0.041173 | 0.056269 | 0.059678 |
| K00133 | aspartate-semialdehyde dehydrogenase [EC:1.2.1.11] | 0.034409 | 0.042257 | 0.056923 | 0.059075 |
| K10206 | LL-diaminopimelate aminotransferase [EC:2.6.1.83] | 0.062264 | 0.045507 | 0.028789 | 0.028935 |
| K05823 | N-acetyldiaminopimelate deacetylase [EC:3.5.1.47] | 0.05571 | 0.044424 | 0.026826 | 0.025921 |
| K05825 | 2-aminoadipate transaminase [EC:2.6.1.-] | 0.004916 | 0.01842 | 0.051689 | 0.050636 |
| K01778 | diaminopimelate epimerase [EC:5.1.1.7] | 0.039325 | 0.031422 | 0.020937 | 0.021098 |
| K00290 | saccharopine dehydrogenase (NAD+, L-lysine forming) [EC:1.5.1.7] | 0.026217 | 0.035756 | 0.021591 | 0.021098 |
| K03340 | diaminopimelate dehydrogenase [EC:1.4.1.16] | 0.001639 | 0.005418 | 0.043837 | 0.047019 |
| K05822 | tetrahydrodipicolinate N-acetyltransferase [EC:2.3.1.89] | 0.037686 | 0.027088 | 0.016357 | 0.015673 |
| K00674 | 2,3,4,5-tetrahydropyridine-2,6-dicarboxylate N-succinyltransferase [EC:2.3.1.117] | 0 | 0 | 0.002617 | 0.002411 |
| K00293 | saccharopine dehydrogenase (NADP+, L-glutamate forming) [EC:1.5.1.10] | 0 | 0 | 0.001963 | 0.001808 |

| **(b)** **Biosynthesis of alanine, aspartate and glutamate metabolism and 4-aminobutanoic acid (GABA)** | | | | | |
| --- | --- | --- | --- | --- | --- |
| KO IDs | Functions | 3ds | 4ds | 5ds | Sds |
| K01955 | carbamoyl-phosphate synthase large subunit [EC:6.3.5.5] | 0.249058 | 0.282795 | 0.230964 | 0.242328 |
| K13566 | asparagine synthase (glutamine-hydrolysing) [EC:6.3.5.4] | 0.226118 | 0.184196 | 0.120389 | 0.119355 |
| K00820 | glucosamine---fructose-6-phosphate aminotransferase (isomerizing) [EC:2.6.1.16] | 0.131083 | 0.153858 | 0.139363 | 0.139248 |
| K00135 | succinate-semialdehyde dehydrogenase / glutarate-semialdehyde dehydrogenase [EC:1.2.1.16 1.2.1.79 1.2.1.20] | 0.040963 | 0.085597 | 0.14656 | 0.16758 |
| K01424 | L-asparaginase [EC:3.5.1.1] | 0.126167 | 0.10185 | 0.102069 | 0.095243 |
| K01956 | carbamoyl-phosphate synthase small subunit [EC:6.3.5.5] | 0.103228 | 0.112685 | 0.080477 | 0.078968 |
| K01756 | adenylosuccinate lyase [EC:4.3.2.2] | 0.083565 | 0.075845 | 0.098797 | 0.110916 |
| K00265 | glutamate synthase (NADPH) large chain [EC:1.4.1.13] | 0.101589 | 0.102933 | 0.066083 | 0.063897 |
| K01915 | glutamine synthetase [EC:6.3.1.2] | 0.077011 | 0.082346 | 0.085712 | 0.088612 |
| K00262 | glutamate dehydrogenase (NADP+) [EC:1.4.1.4] | 0.086843 | 0.095349 | 0.074589 | 0.075351 |
| K01940 | argininosuccinate synthase [EC:6.3.4.5] | 0.063903 | 0.08668 | 0.088329 | 0.091024 |
| K00294 | 1-pyrroline-5-carboxylate dehydrogenase [EC:1.2.1.88] | 0.091758 | 0.097516 | 0.066083 | 0.066911 |
| K01755 | argininosuccinate lyase [EC:4.3.2.1] | 0.062264 | 0.081263 | 0.084403 | 0.087407 |
| K01939 | adenylosuccinate synthase [EC:6.3.4.4] | 0.070457 | 0.074762 | 0.081786 | 0.084393 |
| K00764 | amidophosphoribosyltransferase [EC:2.4.2.14] | 0.072096 | 0.06176 | 0.06412 | 0.065103 |
| K00278 | L-aspartate oxidase [EC:1.4.3.16] | 0.080288 | 0.081263 | 0.05038 | 0.046416 |
| K00259 | alanine dehydrogenase [EC:1.4.1.1] | 0.080288 | 0.060676 | 0.039912 | 0.042196 |
| K00261 | glutamate dehydrogenase [EC:1.4.1.2] | 0.054072 | 0.049841 | 0.030097 | 0.033757 |
| K01744 | aspartate ammonia-lyase [EC:4.3.1.1] | 0.006554 | 0.020587 | 0.060849 | 0.063295 |
| K01953 | omega-amidase [EC:3.5.1.3] | 0.044241 | 0.046591 | 0.029443 | 0.028332 |
| K00812 | aspartate aminotransferase [EC:2.6.1.1] | 0.044241 | 0.037923 | 0.028789 | 0.03014 |
| K00260 | glutamate dehydrogenase (NAD(P)+) [EC:1.4.1.3] | 0 | 0.017336 | 0.039257 | 0.037374 |
| K14260 | alanine-synthesizing transaminase [EC:2.6.1.66 2.6.1.2] | 0.037686 | 0.024921 | 0.015049 | 0.013865 |
| K01580 | glutamate decarboxylase [EC:4.1.1.15] | 0 | 0.010835 | 0.029443 | 0.028332 |
| K01779 | aspartate racemase [EC:5.1.1.13] | 0.024578 | 0.016253 | 0.009814 | 0.010248 |
| K22457 | asparagine---oxo-acid transaminase [EC:2.6.1.14] | 0.021301 | 0.016253 | 0.009814 | 0.010248 |
| K00264 | glutamate synthase (NADH) [EC:1.4.1.14] | 0.006554 | 0.004334 | 0.002617 | 0.002411 |
| K17761 | succinate-semialdehyde dehydrogenase, mitochondrial [EC:1.2.1.24] | 0.001639 | 0.004334 | 0.002617 | 0.002411 |
| K01948 | carbamoyl-phosphate synthase (ammonia) [EC:6.3.4.16] | 0.001639 | 0.001084 | 0.000654 | 0.000603 |
| K16871 | 4-aminobutyrate---pyruvate transaminase [EC:2.6.1.96] | 0 | 0 | 0.001963 | 0.001808 |
| K01914 | aspartate--ammonia ligase [EC:6.3.1.1] | 0 | 0 | 0.001309 | 0.001206 |
| K11541 | carbamoyl-phosphate synthase / aspartate carbamoyltransferase [EC:6.3.5.5 2.1.3.2] | 0 | 0.001084 | 0.000654 | 0.000603 |
| K14268 | 5-aminovalerate/4-aminobutyrate aminotransferase [EC:2.6.1.48 2.6.1.19] | 0 | 0.001084 | 0.000654 | 0.000603 |
| K01425 | glutaminase [EC:3.5.1.2] | 0 | 0 | 0 | 0.001808 |
| K19244 | alanine dehydrogenase [EC:1.4.1.1] | 0 | 0 | 0.000654 | 0.000603 |

| **(c)** **Glycine, serine, threonine metabolism and ectoine biosynthesis** | | | | | |
| --- | --- | --- | --- | --- | --- |
| KO IDs | Functions | 3ds | 4ds | 5ds | Sds |
| K00382 | dihydrolipoamide dehydrogenase [EC:1.8.1.4] | 0.245781 | 0.235121 | 0.233581 | 0.233889 |
| K00058 | D-3-phosphoglycerate dehydrogenase / 2-oxoglutarate reductase [EC:1.1.1.95 1.1.1.399] | 0.132722 | 0.132188 | 0.11908 | 0.119958 |
| K00928 | aspartate kinase [EC:2.7.2.4] | 0.117975 | 0.118102 | 0.119735 | 0.121164 |
| K01752 | L-serine dehydratase [EC:4.3.1.17] | 0.163854 | 0.128937 | 0.083095 | 0.084996 |
| K00600 | glycine hydroxymethyltransferase [EC:2.1.2.1] | 0.095035 | 0.098599 | 0.102723 | 0.102477 |
| K01754 | threonine dehydratase [EC:4.3.1.19] | 0.049156 | 0.08668 | 0.126932 | 0.131412 |
| K01754 | threonine dehydratase [EC:4.3.1.19] | 0.049156 | 0.08668 | 0.126932 | 0.131412 |
| K15633 | 2,3-bisphosphoglycerate-independent phosphoglycerate mutase [EC:5.4.2.12] | 0.108144 | 0.082346 | 0.06412 | 0.063295 |
| K02437 | glycine cleavage system H protein | 0.07865 | 0.066094 | 0.065429 | 0.060883 |
| K00605 | aminomethyltransferase [EC:2.1.2.10] | 0.088481 | 0.066094 | 0.04122 | 0.040388 |
| K00282 | glycine dehydrogenase subunit 1 [EC:1.4.4.2] | 0.057349 | 0.062843 | 0.037949 | 0.035566 |
| K00283 | glycine dehydrogenase subunit 2 [EC:1.4.4.2] | 0.063903 | 0.057426 | 0.034677 | 0.037374 |
| K00133 | aspartate-semialdehyde dehydrogenase [EC:1.2.1.11] | 0.034409 | 0.042257 | 0.056923 | 0.059075 |
| K01733 | threonine synthase [EC:4.2.3.1] | 0.032771 | 0.04009 | 0.05496 | 0.061486 |
| K00831 | phosphoserine aminotransferase [EC:2.6.1.52] | 0.031132 | 0.045507 | 0.047763 | 0.048224 |
| K01834 | 2,3-bisphosphoglycerate-dependent phosphoglycerate mutase [EC:5.4.2.11] | 0.049156 | 0.045507 | 0.037294 | 0.039785 |
| K01695 | tryptophan synthase alpha chain [EC:4.2.1.20] | 0.024578 | 0.035756 | 0.034023 | 0.039182 |
| K00865 | glycerate 2-kinase [EC:2.7.1.165] | 0.032771 | 0.030338 | 0.032714 | 0.034963 |
| K01079 | phosphoserine phosphatase [EC:3.1.3.3] | 0.006554 | 0.017336 | 0.03664 | 0.03436 |
| K00836 | diaminobutyrate-2-oxoglutarate transaminase [EC:2.6.1.76] | 0 | 0.017336 | 0.03664 | 0.03858 |
| K00639 | glycine C-acetyltransferase [EC:2.3.1.29] | 0.032771 | 0.02167 | 0.01832 | 0.018687 |
| K00872 | homoserine kinase [EC:2.7.1.39] | 0.034409 | 0.022754 | 0.014394 | 0.01507 |
| K00018 | glycerate dehydrogenase [EC:1.1.1.29] | 0 | 0.010835 | 0.034677 | 0.039785 |
| K00274 | monoamine oxidase [EC:1.4.3.4] | 0 | 0.019503 | 0.03206 | 0.032551 |
| K06718 | L-2,4-diaminobutyric acid acetyltransferase [EC:2.3.1.178] | 0 | 0.009752 | 0.031406 | 0.03014 |
| K10674 | ectoine hydroxylase [EC:1.14.11.55] | 0 | 0.009752 | 0.026826 | 0.027729 |
| K22305 | phosphoserine phosphatase [EC:3.1.3.3] | 0.026217 | 0.017336 | 0.010469 | 0.009645 |
| K15634 | probable phosphoglycerate mutase [EC:5.4.2.12] | 0.003277 | 0.008668 | 0.025517 | 0.024112 |
| K06720 | L-ectoine synthase [EC:4.2.1.108] | 0 | 0.006501 | 0.026826 | 0.027126 |
| K11529 | glycerate 2-kinase [EC:2.7.1.165] | 0 | 0.002167 | 0.028789 | 0.027729 |
| K00130 | betaine-aldehyde dehydrogenase [EC:1.2.1.8] | 0 | 0.003251 | 0.019629 | 0.028935 |
| K00303 | sarcosine oxidase, subunit beta [EC:1.5.3.1] | 0.01147 | 0.013002 | 0.012431 | 0.011453 |
| K16066 | 3-hydroxy acid dehydrogenase / malonic semialdehyde reductase [EC:1.1.1.381 1.1.1.-] | 0.004916 | 0.003251 | 0.015703 | 0.016879 |
| K00090 | glyoxylate/hydroxypyruvate/2-ketogluconate reductase [EC:1.1.1.79 1.1.1.81 1.1.1.215] | 0.003277 | 0.002167 | 0.013086 | 0.016276 |
| K1140 | choline dehydrogenase [EC:1.1.1.1] | 0.009831 | 0.006501 | 0.003926 | 0.004822 |
| K01753 | D-serine dehydratase [EC:4.3.1.18] | 0 | 0.002167 | 0.008506 | 0.008439 |
| K00108 | choline dehydrogenase [EC:1.1.99.1] | 0 | 0 | 0.003926 | 0.004822 |
| K01697 | cystathionine beta-synthase [EC:4.2.1.22] | 0 | 0 | 0.003926 | 0.004822 |
| K18896 | glycine/sarcosine N-methyltransferase [EC:2.1.1.156] | 0.003277 | 0.002167 | 0.001309 | 0.001206 |
| K12524 | bifunctional aspartokinase / homoserine dehydrogenase 1 [EC:2.7.2.4 1.1.1.3] | 0 | 0 | 0.003926 | 0.003617 |
| K15789 | threonine 3-dehydrogenase [EC:1.1.1.103] | 0 | 0 | 0.003271 | 0.003014 |
| K00281 | glycine dehydrogenase [EC:1.4.4.2] | 0 | 0 | 0.003271 | 0.003014 |
| K01620 | threonine aldolase [EC:4.1.2.48] | 0 | 0 | 0.002617 | 0.002411 |
| K01758 | cystathionine gamma-lyase [EC:4.4.1.1] | 0 | 0.001084 | 0.000654 | 0.001206 |
| K00302 | sarcosine oxidase, subunit alpha [EC:1.5.3.1] | 0 | 0 | 0 | 0.001206 |
| K00544 | betaine-homocysteine S-methyltransferase [EC:2.1.1.5] | 0 | 0 | 0 | 0.001206 |
| K00998 | CDP-diacylglycerol---serine O-phosphatidyltransferase [EC:2.7.8.8] | 0 | 0 | 0 | 0.000603 |

| **(d)** **Pentose phosphate pathways** | | | | | |
| --- | --- | --- | --- | --- | --- |
| KO IDs | Functions | 3ds | 4ds | 5ds | Sds |
| K00615 | transketolase [EC:2.2.1.1] | 0.139276 | 0.150607 | 0.133475 | 0.133823 |
| K00033 | 6-phosphogluconate dehydrogenase [EC:1.1.1.44 1.1.1.343] | 0.168769 | 0.140856 | 0.085057 | 0.084996 |
| K01810 | glucose-6-phosphate isomerase [EC:5.3.1.9] | 0.106505 | 0.087764 | 0.090292 | 0.090421 |
| K01783 | ribulose-phosphate 3-epimerase [EC:5.1.3.1] | 0.093397 | 0.089931 | 0.092255 | 0.092229 |
| K00948 | ribose-phosphate pyrophosphokinase [EC:2.7.6.1] | 0.086843 | 0.08343 | 0.080477 | 0.077762 |
| K01835 | phosphoglucomutase [EC:5.4.2.2] | 0.116336 | 0.092098 | 0.056923 | 0.056664 |
| K00036 | glucose-6-phosphate 1-dehydrogenase [EC:1.1.1.49 1.1.1.363] | 0.093397 | 0.071511 | 0.044492 | 0.046416 |
| K01839 | phosphopentomutase [EC:5.4.2.7] | 0.096674 | 0.071511 | 0.043183 | 0.042799 |
| K00616 | transaldolase [EC:2.2.1.2] | 0.049156 | 0.055259 | 0.070009 | 0.071131 |
| K00852 | ribokinase [EC:2.7.1.15] | 0.075373 | 0.056342 | 0.042529 | 0.047019 |
| K01807 | ribose 5-phosphate isomerase A [EC:5.3.1.6] | 0.040963 | 0.034672 | 0.049726 | 0.04943 |
| K01619 | deoxyribose-phosphate aldolase [EC:4.1.2.4] | 0.063903 | 0.046591 | 0.028134 | 0.026523 |
| K00850 | 6-phosphofructokinase 1 [EC:2.7.1.11] | 0.060626 | 0.045507 | 0.028789 | 0.028935 |
| K01621 | xylulose-5-phosphate/fructose-6-phosphate phosphoketolase [EC:4.1.2.9 4.1.2.22] | 0.049156 | 0.048758 | 0.032714 | 0.03014 |
| K03841 | fructose-1,6-bisphosphatase I [EC:3.1.3.11] | 0 | 0.031422 | 0.057577 | 0.060281 |
| K00034 | glucose 1-dehydrogenase [EC:1.1.1.47] | 0.034409 | 0.023837 | 0.019629 | 0.018084 |
| K01623 | fructose-bisphosphate aldolase, class I [EC:4.1.2.13] | 0.032771 | 0.022754 | 0.014394 | 0.014467 |
| K00874 | 2-dehydro-3-deoxygluconokinase [EC:2.7.1.45] | 0.027855 | 0.01842 | 0.016357 | 0.018084 |
| K08094 | 6-phospho-3-hexuloisomerase [EC:5.3.1.27] | 0.026217 | 0.017336 | 0.010469 | 0.011453 |
| K21071 | ATP-dependent phosphofructokinase / diphosphate-dependent phosphofructokinase [EC:2.7.1.11 2.7.1.90] | 0.021301 | 0.014086 | 0.014394 | 0.015673 |
| K01808 | ribose 5-phosphate isomerase B [EC:5.3.1.6] | 0.024578 | 0.016253 | 0.009814 | 0.012056 |
| K01625 | 2-dehydro-3-deoxyphosphogluconate aldolase / (4S)-4-hydroxy-2-oxoglutarate aldolase [EC:4.1.2.14 4.1.3.42] | 0.02294 | 0.015169 | 0.010469 | 0.011453 |
| K11529 | glycerate 2-kinase [EC:2.7.1.165] | 0 | 0.002167 | 0.028789 | 0.027729 |
| K05774 | ribose 1,5-bisphosphokinase [EC:2.7.4.23] | 0 | 0.002167 | 0.01832 | 0.016879 |
| K06151 | gluconate 2-dehydrogenase alpha chain [EC:1.1.99.3] | 0 | 0.007585 | 0.013086 | 0.016276 |
| K08093 | 3-hexulose-6-phosphate synthase [EC:4.1.2.43] | 0.014747 | 0.009752 | 0.005889 | 0.005425 |
| K00090 | glyoxylate/hydroxypyruvate/2-ketogluconate reductase [EC:1.1.1.79 1.1.1.81 1.1.1.215] | 0.003277 | 0.002167 | 0.013086 | 0.016276 |
| K01690 | phosphogluconate dehydratase [EC:4.2.1.12] | 0 | 0 | 0.001309 | 0.001808 |
| K00131 | glyceraldehyde-3-phosphate dehydrogenase (NADP+) [EC:1.2.1.9] | 0 | 0 | 0.000654 | 0.001206 |
| K00117 | quinoprotein glucose dehydrogenase [EC:1.1.5.2] | 0 | 0 | 0 | 0.001808 |
| K01057 | 6-phosphogluconolactonase [EC:3.1.1.31] | 0 | 0 | 0.000654 | 0.000603 |
| K11645 | fructose-bisphosphate aldolase, class I [EC:4.1.2.13] | 0 | 0 | 0 | 0.001206 |
| K22345 | glucosaminate ammonia-lyase [EC:4.3.1.9] | 0 | 0 | 0 | 0.000603 |

| **(e) Galactose metabolism** | | | | | |
| --- | --- | --- | --- | --- | --- |
| KO IDs | Functions | 3ds | 4ds | 5ds | Sds |
| K07407 | alpha-galactosidase [EC:3.2.1.22] | 0.306407 | 0.273043 | 0.16488 | 0.156127 |
| K00845 | glucokinase [EC:2.7.1.2] | 0.180239 | 0.127854 | 0.088983 | 0.089215 |
| K01784 | UDP-glucose 4-epimerase [EC:5.1.3.2] | 0.114698 | 0.127854 | 0.105995 | 0.101874 |
| K00963 | UTP--glucose-1-phosphate uridylyltransferase [EC:2.7.7.9] | 0.119613 | 0.117019 | 0.10076 | 0.097052 |
| K01785 | aldose 1-epimerase [EC:5.1.3.3] | 0.131083 | 0.109434 | 0.066083 | 0.062692 |
| K01835 | phosphoglucomutase [EC:5.4.2.2] | 0.116336 | 0.092098 | 0.056923 | 0.056664 |
| K01190 | beta-galactosidase [EC:3.2.1.23] | 0.114698 | 0.075845 | 0.048417 | 0.045813 |
| K01635 | tagatose 1,6-diphosphate aldolase [EC:4.1.2.40] | 0.098312 | 0.081263 | 0.049072 | 0.04521 |
| K00965 | UDPglucose--hexose-1-phosphate uridylyltransferase [EC:2.7.7.12] | 0.088481 | 0.079096 | 0.051689 | 0.04943 |
| K01193 | beta-fructofuranosidase [EC:3.2.1.26] | 0.101589 | 0.071511 | 0.043183 | 0.040991 |
| K01187 | alpha-glucosidase [EC:3.2.1.20] | 0.085204 | 0.068261 | 0.044492 | 0.043402 |
| K00849 | galactokinase [EC:2.7.1.6] | 0.057349 | 0.053092 | 0.034023 | 0.033154 |
| K01182 | oligo-1,6-glucosidase [EC:3.2.1.10] | 0.057349 | 0.054175 | 0.032714 | 0.033154 |
| K01854 | UDP-galactopyranose mutase [EC:5.4.99.9] | 0.060626 | 0.052008 | 0.031406 | 0.028935 |
| K00850 | 6-phosphofructokinase 1 [EC:2.7.1.11] | 0.060626 | 0.045507 | 0.028789 | 0.028935 |
| K00917 | tagatose 6-phosphate kinase [EC:2.7.1.144] | 0.060626 | 0.04009 | 0.024209 | 0.022304 |
| K01684 | galactonate dehydratase [EC:4.2.1.6] | 0.042602 | 0.039006 | 0.023554 | 0.021701 |
| K12111 | evolved beta-galactosidase subunit alpha [EC:3.2.1.23] | 0.040963 | 0.027088 | 0.016357 | 0.01507 |
| K01819 | galactose-6-phosphate isomerase [EC:5.3.1.26] | 0.036048 | 0.023837 | 0.014394 | 0.013262 |
| K02746 | PTS system, N-acetylgalactosamine-specific IIC component | 0.014747 | 0.013002 | 0.007851 | 0.007234 |
| K02745 | PTS system, N-acetylgalactosamine-specific IIB component [EC:2.7.1.-] | 0.014747 | 0.009752 | 0.005889 | 0.005425 |
| K02747 | PTS system, N-acetylgalactosamine-specific IID component | 0.008193 | 0.005418 | 0.003271 | 0.003014 |
| K02744 | PTS system, N-acetylgalactosamine-specific IIA component [EC:2.7.1.-] | 0.006554 | 0.004334 | 0.002617 | 0.002411 |
| K07406 | alpha-galactosidase [EC:3.2.1.22] | 0 | 0.004334 | 0.002617 | 0.002411 |
| K00094 | galactitol-1-phosphate 5-dehydrogenase [EC:1.1.1.251] | 0.001639 | 0.001084 | 0.000654 | 0.000603 |
| K00011 | aldehyde reductase [EC:1.1.1.21] | 0 | 0.001084 | 0.000654 | 0.001206 |
| K02774 | PTS system, galactitol-specific IIB component [EC:2.7.1.200] | 0 | 0 | 0 | 0.000603 |
